# Elucidating Ligand‐Dependent Selectivities in Pd‐Catalyzed C–H Activations

**DOI:** 10.1002/anie.202507500

**Published:** 2025-10-23

**Authors:** Fritz Deufel, Monika Ravi, Manuel van Gemmeren

**Affiliations:** ^1^ Otto Diels‐Institut für Organische Chemie Christian‐Albrechts‐Universität zu Kiel Otto‐Hahn‐Platz 4 24118 Kiel Germany

**Keywords:** Catalysis, Selectivity prediction, C─H activation, Dispersion, Mechanism

## Abstract

Achieving regioselectivity in C–H activation remains a major challenge. Controlling or even reversing the regioisomeric outcome through the choice of ligand is even harder to achieve, let alone to predict. In this study, we investigated ligand effects in the regioselective Pd‐catalyzed alkynylation of thiophenes using multivariate linear regression to build predictive models that offer deeper insight into the structural factors driving regioselectivity and enabled the discovery of a more selective ligand. Combining experimental and DFT studies, we propose a Curtin–Hammett scenario between the C–H activation and migratory insertion as the origin of the ligand‐controlled selectivities. A detailed investigation of solvent effects uncovered an inadequate description of solvent‐solute dispersion interactions by implicit solvation models. The crucial role of the solvent in substrate coordination is further evidenced by an inverse solvent kinetic isotope effect. Additionally, the often poorly understood role of silver was scrutinized, showing that it serves to mitigate a detrimental side reaction. This study provides generalizable insights for the computational description of challenging regio‐ and stereoselectivities and is expected to aid future mechanistic investigations in Pd‐catalyzed C–H activation that holistically consider the role of all reaction components.

## Graphical abstract

Strategies to understand ligand effects on regioselectivities of C–H activation processes were investigated using an alkynylation of thiophenes as a model system. The results shed light on the role of dispersion models for the correct prediction of reaction outcomes and propose new roles for the solvent and silver additive. The utility of multivariate linear regression in predicting selectivities across mechanistic regimes is demonstrated.

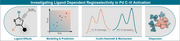

## Introduction

C─H activation generally faces two major challenges: reactivity and selectivity. Nondirected C─H activations, especially when substrate‐limited, lack the thermodynamic and kinetic edge provided by directing groups and therefore often suffer from low reactivity. Furthermore, for substrates without a strong electronic or steric bias, the presence of multiple energetically similar C─H bonds renders the selectivity challenging to control.^[^ ^1^, ^2^ ^]^

Addressing these challenges requires the design of highly active and selective catalysts as well as a fine‐tuning of the reaction conditions. Ideally, one would like to identify several sets of catalysts and conditions, each allowing the selective functionalization of a molecule in one of several competing positions with full catalyst control.

In the absence of directing groups, the selectivity is often governed by the electronic properties of the competing C─H bonds. For instance, electron‐rich phenol derivatives are typically functionalized in para and, for small substituents on oxygen, ortho‐position, although some sterically sensitive catalysts deliver meta:para mixtures.^[^ ^3^ ^]^

**Figure 1:**
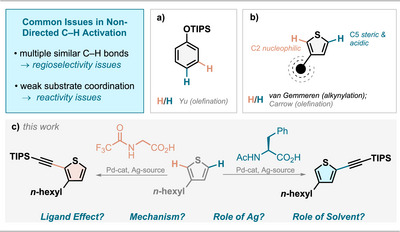
Literature background and ligand‐controlled selectivity studied herein.

To date, only a few examples are reported, where a complete switch in selectivity is based only on the nature of the ligand rather than the use of a different strategy altogether. A prominent example is the switch between meta‐ and para olefination of aromatic silyl ethers by Yu (Figure 1).^[^ ^4^ ^]^ Toward the functionalization of heteroarenes,^[^ ^5^, ^6^ ^]^ Carrow reported a catalyst‐controlled regioselective olefination.^[^ ^7^, ^8^ ^]^ Unlike these two examples, where completely different ligand classes are employed to access the competing regioisomers, De Vos, Jiao, and Chang independently reported switchable selectivity in indoles arising from different ligands.^[^ ^9^, ^10^, ^11^ ^]^ Recently, we reported a regioselective alkynylation of thiophenes, where under otherwise identical reaction conditions, high levels of regioselectivity for either the C2 or C5 position could be achieved, solely through the choice of different *N*‐acyl amino acid ligands.^[^ ^12^, ^13^ ^]^

The moderate to good selectivities in all of these systems indicate rather small energy differences (1–2 kcal mol^−1^) between the product‐forming pathways. We realized that rational insights into these subtle ligand effects could lead to improved catalysts and would potentially be applicable to other arene functionalizations where the regioselectivity still requires improvement.^[^ ^3^ ^]^ Considering that for C─H activations the mechanistic understanding often lags far behind the synthetic utility of such transformations, we became interested in understanding the factors governing the regioselectivity in nondirected C─H activations. We selected our regioselective thiophene alkynylation as a model system and systematically probed various approaches to identify the regioselectivity‐controlling factors.

## Results and Discussion

### Selectivity Prediction via MLR

Designing selective ligands is often difficult since multifactorial ligand effects on reactivity and selectivity are challenging to evaluate for humans simultaneously. Often, very subtle effects need to be exploited since energy differences as small as 1–3 kcal mol^−1^ can cause a reversal of selectivity. Predicting these changes in silico remains challenging even in the advent of modern computational methods, since a very sound understanding of ligand effects and the overall reaction mechanism is required.

**Figure 2:**
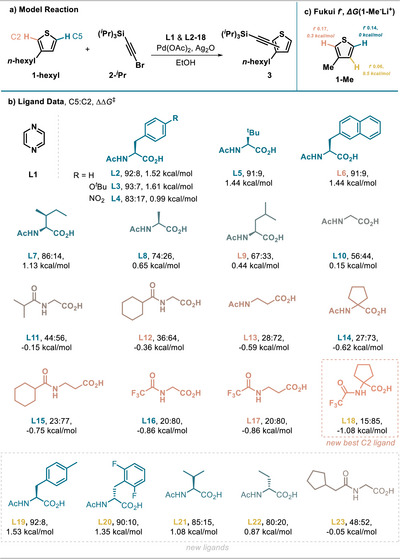
a) Model reaction for thiophene alkynylation with 0.1 mmol **1‐hexyl**, **2‐Si*^i^* Pr_3_** (1.5 equiv), Pd(OAc)_2_ (10 mol%), Ag_2_O (2.0 equiv), **L1** (20 mol%), **L2**‐**18** (30 mol%), EtOH (1 mL), 40 °C, 18 h. b) Overview of the ligands and C5:C2 selectivities. Ligand numbers **L** in blue, orange, or yellow indicate ligands for modeling as train‐set, test‐set, and newly acquired test‐set data beyond the original study. c) Fukui nucleophilicity index *f* ^−^(*r*) and free energy differences (ωB97X‐D3BJ/def2‐TZVP) for lithiated thiophene **1**‐Me for C2, C5, and C4 positions.

With information from a previous ligand screening at hand (Figure 2), we investigated whether data science is suited to predict the selectivity in our small dataset. Data science techniques have already been applied to N‐acyl amino acid ligands by Sigman,^[^ ^14^ ^]^ Yu,^[^ ^15^ ^]^ Ackermann,^[^ ^16^ ^]^ and Sunoj^[^ ^17^ ^]^ for predicting enantioselectivity in C─H functionalization reactions. To date, only a few studies have investigated the ligand/additive‐dependent regioselectivity in C─H activation using data‐driven modelling.^[^ ^10^, ^18^ ^]^ To the best of our knowledge, regioselectivities controlled by *N*‐acyl amino acid ligands have not been analyzed with data science methods to date, motivating us further to probe the feasibility of this approach. We reasoned that multivariate linear regression (MLR) would be a suitable tool, since it is operationally easy, lightweight, and less prone to overfitting in small dataset regimes than common machine learning (ML) models.

**Figure 3:**
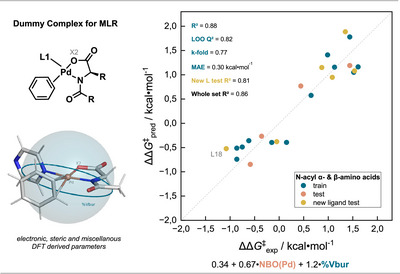
MLR analysis of the full dataset of *N*‐acyl α‐ and β‐amino acids with the two‐parameter equation.

Additionally, our dataset evenly covers a broad range of experimental selectivities, making it well‐suited for MLR, which enables a direct interpretation of the key parameters and thus molecular effects.^[^ ^19^, ^20^, ^21^ ^]^To compute the required descriptors, we chose a model compound that features an *N*‐acyl amino acid ligand coordinated to Pd, the additional pyrazine ligand **L1,** and benzene (Figure 3). Benzene was chosen as a dummy substituent at this stage, as it reduces the number of conformers compared to thiophene but offers a similar steric environment, allowing us to efficiently obtain meaningful descriptors. Several steric and electronic parameters were evaluated at the PBE0‐D3(BJ)/def2‐TZVP level of theory in the gas phase (see Supporting Information for details).

A two‐parameter model was obtained for the full dataset containing N‐acyl α‐ and β‐amino acids with reasonable stats (Figure 3, see Supporting Information for full statistics). Using normalized parameters, the magnitude of the prefactor indicates the importance of the respective factor in controlling the reaction outcome. Most models obtained in our MLR analysis feature a strong steric parameter, namely the buried volume (%Vbur) and an electronic parameter, the most robust model pairing the %Vbur with the NBO charge on Pd. The MLR model indicates that a more electrophilic Pd catalyst would favor C2 selectivity and more steric bulk would favor C5 selectivity.

To probe the generalizability beyond the test set, several new ligands beyond the original study were evaluated experimentally and computationally. More accurate predictions can be reached when limiting the analysis to only α‐amino acid‐derived ligands (*R* ^2^ = 0.93, k‐fold = 0.85, MAE = 0.19 kcal mol^−1^, see dataset A, Supporting Information). This can be justified by the fact that the ring size in palladacycles has been reported to significantly alter reactivity.^[^ ^22^, ^23^ ^]^ In the α‐amino acid‐derived dataset, a more refined electronic parameter of the Pd‐binding carboxylate oxygen NBO(X2) gives improved out‐of‐sample predictability for the test/train/new ligand test split (*R* ^2^ = 0.96, MAE of 0.15 kcal mol^−1^ for new ligand test set). Even the newly synthesized ligand **L18** was predicted well in this more focused dataset, which showcases the utility of the MLR approach for out‐of‐sample ligand prediction (*N*‐acyl α‐amino acids: see Figure S9b calc. −0.99 kcal mol^−1^ versus exp −1.07 kcal mol^−1^). This ligand was not contained in the original study and constitutes now the most C2‐selective ligand for our transformation.

### Initial Mechanistic Hypothesis

Achieving C2 selectivity in thiophenes is possible using electrophilic catalysts since this is the most nucleophilic position, as evidenced by the highest *f*‐ Fukui index (Figure 2a). The C5 position is the sterically most accessible and most acidic position, as evidenced by the highest relative stability (ΔG **1‐Me^−^** Li^+^) of lithiated **1** (Figure 2a).

We initially hypothesized that the selectivities in our system could originate from ligand control in a selectivity‐determining C─H activation step.

Accordingly, as our *hypothesis 1* we postulated that the C2 selective reaction might be due to an electrophilic nature of the C─H activation step whereas the C5 selective reaction would proceed via a C─H activation favoring the more acidic position. This hypothesis could be probed using a More O'Ferrall‐Jencks^[^ ^24^, ^25^ ^]^ diagram, which indicates the degree of C─H bond cleavage and C─Pd bond formation in the respective TS and allows the distinction of several mechanistic scenarios.^[^ ^26^, ^27^, ^28^ ^]^ Originally meant to describe the entirety of concerted pathways,^[^ ^29^, ^30^ ^]^ the terms concerted metalation‐deprotonation (CMD) and ambiphilic metal ligand activation (AMLA) are nowadays more commonly used to refer to regimes, in which C─H cleavage is more advanced than C─Pd bond formation and consequently the most acidic position is more readily activated. This scenario was computed to be operative for thiophenes using electron‐rich phosphine ligands or using silver as the catalytic metal.^[^ ^27^ ^]^ On the other hand, base‐assisted internal electrophilic substitution (BIES) or electrophilic concerted metalation‐deprotonation (eCMD) can be identified, where the C─Pd bond formation is more advanced than C─H bond cleavage and consequently the most electron‐rich position is functionalized. This therefore yields similar selectivities to classical S_E_Ar reactions.^[^ ^26^ ^]^ These mechanistic regimes are separated by a region with (virtually) synchronous pathways, where neither electrophilic nor nucleophilic effects are predominant, and hence steric effects can strongly influence the selectivity as shown in our previous work.^[^ ^31^ ^]^

**Figure 4:**
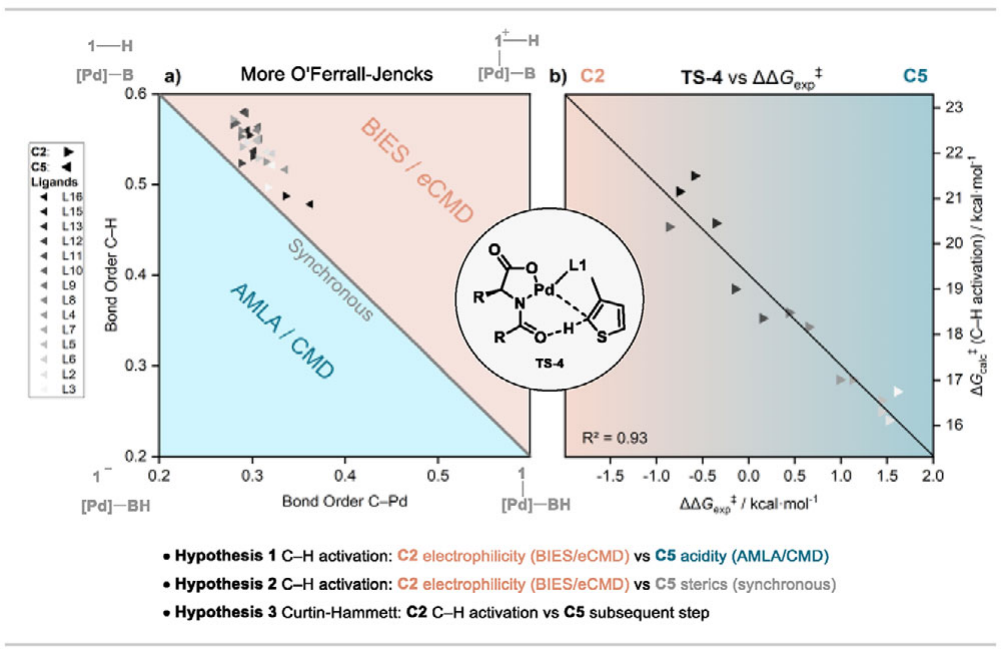
a) More O'Ferrall‐Jencks plot for the C─H activation step of **L2**‐**L16** with Wiberg bond orders (ωB97X‐D3BJ/def2‐TZVP). b) Experimental selectivities as a function of the calculated C─H activation barrier (SMD(EtOH) PWPB95‐D3(BJ)/def2‐QZVP// ωB97X‐D3BJ/def2‐TZVP).

To probe *hypothesis 1*, several TS for all N‐acyl amino acid‐derived ligands were computed and plotted in a More O'Ferrall‐Jencks diagram (Figure 4a). 3‐Methyl thiophene (**1‐Me**) was used for the computations, as it displays less conformational flexibility than 3‐hexyl thiophene (**1‐hexyl**) and experimentally displays a similar selectivity reversal. As can be seen from Figure 4a, our initial *hypothesis 1* could be refuted, since all ligands promote C─H activation via a TS in the eCMD/BIES regime, irrespective of the experimentally observed selectivities.

Since the C5‐isomeric TS are rather close to the diagonal of the More O'Ferrall‐Jencks diagram, we formulated *hypothesis 2*: the C2 selective reaction might be due to a more electrophilic C─H activation step, whereas the C5 selective reaction would proceed via a synchronous path and hence be controlled by steric effects.^[^ ^31^, ^32^ ^]^ However, we noted that the calculated relative energy differences between C2‐ and C5‐forming TS isomers (ΔΔ*G* ^‡^) indicate a preference for the C─H activation in the C2 position in all cases. This implies that the observed ligand‐controlled selectivity switch cannot be explained by the selectivity of the C─H activation step alone, and consequently, *hypothesis 2* could also be ruled out.

Interestingly, we observed a strong correlation (*R* ^2^ = 0.93) of the experimental ΔΔ*G* ^‡^ (*cf*. Figure 2) with the calculated C─H activation barrier Δ*G* ^‡^ in the C2 position (Figure 4b), meaning that the higher the C─H activation barrier, the more C2 product was observed. This demonstrated that while not being the sole factor, the C─H activation step is decisively involved in controlling the regioselectivity.

**Figure 5:**
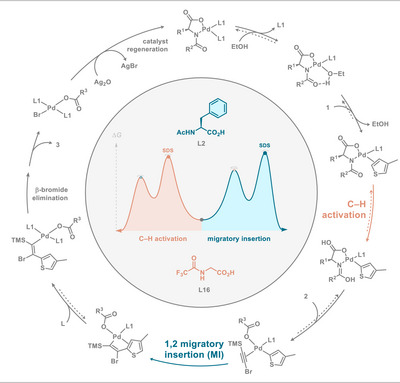
Tentative catalytic cycle.

We therefore formulated *hypothesis 3*: a Curtin–Hammett scenario^[^ ^33^ ^]^ involving a reversible C─H activation and a subsequent step could be operative (Figure 5). Depending on the ligand either step could become selectivity‐determining. A higher barrier in the C2 selective C─H activation step would make this step selectivity‐determining, since the subsequent barriers would now be lower in comparison. In contrast, a lower barrier would render this step reversible and impose C5 selectivity with this position being favored in the subsequent, now selectivity‐determining step. Carrow proposed a similar scenario for their thiophene olefination.^[^ ^8^ ^]^ As indicated in Figure 5, we expected that either the C2 selective C─H activation or the C5‐selective migratory insertion (MI) of the alkyne could impose the selectivity. Paton^[^ ^34^ ^]^ and Musaev^[^ ^35^, ^36^ ^]^ have shown that alkynyl bromides similar to **2** preferentially react via a 1,2‐MI.

With *hypothesis 3* and the mechanism from Figure 5 in mind, we proceeded to scrutinize the barriers for the MI step with various ligands and bromo(trimethylsilyl)acetylene (**2‐SiMe_3_**). Surprisingly, our calculations indicated a preference for the C2 position with **L2**, an experimentally C5‐selective ligand. We probed different conformers, the involvement of silver, and different ligand orientations, but a preference of C2 over C5 was always pronounced. To probe whether this is a computational artefact, several implicit solvation models (CPCM, SMD, openCOSMORS, dCOSMORS) were tested, with different dispersion corrected DFT functionals and CCSD(T) with complete basis set (CBS) extrapolation. All calculations pointed consistently toward C2 (see Supporting Information for further discussion).

After extensively investigating alternate reaction pathways, like an oxidative addition (Pd^II^/Pd^IV^), as suggested for alkynyl iodides,^[^ ^35^ ^]^ or an oxidative addition of alkynyl bromide with an intermittently formed Pd^0^ species, followed by a Heck‐like insertion of the thiophene,^[^ ^37^, ^38^ ^]^ we concluded that these alternatives are energetically unfavorable (see Supporting Information for further details).

### Mechanistic Experiments

**Figure 6:**
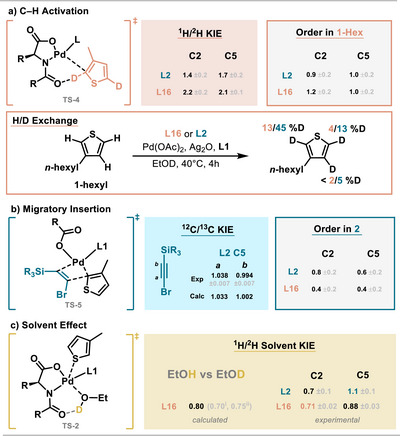
Experimental investigation of key steps with **L2** and **L16**. Calculations at the ωB97X‐D3BJ/def2‐TZVP level. a) Parallel ^1^H/^2^H‐KIE of **1‐hexyl** and 2,5‐*d*_2_‐**1‐hexyl** and order determination for **1‐hexyl** and solvent H/D exchange under standard conditions. b) ^12^C/^13^C‐KIE determination for **2‐Si*^i^* Pr_3_** and comparison with calculated **TS‐5**‐AcO‐SiMe_3_; order determination for **2‐Si*^i^* Pr_3_** at low and high concentrations. c) Inverse ^1^H/^2^H solvent KIE with EtOH/D and comparison with calculated values. ^i^ TPSS‐D3(BJ)/def2‐SVP, ^ii^ PBE0‐D3(BJ)/def2‐TZVP.

To further probe *hypothesis 3* and gain insights into the step(s) besides C─H activation influencing the regioselectivity, we acquired experimental mechanistic data. We selected **L2** and **L16** for our experimental and more detailed computational endeavors and determined the parallel kinetic isotope effects (KIE). Reliable competition KIE data were impossible to obtain for **L2** since significant isotope exchange with the solvent occurred. This phenomenon has previously been observed and was exploited in our (hetero)arene deuteration methodology.^[^ ^39^, ^40^, ^41^, ^42^ ^]^ H/D exchange occurs preferentially in C2 position in the absence of **2**‐**Si*^i^* Pr_3_** for both ligands and with higher overall deuteration degrees for **L2** indicating that a) a reversible C─H activation occurs with both ligands b) the C─H activation is C2 selective for both ligands and c) the barrier for C─H activation is higher for **L16** than for **L2** (Figure 6a).

Consequently, subsequent parallel KIE experiments were conducted in EtOD to avoid misleading results (Figure 6a). The KIEs (1.4–2.1) are larger than the maximum for a secondary KIE. Values for **L2** are smaller than for **L16** and comparably small for a primary KIE.^[^ ^43^, ^44^, ^45^ ^]^

The presence of a second step with a similar or higher barrier on the product‐forming pathway would explain the truncation of the primary KIE in the C─H activation step. This step is expected to be higher in the case of **L2**.

To get more insights regarding the second selectivity‐controlling step, we determined the kinetic orders in thiophene and alkyne for **L2** and **L16** using variable time normalization analysis.^[^ ^46^, ^47^ ^]^ Near unity orders in **1‐hexyl** were observed in both cases. Lower orders in **2‐Si*^i^* Pr_3_** in **L16** compared to **L2** were observed (Figure 6b). These orders indicate that both reactions feature a high‐energy TS for the C─H activation step involving thiophene. Since this step does not involve the alkyne,^[^ ^48^ ^]^ a second TS featuring the alkyne must be close in energy. Non‐unity (MI turnover‐limiting) and non‐zero (C─H activation turnover‐limiting) orders are expected when barriers are close in energy (see elasticity coefficient discussion in the SI).^[^ ^48^ ^]^ We also determined ^12^C/^13^C KIEs for the reaction involving **L2** to compare them with our calculated TS and observed an inverse solvent kinetic isotope effect (Figure 6b,c). These results proved helpful in interpreting our later computational results and are discussed in more detail there.

### Investigation of Dispersion Attenuation

The experimental results are in line with a Curtin–Hammett‐like scenario involving a C5 selective MI, prompting us to further question the C2 selectivity predicted by our computed TS. Due to the similarity of the system, we turned our attention to the results reported by Carrow.^[^ ^8^ ^]^ In Carrow's case, the PCM BP86/6‐31G* basis set and functional for thermochemistry and single point energy reproduce their experimental findings well, despite the generally inadvisable choice of a rather small basis set and the omission of a dispersion correction.^[^ ^49^ ^]^ Unfortunately, no detailed discussion regarding the choice of computational methodology was provided. We reoptimized and reinvestigated the structures from Carrow's work and noted that upon using higher‐level methods, including CCSD(T), the C5 selectivity is not correctly predicted anymore. The main factor leading to a correct prediction of the C5 selectivity with BP86/6–31G* was the absence of a dispersion correction.

Indeed, we found that if the dispersion correction is omitted (see the Supporting Information for more details), our computations also predict the experimentally observed C5 selectivity via **TS‐5**‐AcO, while dispersion corrected DFT results could not predict the experimental C5 selectivity correctly. This effect is even more pronounced for 3‐phenyl thiophene (**1‐Ph**). Here, C2 is predicted to be highly favorable (ΔΔ*G* ^‡^_calc_ = −3.9 kcal mol^−1^ in the MI step), contrasted by the experimental ΔΔ*G* ^‡^_exp_(**L2**) = 1.6 kcal mol^−1^. The computations for the C─H activation step also overestimate the C2 selectivity (ΔΔ*G* ^‡^_calc_ = −5.2 kcal mol^−1^) compared to the experimental ΔΔ*G* ^‡^_exp_(**L16**) = −1.3 kcal mol^−1^ at the SMD(EtOH) ωB97X‐D3BJ/def2‐TZVP//ωB97X‐D3BJ/def2‐TZVP level of theory.

This is surprising, since generally speaking, DFT‐D3 methods outperform conventional DFT functionals without dispersion corrections in most benchmark tests.^[^ ^49^ ^]^ Since the CCSD(T) results also contradict the experimental observations, we investigated the nature of the dispersion interactions in more detail to get “the right answer for the right reasons”. We hypothesized that different levels of intramolecular dispersion interaction could artificially overstabilize the TS, leading to the C2 isomer. Using the local energy decomposition (LED)^[^ ^50^ ^]^ scheme with DLPNO‐CCSD(T)/def2‐TZVP, one can decompose the binding energy into several contributions, like non‐dispersion, dispersion, geometric preparation, etc. It is thus possible to determine whether an electronic preference for one isomer competes with dispersion interactions favoring the other isomer.

**Figure 7:**
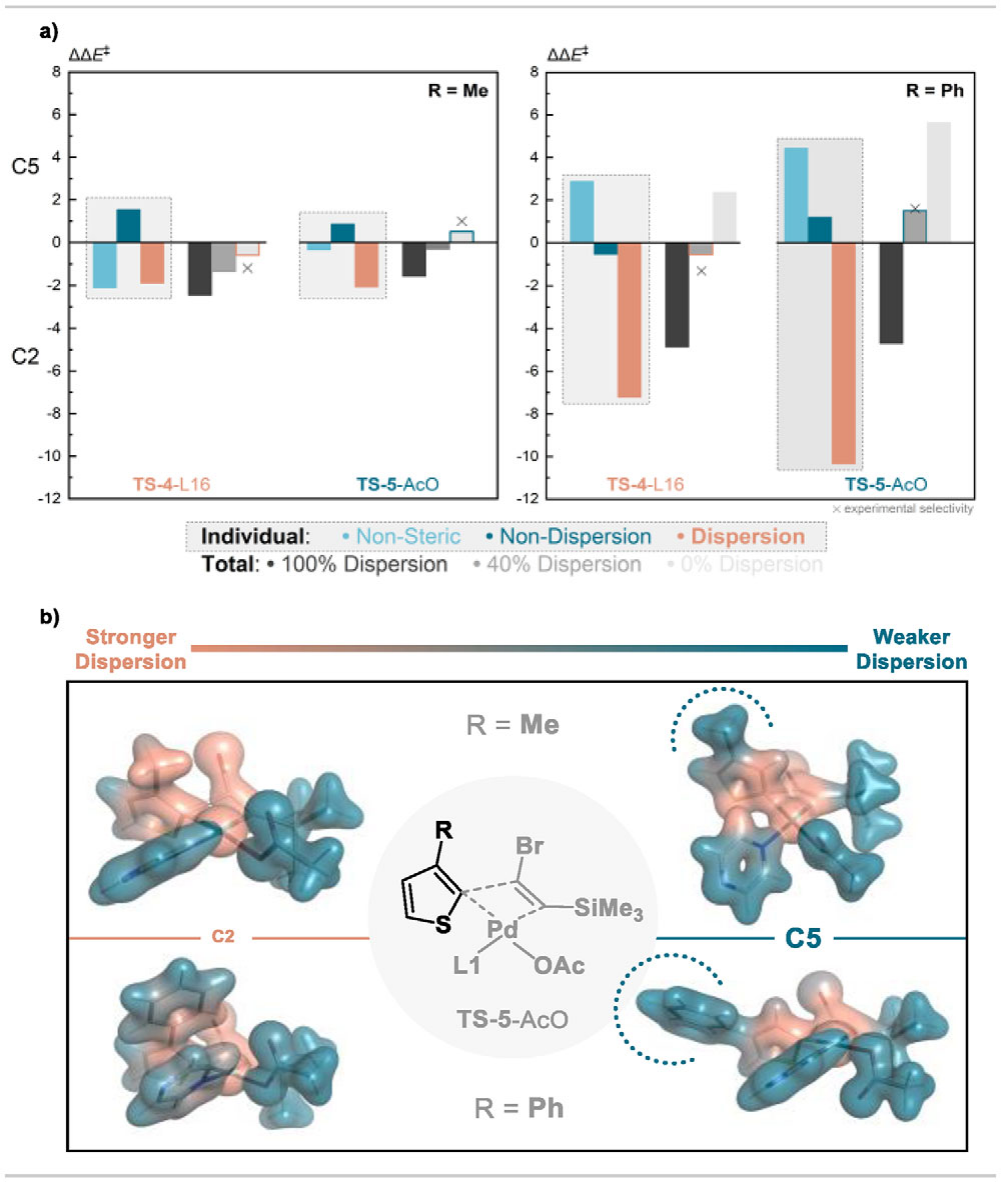
a) Comparison of the relative ΔΔ*E* ^‡^ (C2 versus C5) in kcal mol^−1^ of different decomposed contributions from the LED analysis at the DLPNO‐CCSD(T)/def2‐TZVPP level of theory. b) Dispersion interaction density mapped on the electron density for **TS‐5**‐AcO for C2 and C5 for **1‐Me** and **1‐Ph**.

We performed this analysis for the C─H activation step with **L16** and the MI step with acetate (see Supporting Information for other ligands). The results show that for both **1‐Me** and **1‐Ph**, the dispersion effect favors C2 in the MI step (Figure 7a). Especially the non‐dispersion effects in the case of **1‐Me** and the non‐steric effects in the case of **1‐Ph** favor C5.

Notably, the substituent in the C3 position points outwards during the C5‐functionalization pathway and toward the Pd‐complex during the C2‐functionalization pathway. In the case of C2 functionalization, intramolecular dispersion interactions can stabilize the intermediates and TS relative to the respective C5 isomers. In vacuo, this is a realistic behavior, but in solution, solvent‐solute interactions are expected to a) truncate these intramolecular dispersion interactions and b) stabilize the C5 isomers by solvent‐solute interactions. This can be visualized by inspecting the dispersion interaction densities (Figure 7b), where the different levels of intramolecular dispersion interactions with the C3 substituent are clearly visible.

In the end the C2 versus C5 selectivity depends on the competing isomeric TS rather than the respective ground states (GS). For solution GS conformers determined by vibrational circular dichroism, it has already been discussed that implicit solvation models can perform poorly especially when neglecting crucial explicit solvent interactions.^[^ ^51^, ^52^ ^]^ It was found that omitting dispersion corrections led to superior geometric results^[^ ^53^ ^]^ or reaction enthalpies for ligand coordination reactions.^[^ ^54^ ^]^ This is likely due to the poor performance of the implicit solvation models rather than the dispersion correction itself.^[^ ^55^ ^]^ Several models,^[^ ^56^, ^57^, ^58^, ^59^, ^60^, ^61^ ^]^ often termed molecular balance, have been developed to quantify the attenuation of dispersion interactions due to solvent‐solute interactions. Experimentally, Chen showed an attenuation of intermolecular dispersion interactions in dichloromethane^[^ ^62^ ^]^ and other solvents of up to 70% compared to predicted theoretical and gas phase values.^[^ ^63^ ^]^

Overall, **1‐Ph** has a larger dispersion contribution stabilizing C2 (7–10 kcal mol^−1^) compared to **1‐Me** (∼2 kcal mol^−1^). A full attenuation of intramolecular dispersion interactions in the case of **1‐Me** for **TS‐4** and **TS‐5** results in experimentally correct selectivities. The predicted C5 selectivity for the MI is now in agreement with experiment, and the C2 selectivity in the C─H activation TS is not overestimated anymore. In the case of **TS‐4‐L16** for **1‐Me,** the non‐steric factors such as electronic effects favor C2 selectivity, whereas in the case of **TS‐5**‐AcO, mainly non‐dispersion effects such as steric repulsion favor the C5 selectivity. In addition to solvent‐solute effects, an overestimation of alkyl dispersion interaction could play an additional minor role.^[^ ^64^ ^]^

A lower degree of dispersion attenuation for **1‐Ph** is expected due to the larger nature of the substituent and the overall larger dispersion contribution. It is reasonable to assume that a certain amount of dispersion will not be truncated, and some intramolecular dispersion persists. A 60% attenuation of dispersion interaction in the case of **1‐Ph** yields qualitatively good results for the MI and C─H activation step (Figure 7a, framed boxes). Upon using non‐dispersion corrected structures, larger basis sets, other carboxylates, and implicit solvation models, the precise values for attenuation might change, but the overall effect is expected to be identical and to show a reasonable qualitative agreement with experiment.

The workflow above enables an estimation of how much truncation of dispersion interaction is required for an agreement with experiment, and shows if other factors can be used to predict the regioselectivity correctly.

A conceptual alternative would be to study the system with explicit solvation. However, obtaining quantitatively meaningful results with explicit solvation is very challenging since extensive conformational sampling of all solvent molecules is required, and many additional solvent molecules are required for adequate solvent‐solute interactions, thus rendering the system prohibitively large for long QM/MD simulations and high‐level DFT methods. We nevertheless carried out preliminary calculations with explicit solvation for **1‐Ph** (see Supporting Information), which qualitatively corroborate the conclusion that the C5 isomer is preferred in the MI step (**TS‐5**).

We concluded that an attenuation of intramolecular dispersion interactions due to solvent‐solute dispersion interaction is likely operative in our system, since a) alternate pathways explaining the observed selectivities were probed and found to have prohibitively high barriers, b) literature reports show ground state dispersion attenuation in related systems, c) an attenuation was shown to predict the correct isomer in case of the MI **TS‐5** and improve the agreement with the observed selectivity in the C─H activation **TS‐4**, and d) preliminary calculations with explicit solvation corroborate to this interpretation. This study constitutes a rare report, in which an attenuation of dispersion interactions on the TS was probed as a chemically consistent explanation for qualitatively correctly predicting regioselectivities.

### Detailed Mechanistic Picture

**Figure 8:**
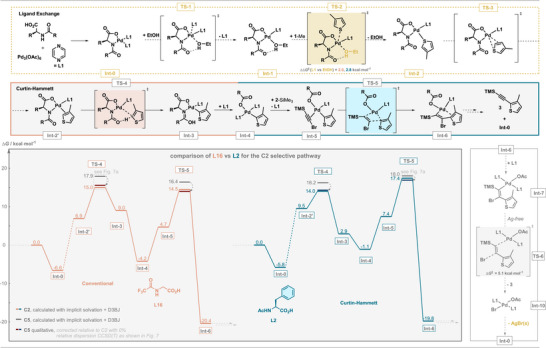
Free energy pathways for the formation of C2 product at the dCOSMORS(EtOH) ωB97X‐D3BJ/def2‐QZVPP//ωB97X‐D3BJ/def2‐TZVP level of theory for the initial C─H activation and MI for **L2** and **L16** (grey background). Proposed structures for the ligand exchange and product liberation are shown (white background).

To verify if the suggested Curtin–Hammett‐like scenario is indeed responsible for the regioselective C─H functionalization, the full reaction pathway was investigated using DFT.^[^ ^65^, ^66^, ^67^, ^68^, ^69^ ^]^ Several functional combinations were benchmarked, and dCOSMORS (EtOH) ωB97X‐D3BJ/def2‐QZVPP//ωB97X‐D3BJ/def2‐TZVP was selected, as it accurately predicts experimental results and is most in line with DLPNO‐CCSD(T)/CBS results (Figure 8). The calculations indeed confirm a higher barrier for the C─H activation (**TS‐4**) when using **L16** and a higher barrier for the MI (**TS‐5**) with **L2**. This is in line with a Curtin–Hammett‐like scenario, in which the C─H activation is reversible for **L2** and only the higher barrier imposes the selectivity. The MI is exergonic and irreversible (Δ*G* ^‡^ > 30 kcal mol^−1^ for the reverse reaction). The MI is followed by an additional **L1** coordination (**Int‐7**), a β‐bromide elimination (**TS‐6**), bromide coordination, and product liberation (**Int‐10**).

The possibility of an inverse MI and a subsequent silyl migration, as investigated by Paton,^[^ ^34^ ^]^ was also evaluated. The comparison of calculated and experimental ^12^C/^13^C KIEs with **L2** allowed us to exclude this scenario. As outlined before, implicit solvation fails to qualitatively predict the relative barriers in **TS‐5** when combined with CCSD(T) or DFT‐D3. We therefore corrected the C5 barriers relative to the DFT calculated C2 barriers using the CCSD(T) dispersion‐attenuated relative energies from Figure 7a to give a qualitative overview of the reaction pathway in Figure 8.

During the KIE studies, we noted that reactions using either **L2** or **L16** both feature an inverse solvent KIE (Figure 6c) in EtOH/D.^[^ ^70^, ^71^, ^72^ ^]^ The effect is more pronounced for **L16** and in both cases more for C2 than for C5. Explicit coordination of EtOH in **TS‐4** was probed and found unfavorable. It seemed reasonable that upon ligand decoordination from the resting state (**Int‐0**), an intermediate EtOH coordination to the metal would take place, giving **Int‐1**. The subsequent ligand exchange from EtOH to substrate would then give rise to an inverse solvent KIE. Maiti suggested a similar coordination of HFIP as the resting state in two directed N‐acyl amino catalyzed reactions.^[^ ^73^, ^74^ ^]^ In our case, the EtOH coordination is endergonic compared to the resting state (**Int‐0**), which resembles the one proposed in our previous experimental mechanistic study.^[^ ^31^ ^]^

Comparing the relative barriers for associative and dissociative ligand exchange pathways, the formation of **Int‐2** was indeed found to be more favorable through the intermediacy of **Int‐1** rather than directly from **Int‐0** (2.0 kcal mol^−1^ for **L16** and 2.8 kcal mol^−1^ for **L2**). We calculated the KIEs and found that it is in agreement with the experimentally observed values (Figure 6b). The reaction orders suggest that **TS‐4** or **TS‐5** are indeed turnover‐limiting, but **TS‐2**, **TS‐4**, and **TS‐5** are all energetically similar, as evidenced by the KIEs for **1‐hexyl**, **2‐Si*^i^* Pr_3_**, and EtOH/D (Figure 6). The results explain the experimental observations well, but should be interpreted taking into consideration the fundamentally challenging nature of this system. Even high‐level methods with “chemical accuracy” show errors of at least 1 kcal mol^−1^ in the gas phase, and additional errors due to implicit solvation models (e.g., errors of 2–3 kcal mol^−1^ for COSMO‐RS) are expected.^[^ ^49^, ^55^, ^75^ ^]^

Considering the various roles silver salts are known to play in C─H activation,^[^ ^76^, ^77^, ^78^, ^79^, ^80^, ^81^ ^]^ we investigated computationally whether silver would lower any of the barriers, but, unlike in some related systems,^[^ ^34^ ^]^ found that the redox neutral (Pd^II^/Pd^II^) pathway itself neither requires silver as oxidant, nor for bromide stripping in the steps following the MI. In the subsequent steps after **Int‐6**, the barriers are all comparably low in energy in the absence of silver. In the formation of **3** Ag is only required to regenerate the catalyst from **Int‐10**.

**Figure 9:**
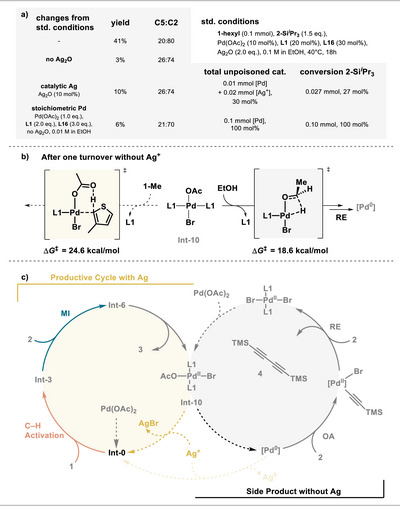
a) Control experiments to study the role of silver. b) C─H activation versus Pd^0^ formation in the absence of silver and presence of bromide. c) Simplified catalytic cycles rationalizing the role of silver in suppressing side product formation.

We conducted control experiments (Figure 9a) which confirm that silver is required for product formation and only traces of product are formed in the absence of silver. Interestingly, even in the presence of stoichiometric amounts of Pd but absence of silver, only little product formation was observed. The reagent **2‐Si*^i^* Pr_3_** was nevertheless consumed to a large degree. We could trace back the high conversion of the bromo alkyne to an alkyne‐alkyne homocoupling forming a Glaser‐like product **4**. The conversion of alkyne was correlated to the amount of Pd‐catalyst available. When adding catalytic amounts of silver (20 mol% Ag^+^) and Pd (10 mol%), 27 mol% of **2‐Si*^i^* Pr_3_** were consumed. Consequently, in the stoichiometric experiment one equivalent of **2‐Si*^i^* Pr_3_** was consumed. These experiments show that silver is needed to enable the desired thiophene alkynylation reactivity, but can also promote catalyst turnover in the alkyne homocoupling side‐reaction.

We investigated the formation of the side‐product computationally (Figure 9b, for more details see Supporting Information) and found that in the presence of bromide ions, i.e., after completing the initial catalytic cycle, Pd(0) forms rapidly and a subsequent oxidative addition of alkyne triggers homocoupling, which proceeds faster than thiophene alkynylation. Ag_2_O acts as a base and halide scavenger to formally trap HBr and cou also reoxidize Pd^0^ that is formed in side reactions. In the absence of silver, Pd(OAc)_2_ is gradually converted to a fully inactive palladium dibromide species. These mechanistic findings are summarized in Figure 9c.

## Conclusion

Several important conclusions can be drawn from this study:

- MLR may be a valuable tool to rationalize and, in some circumstances, even predict ligand effects on the regioselectivity of nondirected C─H activation processes as exemplified in this case study
- Traditional implicit solvation may fail to (even qualitatively) describe the regioselectivity for such complex systems
- LED analysis and visualizing dispersion interactions can help to understand if an attenuation of dispersion interactions could be responsible for inaccurate predictions
- A Curtin‐Hammett scenario with a switch in selectivity determining step **TS‐4** versus **TS‐5** is responsible for the different selectivities observed with ligands **L2** and **L16**
- Ethanol plays a crucial role in facilitating substrate coordination by prior direct coordination to the catalyst center
- The role of silver is to suppress the undesired formation of homocoupled alkyne and recycle the catalyst

MLR is able to bridge the gap between two distinct mechanistic regimes (**TS‐4** and **TS‐5**) and predicts ligand‐dependent selectivities with reasonable accuracy, only using a simple model compound and well‐interpretable descriptors. This is interesting, especially in light of the failure of even high‐quality CCSD(T) with implicit solvation to qualitatively predict the correct regioisomeric outcome. We provide an explanation and visualization method showing why the relative stabilities of **TS‐5** were incorrectly modelled with implicit solvation. We expect this work will prove highly useful as a basis for future computational studies aiming to assess challenging selectivities. Notably, a very recent theoretical study by Bistoni in the field of asymmetric organocatalysis also showcases the need for methods to study computational shortcomings due to explicit solvent–solute effects.^[^ ^82^ ^]^

Alcoholic solvents, including hexafluoroisopropanol (HFIP), are often vital in palladium‐catalyzed C─H activation despite their ambiguous role.^[^ ^83^ ^]^ It is commonly assumed that hydrogen bonding lowers the C─H activation barrier. We herein introduce an additional explanation for the role of alcoholic solvents: the direct coordination to the metal center and simultaneous H─bond to the ligand as a way to increase the rate of substrate coordination.

We furthermore provide evidence for the role of silver in our transformation. Understanding the roles of silver in C─H functionalization is crucial for developing silver‐free variants – one of the major challenges in the field of C─H activation.^[^ ^76^ ^]^ The role of silver to suppress detrimental side reactions has not been described to date and may be quite prevalent considering the widespread use of alkynyl bromides in C─H activation.

Overall, this study offers new directions for a holistic investigation of reaction mechanisms in C─H activation, taking into consideration all reaction partners. Several methodological aspects are expected to prove useful in the future, and the study opens up new perspectives concerning solvent effects and the role of silver salts in the field of C─H activation.

## Supporting Information

The authors have cited the following references within the Supporting Information.^[^ ^2^, ^8^, ^12^, ^20^, ^22^, ^23^, ^27^, ^31^, ^35^, ^36^, ^37^, ^38^, ^39^, ^43^, ^46^, ^47^, ^49^, ^50^, ^51^, ^52^, ^53^, ^54^, ^55^, ^56^, ^57^, ^59^, ^61^, ^62^, ^63^, ^64^, ^65^, ^66^, ^67^, ^68^, ^69^, ^70^, ^76^, ^78^, ^81^, ^84^, ^85^, ^86^, ^87^, ^88^, ^89^, ^90^, ^91^, ^92^, ^93^, ^94^, ^95^, ^96^, ^97^, ^98^, ^99^, ^100^, ^101^, ^102^, ^103^, ^104^, ^105^, ^106^, ^107^, ^108^, ^109^, ^110^, ^111^, ^112^, ^113^, ^114^, ^115^, ^116^, ^117^, ^118^, ^119^, ^120^, ^121^, ^122^, ^123^, ^124^, ^125^, ^126^, ^127^, ^128^, ^129^, ^130^, ^131^, ^132^, ^133^, ^134^, ^135^, ^136^, ^137^, ^138^, ^139^, ^140^, ^141^, ^142^, ^143^, ^144^, ^145^, ^146^, ^147^, ^148^, ^149^, ^150^, ^151^, ^152^, ^153^, ^154^, ^155^, ^156^, ^157^, ^158^, ^159^, ^160^, ^161^, ^162^, ^163^, ^164^, ^165^, ^166^, ^167^, ^168^ ^]^ Coordinates as xyz files, descriptor datasheets, and further supporting material were deposited at the open access repository ZENODO, https://doi.org/10.5281/zenodo.15126469.

## Conflict of Interests

The authors declare no conflict of interest.

## Supporting information



Supporting information

## Data Availability

The data that support the findings of this study are openly available in [Zenodo] at [https://doi.org/10.5281/zenodo.15126469].
