# Supplementary material for: Elucidating Ligand‐Dependent Selectivities in Pd‐Catalyzed C–H Activations
Source: Angew Chem Int Ed Engl. 2025 Oct 23;64(51):e202507500. doi: 10.1002/anie.202507500 (PMC12707355; doi:10.1002/anie.202507500)
Supplement: Supplementary file 1 — Supporting information [file ANIE-64-e202507500-s001.pdf]

Supporting Information

Elucidating Ligand-Dependent Selectivities in Pd-Catalyzed C–H Activations

Fritz Deufel[1], Monika Ravi[1], and Manuel van Gemmeren[1]

[1]Otto-Diels-Institut für Organische Chemie, Christian-Albrechts-Universität zu Kiel, Otto-Hahn-Platz 4, 24118 Kiel, Germany

1 General Computational Details ... 1
2 Multivariate Linear Regression ... 2
3 DFT Exploration of the PES ... 15
3.1 Initial Hypotheses for Selectivity Switch ... 16
3.2 Switch in Turnover Limiting Step ... 25
3.3 Proposed Simplified Catalytic Cycle ... 45
3.4 Role of silver ... 54
4 Experimental Studies ... 61
4.1 General Information ... 61
4.2 Deuterium Kinetic Isotope Effect ... 79
4.3 Variable Time Normalization Analysis ... 88
4.4 $^{12}C/^{13}C$ KIE ... 109
5 References ... 117

1 General Computational Details

All quantum chemical calculations were carried out using the ORCA software[1,2] (version 5.0.4)[3,4] with default settings unless otherwise specified. Structures were visualized either with VMD[5] or PyMol.[6] Structures were built using Avogadro[7] and if needed preoptimized using the extended tight binding method (version 6.6.1).[8] The semiempirical GFN1-xtb[9] was often found to reproduce DFT optimized geometries better for the Pd complexes under study. Conformers were sampled manually and additionally generated using the conformer rotamer ensemble tool (CREST)[10] in case of conformationally more flexible structures. The structures were reoptimized using a DFT method as specified in the respective section in conjunction with an Ahlrich[11] type basis set (def2) unless specified otherwise. All calculations made use of the resolution of identity approximation (RIJCOSX) to economize the evaluation of integrals. For final single point energy calculations (wavefunction and DFT), tighter grid (defgrid3), and SCF (tightscf) keywords were used. Experimental C2 vs C5 (*N*(C2):*N*(C5))selectivities were converted to $\Delta\Delta G^{\ddagger}$ values at the experimental temperature according to eq. (1).

$$\Delta\Delta G^{\ddagger} = R \cdot T \cdot \ln\left(\frac{N(\mathrm{C2})}{N(\mathrm{C5})}\right) \tag{1}$$

All DLPNO-CCSD(T) calculations made use of defgrid3, tightscf, and tightpno settings. For the CBS (complete basis set) scheme def2-TZVPP and def2-QZVPP with the respective auxiliary basis set def2/J for coulomb fitting and def-TZVPP/C or def2-QZVPP/C for correlation calculations were used and the 3/4 extrapolation was done manually as suggested in the literature.[12,13]

Additional raw data for the computational analysis (.xyz files, energy values, descriptors, etc.) are deposited at the ZENODO archive (DOI: 10.5281/zenodo.15126469).

2 Multivariate Linear Regression

Alternate methods for predicting ligand performance due to potential complications regarding quantitative predictions using conventional DFT and TST were assessed. MLR analysis seemed like a viable tool that could be employed for modelling ligand performance and potentially predict out-of-sample ligands with higher accuracy than the respective DFT model. The originally reported bidentate ligand screening Figure S1 provides a suitable dataset for this purpose. MLR has proven to also be suitable for small data-set regimes to which we are limited since the heterogene nature of the rection (silver) as well as limited ligand space (many ligands need to be self-synthesized) render high-throughput experimentation prohibitively laborious. The selectivity data-set at hand is nevertheless sufficiently diverse containing 18 well dispersed datapoints with selectivities ranging from C2:C5 = 93:7 to 20:80 so it is not skewed. The data also covers enough intermediate points such as not to having to be classified as binned making it generally suitable for MLR.[14]

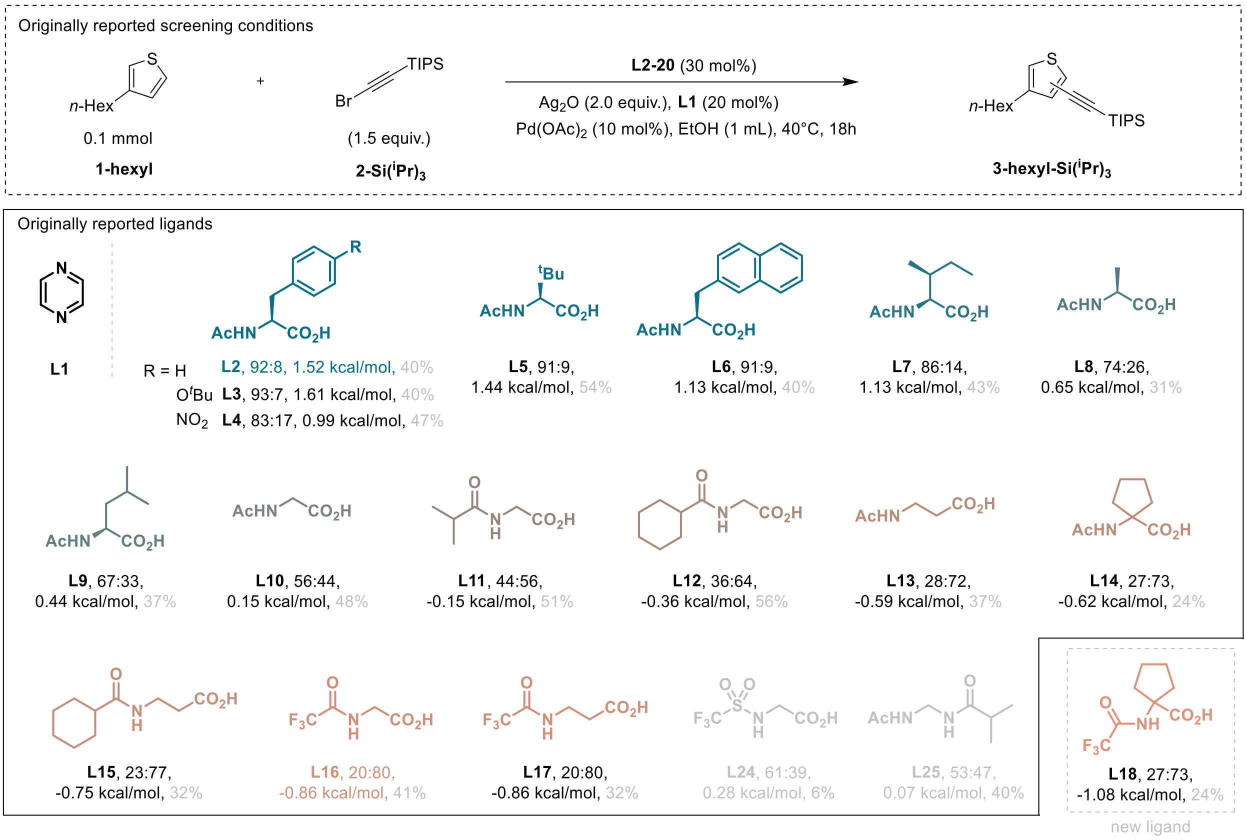

Figure S1: Overview of the originally reported screening results for the bidentate ligand and new ligand L18.

Parameters

We envisioned that a model compound as shown in Figure S2 would be suitable for extracting relevant DFT-based parameters for multivariate modelling.

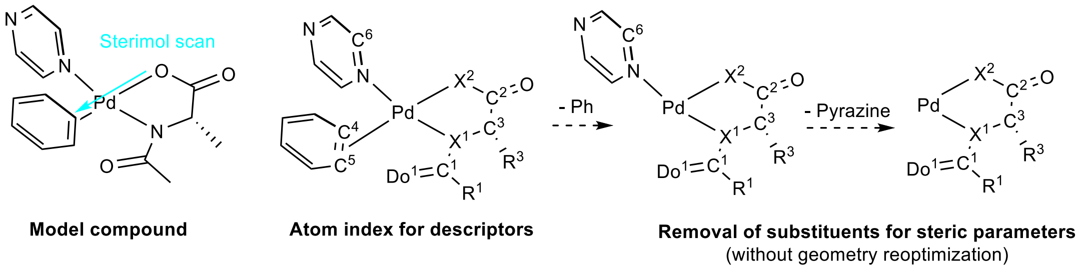

Figure S2: Simplified model compound for extraction of suitable DFT paramters.

The compound was chosen to be as similar to the complex of interest as possible. The model compound resembles a pre-reactive intermediate, but thiophene was replaced with benzene to reduce the number of conformers. Conformational sampling was performed but only the most stable conformer at the geometry optimization level was considered for DFT analysis. The compound was optimized using the PBE0-D3(BJ)/def2-TZVP functional and basis set combination. All electronic descriptors were extracted at the same level of theory.

Parameters outlined in Table S1 were considered for generating a suitable model. The sterimol[15–18] parameters Bmax, Bmin, L as well as %Vbur were extracted with the help of the DBSTEP python script developed by the Paton group.[19] The remaining values were extracted directly from the ORCA output file or in case of dihedral angles, distances and angles calculated from the coordinates. ORCA was interfaced with the NBO software[20,21] for NPA based charges and occupancy and energy of NBO based orbitals.

Table S1: DFT derived parameters considered for MLR.

Parameter	Annotation	Parameter	Annotation	Parameter	Annotation
Chelpg(Pd)	*Chelpg Charge on Pd*	BD*_Occ (X1-Pd)	*Occupancy of the anti-bonding orbital between X1 and Pd*	3_bmin_all	*Bmin at a 3 Å distance for the entire molecule*
NBO (Pd)	*NPA charge on Pd*	BD*_E (N1-C)	*Energy of the anti-bonding orbital between N1 and C*	4_bmin_all	*Bmin at a 4 Å distance for the entire molecule*
LP_E_Pd(1)	*Energy of the first lone pair orbital on Pd*	BD*_Occ (N1-C)	*Occupancy of the anti-bonding orbital between N1 and C*	0_bmax_all	*Bmax at a 0 Å distance for the entire molecule*
LP_Occ_Pd(1)	*Occupancy of the first lone pair orbital on Pd*	BO (Pd-X1)	*Bond order (Mayer) between Pd and X1*	1_bmax_all	*Bmax at a 1 Å distance for the entire molecule*
LP_E_Pd(2)	*Energy of the second lone pair orbital on Pd*	BO (Pd-X2)	*Bond order (Mayer) between Pd and X2*	2_bmax_all	*Bmax at a 2 Å distance for the entire molecule*
LP_Occ_Pd(2)	*Occupancy of the second lone pair orbital on Pd*	BO (Pd-N1)	*Bond order (Mayer) between Pd and N1*	3_bmax_all	*Bmax at a 3 Å distance for the entire molecule*
LP_E_Pd(3)	*Energy of the third lone pair orbital on Pd*	BD (Pd-X1)	*Bond distance between Pd and X1*	4_bmax_all	*Bmax at a 4 Å distance for the entire molecule*

LP_Occ_Pd(3)	*Occupancy of the third lone pair orbital on Pd*	BD (Pd-X2)	*Bond distance between Pd and X2*	3.5vbur_noph	*Buried volume at a 3.5 Å for the Ph truncated molecule*
LP_E_Pd(4)	*Energy of the fourth lone pair orbital on Pd*	BD (Pd-N1)	*Bond distance between Pd and N1*	0_bmin_noph	*Bmin at a 0 Å distance for the Ph truncated molecule*
LP_Occ_Pd(4)	*Occupancy of the fourth lone pair orbital on Pd*	BD (Pd-MidPh)	*Bond distance between Pd and center of C4 and C5*	1_bmin_noph	*Bmin at a 1 Å distance for the Ph truncated molecule*
Chelpg(X1)	*Chelpg Charge on X1*	BD (Do1-MidPh)	*Bond distance Do1 and center of C4 and C5*	2_bmin_noph	*Bmin at a 2 Å distance for the Ph truncated molecule*
NBO (X1)	*NPA charge on X1*	BD (Do1-c4)	*Bond distance Do1 and C4*	3_bmin_noph	*Bmin at a 3 Å distance for the Ph truncated molecule*
LP_E_X1 (1)	*Energy of the first lone pair orbital on X1*	BD (Do1-c5)	*Bond distance Do1 and C5*	4_bmin_noph	*Bmin at a 4 Å distance for the Ph truncated molecule*
LP_Occ_X1 (1)	*Occupancy of the first lone pair orbital on X1*	BD (Do1-c4or5)min	*Minimal bond distance: either Do1-c4 or Do1-c5*	0_bmax_noph	*Bmax at a 0 Å distance for the Ph truncated molecule*
Chelpg(X2)	*Chelpg Charge on X1*	BD (Do1-c4or5)max	*Maximum bond distance: either Do1-c4 or Do1-c5*	1_bmax_noph	*Bmax at a 1 Å distance for the Ph truncated molecule*
NBO (X2)	*NPA charge on X2*	Diff BD(Do1-c4andc5)	*Difference between bond distances Do1-C4 and Do1-C5*	2_bmax_noph	*Bmax at a 2 Å distance for the Ph truncated molecule*
LP_E_X2 (1)	*Energy of the first lone pair orbital on X2*	BA (X1-Pd-X2)	*Bond angle between X1, Pd and X2*	3_bmax_noph	*Bmax at a 3 Å distance for the Ph truncated molecule*
LP_Occ_X2 (1)	*Occupancy of the first lone pair orbital on X2*	BA (N1-Pd-X2)	*Bond angle between N1, Pd and X2*	4_bmax_noph	*Bmax at a 4 Å distance for the Ph truncated molecule*
LP_E_X2 (2)	*Energy of the second lone pair orbital on X2*	BA (X1-Pd-c45)	*Bond angle between X1, Pd and midpoint between C4 and C5*	L_bmax_noph	*L along the scan axis for the Ph truncated molecule*
LP_Occ_X2 (2)	*Occupancy of the second lone pair orbital on X2*	BA (X1-C1-Do1)	*Bond angle between X1, C1 and Do1*	3.5_vbur_noph_nopy	*Buried volume at a 3.5 Å for the Ph and Pyrazine truncated molecule*
Chelpg(Do1)	*Chelpg Charge on Do1*	DH (Pd-X1-C1-Do1)	*Dihedral angle Pd, X1, C1 and Do1*	0_bmax_noph_nopy	*Bmax at a 0 Å distance for the Ph and Pyrazine truncated molecule*
NBO (Do1)	*NPA charge on Do1*	DH (X1-C3-C2-X2)	*Dihedral angle X1, C3, C2 and X2*	1_bmax_noph_nopy	*Bmax at a 1 Å distance for the Ph and Pyrazine truncated molecule*
LP_E_Do1 (1)	*Energy of the first lone pair orbital on Do1*	DH (X2-Pd-X1-C1)	*Dihedral angle X2, Pd, X1 and C1*	2_bmax_noph_nopy	*Bmax at a 2 Å distance for the Ph and Pyrazine truncated molecule*
LP_Occ_Do1 (1)	*Occupancy of the first lone pair orbital on Do1*	DH (R1-C3-X1-C1)	*Dihedral angle R1, C3, X1 and C1*	3_bmax_noph_nopy	*Bmax at a 3 Å distance for the Ph and Pyrazine truncated molecule*
Chelpg(C1)	*Chelpg Charge on C1*	DH (R1-C3-X1-Pd)	*Dihedral angle R1, C3, X1 and Pd*	4_bmax_noph_nopy	*Bmax at a 4 Å distance for the Ph and Pyrazine truncated molecule*
NBO (C1)	*NPA charge on C1*	DH (R1-C3-C2-X2)	*Dihedral angle R1, C3, C2 and X2*	L_noph_nopy	*L along the scan axis for the Ph and Pyrazine truncated molecule*
Chelpg(C2)	*Chelpg Charge on C2*	DH (c6-N1-Pd-X2)	*Dihedral angle C6, N1, Pd and X2*	Vbur3.5_R1	*Buried volume at a 3.5 Å for isolated R1*

NBO (C2)	*NPA charge on C2*	DH (c6-N1-Pd-c45)	*Dihedral angle C6, N1, Pd and midpoint between C4 and C5*	Bmin_R1	*Bmin for isolated R1*
Chelpg(C3)	*Chelpg Charge on C3*	DH (Pd-X1-C3-C2)	*Dihedral angle Pd, X1, C3 and C2*	Bmax_R1	*Bmax for isolated R1*
NBO (C3)	*NPA charge on C3*	DH (Pd-X2-C2-C3)	*Dihedral angle Pd, X2, C3 and C2*	L_R1	*L for isolated R1*
Chelpg(N1)	*Chelpg Charge on N1*	DH (c4-c5-Pd-X1)	*Dihedral angle C4, C5, Pd and X1*	Vbur3.5_R3	*Buried volume at a 3.5 Å for isolated R3*
NBO(N1)	*NPA charge on N1*	Polarizability	*Polarizability of the entire molecule*	Bmin_R3	*Bmin for isolated R1*
LP_E_N1 (1)	*Energy of the first lone pair orbital on N1*	HOMO	*Highest occupied molecular orbital*	Bmax_R3	*Bmax for isolated R3*
LP_Occ_N1 (1)	*Occupancy of the first lone pair orbital on N1*	LUMO	*Lowest unoccupied molecular orbital*	L_R3	*L for isolated R3*
BD_E (X1-Pd)	*Energy of the bonding orbital between X1 and Pd*	Dipole Moment	*Dipolar moment of the overall molecule*	v_C1O	*Vibrational frequency of the C=O stretch of C1 and Do1*
BD_Occ (X1-Pd)	*Occupancy of the bonding orbital between X1 and Pd*	Vbur 3.5_All	*Buried volume at a 3.5 Å for the entire molecule*	I_C1O	*Intensity of the C=O stretch of C1 and Do1*
BD_E (N1-Pd)	*Energy of the bonding orbital between N1 and Pd*	0_bmin_all	*Bmin at a 0 Å distance for the entire molecule*	v_C2O	*Vibrational frequency of the C=O stretch of C2 and O*
BD_Occ (N1-Pd)	*Occupancy of the bonding orbital between N1 and Pd*	1_bmin_all	*Bmin at a 1 Å distance for the entire molecule*	I_C2O	*Intensity of the C=O stretch of C2 and O*
BD*_E (X1-Pd)	*Energy of the anti-bonding orbital between X1 and Pd*	2_bmin_all	*Bmin at a 2 Å distance for the entire molecule*		

Multivariate Modelling

Two separate datasets were considered (Figure S3):

Dataset A: a more focused dataset with only N-acyl α-amino acid ligands

Dataset B: a more general dataset which also contains N-acyl β-amino acid derived ligand structures

L25 potentially reacts in a different way (higher pK_A value compared to a carboxylic acid handle) and **L24** has a low yield so the selectivity could be due to background reaction. Therefore, both ligands were excluded from modelling.

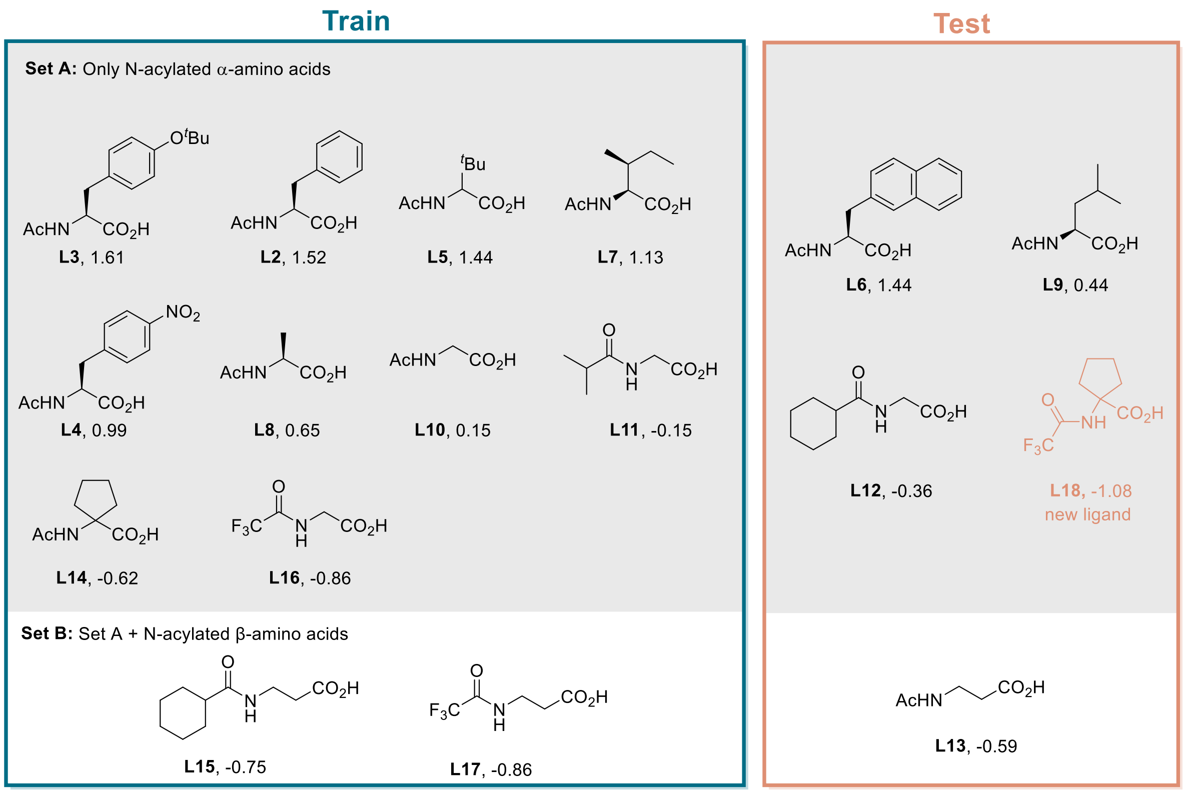

Figure S3: Train/Test split of ligands used in for MLR analysis with dataset A and B.

A recent review by Milo that covers modelling practices for small data-set and provides additional literature references in case more background information is desired.[22] A Matlab[23] script developed by the group of Sigman[24] was used for finding appropriate models. For modeling purposes, the input parameters (matrix form) were normalized according to equation 2. Normalization of parameters is important so the final models can be discussed by importance of the pre-factor in the final linear equation by MLR. The larger a pre-factor, the more important is the respective parameter for the observed selectivity.

$$P_{\mathrm{norm}} = \frac{P - \mu_{\mathrm{P}}}{\sigma_{\mathrm{P}}} \tag{2}$$

With P the non-normalized parameter, σ the standard deviation and μ the mean.

For details on the implementation of precise modelling workflow and details of the Matlab scripts we refer to the respective original work.[24]

As outlined by Sigman et al.,[24] the final models were curated by removing models with strongly intercorrelated variables (R^2>0.4). This was done to reduce spurious effects of highly correlated parameters. Statistical tests were applied to test model performance and robustness. For the full model (no external test/train split), the R^2 value was determined as a measure of how good the model predicts experimental $\Delta\Delta G^{\ddagger}$ values. The leave-one-out validation is also given, where one datapoint is removed and the model is cross-validated. In an iterative fashion one datapoint is removed, the model is trained on the remaining datapoints and the respective test datapoint is predicted. Q^2 is hereby the Pearson R^2 value from the iteration over all test datapoints. Additionally, k-fold testing (4-fold split averaged over 5 runs) was performed to assess model performance. A 4-fold split was signified hereby that the dataset was split randomly into 4 equal portions, 3 were used for training one for testing. The test R^2 value is then averaged over five independent random seeds and the R^2 value is given as k-fold in the respective sessions. This essentially is an automated version of a

train/test split and is what is commonly recommended for small-datasets.[24,25] The Topliss and Costello rule of thumb states that the ratio between experimental input, i.e. ΔΔ*G* values to descriptors used in the final model should be larger or equal to 5:1.[26] To further ensure physically meaningful models, we restricted ourselves to univariate, two- and maximum three-parameter models (in case of Set B). Models with too many parameters especially in case of small data sets are prone to overfitting.[22] Models with a higher Q^2 than R^2 were also excluded since they mostly originate from far-off LOO predictions that line up arbitrarily but have wrong slope and intercept values.[24]

To further ensure the model robustness, a 70/30 train/test split was carried out as show in Figure S3 including **L18** that was not considered in the original screening. For the train/test split additionally the R^2 value of the train set (R^2), the test set (R^2 validation) and of both combined (R^2 whole set) is give. For the final model sorting and ranking (higher ranking = more robust model), we weighted the different statistical outputs using the default values suggested by Sigman in their original script.[24] A higher value signifies a higher importance of the parameter with R^2_{imp}: 0.4, Q^2_{imp}: 0.8, k-fold$_{imp}$: 0.5, validation $R_{val}{}^2{}_{imp}$: 2, whole set $R_{whole}{}^2{}_{imp}$: 0.4, weighed whole set $R_{weightedwhole}{}^2{}_{imp}$: 0.8). We adjusted the importance of number of parameters *N* parameter N_{imp} from 0.6 to 0.9 to give more importance to models with less parameters. The final weighting is calculated according to the formula:

$$\boldsymbol{score}_{\mathbf{i}} = \left(\left(1 - N_{\mathrm{imp}}\right) + 0.1 \cdot \left(N_{\max} - \boldsymbol{N}_{\mathbf{i}}\right)\right)\Big(\mathrm{R}^2{}_{\mathrm{imp}} \cdot \mathbf{R^2}_{\mathbf{i}} + \mathrm{Q}^2{}_{\mathrm{imp}} \cdot \mathbf{Q^2}_{\mathbf{i}} + \mathrm{k}fold_{\mathrm{imp}} \cdot \mathbf{k}\boldsymbol{fold}_{\mathbf{i}} + \mathrm{R}^2_{val,\mathrm{imp}} \cdot \mathbf{R}^2_{\boldsymbol{val},\mathbf{i}} + \mathrm{R}^2_{whole,\mathrm{imp}} \cdot \mathbf{R}^2_{\boldsymbol{whole},\mathbf{i}} + \mathrm{R}^2_{whole-weighted,\mathrm{imp}} \cdot \mathbf{R}^2_{\boldsymbol{whole-weighted},\mathbf{i}}\Big)$$

with **bold**: from individual model parameter vector, $_{imp}$: importance of the respective parameter as indicated above and grey: stats if external test/train set is used.

The individual models i could then be sorted according to their score (see e.g ZENODO archive or Table S4).

Dataset B (full dataset)

Initially we also examined some univariate correlations that were detected for dataset B and are shown in Table S2. Please note that some of these parameters are to some extent correlated (Figure S4).

Table S2: Strong (R^2>0.3) univariate correlations with ΔΔ*G*‡(exp).

Parameter	R^2
NBO (X1)	0.41
LP_Occ_X2 (1)	0.66
LP_Occ_X2 (2)	0.66
BA (X1-Pd-X2)	0.41
BA (N1-Pd-X2)	0.64
DH (X1-C3-C2-X2)	0.54
DH (R1-C3-X1-Pd)	0.58
DH (c4-c5-Pd-X1)	0.49
Polarizability	0.35
HOMO	0.38
Vbur 3.5_All	0.61
Bmin_R1	0.46
Vbur3.5_R3	0.48
Bmax_R3	0.54
L_R3	0.65

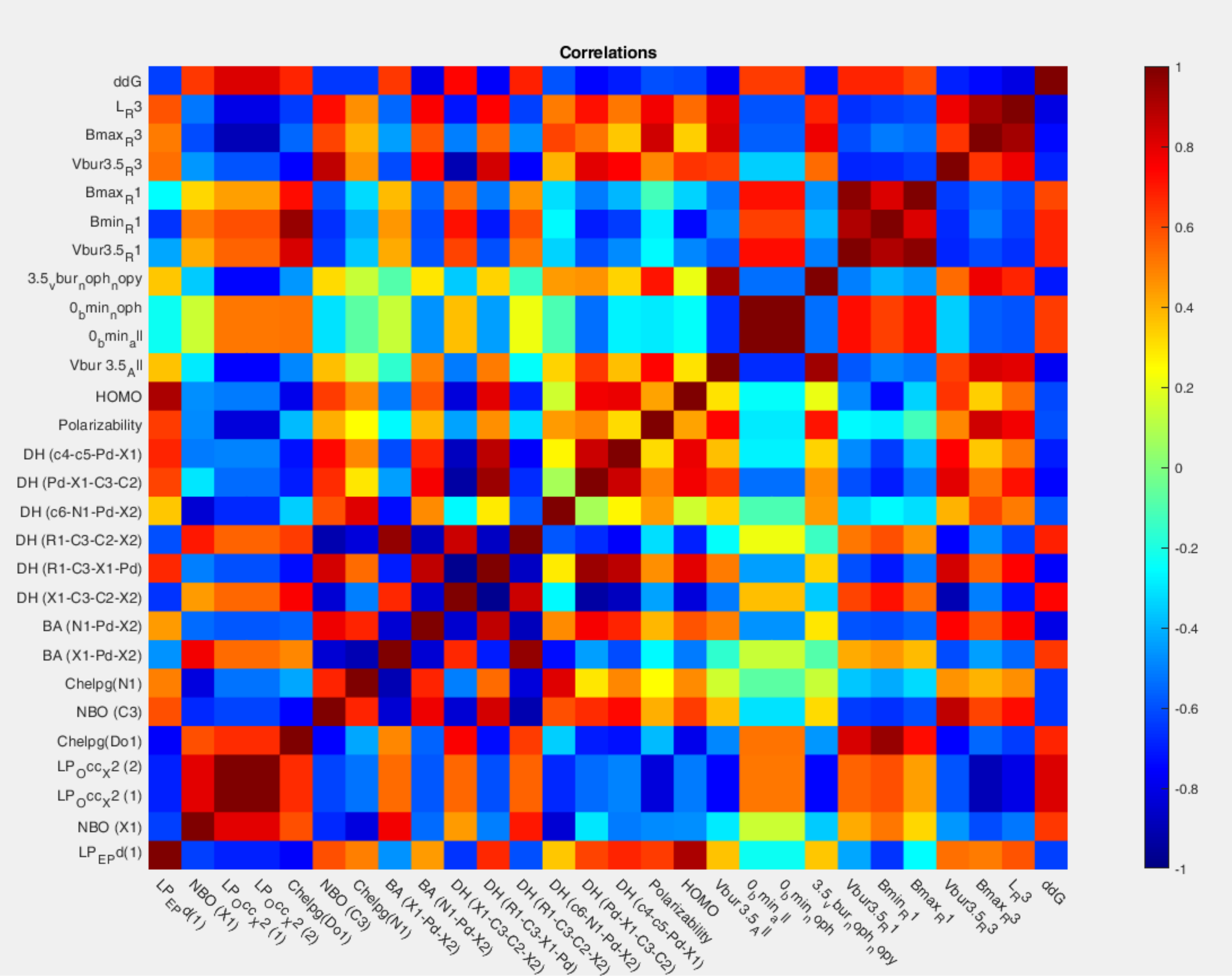

Figure S4: Correlation Matrix for parameters in Table S2.

Since no single parameter gave satisfactory results, we reverted to the aforementioned two (and three) parameter models. We initially focused on dataset B, including N-acyl β-amino acid derived ligands. Many three parameter models performed well but are somewhat less interpretable. We also wanted to identify a model that can be applied on both set A and B, therefore we investigated especially two parameter models in more detail.

Most of these models featured a steric parameter related to the %Vbur. Models with seemingly poorer performance were reinvestigated to see if a more robust two-parameter model could be identified that would be suitable in both datasets A and B hence showing a more interpretable and robust performance. It was found that especially NBO(Pd) and %Vbur_3.5Å_all gave good stats for a two-parameter model in dataset B and on the respective train/test splits (Table S3). We manually checked the same parameters for dataset A alone since it was not shown in the original modelling of dataset A alone. It also shows acceptable statistics for dataset A even though better performing two-parameter models could be found for the isolated set A using different parameter (*vide infra*) and in this sub-dataset the NBO(Pd) and %VBur are to a larger extent correlated (R^2 = 0.63 dataset A vs 0.36 dataset B).

Table S3: Example of a robust two parameter model for predicting ΔΔ$G^‡$ for dataset B. Additional stats for dataset A.

**B test/train**	**R²**	**Q²**	***k*-fold**	**MAE**	**Validation R²**	**Whole set R²**
0.33568 – 0.67242NBO (Pd) + 1.16148Vbur 3.5_All	0.87	0.78	0.75	0.30	0.87	0.86
**B full set**	**R²**	**Q²**	***k*-fold**	**MAE**	**Validation R²**	**Whole set R²**
0.2408 – 0.64407NBO (Pd) + 1.1312Vbur3.5_All	0.88	0.82	0.77	0.29	-	-
**A test/train**	**R²**	**Q²**	***k*-fold**	**MAE**	**Validation R²**	**Whole set R²**
0.58521 – 0.37533NBO(Pd) + 1.1083 Vbur 3.5_All	0.85	0.72	0.75	0.30	0.85	0.85
**A full set**	**R²**	**Q²**	***k*-fold**	**MAE**	**Validation R²**	**Whole set R²**
0.44964 – 0.51664NBO (Pd) + 1.2217Vbur 3.5_All	0.85	0.75	0.79	0.30	-	-

For the full dataset B we also calculated the 3-fold, 4-fold and 5-fold k-fold cross validation averaged over 500 repetitions for which we obtain values of 0.76, 0.76 and 0.77 respectively. A k-fold (4-fold, 500 repetitions) of 0.45 would be obtained using %Vbur_3.5Å_all alone. For NBO(Pd) alone a R^2 of 0.002 and k-fold of 0.41 is obtained.[1] The well-performing LP_Occ_X2 (1) (see Table S2) results in a k-fold of 0.51 would be obtained showing the superiority of a two-parameter model.

The best two parameter model, as shown in Table S3 is plotted in Figure S5.

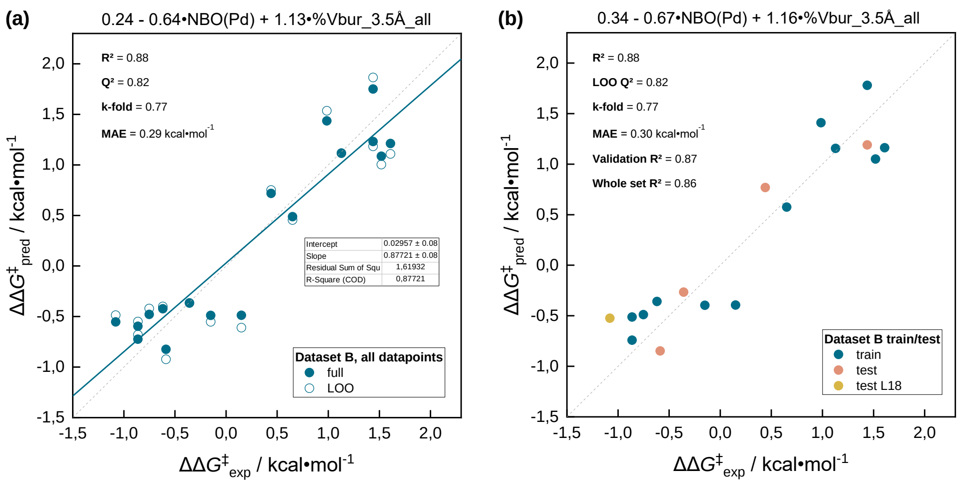

Figure S5: Experimental vs predicted performance of (a) the whole dataset and (b) the train/test split of the dataset B using equations from Table S3.

The normalized parameters offer a certain interpretability. One needs to be cautious with the conclusions since certain parameters can have several effects incorporated. A steric parameter is always of importance as

[1] The higher k-fold value would have been sorted out in our modelling, for reasons indicated above.

well as an electronic parameter. We interpret this in a way that higher steric bulk could improve ligand de-coordination and might be beneficial for a higher C5 selectivity in the MI insertion due to more strain/bulk since higher bulk (higher %Vbur) leads to higher C5 selectivity (negative sign). The coefficient of the normalized dataset of the %Vbur contribution is also larger than for NBO(Pd) illustrating the major role of sterics. The NBO(Pd) indicated that more electrophilic catalysts lead to more C2 selective product. This would be in line with the argument that the C2 position is the electronically more favored position following classical S_EAr rules.

Since different parameters describe e.g. an electronic or steric situation only to some extent, it can be expected that certain descriptors might yield a better model in some cases without necessarily being the most general model. We therefore recommend the subset model in cases where only the subset is to be predicted. For set B other two parameter models did not yield significantly improved performance but several three parameter models gave superior stats. Since here several models performed almost identical with different parameters and no clear "winner" could be identified, we assumed a certain level of overfitting and are hence careful regarding interpretability of the respective models.

Dataset A (N-acyl α-amino acids)

We turned our focus now towards dataset A, since the expected five- and six-membered palladacycle reportedly have substantially different properties and if prediction of another five-membered palladacycle forming ligand should be tested, it might be beneficial to investigate the performance of a truncated dataset as well.[27–29] Focusing on dataset A (Table S4) it becomes apparent that most two-parameter models feature a %Vbur parameter and a more electronic parameter. Please note that the electronic parameters are to some extent intercorrelated and hence exchangeable e.g. B.D.(Pd-X1) (R^2= 0.51) and LP_Occ_Pd(2) (R^2=0.80) show correlation with NBO(X2) for dataset A. For NBO(Pd) the correlation in dataset A with NBO(X2) is R^2 = 0.18 and %VBur_3.5A_all is not intercorrelated (R^2 = 0.0003). LP_Occ_Pd(2) describes the natural orbital derived occupancy of a Pd d-orbital and is strongly correlated with the NBO(X2) parameter. Since this d-orbital points towards X2 (the carboxylate X-type donating site, see Figure S7) a certain correlation is not unexpected.

The buried volume parameters considered in this dataset A are also highly correlated, so it is not surprising that different models featuring one or another %VBur are featured. Note some ligands in other datasets showed diverging %Vbur depending on the ligand and whether Ph or L1 was truncated or not. Here, 3.5_vbur_noph_nopy (R^2 = 0.99) and 3.5vbur_noph (R^2 = 0.99) both correlate strongly with %Vbur_3.5Å_All.

Table S4: Overview of different normalized two-parameter MLR models for predicting $\Delta\Delta G^{\ddagger}$ for subset A.

**A train/test**	**R^2**	**Q^2**	***k*-fold**	**MAE**	**Validation R^2**	**Validation MAE**	**Whole set R^2**
0.58521 – 0.39377LP_Occ_Pd(2) + 0.95724Vbur 3.5_All	0.95	0.92	0.90	0.16	0.89	0.37	0.92
0.58521 – 0.43266LP_Occ_Pd(2) + 0.96731 3.5_vbur_noph_nopy	0.94	0.90	0.90	0.18	0.87	0.39	0.91
0.58521 + 0.39107LP_E_Pd(4) + 0.76602 BD (Pd-X2)	0.97	0.95	0.94	0.13	0.85	0.53	0.86
**0.58521 – 0.34293NBO (X2) + 0.83953Vbur 3.5_All**	**0.93**	**0.85**	**0.84**	**0.19**	**0.90**	**0.27**	**0.91**

0.58521 – 0.43441LP_Occ_Pd(2) + 0.9676 3.5vbur_noph	0.94	0.90	0.87	0.18	0.87	0.39	0.91
0.58521 – 0.6226LP_Occ_X2 (2) – 0.55278 BD (Pd-X1)	0.93	0.84	0.85	0.21	0.89	0.32	0.90
0.58521 – 0.6226LP_Occ_X2 (1) – 0.55278 BD (Pd-X1)	0.93	0.84	0.83	0.21	0.89	0.32	0.90
0.58521 – 0.38583NBO (X2) + 0.8416 3.5vbur_noph	0.93	0.85	0.84	0.20	0.89	0.29	0.90
0.58521 – 0.39341 BD (Pd-X1) + 0.67713Vbur 3.5_All	0.96	0.92	0.89	0.16	0.86	0.39	0.87
**A full set**	**R^2**	**Q^2**	***k*-fold**	**MAE**	**Validation R^2**	**Validation MAE**	**Whole set R^2**
**0.44964 – 0.40861LP_Occ_Pd(2) + 0.90179Vbur 3.5_All**	**0.92**	**0.89**	**0.88**	**0.20**	**-**	**-**	**-**
0.44964 – 0.46027LP_Occ_Pd(2) + 0.90884 3.5_vbur_noph_nopy	0.91	0.87	0.86	0.21	-	-	-
**0.44964 – 0.38693NBO (X2) + 0.81804Vbur 3.5_All**	**0.91**	**0.86**	**0.84**	**0.22**	**-**	**-**	**-**
0.44964 – 0.46458LP_Occ_Pd(2) + 0.90981 3.5vbur_noph	0.91	0.870	0.81	0.21	-	-	-
0.44964 – 0.43778NBO (X2) + 0.81645 3.5_vbur_noph_nopy	0.90	0.85	0.84	0.23	-	-	-
0.44964 – 0.44074NBO (X2) + 0.81602 3.5vbur_noph	0.90	0.85	0.84	0.23	-	-	-

The model featuring NBO(X2) shows the best R^2 and mean average error (MAE) for the validation set and offers a great predictability for the new ligand **L18** (exp -1.07 kcal/mol vs pred -0.99 kcal/mol, Figure S6b). The model featuring LP_Occ_Pd(2) shows slightly better stats for the training set and the full dataset but predicts **L18** slightly worse **L18** (exp -1.07 kcal/mol vs pred -1.45 kcal/mol, Figure S6d) in the test split, which corresponds to a real ligand prediction campaign. When all points are included in the training set the predictions become better for L18 with LP_Occ_Pd(2) (exp -1.07 kcal/mol vs pred -1.02 kcal/mol, Figure S6c).

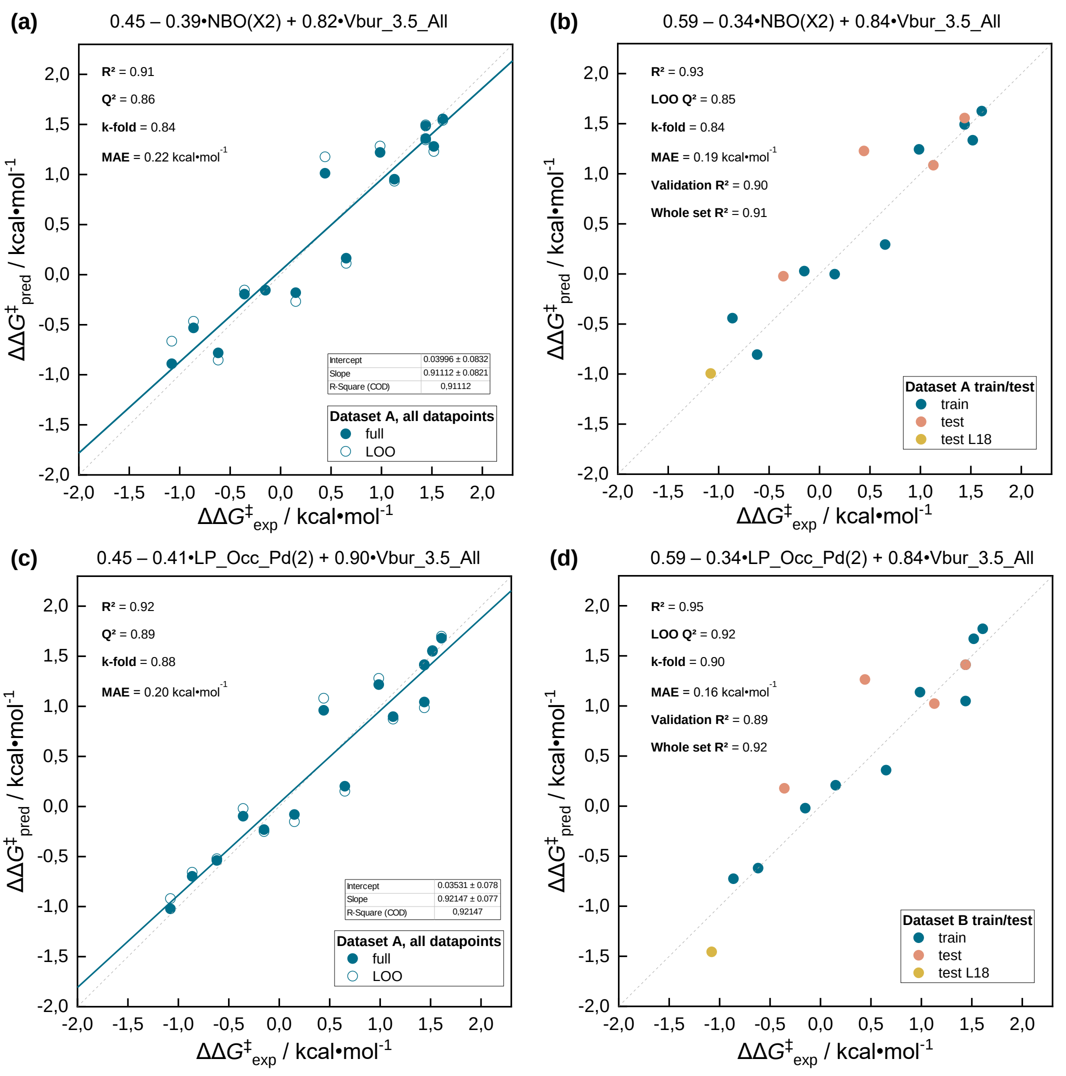

Figure S6: Experimental vs predicted $\Delta\Delta G^{\ddagger}$ / kcal·mol^{-1} for dataset A with a model featuring NBO(X2) and %VBur_3.5 Å_all for (a) all datapoints, (b) a train/test split or a model featuring LP_Occ_Pd(2) and %VBur_3.5 Å_all for (c) all datapoints and (d) a train/test split.

Treating the model involving NBO(X2) and %VBur_3.5 Å_all over the full dataset A with 3-fold, 4-fold and 5-fold k-fold cross validation averaged over 500 repetitions, we obtain values of 0.84, 0.84, 0.83 respectively for LP_Occ_Pd(2) and %Vbur_All 0.86, 0.87, 0.88. The model in Table S3 with NBO(Pd) and a 4-fold cross validation over 500 repetitions yields a k-fold value of 0.74 and the %Vbur parameter alone results in a R^2 of 0.74 and a 4-fold cross validation over 500 repetitions of 0.66.

Looking at the univariate parameters more in detail it becomes apparent that %Vbur performs well at predicting especially more C5 selective ligands (to around 50:50 C2:C5) but does increasingly poorly upon higher C2 selectivities. Looking only at the mostly C2 selective ligands new correlations emerge: BD(Pd-X1) (R^2=0.76), LP_Occ_Pd(2) (R^2=0.79), NBO(X1) (R^2=0.77), NBO(X2) (R^2=0.68), NBO(Pd) (R^2=0.60). It seems like the more occupied the Pd d-orbital or the more electropositive X1 or X2, the more C2 selective is the reaction (Figure S3). We assume that these descriptors perform better in describing the electronic details of the more specialized subset A, but struggle upon including more diverse structures.

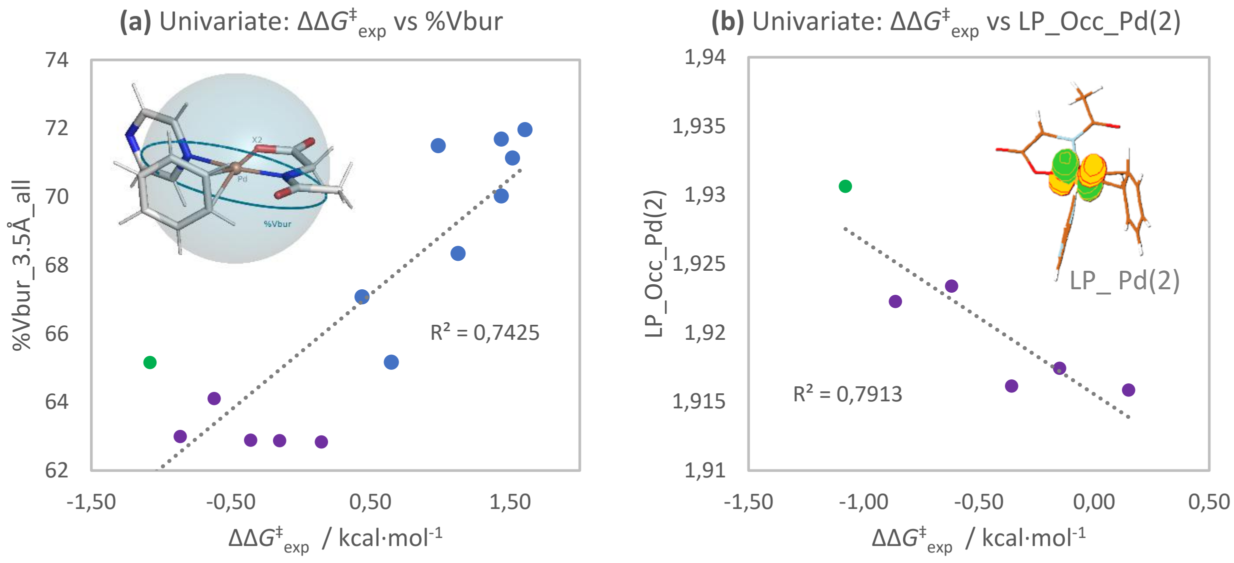

Figure S7: Univariate correlation of $\Delta\Delta G^{\ddagger}$(exp) for the complete dataset A with (a) %Vbur and (b) LP_Occ_Pd(2) for the subset of (a) with more C2 selective ligands indicated in purple and with **L18** in green.

Interestingly, using models derived for dataset A and applying them on unseen data of dataset B gave rather poor performance even when allowing for new weighting of the respective parameters (model with NBO(X2) and %VBur_3.5A_all: R^2 = 0.67, Q^2 = 0.55, k-fold = 0.57, model with LP_Occ_Pd(2) and %Vbur_3.5A_all: R^2 = 0.60, Q^2 = 0.34, k-fold = 0.26). Better performing models for set B either require the exclusion of ligands featuring the cyclopentyl backbone or require the use of a 3-parameter model. The stats are indeed superior to the NBO(Pd), %Vbur parameter model but come with the caveat of potential overfitting and reduced ease of interpretability.

To further test the generalizability of the models developed herein, we tested the models derived from the train set B and A (see Figure S5 and Figure S6b respectively) on additional ligands that were not contained in the original study. These ligands were not used in the prior modelling altogether and therefore provide valuable out-of-sample datapoints.

New ligands (not in the original ACIE publication)

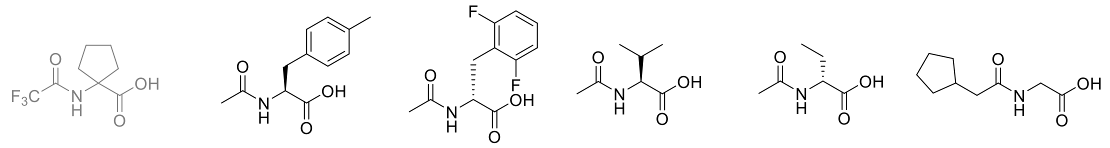

**LX**, C5:C2, exp. $\Delta\Delta G^{\ddagger}$ pred. $\Delta\Delta G^{\ddagger}$ (model A train/test)	**L18**, 15:85,	**L19**, 92:8,	**L20**, 90:10,	**L21**, 85:15,	**L22**, 80:20,	**L23**, 48:52,
	-1.08 kcal/mol	1.53 kcal/mol	1.35 kcal/mol	1.08 kcal/mol	0.87 kcal/mol	-0.05 kcal/mol
	-0.99 kcal/mol	*1.25 kcal/mol*	*1.55 kcal/mol*	*0.81 kcal/mol*	*0.91 kcal/mol*	*-0.003 kcal/mol*

Figure S8: Additional experimental entries to test the model's generalizability and predicted selectivities from the model A test/train.

Both dataset B (featuring the train/test model with NBO(Pd) and %VBur) as well as dataset A (featuring the train/test model with NBO(X2) and %VBur) show reasonable stats for the unseen ligands. The more focused dataset seems to yield slightly better stats, as already seen before.

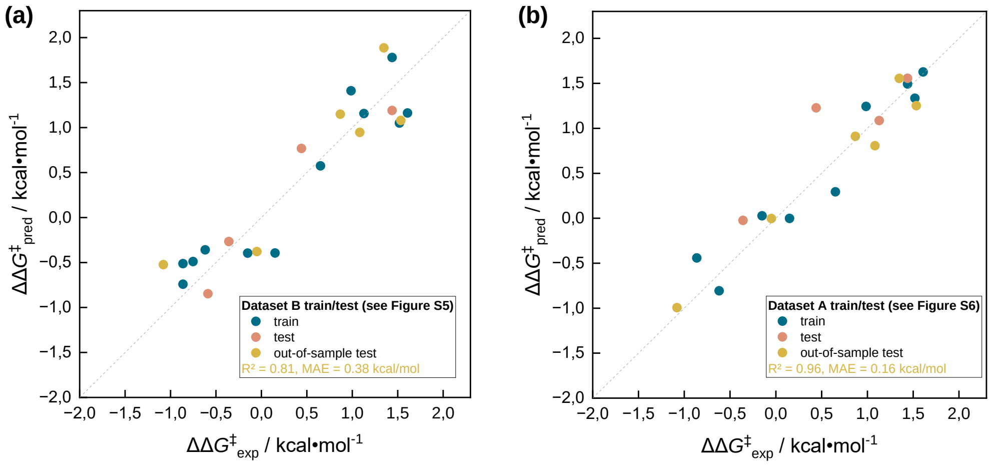

Figure S9: Additional ligands (yellow) as a test set for (a) dataset B train/test and (b) dataset A train/test to test model generalizability.

To conclude, we have focused on finding a more C2 selective ligand, as this was the major shortcoming of the method so far. Predicting more C5 selective ligands was not probed but could potentially be more difficult since exchange with ancillary ligands (e.g. acetate) can be expected making it generally difficult to tune the already intrinsically high selectivities in this step.

3 DFT Exploration of the PES

Initial guesses for transition states (TS) were localized using a constrained bond distance, angle or dihedral scan, or employing the nudged elastic band (NEB) method as implemented in ORCA in combination with GFN1-xtb or a faster DFT method (TPSS-D3(BJ)/def2-SVP). Frequency calculations at the same level of theory as for the geometry optimization for TS, intermediates, and reactants were used to confirm the absence of imaginary frequencies (ground state) or the existence of only one imaginary frequency (transition state). In a few indicated rare instances that were not relevant for relative stabilities ($\Delta\Delta G^{\ddagger}$) (mainly CF_3 group rotation or pyrazine ligand orientation in symmetric and/or sterically undemanding substrates) ground state structures were found to still possess one small imaginary frequency (>20cm^{-1}) that was found impossible to remove even with adjusted grid settings, convergence criteria/thresholds and differing starting geometries or a newer software version (ORCA 6.0).[30] In these cases, geometric permutations and subsequent energy evaluations around the respective vibrational mode were done in a constrained run to verify that the structure indeed is the lowest point on the PES and even values closely around the optimized structure diverged less than <1kcal/mol in free energy). They likely arise from too small grid or RIJCOSX artifacts and can be ignored if properly accounted for.[31] All imaginary modes are indicated in the supporting xyz-files. The nature of the vibrational frequency and intrinsic reaction coordinate (IRC) calculations were used for all transition states followed by geometry optimizations of the respective starting and end geometries to confirm that the proposed transition state does indeed connect the suggested intermediates. Thermochemical correction to obtain free energies at the reaction screening temperature of 313K were evaluated at the same level of theory using the quasi-RRHO approach[32] for small vibrational frequencies as implemented in ORCA. A correction of 2.02 kcal/mol (eq. 3) was added to all structures account for the standard state in solution (1 M) at 313K as is recommended.[31,33] For explicit ethanol solvent molecules a correction of 3.79 kcal/mol was added instead to the free energy at 313K due to the higher bulk solvent concentration of 17.1 M.

$$R \cdot T \cdot \ln(V_{\mathrm{M}}) = 8.314 \frac{\mathrm{J}}{\mathrm{K}} \cdot 313\ \mathrm{K} \cdot \ln\left(\frac{8.314 \frac{\mathrm{J}}{\mathrm{K}} \cdot 313\ \mathrm{K}}{1.013 \cdot 10^{5}\ \mathrm{Pa}} \cdot 10^{3}\right) \cdot 2.392 \cdot 10^{-4} \frac{\mathrm{kcal} \cdot \mathrm{mol}^{-1}}{\mathrm{J}} = 2.02\ \mathrm{kcal} \cdot \mathrm{mol}^{-1} \tag{3}$$

$$\text{with } V_{\mathrm{M}} = \frac{R \cdot T}{P} \tag{4}$$

Implicit solvation effects of ethanol were taken into account by continuum solvation models namely the conductor-like continuum polarization model (CPCM)[34], solvation model based on density (SMD)[35] as implemented in ORCA or the direct conductor-like screening model for real solvents (dCOSMORS),[36] a self-consistent version of COSMO-RS[37–39] as implemented in TURBOMOLE[40–42] (*vide infra*). Solvation model comparisons with dCOSMORS were done by subtracting the unsolvated from the solvated energies and adding this correction to energies obtained from ORCA. Final energies with dCOSMORS shown e.g. in reactions paths in combination with ωB97X-d (as implemented in Turbomole) were directly used from Turbomole (solvation correction + DFT functional), other energies from ORCA. Geometries and free energy corrections were all obtained from ORCA. In Turbomole tighter grid (m4), ri and marij approximations were used.

3.1 Initial Hypotheses for Selectivity Switch

The aim of the initial DFT mechanistic investigation was to decipher the origin of the observed ligand dependent selectivity switch. Different mechanistic scenarios were identified and tested to see if a certain pathway can directly be ruled out.

The ωB97X-D3BJ functional in combination with the def2-TZVP basis set was chosen for geometry optimization, frequency calculation and thermochemical corrections. The functional performs reasonably well in our subsequent benchmark study and related studies.[43] Other, well performing functionals like the PBE0-D3(BJ) that we employed in a previous study often did not converge to a TS with some bidentate ligand combinations in the C–H activation step. The PES there was seemingly too flat preventing the convergence to a stable minimum. It was therefore not considered in the initial exploration. We expect only minor differences for the absolute energy differences, since sufficiently good functionals and large enough basis sets, also for the frequency calculation is employed. We assess differences in functional for the relative energies between two TS isomers *vide infra*.

The PWPB95 double hybrid functional with dispersion correction (D3BJ) in combination with the RI approximation for the MP2 part and the def2-QZVPP basis set and the range-separated hybrid ωB97X-D3BJ/def2-TZVP method were used for initial energy calculations. Please note that the keywords "wB97X-D3BJ" were used for the functional with intrinsically implemented D3BJ correction by Najibi and Goerik rather than "D3(BJ) wB97X" where the D3(BJ) correction is added by ORCA or Turbomole as a correction after the calculation. Both were combined with the CPCM and SMD solvation model yielding a total combination of four different energy values. This approach for initial exploration was chosen to avoid misleading conclusion based on the poor performance of one functional or solvent model with regard to a specific transition state. Both functionals perform well in general as well as transition metal benchmarks.[44–47]

For simplification **1-Me** and **2-SiMe$_3$** were chosen as reaction partners. **2-SiMe$_3$** was experimentally found to decompose fast due to the labile nature of the TMS group, but electronically similar alkynes using a TBS protection or sterically similar ones using a ^{t}Bu group also show the relevant selectivity switch. **1-Me** experimentally also showed numerically similar results compared to **1-hexyl** (Figure S76). It can therefore be argued that both simplifications are justified and reduce the number of conformers to be studied significantly. The focus was mainly on C2 and C5 functionalization of 3-substituted thiophenes, since experimentally no C4 functionalization was observed even in cases of 3-unsubstituted thiophenes.

Hypothesis 1 & 2: Selectivity Switch in C–H Activation

The initial hypothesis voiced in our original report states that the C5 selective catalyst system is intrinsically sensitive to steric effects. This would be in line with our previous mechanistic studies on arenes[48] where the overall preference for the sterically less hindered position is attributed to very synchronous C–H activation that mechanistically lies between the BIES/eCMD and AMLA/CMD (hypothesis 2). By making the catalyst more electrophilic, we assumed to shift the positioning more towards the BIES/eCMD regime, rendering it more sensitive to electronic effects and enabling a C–H activation in the more nucleophilic position. Another possibility would be that the C5 position, i.e. the most acidic position (see manuscript), is preferably functionalized when the C–H activation proceeds via a AMLA/CMD mechanism (hypothesis 1).

To test both hypotheses, the transition states for some relevant N-acyl amino acid derived ligands (Figure S10) for the C2 and C5 C–H activation were computed and their positioning on the More O'Ferrall-Jencks diagram

was evaluated (Figure S13). **Int-0** was assumed to be the resting state since its formation is predicted to be endergonic starting from the palladium acetate trimer. The bidentate X,X-type coordination has previously been suggested and was often found to be energetically favorable compared to a monodentate coordination. As additional L-type ligand thiophene, alkyne, and pyrazine **L1** were probed with the latter one leading to energetically lower TSs (Figure S16d). Also, a monodentate coordination of the carboxylate to act as a CMD group was probed but was found to be higher in energy.

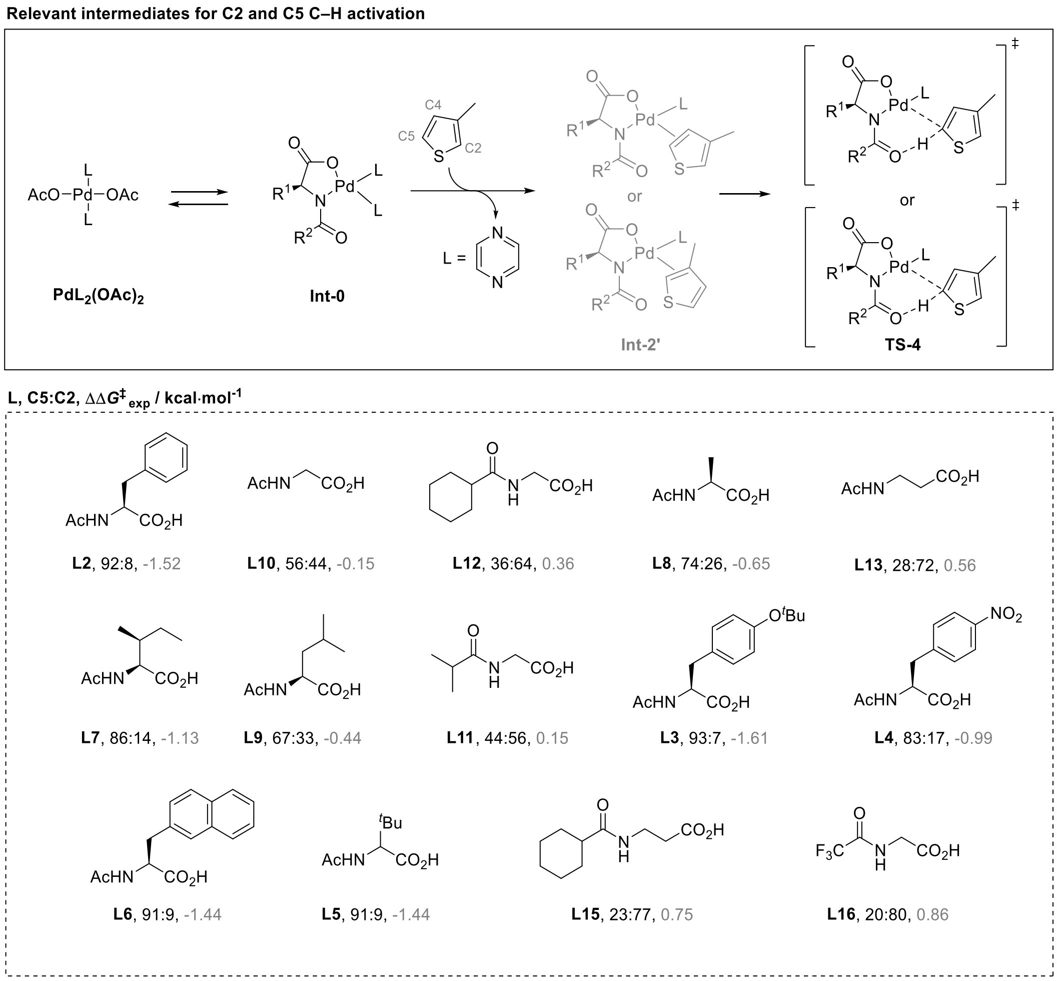

Figure S10: Ligands and experimental selectivity used for TS comparison.

It can be observed that all thiophene ligand combinations are rather clustered and all appear on the eCMD/BIES part of the diagram. This is also in line with other reports on more electrophilic Pd systems employing thiophenes.[49]

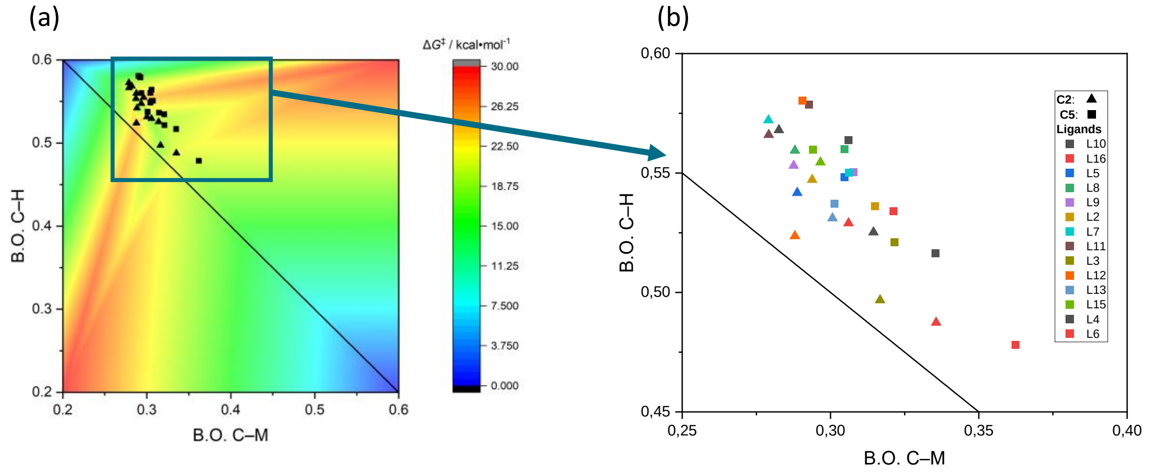

Figure S11: More O'Ferrall-Jencks plot for selected ligands for thiophene C2 and C5 activation using Wiberg bond orders. (a) Large overview with an estimate of the PES and (b) zoom with individual ligands.

As expected from the positioning in the diagram, all transition states show $\Delta\Delta G^{\ddagger}$ values favoring the more nucleophilic C2 position (Figure S12c). The difference in $\Delta\Delta G^{\ddagger}$ was furthermore found to be rather large compared to the experimentally observed $\Delta\Delta G^{\ddagger}$ of 1.18 kcal/mol at 313K for **L16**.

Interestingly a striking correlation of the experimental $\Delta\Delta G^{\ddagger}$ with the calculated barrier of activation ($\Delta G^{\ddagger}$) is observed shown in Figure S12. A weak correlation is observed for all the ligands considering only **Int-0** as the lowest intermediate (Figure S12a). Especially the bulkier ligands seem to not cohere as well to the overall trend. If now the possibility of $PdL1_2(OAc)_2$ as resting state is factored in, a remarkable good correlation (R^2 = 0.93) between the experimental differences in C2 and C5 selectivity ($\Delta\Delta G^{\ddagger}$) and calculated free energy of the C–H activation for the C2 thiophene is observed (Figure S12b). This is irrespective of the solvation model and an equally good correlation is obtained using CPCM (R^2 = 0.91).

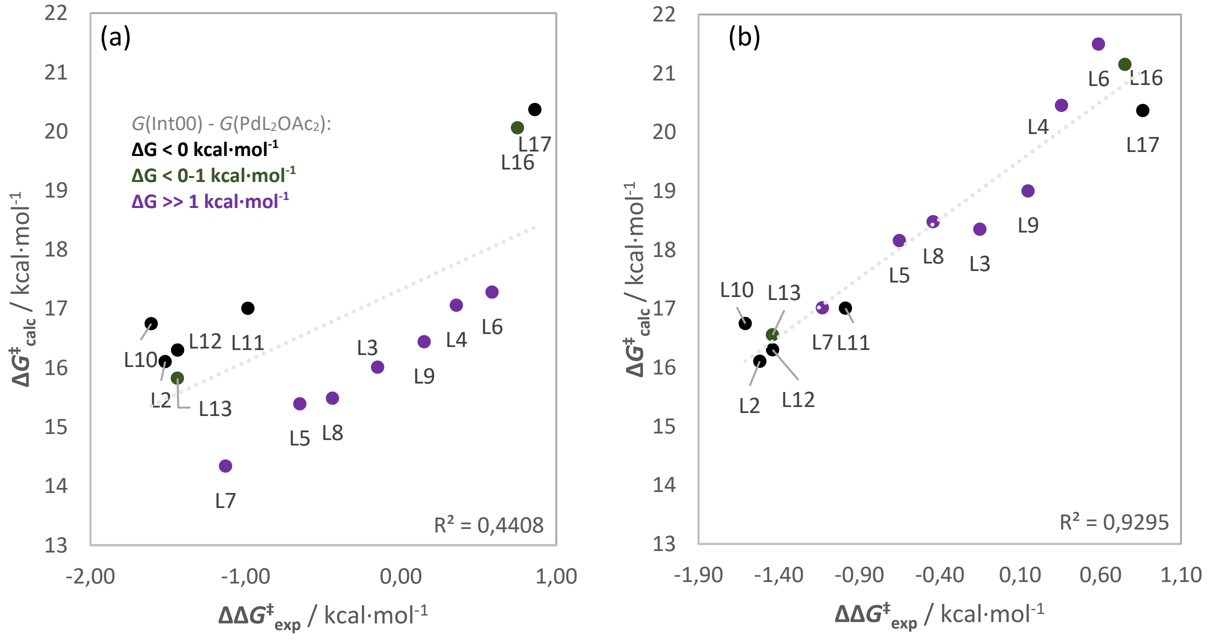

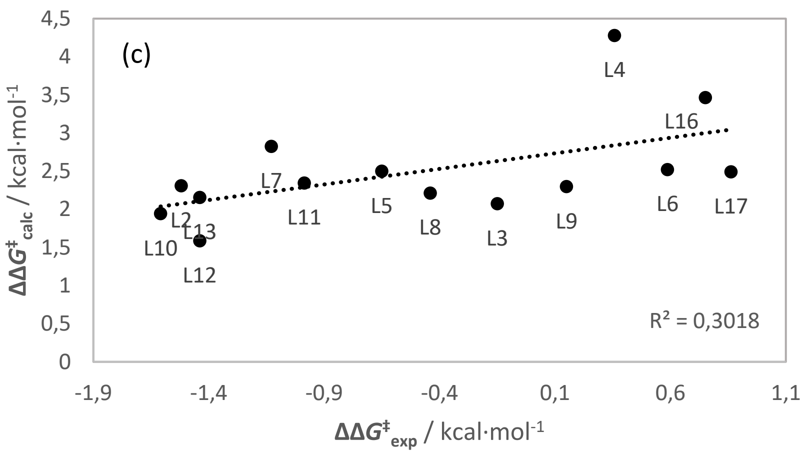

Figure S12: Correlation of the calculated barrier for C–H activation with the experimentally observed selectivity $\Delta\Delta G^{\ddagger}$ with (a) considering **Int-0** as reference point and (b) considering the more stable intermediate so either **Int-0** or $PdL1_2(OAc)_2$ as reference point. (c) Experimental selectivity vs the calculated $\Delta\Delta G^{\ddagger}$ of C2/C5 selectivity for the C–H activation. All calculated at the SMD(EtOH) PWPB96-D3(BJ)/def2-QZVPP//ωB97X-D3BJ/def2-TZVP level of theory.

This hints towards a relevance of the C–H activation for the selectivity. Due to a consistent preference for C2 for the proposed C–H activation step ($\Delta G^{\ddagger}_{calc}$(C2)<< $\Delta G^{\ddagger}_{calc}$(C5)) we nevertheless preliminarily concluded that the C–H activation alone cannot be the driver for the observed ligand dependent selectivity switch. Since the intermediate, prior to the C–H activation was found to be more stable for the C5 thiophene transition, a scan of the C–H bond distance was conducted for **L5**. This was done to ensure that no erroneous convergence of transition state was visible, in case the crossing point of the C2 vs C5 relative PES was very close to the actual transition state. The crossing point nevertheless (see Figure S13a) is still at rather long bond distances. The positioning of the transition state also remains on the eCMD/BIES side of the More O'Ferrall-Jencks diagrams for bond distance values around the maximum (see Figure S13a). This verifies again that the C2 C–H activation step is indeed lower in energy even for a ligand with experimentally observed C5 selectivity.

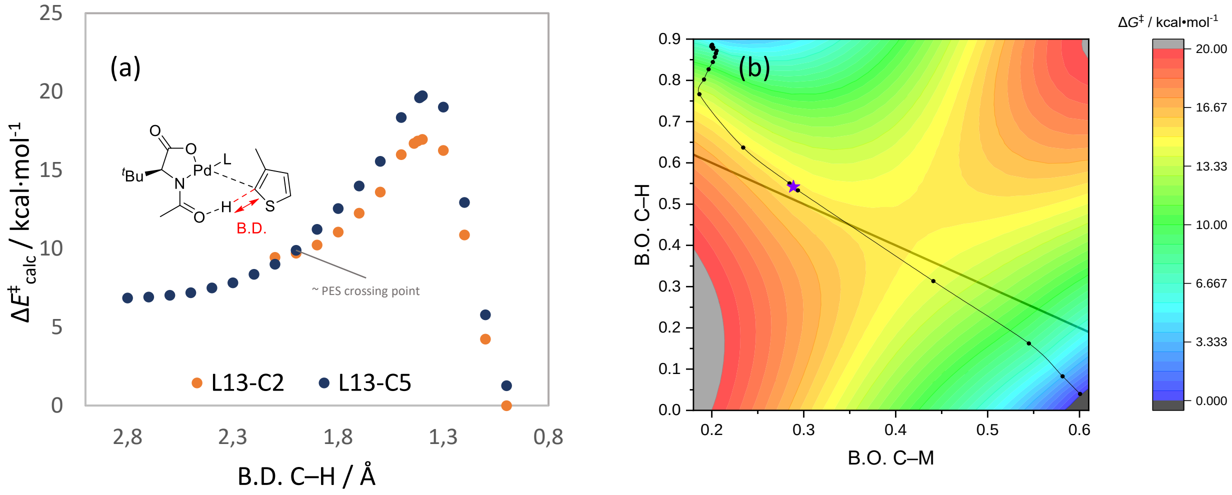

Figure S13: (a) Scan of the electronic energy as a function of the C–H bond distance for **L5**. (b) Potential energy surface mapped on a More O'Ferral-Jencks diagram for the C5 C–H activation. Both at the CPCM ωB97X-D3BJ/def2-TZVP//ωB97X-D3BJ/def2-TZVP level of theory.

Hypothesis 3: Selectivity Switch in C–H Activation and Migratory Insertion

We subsequently hypothesized that a similar scenario, as proposed by Carrow,[50] could explain the selectivity: namely a switch in turnover limiting step could be the origin of selectivity. Previous studies on arenes indicated that our dual-ligand system can at least in part reversibly activate arene C–H bonds. This fact was the foundation of our non-directed arene and heteroarene deuteration studies. Previous works by Maiti and Paton[51] as well as the detailed studies by Musaev and Sarpong[52,53] were used as additional starting points for the alkyne involvement. This will be further discussed in more detail in the following section.

Further discarded hypotheses

Hypothesis 2 appears to be the most likely but further mechanistic scenarios are briefly discussed to rule out their involvement. To facilitate comparison of different pathways, the $PdL1_2(OAc)_2$ is used as the reference point.

Pd(II)/Pd(IV) oxidative addition

Instead of a migratory insertion of the alkyne the possibility of an oxidative addition to Pd(IV) and subsequent reductive elimination was also investigated. This pathway was found to be favorable for the alkyne iodide but unfavorable for the respective chloride and iodide in directed regiodivergent C–H alkynylation described by Musaev and Sarpong[53]. Since the literature system is more strained due to the directing template, we nevertheless calculated the barriers for oxidative addition from several already C–H activated Pd(II) complexes. Different coordination permutations including and not including silver with the respective conformers were investigated. An overview of the most stable structure with the respective category is given in Figure S14. “Monodentate” coordination of N-acyl amino acid ligands with only the carboxylate group was modelled with acetate to reduce the number of conformers for this exploration. Only the C2 selective steps were modelled, since the energy difference between C2 and C5 is expected to be small compared to the overall energy difference between the respective step.

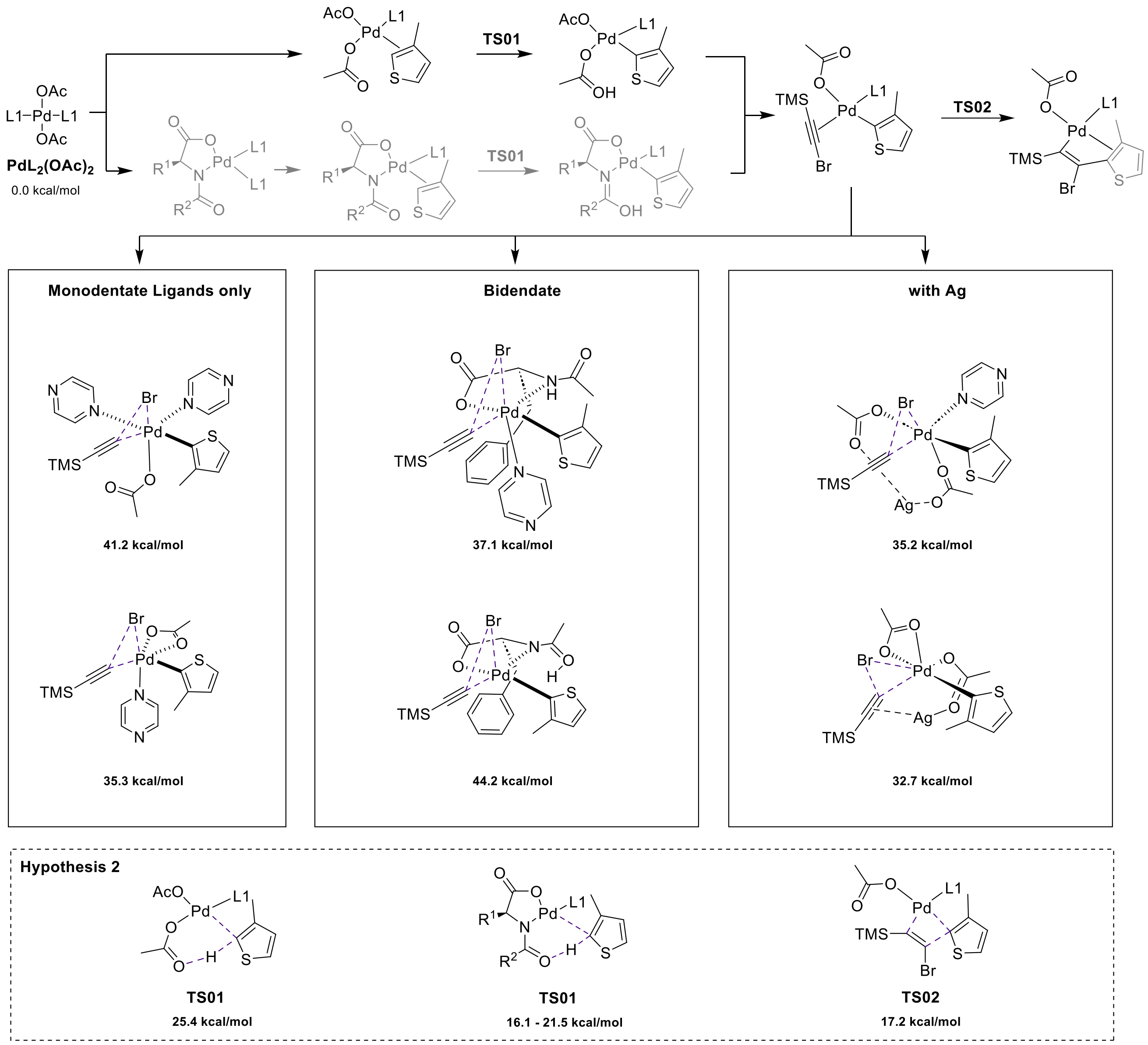

Figure S14: Possible TS for the Pd(II) -> Pd(IV) oxidative addition of bromoalkyne for C2 functionalization. All calculated at the SMD(EtOH) PWPB96-D3(BJ)/def2-QZVPP//ωB97X-D3BJ/def2-TZVP level of theory.

The lowest barrier is in the order of 32.7 kcal/mol and therefore prohibitively large compared to the migratory insertion (17.2 kcal/mol), the C–H activation with various ligands (16.1 - 21.5 kcal/mol) or even without bidentate ligand (25.3 kcal/mol).

This supports the assumption that a Pd(II) to Pd(IV) oxidative addition with the bromoalkyne is less favorable than a possible migratory insertion.

Pd(0)/Pd(II) Oxidative Addition and Follow-up Pathways

In order for this pathway to be feasible, a Pd(0) species would need to be generated. In section 3.4 a similar pathway leading to the formation of the homo-coupled Glaser side-product in the absence of silver is discussed. The presence of silver could potentially reoxidize Pd(0) to Pd(II). Since this thermodynamically favored process could potentially proceed slowly, we briefly discussed potential follow-up steps in Figure S15.

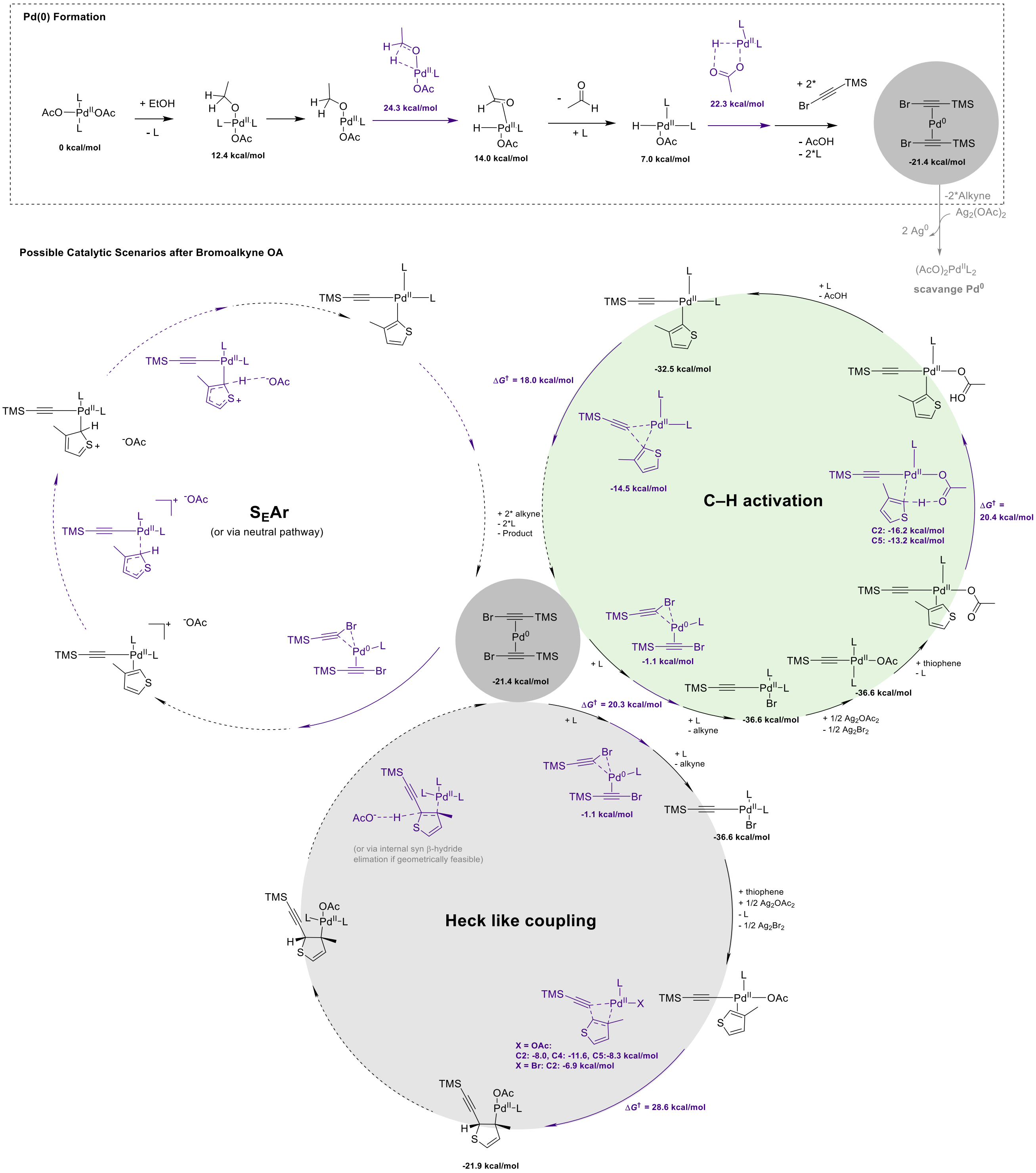

Figure S15: Generation of Pd(0) and possible reaction pathways for product formation after Pd(0) -> Pd(II) oxidative addition. All calculated at the SMD(EtOH) PWPB96-D3(BJ)/def2-QZVPP//ωB97X-D3BJ/def2-TZVP level of theory.

In the literature several Pd catalyzed C–H functionalization reactions, namely arylations are discussed to proceed initially via a Pd(0) -> Pd(II) oxidative addition of an aryl halide. Larrosa et al.[54] discuss the possibility of a subsequent Heck-like insertion of the thiophene to the aryl Pd complex, a CMD assisted C–H activation of the thiophene as well as a S_EAr attack of the electrophilic Pd complex to the thiophene. In their system KIE studies point towards the Heck-like pathway. It has to be noted that no additional ligand is added, a Pd(0) source is given and experimentally C4 arylation is observed for **1-Me**. Grimme, Studer, and Itami[55] explain

diverging regioselectivities depending on the chosen counterion in their stoichiometric thiophene arylation by a fast Heck-like MI that is presumed to be C3 selective[2] (over C2) but reversible. In case of the weakly basic triflate the deprotonation is proposed to be similar in rate for C2 and C3 isomer. Therefore, the selectivity would be imposed by the previous MI leading to the C3 product. According to the authors, different deprotonation rates due to a more basic acetate could then preferentially lead to the C2 product which was not computationally investigated. The authors did also not investigate the possibility of a CMD-type activation of thiophene by the arylated Pd complex bearing an acetate or carbonate ligand which are known to be CMD-enabling groups as shown in Figure S15. This C–H activation would also lead to C2 product. With weakly coordinating triflate on the other hand, a C–H activation would not be possible which could in turn enable the C3-selective Heck-coupling proposed by the authors, thereby providing an alternative explanation for the observed selectivity switch.

Since the initial formation of Pd(II) is unfavorable in our case compared to the N-acyl amino acid catalyzed pathway (24.3 kcal/mol vs ~20 kcal/mol depending on the ligand) this pathway seems less likely. Unlike in section 3.4, where Pd(0) needs to be regenerated for each round of product formation, here catalytic amounts of Pd(0) would suffice since it can get regenerated. But even considering the formation of Pd(0) to be feasible, i.e. small quantities can be formed despite the higher barrier and no reoxidation with Ag, the follow up steps display barriers for e.g. the C–H activation pathway that are higher compared to most typical steps in the Pd(II) N-acyl amino acid catalyzed redox neutral cycle. The largest barrier in the putative Heck like pathway is 28.6 kcal/mol and in the C–H activation pathway with alkyne coordination 20.4 kcal/mol. The energetically more feasible C–H activation is also predicted to yield C2 product and is still higher in energy compared to the typical N-acyl amino acid enabled C–H activation steps (Figure S12). The S_EAr pathway was not considered further since it would essentially relate to an extreme of a C–H activation pathway using external base which is unlikely in the presence of acetate (not present in the Larrosa study) that is known to readily promote CMD. Additionally an S_EAr reaction is expected to also favorably yield C2 product in our case, as evidenced by the higher Fukui index f^- for C2 vs C5 position (C2: 0.17 vs C5: 0.14).

In the absence of an N-acyl amino acid ligand using only pyrazine and acetate the Pd(0)/Pd(II) cycle could in principle be operative. Here the barrier of 25.4 kcal/mol for the silver free C–H activation is competitive with steps in the Pd(0)/Pd(II) cycle. This is important to note since it limits the ligands employable for C2 selective C–H activation: in Figure S12 we argue that a higher barrier of C–H activation renders the C–H activation step more selectivity determining and since this step is predicted to be mostly C2 selective, one would expect even higher selectivities for a more "unfavorable" C–H activation. At some point it is nevertheless likely to be a break in mechanistic regime favoring a different or multiple different paths, which might explain the lower selectivity for a system without N-acyl amino acid ligand despite the predicted C2 vs C5 selectivity in this step (Figure S56: C2:C5 = 61:39 or 46:53 depending in different final conditions).

Ag Mediated C–H Activation

Sanford reports the silver mediated C–H activation of thiophenes.[56] Interestingly it is one of the fewer reports, where the thiophene C–H activation is clearly on the AMLA/CMD part of the mechanistic continuum.[49] So

[2] Please note the difference in nomenclature: In the literature 1-substituted thiophenes are studied. C3-functionalization would be rather C4 in this manuscript assuming a similar mechanism.

similar transition states involving one-center and two-center bimetallic centers were probed under the present conditions. Additionally mixed Pd, Ag transition states were considered (Figure S16).

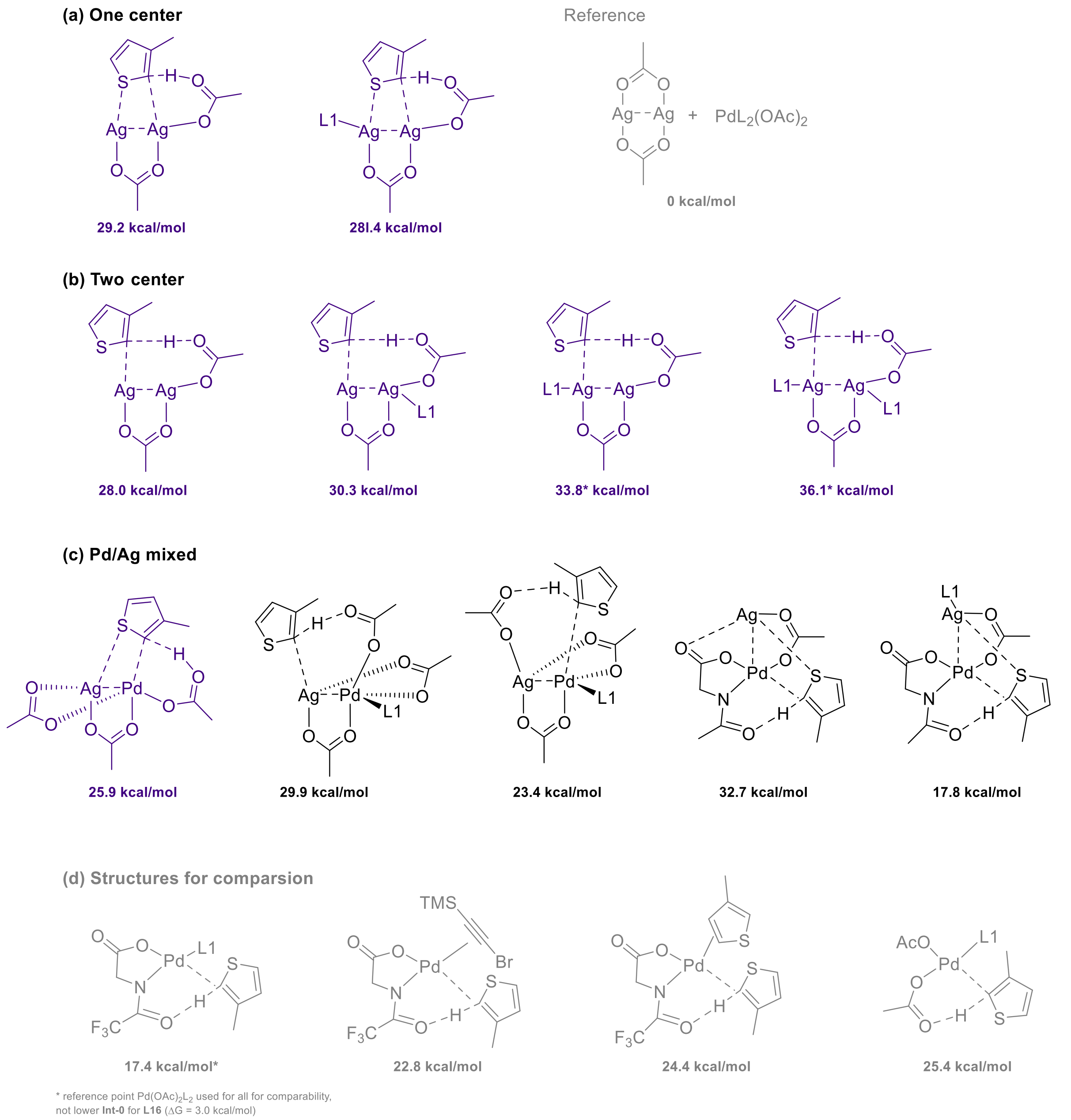

Figure S16: Silver mediated C–H activation. (a) TS involving two Ag atoms with the CMD base and the forming C–M bond at the same atom, (b) TS involving two Ag atoms with the CMD base and the forming C–M bond at two distinct atoms, (c) mixed Pd and Ag TS, (d) silver free TS structures for comparison. All calculated at the SMD(EtOH) PWPB96-D3(BJ)/def2-QZVPP//ωB97X-D3BJ/def2-TZVP level of theory.

All transition states at which the C–H activation occurs at a silver atom (formation of Ag–C bond) and which would potentially be more on the CMD/AMLA side or closer to the diagonal of the More O'Ferrall-Jencks diagram are higher in energy. The Ag mediated TS in which a Pd–C is formed are expected to follow selectivity patterns typical for eCMD/BIES regimes. Some selected structures are lower compared to the silver free, N-acyl amino acid free C–H activation. The structures involving silver acetate as a replacement for pyrazine with a bidentate N-acyl amino acid ligand are shown for completeness as well (Figure S16d). They are found to be higher in energy compared to the TS suggested *vide supra*.

It can be concluded that, structures involving a C–H activation over one or multiple silver centers are energetically less favorable than at the respective Pd center. The switch in selectivity or more generally a preference for C5 functionalization can therefore not be rooted in a different TS for C–H activation.

3.2 Switch in Turnover Limiting Step

The preliminary results in section 3.1 suggest that the origin for a switched selectivity could be rooted in a reversible C–H activation and an energetically similar migratory insertion. Since the C–H activation is conclusively found to be C2 selective (Figure S12c), the migratory insertion would require a preference for the C5 product and would need to be irreversible.

We probed several migratory insertion steps computationally. Migratory insertions using the same alkyne source are reported in the literature to proceed without the need of silver involvement.[51,57] Possible silver containing structures are discussed in more detail in section 3.4, Figure S36 and are found to be slightly higher in energy compared to silver free structures using the appropriate solvation model. Additionally, no preference of C5 over C2 was observed either.

Different coordination environments were considered (Figure S17) and the respective lowest rotamers is used. In addition to orienting the alkyne to form the MI product with identical connectivity with the product, also an inverse coordination is considered, as a possible silyl migration was suggested and deemed possible by Maiti and Paton.[57] For selected structures TIPS was used instead of TMS to ensure that the choice of model alkyne does not distort the conclusions.

Please note that the chosen solvation model likely does not capture the $\Delta G^{\ddagger}$ trends well for the MI step: acetate vs (*S*)-2-acetamido-3-phenylpropanoate show an energy difference of ~6.5 kcal/mol which seems very large for a seemingly similar monodentate coordination and rather similar acidity/donating ability. The positioning of silver containing structures was also found to be solvation dependent. The appropriate solvation model for **TS-4** vs **TS-5** will be discussed in more detail in section 3.3. For the relative energetics C2 vs C5 or isomers cis vs trans the solvation model is expected to be less influential.

Under the selected methodology (SMD(EtOH) PWPB95-D3(BJ)/def2-QZVPP//ωB97X-D3BJ/def2-TZVP) only **TS-Alkyne-MI-1** displayed a preference for C5, i.e. a lower Gibbs free enthalpy of the C5 TS. Energetically C2 is favored, and only a slight entropic difference appears to favor C5. Experimental controls indicate that other Pd-sources with different counteranions like trifluoroacetate or pivalate do not distort the selectivity (Figure S78) which indicates that also the carboxylate of N-acyl amino acid ligand can be operative as a monodentate ligand. The apparent preference for C2 over C5 selectivity was independent of solvation model (SMD or CPCM). Similar observations are made using SMD(EtOH) ωB97X-D3BJ/def2-TZVP for the energy evaluation. Interestingly, upon exclusion of the D3BJ contribution the C5 isomer appears to be favored in all cases. Please note that sometimes another low-energy conformer is preferred upon excluding the D3BJ contribution but at times the identical conformers result in an inverted energetic ordering. This effect is studies in more detail in the following part.

Alkyne MI

with AcO

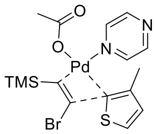

TS-Alkyne-MI-01

$\Delta\Delta G^{\ddagger}$ = 0.8 kcal/mol
$\Delta\Delta E^{\ddagger}$ = -0.4 kcal/mol
$\Delta G_{C2}^{\ddagger}$ = 17.2 kcal/mol
$\Delta\Delta E^{\ddagger *}$ = -0.7 kcal/mol
$\Delta\Delta E^{\ddagger **}$ = 1.3 kcal/mol
$\Delta G_{C2}^{\ddagger *}$ = 19.4 kcal/mol
= **TS-5-AcO**

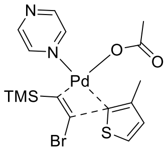

TS-Alkyne-MI-02

$\Delta\Delta G^{\ddagger}$ = -1.7 kcal/mol
$\Delta\Delta E^{\ddagger}$ = -1.8 kcal/mol
$\Delta G_{C2}^{\ddagger}$ = 17.1 kcal/mol
$\Delta\Delta E^{\ddagger *}$ = -1.7 kcal/mol
$\Delta\Delta E^{\ddagger **}$ = 0.6 kcal/mol
$\Delta G_{C2}^{\ddagger *}$ = 19.6 kcal/mol

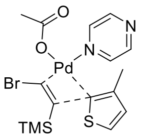

TS-Alkyne-MI-03

$\Delta\Delta G^{\ddagger}$ = -0.7 kcal/mol
$\Delta\Delta E^{\ddagger}$ = -1.0 kcal/mol
$\Delta G_{C2}^{\ddagger}$ = 17.0 kcal/mol

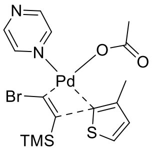

TS-Alkyne-MI-02

$\Delta\Delta G^{\ddagger}$ = -0.2 kcal/mol
$\Delta\Delta E^{\ddagger}$ = -0.1 kcal/mol
$\Delta G_{C2}^{\ddagger}$ = 17.8 kcal/mol

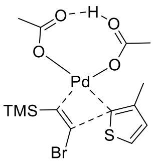

TS-Alkyne-MI-04

$\Delta\Delta G^{\ddagger}$ = -1.4 kcal/mol
$\Delta\Delta E^{\ddagger}$ = -1.8 kcal/mol
$\Delta G_{C2}^{\ddagger}$ = 21.4 kcal/mol

with TIPS

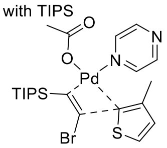

TS-TIPSAlkyne-MI-05

$\Delta\Delta G^{\ddagger}$ = -2.1 kcal/mol
$\Delta\Delta E^{\ddagger}$ = -2.2 kcal/mol
$\Delta G_{C2}^{\ddagger}$ = 17.2 kcal/mol
$\Delta\Delta E^{\ddagger *}$ = -2.4 kcal/mol
$\Delta\Delta E^{\ddagger **}$ = -1.4 (-0.2) kcal/mol
$\Delta G_{C2}^{\ddagger *}$ = 19.4 kcal/mol

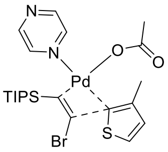

TS-TIPSAlkyne-MI-02

$\Delta\Delta G^{\ddagger}$ = -1.5 kcal/mol
$\Delta\Delta E^{\ddagger}$ = -1.4 kcal/mol
$\Delta G_{C2}^{\ddagger}$ = 17.8 kcal/mol
$\Delta\Delta E^{\ddagger *}$ = -0.6 kcal/mol
$\Delta\Delta E^{\ddagger **}$ = 0.1 kcal/mol
$\Delta G_{C2}^{\ddagger *}$ = 21.9 kcal/mol

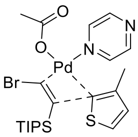

TS-TIPSAlkyne-MI-06

$\Delta\Delta G^{\ddagger}$ = 0.2 kcal/mol
$\Delta\Delta E^{\ddagger}$ = -0.9 kcal/mol
$\Delta G_{C2}^{\ddagger}$ = 21.9 kcal/mol

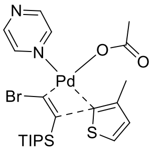

TS-TIPSAlkyne-MI-02

$\Delta\Delta G^{\ddagger}$ = -0.7 kcal/mol
$\Delta\Delta E^{\ddagger}$ = -1.1 kcal/mol
$\Delta G_{C2}^{\ddagger}$ = 21.9 kcal/mol

with L2 and L17

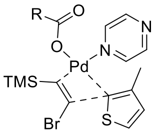

TS-Alkyne-MI-07

$\Delta\Delta G^{\ddagger}$ = -1.9 kcal/mol
$\Delta\Delta E^{\ddagger}$ = -1.3 (-1.9) kcal/mol
$\Delta G_{C2}^{\ddagger}$ = 13.5 kcal/mol
$\Delta\Delta E^{\ddagger *}$ = -1.5 (-1.7) kcal/mol
$\Delta\Delta E^{\ddagger **}$ = 0.5 kcal/mol
$\Delta G_{C2}^{\ddagger *}$ = 16.1 kcal/mol

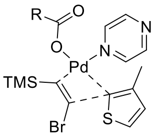

TS-Alkyne-MI-08

$\Delta\Delta G^{\ddagger}$ = -2.5 kcal/mol
$\Delta\Delta E^{\ddagger}$ = -2.8 (-1.9) kcal/mol
$\Delta G_{C2}^{\ddagger}$ = 10.7 kcal/mol
$\Delta\Delta E^{\ddagger *}$ = -2.2 (-2.0) kcal/mol
$\Delta\Delta E^{\ddagger **}$ = 0.1 (0.4) kcal/mol
$\Delta G_{C2}^{\ddagger *}$ = 13.6 kcal/mol

with Ag

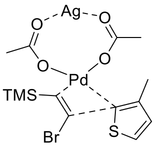

TS-Alkyne-MI-09

$\Delta\Delta G^{\ddagger}$ = -1.8 kcal/mol
$\Delta\Delta E^{\ddagger}$ = -2.2 kcal/mol
$\Delta G_{C2}^{\ddagger}$ = 15.5 kcal/mol
$\Delta\Delta E^{\ddagger *}$ = -2.0 kcal/mol
$\Delta\Delta E^{\ddagger **}$ = 0.2 kcal/mol
$\Delta G_{C2}^{\ddagger *}$ = 15.6 kcal/mol

with 3-phenylthiophene

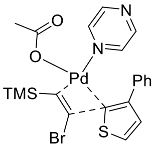

TS-PhAlkyne-MI-01

$\Delta\Delta G^{\ddagger}$ = -4.0 kcal/mol
$\Delta\Delta E^{\ddagger}$ = -4.9 kcal/mol
$\Delta G_{C2}^{\ddagger}$ = 15.2 kcal/mol
$\Delta\Delta E^{\ddagger *}$ = -2.0 kcal/mol
$\Delta\Delta E^{\ddagger **}$ = 0.2 kcal/mol
$\Delta G_{C2}^{\ddagger *}$ = 18.9 kcal/mol

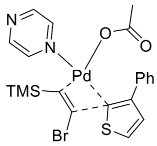

TS-PhAlkyne-MI-02

$\Delta\Delta G^{\ddagger}$ = -4.6 kcal/mol
$\Delta\Delta E^{\ddagger}$ = -4.6 kcal/mol
$\Delta G_{C2}^{\ddagger}$ = 15.8 kcal/mol

Olefin MI

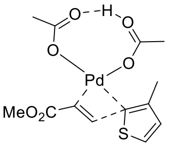

TS-Olefin-MI-01

$\Delta\Delta G^{\ddagger}$ = -1.4 kcal/mol
$\Delta\Delta E^{\ddagger}$ = -1.4 kcal/mol
$\Delta G_{C2}^{\ddagger}$ = 25.5 kcal/mol

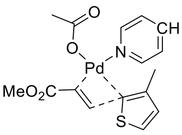

TS-Olefin-MI-02

$\Delta\Delta G^{\ddagger}$ = -1.6 kcal/mol
$\Delta\Delta E^{\ddagger}$ = -1.6 kcal/mol
$\Delta G_{C2}^{\ddagger}$ = 21.5 kcal/mol

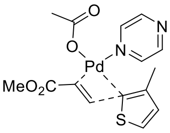

TS-Olefin-MI-03

$\Delta\Delta G^{\ddagger}$ = -1.5 kcal/mol
$\Delta\Delta E^{\ddagger}$ = -1.5 kcal/mol
$\Delta G_{C2}^{\ddagger}$ = 20.2 kcal/mol

C–H Activation

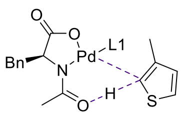

TS-MeAlkyne-CH-01

$\Delta\Delta G^{\ddagger}$ = -2.3 kcal/mol
$\Delta\Delta E^{\ddagger}$ = -2.6 kcal/mol
$\Delta G_{C2}^{\ddagger}$ = 14.1 kcal/mol
= **TS-4-L2**

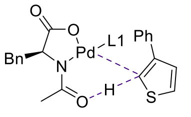

TS-PhAlkyne-CH-01

$\Delta\Delta G^{\ddagger}$ = -4.7 kcal/mol
$\Delta\Delta E^{\ddagger}$ = -5.2 kcal/mol
$\Delta G_{C2}^{\ddagger}$ = 12.3 kcal/mol
= **TS-4-L2-Ph**

SMD (EtOH) D3BJ PWPB96/def2-QZVPP//ωB97X-D3BJ/def2-TZVP; Reference: PdL_2OAc_2
* SMD (EtOH) ωB97X-D3BJ/def2-TZVP//ωB97X-D3BJ/def2-TZVP
** SMD (EtOH) ωB97X/def2-TZVP//ωB97X-D3BJ/def2-TZVP
Values in without parenthesis: lowest free energy structures, in parenthesis: lowest electronic energy structures; sometimes different in case very similar energies

C2 selective: $\Delta\Delta G^{\ddagger} < 0$

Figure S17: Different isomers and structures for a potential MI of silylated bromoalkyne.

Benchmarking and Comparison of MI for Olefination and Alkynylation

Since experimentally a selectivity switch is also observed using ethyl acrylate instead of alkyne under otherwise identical reaction conditions (Figure S77), the respective structures were also investigated. These structures resemble the structures recently computed by Carrow et al.[50] who computationally predict C5 selectivity (for energies and free energies). The authors use PCM(AcOH) BP86/6-31G* with SDD for Pd as computational approach for geometries, frequencies, and single point energies. Several things are to be noted: a rather small basis set is chosen for energy calculations, additionally no dispersion correction is being applied, the reaction is computed at 298.15 K and 1 atm, and not at the reaction relevant solution state and temperature of 60°C or 40°C. The authors do not provide arguments to why this specific functional basis set combination and procedure is chosen which diverts from the generally recommended approach for unknown/unbenchmarked systems in solution.[31,33]

We consequently reproduced the results from Carrow using their optimized structure and found that their computational approach indeed predicts C5 selective MI for the olefination. Using ωB97X-D3BJ/def2-TZVP geometries in combination with the BP86 functional also predicts a preference for the C5 isomer even with larger bases sets (def2-TZVP) (see Table S5).

Table S5: Relative energies of ΔΔ$E^{\dagger}$ in kcal/mol for the acrylate MI **TS-Olefin-MI-02**. Values >0 indicate a preference for C5. Comparison of basis set dependency of the originally reported structure and the reoptimized structure. SMD(AcOH) is used for the energy calculations. The reference energy calculated in the literature with Gaussian at the PCM(AcOH) BP86/6-31G* level is also indicated.

Structure	Gaussian (Lit)	BP86/**6-31G* SDD**	BP86/**def2-SVP**	BP86/**def2-TZVP**
Lit. PCM(AcOH) BP86/6-31G*	(0.5)	0.38	-0.28	0.44
Reopt ωB97X-D3BJ/def2-TZVP		0.96	0.51	0.95

One striking difference between both approaches SMD PWPB96-D3(BJ)/def2-QZVPP//ωB97X-D3BJ/def2-TZVP vs PCM BP86/6-31G* and SDD is the absence of an empirical dispersion correction. This effect was investigated in Table S6. A complete reversal in predicted lower energy structure is observed upon switching on dispersion correction. The differences between D3BJ and D4 are rather small on the other hand.

Table S6: Relative energies of ΔΔ$E^{\dagger}$ in kcal/mol for the acrylate MI **TS-Olefin-MI-02**. Values >0 indicate a preference for C5. Comparison of dispersion interaction effects of the originally reported structure and the reoptimized structure. SMD(AcOH) is used for the energy calculations.

Structure	BP86/def2-TZVP	BP86-**D3(BJ)**/def2-TZVP	BP86-**D4**/def2-TZVP
Lit. PCM(AcOH) BP86/6-31G*	0.44	-1.90	-1.55
Reopt ωB97X-D3BJ/def2-TZVP	0.95	-1.68	-1.31

Another difference is the implementation of solvation model PCM vs SMD. The use of another polarizable continuum model (CPCM) as implemented in ORCA did not outweigh the differences induced by the presence or absence of dispersion correction, even though a slight preference for a higher selectivity for the C5 isomer for CPCM model in combination with BP86 is observed (Table S7).

Table S7: Relative energies of ΔΔ$E^{†}$ in kcal/mol for the acrylate MI **TS-Olefin-MI-02**. Values >0 indicate a preference for C5. Comparison of solvation model effects of the originally reported structure and the reoptimized structure. SMD(AcOH) is used for the energy calculations.

	**SMD**-BP86/6-31G* SDD	**CPCM**-BP86/6-31G* SDD	**SMD**-BP86-D3(BJ)/def2-TZVP	**CPCM**-BP86-D3(BJ)/def2-TZVP
Orig	0.38	0.61	-1.90	-1.66
Reopt	0.96	1.17	-1.68	-1.46

To gain more insight in the discrepancy between lower level and higher level of theory, we carried out an energy benchmark using several DFT functionals for structure **TS-Olefin-MI-02** optimized at the ωB97X-D3BJ/def2-TZVP level of theory (Table S8). The results point conclusively to the fact that upon using methods with either intrinsic empirical dispersion correction terms like the Minnesota functionals or with functionals in combination with Grimmes dispersion correction and Becke-Johnson damping (D3BJ) the C2 structure seemed to be favored.

Table S8: Relative energies of ΔΔ$E^{†}$ in kcal/mol for the acrylate MI **TS-Olefin-MI-02**. Values >0 indicate a preference for C5. Comparison of the DFT functional/method for the original and reoptimized structure. SMD(AcOH) is used for the energy calculations.

SMD(AcOH)	**BP86**/6-31G* SDD	**M06L**/def2-TZVP	**wB97X-D3BJ**/def2-TZVP	**PWPB96-D3(BJ)** /def2-QZVPP	**MP2**/def2-QZVPP
Orig	0.38	-1.83	-1.93	-1.83	-2.02
Reopt	0.96	-1.97	-1.54	-1.58	
Reopt Free Energy	1.47	-1.46	-1.03	-1.07	

The effect of solvation AcOH vs EtOH was probed due to the different solvent systems in our and Carrows study (Table S9).

Table S9: Relative energies of ΔΔ$E^{†}$ in kcal/mol for the acrylate MI **TS-Olefin-MI-02**. Values >0 indicate a preference for C5. Comparison of the DFT functional/method for the original and reoptimized structure. SMD(EtOH) is used for the energy calculations.

SMD(EtOH)	**BP86**/def2-TZVP	**B3LYP**/def2-TZVP	**B3LYP-D3BJ**/def2-TZVP	**M06L**/def2-TZVP	**M06**/def2-TZVP	**M06-2x**/def2-TZVP
Reopt	1.39	1.52	-0.96	-1.43	-1.39	-1.43

Finally, the effects on the alkyne MI with **TS-Alkyne-MI-01** optimized at the ωB97X-D3BJ/def2-TZVP level were also probed and the same effect as for the olefination is observed: all DFT functionals with intrinsic or additionally added dispersion correction point towards the C2 TS as more stable (Table S10). Functionals with different amount of HF exchange, MP2, double hybrid and especially also coupled cluster with complete basis set extrapolation SMD(EtOH) DLPNO-CCSD(T)/CBS calculations predict a preference for C2. The small deviation between the CCSD(T) and our previously chosen double hybrid method are pleasing but do not rationalize the experimental results. We still consider the chosen approach chosen by Carrow to be unsatisfactory since a lower level of theory and potential cancellation of error would arbitrarily yield seemingly correct results.

Table S10: Relative energies of $\Delta\Delta E^{\dagger}$ in kcal/mol for the alkyne MI **TS-Alkyne-MI-01**. Values >0 indicate a preference for C5. Comparison of the DFT functional/method for the original and reoptimized structure. SMD(EtOH) is used for the energy calculations.

SMD(EtOH)	**BP86**/def2-TZVP	**M06L**/def2-TZVP	**M06**/def2-TZVP	**M062X**/def2-TZVP	**wB97XD**/def2-TZVP	**PWPB95**/def2-QZVPP	**RI-MP2**/def2-QZVPP	**CCSD(T)**/CBS
Alkyne	1.52	-0.03	-1.03	-0.08	-0.75	-0.43	-2.07	-0.43

Since extensive literature benchmark on vastly different systems have in almost all cases shown a beneficial effect of dispersion corrected functionals[46,58] and since the results are in line with the high level CCSD(T) results, the effect of functional on the geometry was investigated. Several functionals with and without dispersion correction were employed to see the effect of dispersion correction on the relative energy difference since we hypothesized that potentially a poor description of the structure could lead to erroneous energies.

To be sure that appropriate functionals are chosen, a few literature reported crystal structures[59–65] were used and subjected to a functional benchmark. Factors like root mean square deviation (RMSD), deviation in bond distances, etc. were used to evaluate the performance of the respective functional. For structures without CCDC number the tabulated bond distances, etc were used, but an RMSD could not be determined. Please note that crystal structures can be distorted due to crystal packing effects compared to gas phase geometries. These on the other hand can again change compared to solution. Figure S18 highlights the relevant geometries that we assessed to have a certain structural similarity with the relevant intermediates in the C–H activation and the migratory insertion (see ZENODO archive for individual deviations for benchmark A and B separately and structures).

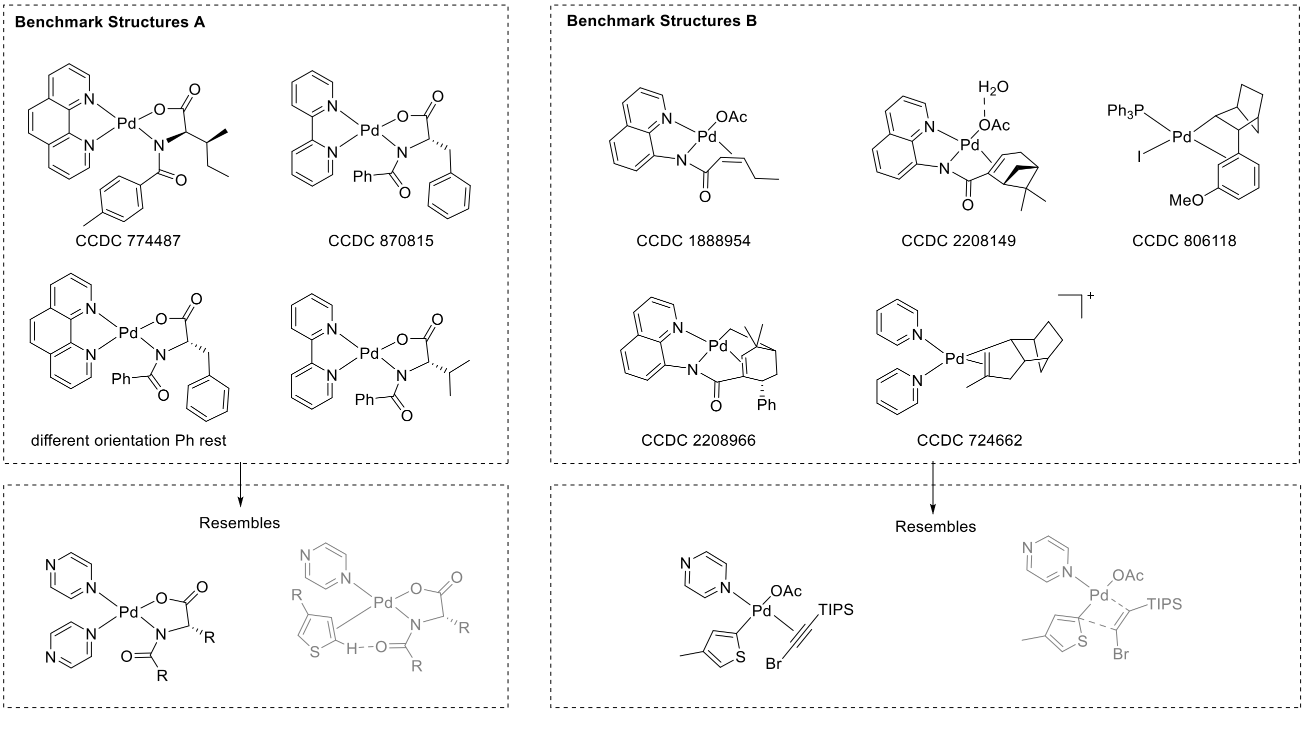

Figure S18: Structures considered for the benchmark study divided into a set A that resembles the intermediate prior to C–H activation (resting state) and B that resembles more the intermediate prior to the MI.

Several functionals with and without D3BJ were employed in combination with the def2-TZVP basis set and corresponding effective core potential (ECP), which we consider sufficiently large and at the same time still computationally feasible. In order to obtain functionals that are relevant for the MI and at the same time for the C–H activation the results for benchmark structures A and B (see Figure S18) were averaged and weighted as 1:1.

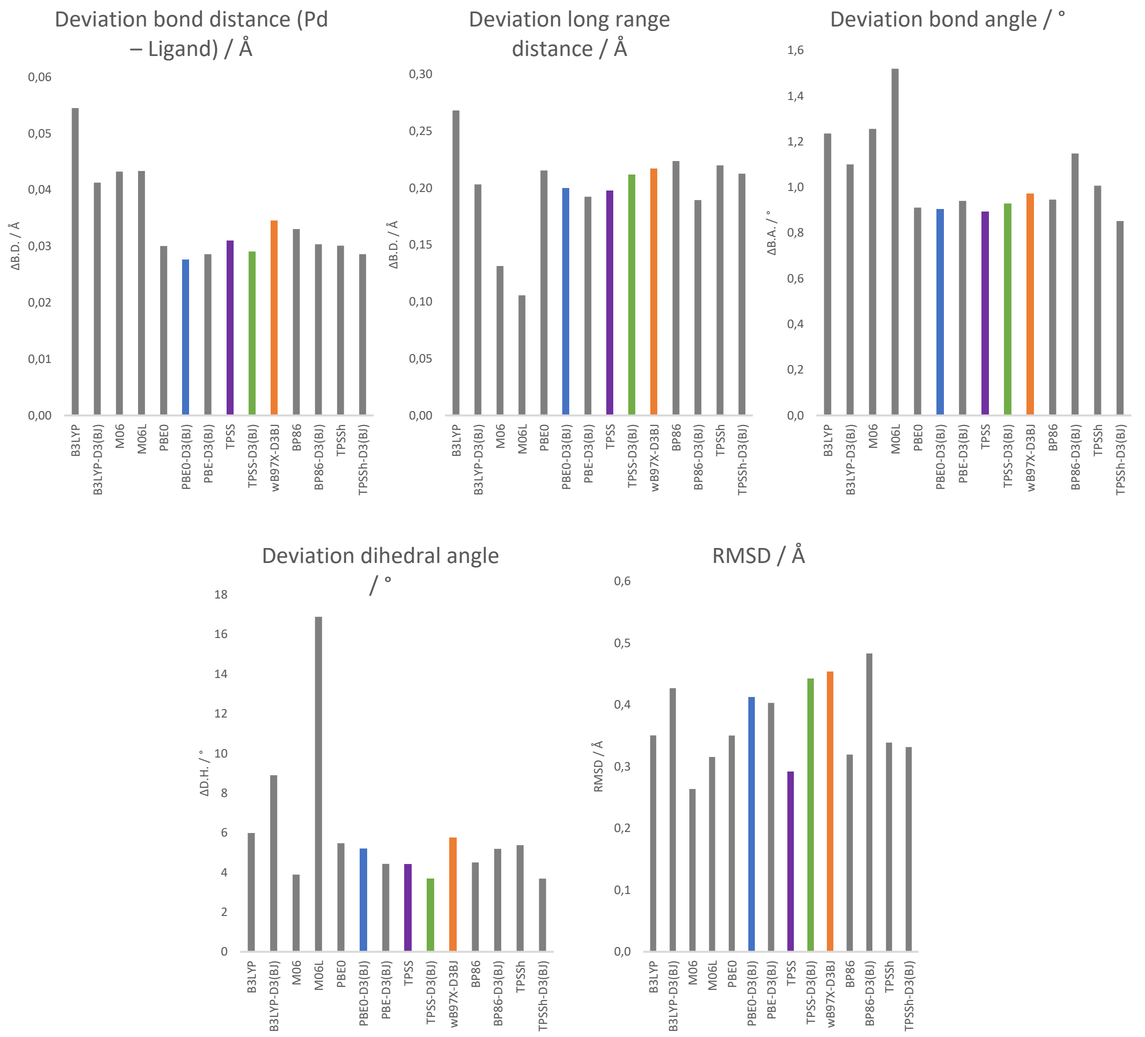

Figure S19: Results of the structural benchmark using different DFT functionals in combination with the def2-TZVP basis set. The deviation in the respective parameters from the crystal structures of benchmark sets A and B were weighted as 1:1.

Looking at the results it becomes apparent that the ωB97X-D3BJ functional employed so far is not the best performer but shows reasonable deviation from e.g. experimental bond distances. PBE0-D3(BJ) was identified as a well performing functional and TPSS as a functional without dispersion correction. Both functionals were used with and without dispersion correction to reoptimize **TS-Alkyne-MI-01** and single point energies were calculated at the SMD(EtOH) PWPB95-D3(BJ)/def2-QZVPP level of theory. The functional indeed has an influence on the selectivity (Table S11). Switching on dispersion correction in the geometry optimization actually led to a different prediction in case of PBE0. Here, D3BJ free optimization predicts C5 to be more stable by 0.4 kcal/mol whereas PBE0-D3BJ predicts it to be destabilized by -0.2 kcal/mol. It is known that dispersion correction for geometry optimizations leads to more compact structures and often better experimental agreement with gas phase structures.[66]

To test the potential hypothesis that *inclusion of dispersion correction in the geometry optimization only improperly accounts for the solution state structure* some additional tests were performed. The hypothesis could potentially hold true since in the benchmark no prohibitively large performance differences in e.g. the bond distance for dispersion corrected and non-corrected functionals were detected.

To probe the effect more thoroughly and avoid "cherry picking" results with e.g. cancellation of errors, we also subjected the substrate **1-Ph** to optimization using dispersion corrected and uncorrected functionals. The effect observed for **1-Me** is also visible: a lower degree of preference for C2 is observed using non-dispersion corrected functional, but qualitatively still a preference for the C2 isomer is observed.

The **1-Me** structures were then reoptimized with implicit solvation turned on at the CPCM(EtOH) level with def2-TZVP and SMD(EtOH) and def2-SVP (for the latter for some functionals only numerical gradients used to be available rendering frequency evaluation at the def2-TZVP level cumbersome). Implicit solvation during geometry optimization seems to render the C2 product slightly more favorable. Here the trend that dispersion corrected functionals lead to more C2 product is also pronounced but qualitatively no overall C5 product was observed.

Table S11: Relative energies of $\Delta\Delta E^{\dagger}$ in kcal/mol. Values >0 indicate a preference for C5. Reoptimized structures at the indicated DFT level with def2-TZVP unless otherwise indicated and without dispersion correction and single point energy evaluation at the SMD(EtOH) PWPB95-D3(BJ)/def2-QZVPP and (CPCM(EtOH) ωB97X-D3BJ/def2-TZVP).

**Structure with def2-TZVP**	**TS-Alkyne-MI-01**	**TS-PhAlkyne-MI-01**	**TS-4-L16**
TPSS	-0.02, (-1.44)	-2.55, (-5.19)	-2.18, (-2.93)
TPSS-D3(BJ)	-0.64, (-1.68)	-3.17, (-5.24)	-2.67, (-3.28)
PBE0	0.36, (-0.68)	-1.97, (-2.57)	-2.28, (-2.98)
PBE0-D3(BJ)	-0.17, (-1.06)	-3.77, (-3.85)	-2.63, (-3.24)
PBE0-D3(BJ) (tightopt)	-0.31, (-1.16)		
PBE0-D4	-0.23, (-1.17)		
PBE0-D3(BJ)/def-SVP	-0.29, (-1.23)		
CPCM PBE0	-1.15, (-1.49)		
CPCM PBE0 (tightopt)	-1.16, (-1.41)		
SMD PBE0/def2-SVP	-1.20, (-1.43)		
SMD PBE0-D3(BJ)/def2-SVP	-1.60, (-1.40)		
ωB97X-D3BJ	-0.43, (-0.74)	-5.6, (-3.28)	-2.58, (-3.15)
ωB97X-D3BJ (tightopt)			-2.79, (-3.29)
ωB97X	-0.18, (-0.85)		
CPCM ωB97X-D3BJ	-1.34, (-1.21)		-2.83, (-3.41)
SMD ωB97X-D3BJ			-2.96, (-3.42)
B3LYP	0.82, (-0.17)	-3.44, (-3.27)	-2.77, (-3.34)
B3LYP-D3(BJ)	-0.09, (-0.86)		-2.29, (-3.00)

Overall, it seems more likely that with the methods chosen for energy evaluation, the C2 isomer is indeed favored. It nevertheless also becomes evident that the geometry is more sensitive to the functional chosen. This becomes apparent when comparing to the overall minor differences in the C–H activation step (Table S11).

Since the initial hypothesis introduced above does not hold up, we subsequently returned to the energy evaluation, now with both the methyl and phenyl variant **1-Me** and **1-Ph** with **L2** and **L16** for both the C–H activation and the MI step in Figure S17.

PBE0/def2-TZVP and ωB97X /def2-TZVP were chosen with and without dispersion correction to evaluate the energies again since e.g. PWPB96 already intrinsically accounts for a certain amount of dispersion correction due to the MP2 part of the double hybrid functional.

Table S12: Relative energies of ΔΔ*E*† in kcal/mol. Values >0 indicate a preference for C5. Optimized structures at the ωB97X-D3BJ/def2-TZVP level with energies with and without dispersion correction and with SMD(EtOH) unless otherwise specified.

**ΔΔ*E*†**	**TS-4-L2** R=Me	**TS-4-L2** R=Ph	**TS-4-L16** R=Me	**TS-4-L16** R=Ph	**TS-5-AcO** R=Me	**TS-5-AcO** R=Ph
ωB97X/def2-TZVP	-1.2	-0.35	-1.8	-0.2	1.0	3.3
ωB97X-D3BJ/def2-TZVP	-3.1	-4.5	-3.1	-5.2	-0.8	-3.9
PBE0/def2-TZVPP	-1.7	-0.5	-1.2	-0.6	0.9	3.0
D3BJ PBE0/def2-TZVPP	-2.7	-4.6	-2.5	-4.2	-0.4	-2.9
D4 PBE0/def2-TZVPP	-2.6	-4.7	-2.4	-4.2	-0.3	-2.9
NL PBE0/def2-TZVPP	-2.6	-4.4	-2.5	-4.0	-0.2	-2.4
SCNL PBE0/def2-TZVPP	-2.7	-4.4	-2.5	-4.0	-0.2	-2.5
Nosolvent PBE0-D3(BJ)/def2-TZVPP	-2.4	-4.0	-2.3	-4.2	-1.1	-4.0
CPCM PBE0-D3(BJ)	-2.7	-4.2	-2.4	-4.0	-0.8	-2.5
dCOSMORS PBE0-D3(BJ)/def2-QZVPP**	-2.6	-3.9	-2.3	-3.6	-0.4	-1.7
dCOSMORS PBE0-D3(BJ)/def2-TZVPP***	-2.7	-4.1	-2.4	-3.7	-0.3	-1.8
openCOSMORS D3BJ****	-2.7	-4.1	-2.4	-4.2	-0.9	-2.1
Exp.*			-1.2	-1.3	1.0	1.6

* assuming **TS-4** is responsible for selectivity for **L16** and **TS-5** for **L2**. **with Turbomole. ***Turbomole solvent correction added to orca energies. ****openCOSMORS calculation done with ORCA6.

It is striking that e.g. PBE0 results in an energy difference much closer to the experimentally observed values without dispersion correction than with dispersion correction. This is not only true for the MI (relevant for **L2**) but also for the C–H activation step (relevant for **L16**). In case of the MI an inversion in predicted selectivity is observed for both Me and Ph substituted thiophene and for the C–H activation a lower preference for C2 but still a large enough difference is observed.

Djukic and Grimme noted a shortcoming of D3 correction in a study of Pd complexes: "*In the case of metals, the electron density around the metal atom in these model systems will be larger than it is the case for typical organometallic compounds. This leads to too large C6 coefficients and to a systematic overestimation of the related dispersion energy.*"[67] The conceptually different VV10 approach to account for dispersion correction has been shown to perform well and outperform D3 for certain transition metal systems.[46,68] The newer

version D4 also accounts for altered partial charges.[69] Both dispersion schemes (VV10 correction = NL and self-consistent treatment = SCNL) do not alter the relative selectivity strongly (Table S12).

As outlined before, we do not propose, especially in regard of the CCSD(T) results that e.g. PBE0 is a more suitable functional.

Different basis set combinations were tested to ensure the absence of a significant effect of the nature or size of the basis set (e.g. basis set superposition error). Additionally, the use of ECPs was scrutinized by reverting to relativistic ZORA calculations for **TS-5-AcO** (Table S13) and using different ECPs. When not explicitly mentioned, the default def2-ECP was used. Neither the use of ECPs or the use of adequately large basis sets (>def2-TZVP) seem to have an impact on the relative energies and even relative energies at the def2-SVP seem to be perfectly in line with larger basis sets. Different, even tighter grid settings and convergence thresholds were also probed in the beginning of the study for both the optimization and energy evaluation but lead to virtually unchanged results for the relative energies.

Table S13: Relative energies of $\Delta\Delta E^{\dagger}$ in kcal/mol for the alkyne MI using SMD(EtOH) with default def2-ECP in case not state otherwise. Values >0 indicate a preference for C5. Optimized structures at the ωB97X-D3BJ/def2-TZVP level.

**ΔΔ*E*†**	**TS-5-AcO** R=Me
ωB97X-D3BJ/def2-SVP	-0.75
ωB97X-D3BJ/def2-SVPD	-1.23
ωB97X-D3BJ/def2-TZVP	-0.75
ωB97X-D3BJ/def2-TZVPP	-0.75
ωB97X-D3BJ/def2-TZVPD	-0.76
ωB97X-D3BJ/def2-TZVPPD	-0.76
ωB97X-D3BJ/def2-QZVP	-0.74
ωB97X-D3BJ/def2-QZVPP	-0.74
ωB97X-D3BJ/def2-QZVPD	-0.74
ωB97X-D3BJ/aug-cc-pVQZ,SDD(Pd)	-0.75
ωB97X-D3BJ/aug-cc-pVQZ, aug-cc-pVQZ(Pd)	-0.71
ZORA ωB97X-D3BJ/def2-TZVPP *	-0.75

* using *ZORA*, *autoaux* keywords and *NewGTO Pd "SARC-ZORA-TZVPP"*, *NewAuxJKGTO Pd "AutoAux"*, *decontract true*.

Adequately accounting for solution seems rather likely to be the problem since on the one hand solution state structures are difficult to obtain experimentally and very few benchmarks are available. On the other hand, several studies using conformer ensembles determined in solution using VCD or NMR[70] point towards the shortcomings of traditional DFT with implicit solvation that often cannot reproduce the experimental conformer ranking.[71–73] Here sometimes non-dispersion corrected functionals outcompete dispersion corrected functionals.

The most likely scenario is therefore the inadequate consideration of solvation effects rather than a shortcoming of dispersion correction. A similar discussion in a response by Grimme[74,75] shows this for ligand binding energies. Here the more physically sound COSMO-RS model actually restores qualitatively the experimental trends. It was therefore probed whether the dCOSMO-RS model as implemented in Turbomole or openCOSMO-RS[76,77] as implemented in the newest version of ORCA (ORCA 6.0)[30] would lead to improved selectivities. Interestingly the qualitative prediction of selectivity still seems to favor C2 over C5 even though

they seem to attenuate it to some extent: comparing nosolvent (-4.0 kcal/mol) with CPCM (-2.5 kcal/mol), SMD (-2.9 kcal/mol), dCOSMORS (-1.8 kcal/mol), openCOSMORS (-2.1 kcal/mol) for **TS-5-AcO** with **1-Ph** shows a less pronounced preference for C2 in the solvated cases (Table S12). For SMD and CPCM different ways to calculate the solvent accessible surface apart from the default vdw_gaussian surface and gaussian charge scheme were attempted like the gepol_ses, gepol_sas, gepol_ses_gaussian, as well as a smaller solvent probe radius and a different scaling factor for the atomic radii with otherwise default settings as implemented in ORCA 5.0.2 and the newly implemented DRACO (dynamic adjustment for continuum solvation)[78] in ORCA 6.0.0, all of which gave similar results for TS02_Me and TS02_Ph respectively.

LED Analysis and Quantification of Dispersion Effects

Our working hypothesis is that the dispersion interaction with the solvent is not adequately accounted for using implicit solvation. Several literature reports[79–83] study exactly this comportment e.g. between two specially designed experimental conformers that in the context are often termed “molecular balance”. Chen et al.[84] for instance show that inter- and intramolecular dispersive interactions are attenuated about 70% in dichloromethane and that this behavior is not adequately accounted for with either SMD or COMSO-RS. The intra- and intermolecular dispersive interactions are ergo outcompeted by the solvent solute dispersive interactions. Another recent study by Chen points towards an overestimation of dispersion interaction by D3 of alkyl fragments for geometry and especially an erroneous conformer ranking in the ground state upon inclusion of dispersion interaction even in the gas phase.[85]

Due to the rather small and simple nature of the catalyst, different levels of solvent exposed surface are imaginable with the C5 isomers showing larger solvent exposed areas whereas the C2 isomers have a larger potential for intramolecular dispersion interactions (Figure S20a).

To investigate if a potential truncation of intramolecular dispersive interactions could be the root for the discrepancy between experimental and observed selectivity, the LED scheme developed by Bistoni and Neese was used to decompose the structures in their relative energy contributions.[86] It has been sown in the literature as a useful tool to gain insight into catalytically relevant transition metal complexes[87] and transition states.[88]

The LED analysis was done for the C–H activation (Figure S20c) and for the migratory insertion (Figure S20d) with structure **TS-5-AcO** for **1-Me** and **1-Ph** at the DLPNO-CCSD(T)/def2-TZVPP level of theory in the gas phase. The following artificial model reactions were chosen to disentangle the molecular parts. Please note that this serves as a qualitative study to investigate dispersion and other non-dispersion effects since for example other implicit solvent effects are not considered and only a triple zeta basis set was chosen. For **TS-4** both the hypothetical neutral and “anionic” coordination to the ligand were investigated since the former seems to be physically more sensible but the latter offers a higher comparability with the MI TS.

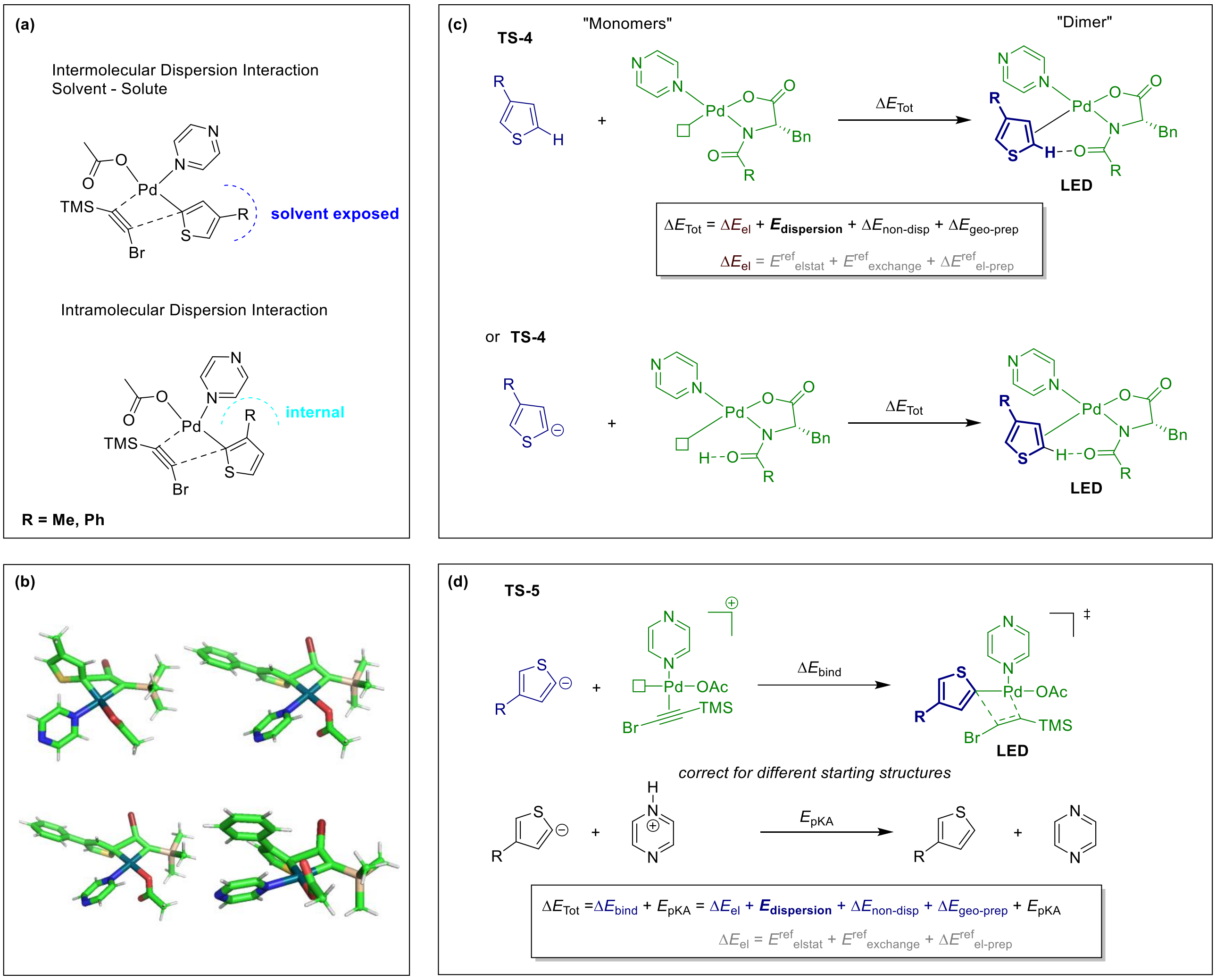

Figure S20: (a) Potential origin for internal dispersion interaction due to differently solvent exposed molecule parts. (b) Structures used for LED analysis of **TS-4** and **TS-5** to study LED interactions of C2 and C5 for **1-Me** and **1-Ph**. (c) Procedures for **TS-4** with either anionic or neutral thiophene coordination, (d) procedure of anionic thiophene for **TS-5-AcO**.

With the LED scheme the total energy for binding (monomers vs dimer) can be decomposed into several terms. Here, especially the dispersion term is of interest. We additionally introduced a “pK_A” term for the model reaction shown in Figure S20d to compare the differences in binding energy between C2 and C5. This is relevant since the deprotonated versions in C2 and C5 are not one common starting point and have different energies to begin with. An example of the total binding energy decomposition for **TS-5-AcO** is given in Figure S21 for C2 and C5. Here the different contributions like electric, geometric preparation (geo prep), etc are very large and a comparison between C2 and C5 is difficult. Additionally, the total binding energy values are somewhat arbitrary, since covalent rather than non-covalent bonding is being investigated and a dissociation of anionic thiophene seems rather unlikely which is also mirrored in the larger overall values ($\Delta \underline{E}_{Bind}$ C2: -71.4 kcal/mol, C5: -69.8 kcal/mol).

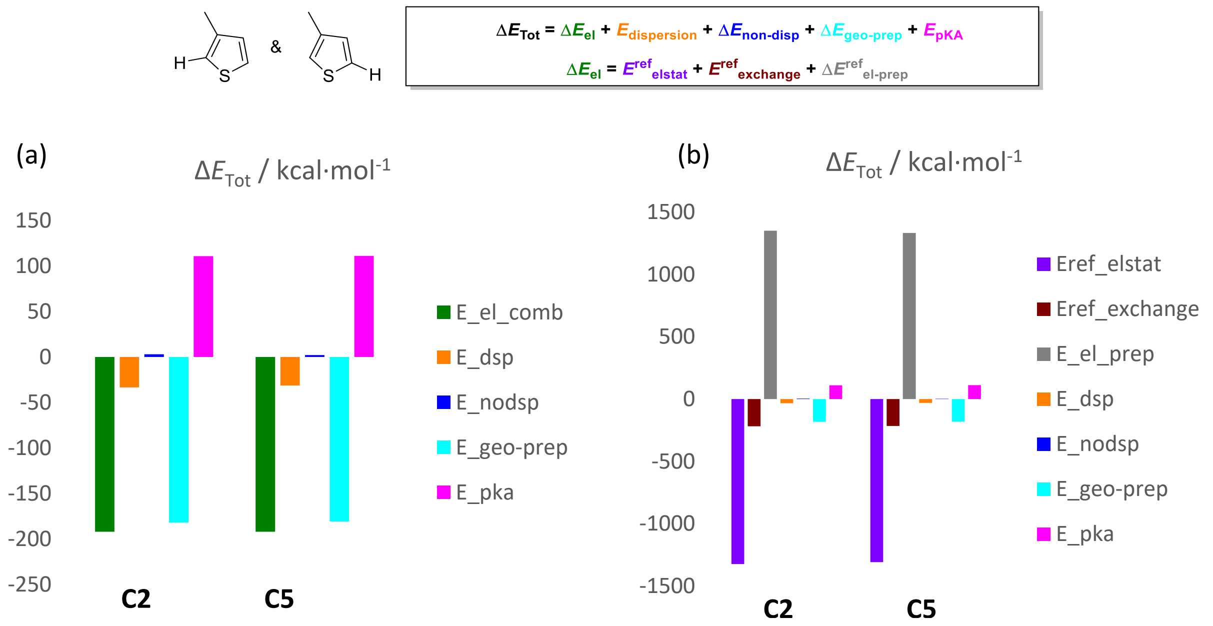

Figure S21: Overall LED contributions for the overall binding energy and decomposition into individual contributions. (a) Contributions from combined electronic contributions, dispersion contribution, non-dispersive contribution, geometry preparation, and "pKa". (b) with additional dissection of the different electronic contributions.

More interesting than the overall energy values for binding is the difference between C2 and C5 for the individual contributions. Here the actual effects for a larger stability of one vs the other isomer can be disentangled. Figure S22 shows this nicely for **TS-4** and **TS-5-AcO** for **1-Me**.

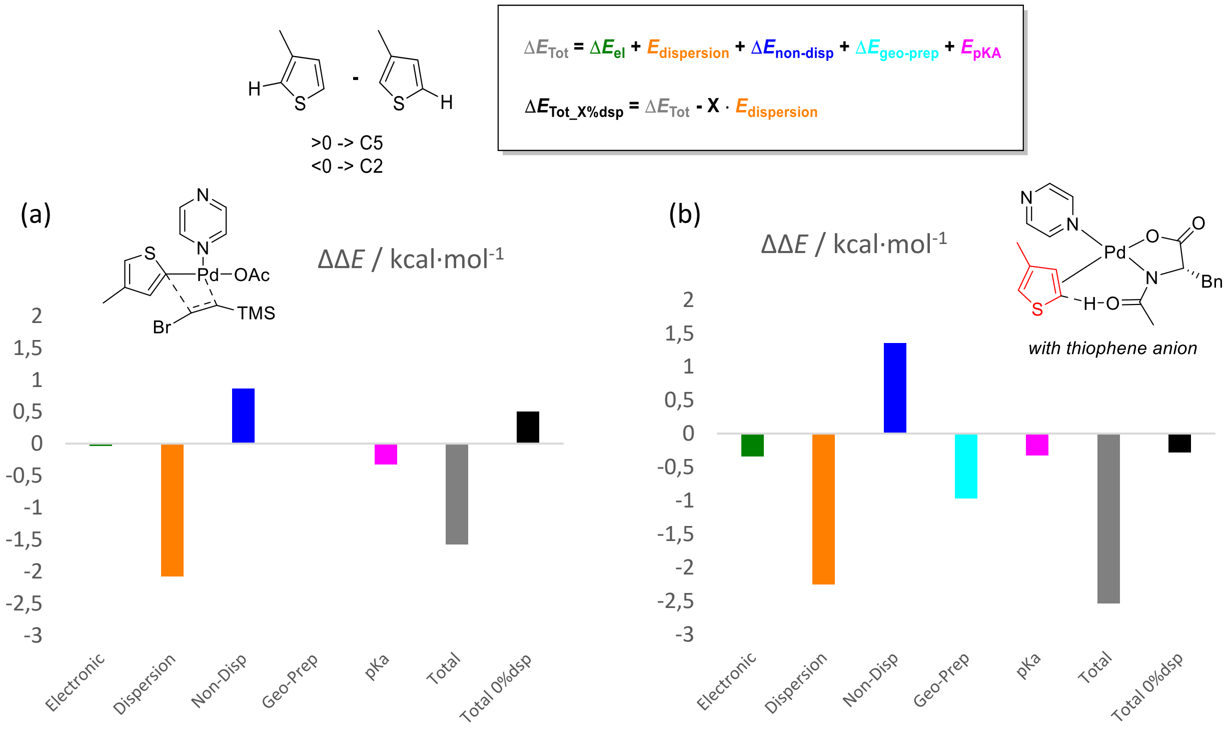

Figure S22: Comparison if the individual energy contributions of C2 vs C5 of (a) **TS-5-AcO** and (b) **TS-4-L2** for **1-Me** anion.

Especially the dispersive term leads to a preference of C2 over C5. Completely attenuating the dispersive interactions of the thiophene binding actually leads to a qualitatively correct prediction of selectivity.

Combining the non-steric terms (electronic, geoprep[3], pKa) in Figure S23 one can see that still dispersion interaction plays a major role in case of **TS-5-AcO**. In **TS-4-L2** other factors like geoprep are also of importance. In fact, these "electronic" effects are the reason for preference of C2 even in the absence of a dispersive contribution. The non-dispersive term (which contains the "steric" repulsion from the HF term)[87,89] actually favors C5. The numerical values of the individual contributions slightly change depending on whether the thiophene anion (including the pKA term) or the neutral thiophene is used (Figure S23 b vs c) but the qualitative trends remain virtually unchanged.

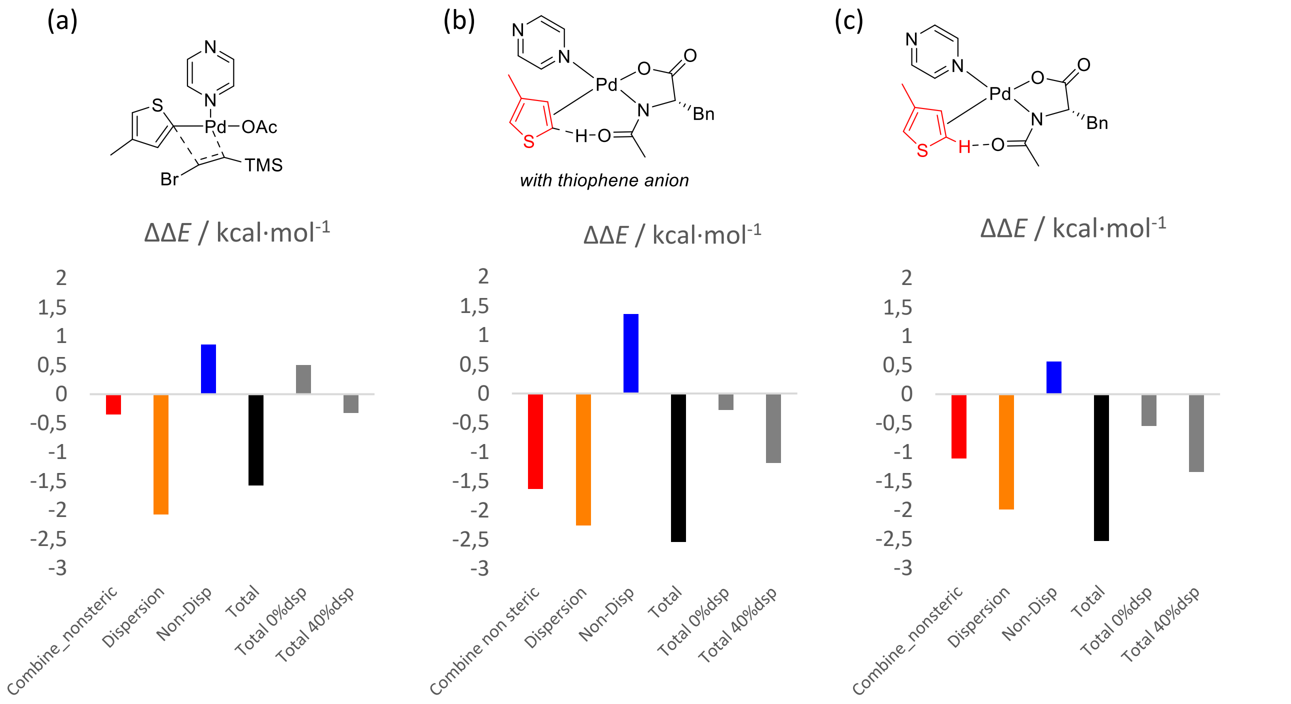

Figure S23: Comparison if the individual energy contributions of C2 vs C5 with combined non-steric contributions to better compare dispersion effects of (a) **TS-5-AcO** and (b) **TS-4-L2** for **1-Me** anion and (c) **TS-4-L2** with **1-Me**.

Similar conclusions can be drawn for **1-Ph** (Figure S24). Here the overall contribution of dispersion is larger (~2 vs ~10 kcal/mol).

[3] Geoprep refers to the electronic energy difference of distorting the separated monomer fragments in dimer geometry vs the fully relaxed monomer geometries and hence experiences mainly electronic but admittedly partially geometric influences.

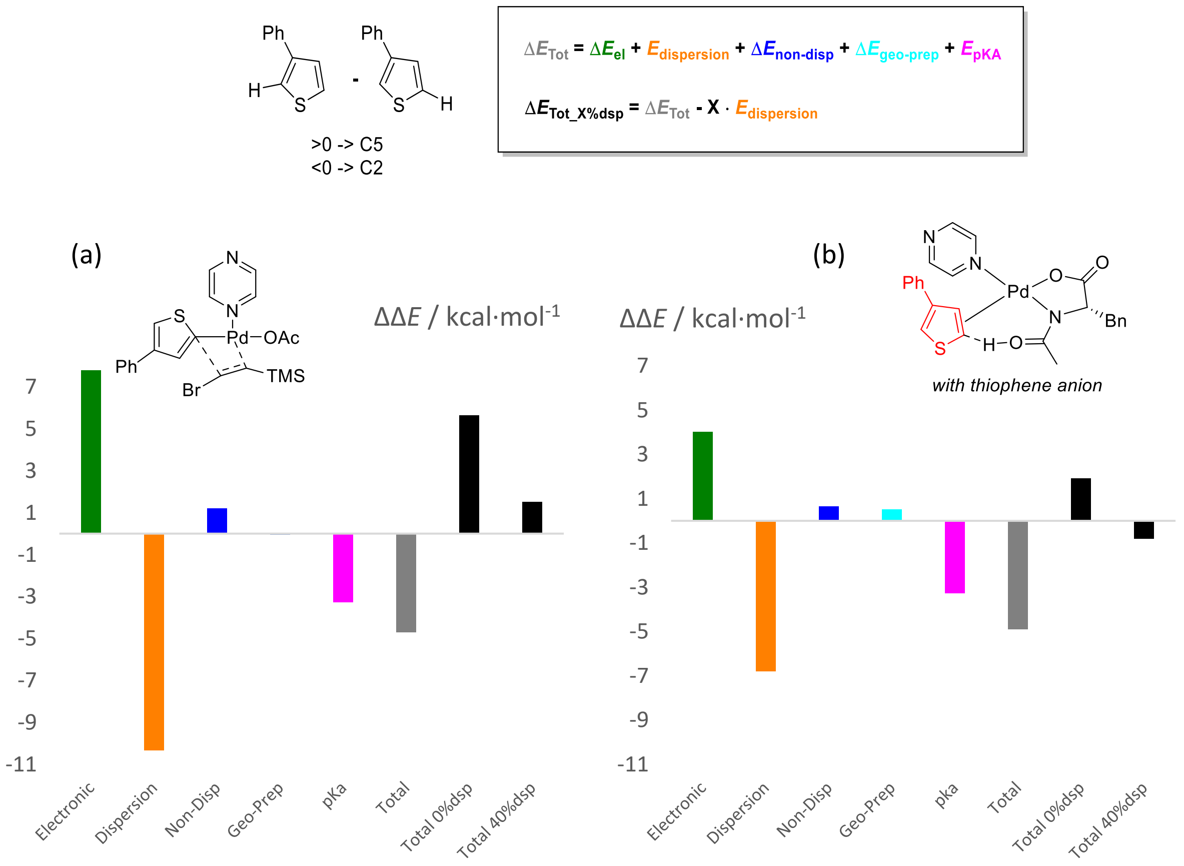

Figure S24: Comparison if the individual energy contributions of C2 vs C5 of (a) **TS-5-AcO** and (b) **TS-4-L2** for **1-Ph** anion.

Assuming a complete truncation of dispersive interaction of the methyl group in Figure S22 would lead to a $\Delta\Delta G^{\ddagger}$ of C2 vs C5 of -0.3 kcal/mol in case of the C–H activation and a $\Delta\Delta G^{\ddagger}$ of 0.5 kcal/mol for the MI. Please not that due to computational demands of the LED analysis only a def2-TZVPP basis set was chosen which is on the smaller side for CCSD(T) calculations so qualitative rather than fully quantitative results are expected.

For **1-Ph** a complete truncation of dispersive interactions seems unlikely and would lead to a C5 selective **TS-4**. But attenuating 60% of dispersion contribution which is closer to what Chen encountered in CH_2Cl_2 would very well better with the experimentally observed selectivity (Figure S25).

Also, in this case dispersive interactions seem to favor the C2 isomer, whereas unlike in the case of **1-Me** were the major driver for C5 selectivity seemed to be non-dispersive factors, here electronic factors seem to be more important for C5 selectivity. This appears rather independent of **TS-4** vs **TS-5** but more substrate dependent.

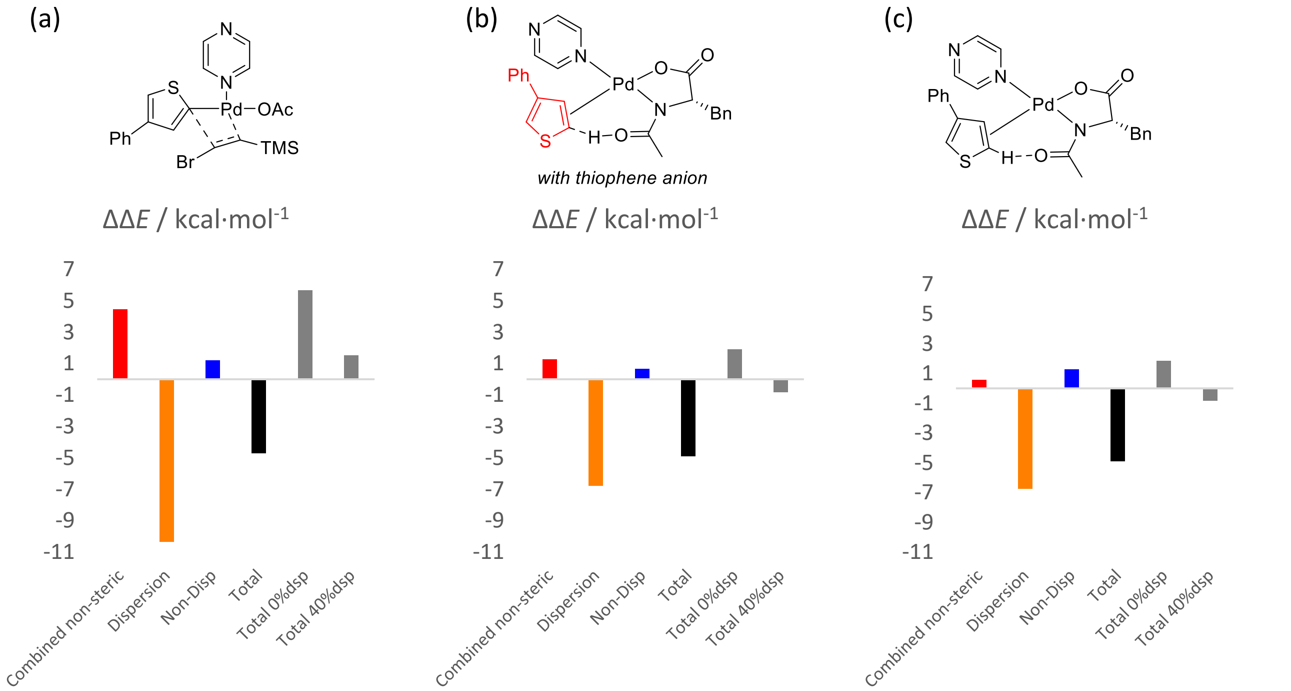

Figure S25: Comparison if the individual energy contributions of C2 vs C5 with combined non-steric contributions to better compare dispersion effects of (a) **TS-5-AcO** and (b) **TS-4-L2** for **1-Ph** anion and (c) **TS-4-L2** for **1-Ph**.

In Figure S26 there is an analogous comparison for **L16** and **TS-5-AcO**. The overall appearance for both **L2** and **L16** in **TS-4** is rather similar and only minor numerical differences are observed.

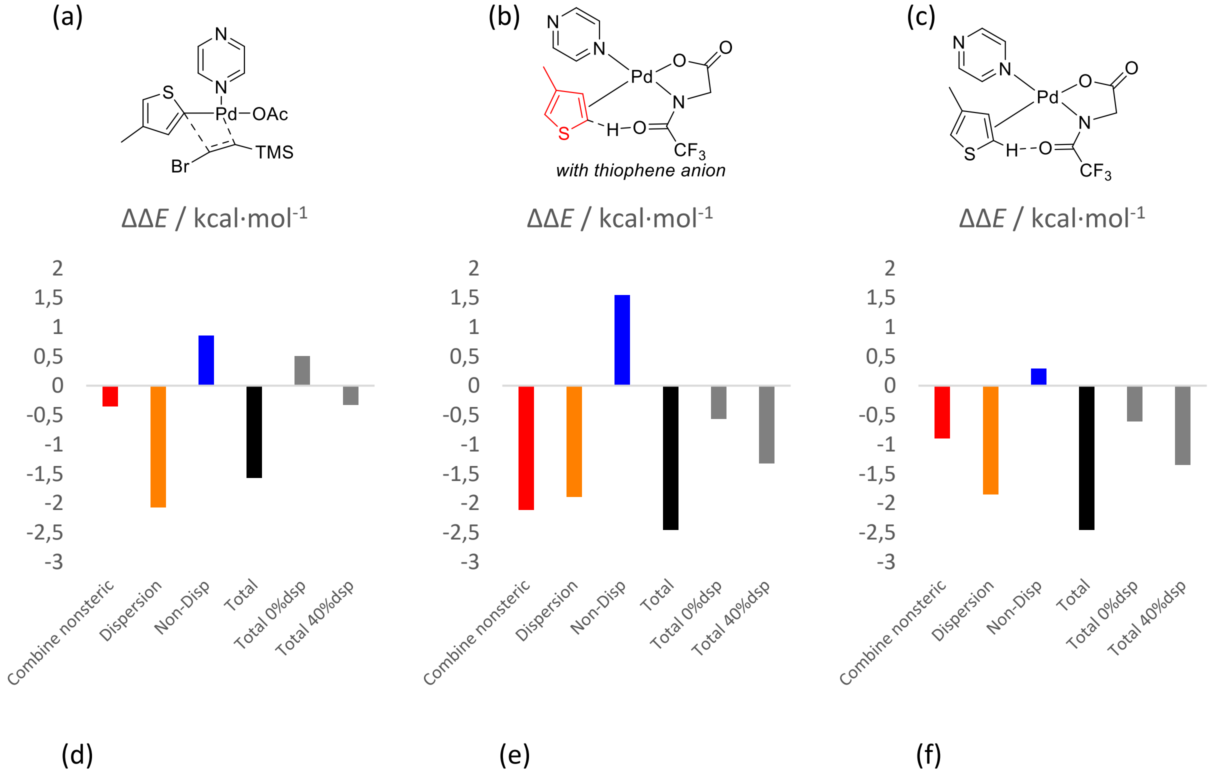

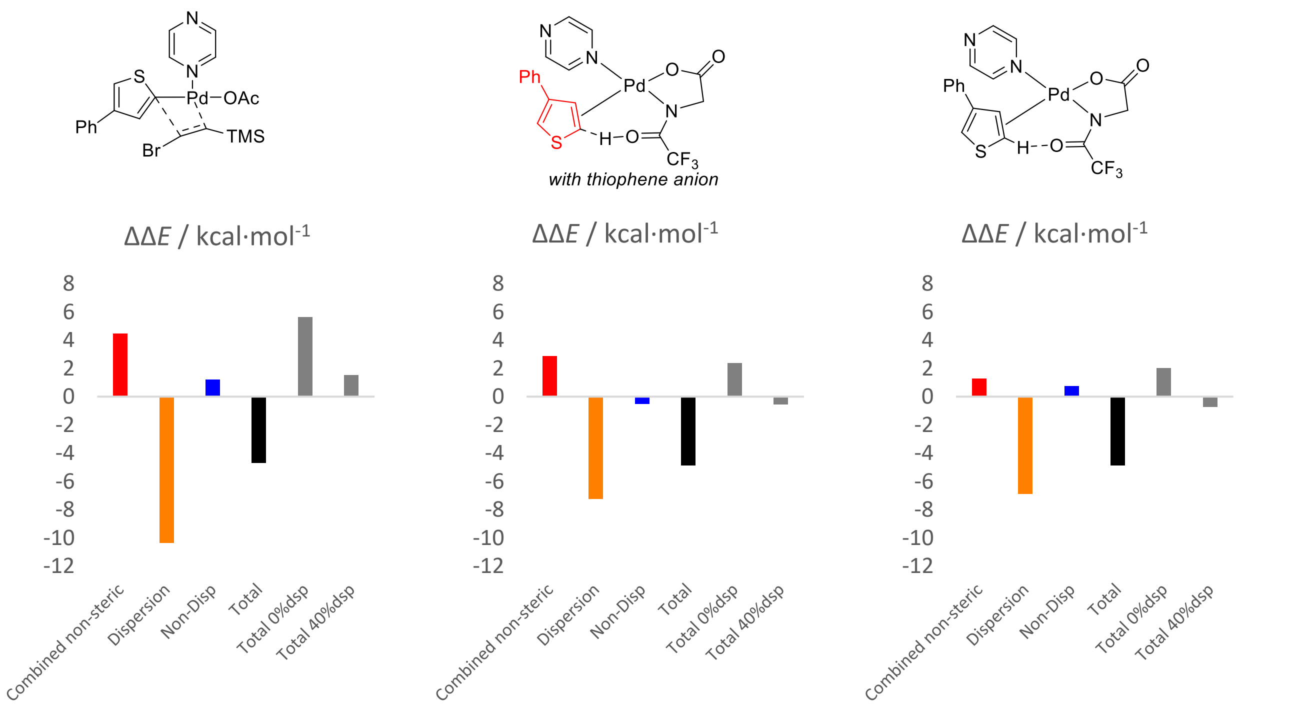

Figure S26: Comparison if the individual energy contributions of C2 vs C5 with combined non-steric contributions to better compare dispersion effects (a) **TS-5-AcO** for **1-Me** for comparison and (b) **TS-4-L16** for **1-Me** anion (c) **TS-4-L16** for **1-Me**. (d) **TS-5-AcO** for **1-Ph** for comparison and (e) **TS-4-L16** for **1-Ph** anion (f) **TS-4-L16** with **1-Ph**.

The dispersive interactions can also be visualized using LED maps (Figure S27). It becomes evident that during the C2 substitution the substituent in C3 position is pointed towards the catalyst and experiences dispersive interactions and hence stabilization. In case of C5 the C3 substituent is pointed outwards and would only interact with the solvent. Intuitively one would assume a methyl substituent to lead to a steric block rather than to an attractive interaction. We therefore assume that by disregarding solvent dispersion interactions C2 is artificially stabilized by overestimating intramolecular dispersion interactions.

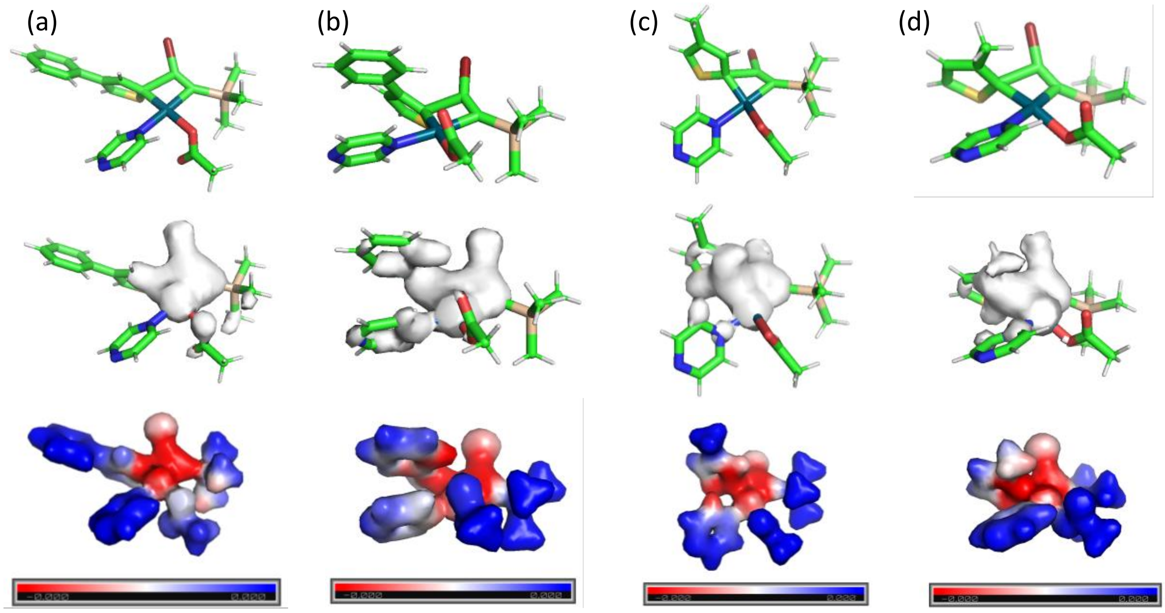

Figure S27: Plots with molecular structure, dispersion interaction density (DID) from LED analysis and DID mapped on electron density for (a) C5 **1-Ph** functionalization, (b) C2 **1-Ph** functionalization, (c) C5 **1-Me** functionalization, (d) C2 **1-Me** functionalization.

In case of the C–H activation of **1-Ph** a certain amount of dispersion interaction actually seems to be important since it contributes to the overall stabilization. Here especially the non-repulsive π-π interaction between pyrazine and phenyl rest is noteworthy (Figure S28).

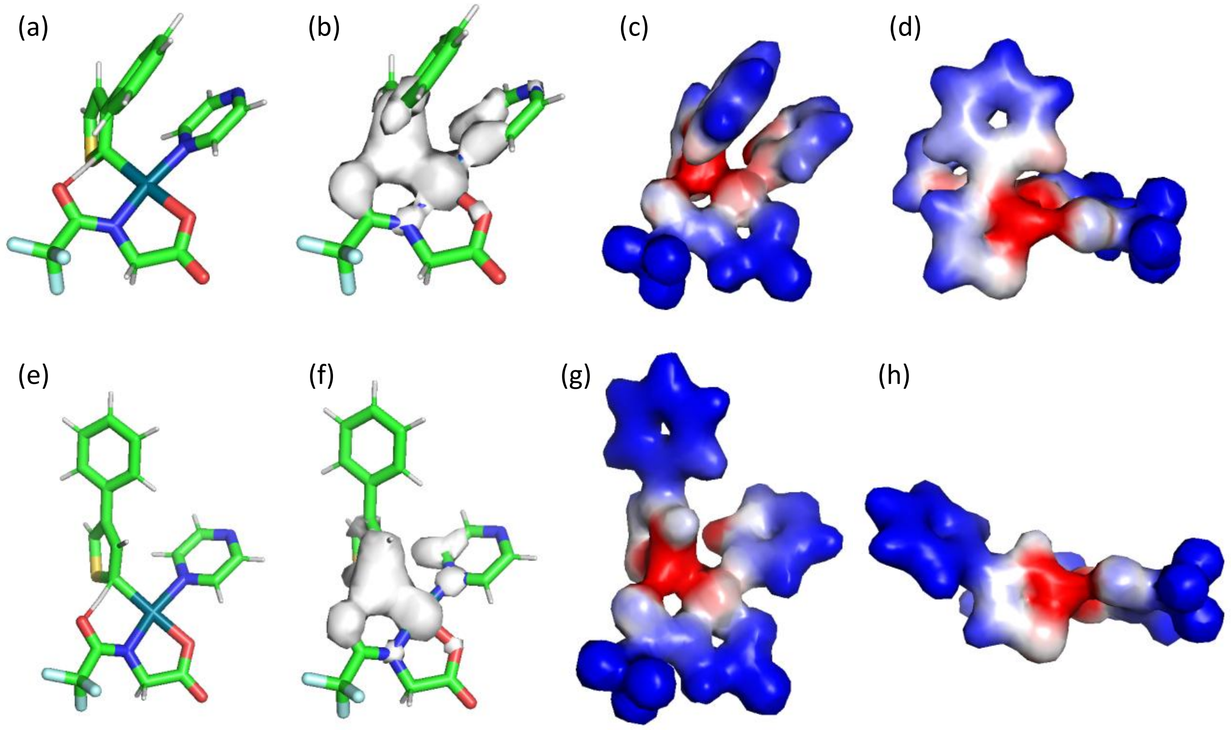

Figure S28: Plot with molecular structure for C–H activation of 3-phenylthiophene with **L16** for (a) C2 and (e) C5, DID plot for (b) C2 and (f) C5, DID mapped on the electron density for (c) C2 and (g) C5, turned plot to better visualize the DID effect on the phenyl ring for (d) C2 and (h) C5.

The trans orientation of thiophene to monodentate carboxylate ligand was found to be favorable to a cis orientation in the **TS-5-AcO** (see Figure S17). Since this is positioned afar from the thiophene C3 substituent, no additional dispersion interaction is expected. Indeed, previous results also suggest a preference of C2 over C5 using conventional D3BJ corrected DFT and a reversal of predicted selectivity upon switching off dispersion correction.

To investigate electronic effects, we carried out an additional LED analysis only focusing on the dispersion and combined other effects to see whether different electronics play a role in the selectivity since the N-acyl amino acid ligands likely possess slightly altered donating properties compared to acetate. For instance the experimental pK_A[90] values differ AcOH: 4.76, AcGly: 3.67, AcPhe: ~3.42 (*s*-trans)[91], TFA: 0.52, pivalic acid: 5.03), but the trends observed in Figure S29 show rather similar effects regardless of the ligand.

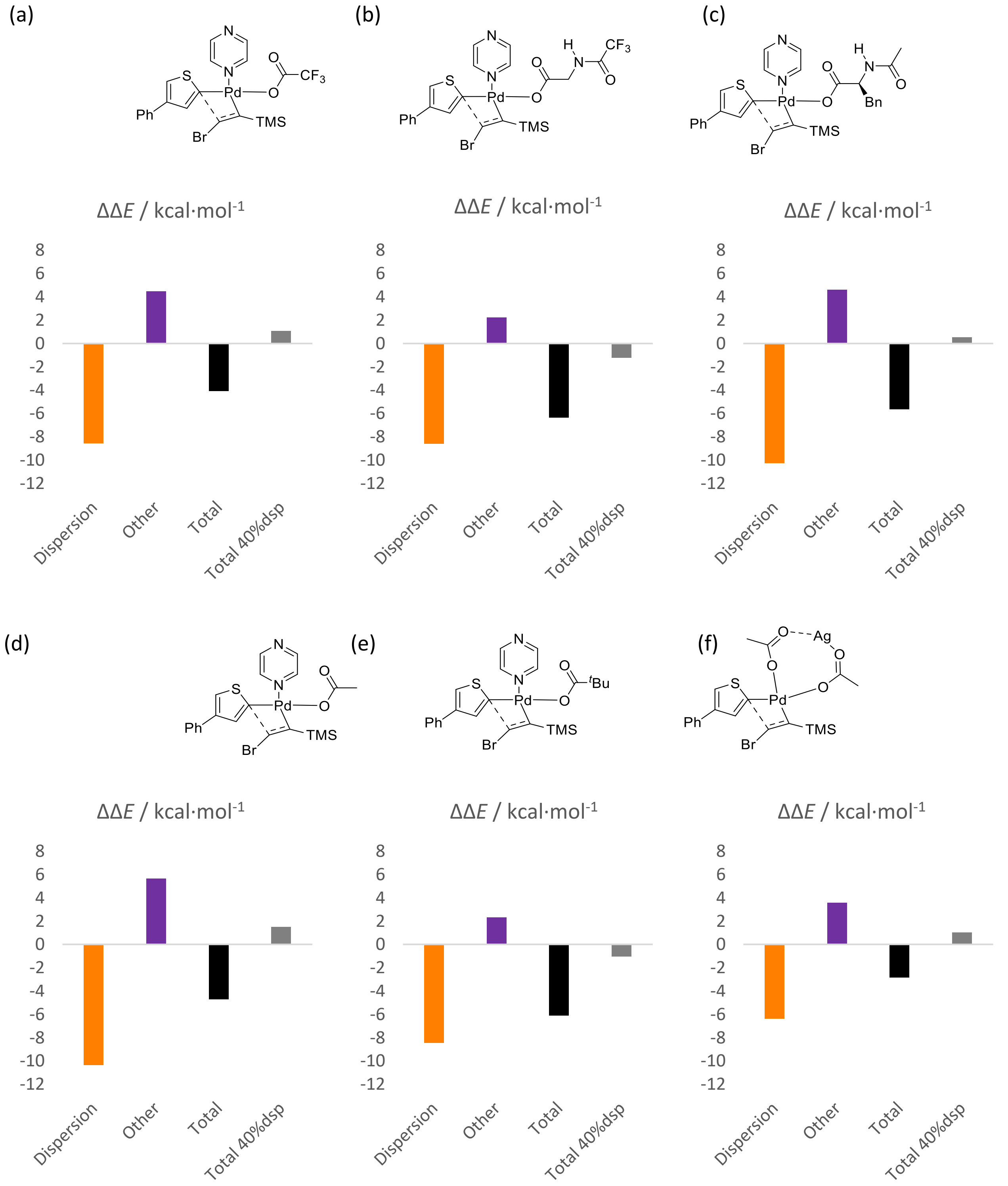

Figure S29: Comparison if the individual energy contributions of C2 vs C5 with combined non-steric contributions to better compare dispersion effects using different monodentate X-type ligands with different donating properties. (a) trifluoroacetate, (b) 2-(2,2,2-trifluoroacetamido)acetate, (c) (*S*)-2-acetamido-3-phenylpropanoate, (d) acetate, (e) pivalate, (f) silver acetate and acetate.

The same effect can also be observed using a silver containing TS. Of course, the precise numerical value for the extent of truncation of dispersion interaction to some extent depends on the ligand but the overall trends remain similar. It is nevertheless possible that the numerical value of preference for C5 selectivity is different depending on the precise ligand involved as evidenced in Figure S29 assuming only 40% of dispersion

interaction. For more precise numerical values, the remaining solvent effects would need to be considered and a larger basis set could be used, but this analysis already gives a rather representative qualitative picture.

Preliminary Studies on Explicit Solvation

To probe this further, several explicit solvent molecules were included in static DFT. **1-Ph** was chosen as the substrate since here the effect of dispersive interactions was shown to be more pronounced. The ORCA 6.0 solvator tool was used on the previously optimized geometries of **TS-5-AcO** and solvent molecules were added keeping the solute structure static. Only the preferred structure from ORCA 6.0 solvator was subjected to geometry optimization without additional conformational sampling. The constrained optimization (keep the bond distance between thiophen and alkyne at 2.05 Å) was carried out for C2 and C5 respectively. This was done as an alternative to TS optimizations to facilitate optimizing highly solvated structures. The constrained geometry optimization was done at the D3BJ TPSS/def2-SVP level for 0 to up to 15 EtOH molecules. The single point energy was then also evaluated at the CPCM(EtOH) ωB97X-D3BJ/def2-TZVP level of theory.

Adding a few explicit ethanol molecules, these were shown to exhibit specific hydrogen bond interactions but do not lead do an improved C2 vs C5 ratio. This is not fully unexpected since non-specific dispersive interactions of bulk solvents rather than specific interactions are expected to be required to fully adequately truncate the intramolecular dispersive interactions. It can be shown, e.g. for 15 solvent molecules that the phenyl group of the C5 thiophene is surrounded by enough EtOH molecules to adequately account for solvent-solute interaction. This solvent – solute interaction might be the reason for an overall more stable structure for C5 even with switched on dispersion correction. But since EtOH offers large conformational freedom with a major impact on the energy due to for example the number of hydrogen bonds, the results are to be taken with a lot of caution as evidenced by the rather large fluctuation in energies for the different number of EtOH molecules (and the different number of hydrogen bonds) and at times a reversal of the selectivity. To investigate the isolated dispersive effect more closely, we also conducted static calculations using methane. Here the “static” option in the ORCA solvator tool was used for 100 methane molecules around the substrates in droplet orientation. Afterwards a geometry minimization using a QM1/QM2 method with GFN2-xtb for the solvent and TPSS-D3(BJ)/def2-SVP for the solute was carried out and converged. The results at the TPSS-D3(BJ)/def2-SVP level also point towards a more stable C5 geometry. These results should be considered with some skepticism since even for methane conformational orientations can play a crucial role and since only a single structure is considered, a selectivity could also be wrongly assigned due to these artifacts.

Since especially the explicit static EtOH calculations proved to be challenging due to the conformational flexibility, we decided to probe this further using molecular dynamics that should adequately account for conformational freedom of the respective molecules.

Table S14: Addition of an increasing number of explicit ethanol molecules and the calculated $\Delta\Delta E^{\ddagger}$ / kcal·mol^{-1} at the CPCM(EtOH) ωB97X-D3BJ/def2-TZVP//TPSS-D3(BJ)/def2-SVP and the TPSS-D3(BJ)/def2-SVP level of theory.

# EtOH	0[a]	0	1	2	3	4	5	6	7	8	9	10	11	12	13	14	15	100 methane
ωB97X-D3BJ	-3.4	-3.8	-4.0	-1.6	-7.7	-7.8	-6.0	3.14						6.7		6.9	2.0	13.6**
TPSS-D3(BJ)	-6.6	-6.1	-6.5	-4.0	-8.0	-4.9	-0.6	11		14.1	11.6	14.3	-4.8	9.5	4.7	31.0	12.5	7.1 (15.4, 11.2[d])[c]
#HB C2	0	0	1	2	3	3	4	3		7	7	6	9	8	10	10	12	-
#HB C5	0	0	1	2	2	3	3	5	6	8	8	8	9	9	11	13	12	-

[a] with TS optimization and not via constrain,[b] with CPCM(hexane), [c] values in parenthesis at the QM1/QM2 level, [d] not fully converged, value after 200 optimization steps.

Some preliminary molecular dynamics simulations using ONIOM (QM1/QM2) were therefore conducted. Using the semiempirical GNF2-xtb[92] method for solvents and TPSS-D3(BJ)/def2-SVP for the migratory insertion. This is aimed to be a very approximate and qualitative rather than quantitative study. The C2 and C5 TS were placed in a sphere of 75 EtOH molecules respectively using Packmol.[93] The solvent sphere was constrained to 12 Å to roughly account for the solvent density. The distance between thiophene and alkyne was constrained to 2.05 Å since a TS is to be modelled. Both orientations of the acetate group were probed and several runs were carried out with random initial velocities. The Berendsen thermostat at 313K with otherwise default settings was used since this led to stable, non-fluctuating energies. Despite this not rigorously describing a canonical NVT ensemble, the errors introduced by fluctuations due to incomplete equilibration, etc. with the available computational resources likely result in smaller errors compared to using the Nosé-Hoover or a comparable thermostat. Timesteps of 0.5 fs were used. Aborted calculations were continued using the "Restart if available" keywords. The same outlined procedure was repeated in the absence of solvent using only TPSS-D3(BJ)/def2-SVP. In the non-solvated case 10 ps were sampled and the average total energy from 5-10 ps was averaged. This was repeated for both conformers of C2 and C5 ten times respectively. The lowest conformer results were considered and an average of -6.4 kcal/mol in favor of C2 was found. In the solvated case, the more stable conformer was used and an average of 5 runs was considered with a total time of 30 ps and sampling from 20-30 ps (Table S15). Different intervals for example with longer equilibration and shorter production time for the same simulations were tried but no qualitative difference was noted.

Table S15: QM1/QM2 MD runs for solvated and unsolvated **TS-5-AcO** with **1-Ph** using QM1=GFN2-xtb and QM2= TPSS-D3(BJ)/def2-SVP. With exemplary MD traces and times for the production run highlighted in grey.

**No solvent #Run**	**C2_Conf01 / Eh**	**C2_Conf02 / Eh**	**C5_Conf01 / Eh**	**C5_Conf02 / Eh**	**C2 vs C5 / kcal/mol**
1	-4462.5776	-4462.5722	-4462.5729	-4462.5592	
2	-4462.5771	-4462.5727	-4462.5674	-4462.5746	
3	-4462.5874	-4462.5898	-4462.5837	-4462.5765	
4	-4462.5822	-4462.5832	-4462.5760	-4462.5708	
5	-4462.5775	-4462.5813	-4462.5736	-4462.5704	
6	-4462.5845	-4462.5731	-4462.5735	-4462.5697	
7	-4462.5852	-4462.5714	-4462.5710	-4462.5739	
8	-4462.5856	-4462.5731	-4462.5690	-4462.5749	
9	-4462.5869	-4462.5731	-4462.5685	-4462.5752	
10	-4462.5826	-4462.5737	-4462.5697	-4462.5583	
**Average**	-4462.5827	-4462.5764	-4462.5725	-4462.5703	**-6.4**
**75 EtOH #Run**	**C2_Conf01 / Eh**		**C5_Conf01 / Eh**		**C2 vs C5 / kcal/mol**
1	-5316.1584		-5316.1681		
2	-5316.1631		-5316.1766		
3	-5316.1874		-5316.1915		
4	-5316.1790		-5316.1825		
5	-5316.1813		-5316.1826		
**Average**	**-5316.1739**		**-5316.1803**		**4.0**

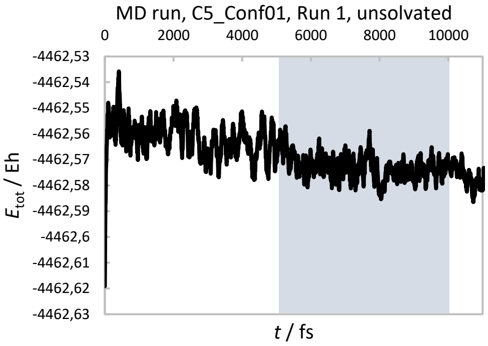

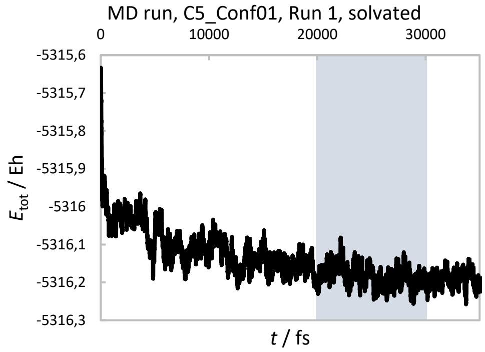

We indeed observed a reversal of the selectivity from the solvated to the unsolvated case. Please note that higher level DFT methods (or machine learned force fields), periodic boundary conditions, and especially longer simulation times to fully converge the simulation would be required for more quantitative results but are prohibitively costly for our set up and outside the scope of this study. These preliminary results nevertheless strengthen our working hypothesis especially considering: a) computationally no other energetically feasible structure was in line with the experimentally observed C5 selectivity. Only a few structures like some OA TS show in some cases a C5 preference but are predicted to be ~10 kcal/mol higher than alternate TS. b) the current hypothesis aligns well with the mechanistic observations in section 4, c) reference literature that describes an attenuation of dispersion interactions not accounted for by implicit solvation models.

3.3 Proposed Simplified Catalytic Cycle

Combining the above experimental results, it is possible to draft the following simplified catalytic cycle (Figure S30). After formation of the resting state (**Int-0** or **$PdL1_2(OAc)_2$** depending on the ligand) one molecule of ligand **L1** de-coordinates with an intermediate coordination of EtOH and then replacement by thiophene. **1-Me** undergoes C–H activation. In case of **L16** this step is turnover limiting and selectivity determining. After ligand exchange, the alkyne coordinates and undergoes MI. In case of **L2** this step is turnover limiting and selectivity determining assuming a reversibility of **TS-4** The MI is followed by a pyrazine coordination and a bromide elimination. The original catalyst can be regenerated by stripping silver.

Several things should be noted: for **TS-4** and **TS-5** (depending on the solvation model), TSs involving silver have barriers only slightly higher than the respective pyrazine analogs. It is crucial to note that an involvement can

therefore not fully be ruled out, but it was computationally not found necessary for catalytic turnover. The role of silver is discussed in detail in section 3.4. Additionally, either acetate, after ligand exchange, or the monodentate carboxylate of the respective ligand after the C–H activation can act as a monodentate anionic ligand in the subsequent steps. Due to the very similar acidity and hence donating ability, it is rather difficult to exclude one or the other scenario assuming a fast ligand exchange. We do not expect any of the conclusions to change due to the nature of this ligand as suggested by the Pd source replacement experiment in Figure S78. Additionally, the inverse migratory insertion was excluded due to higher predicted barriers with TIPS (Figure S17) and the ^{13}C KIE studies.

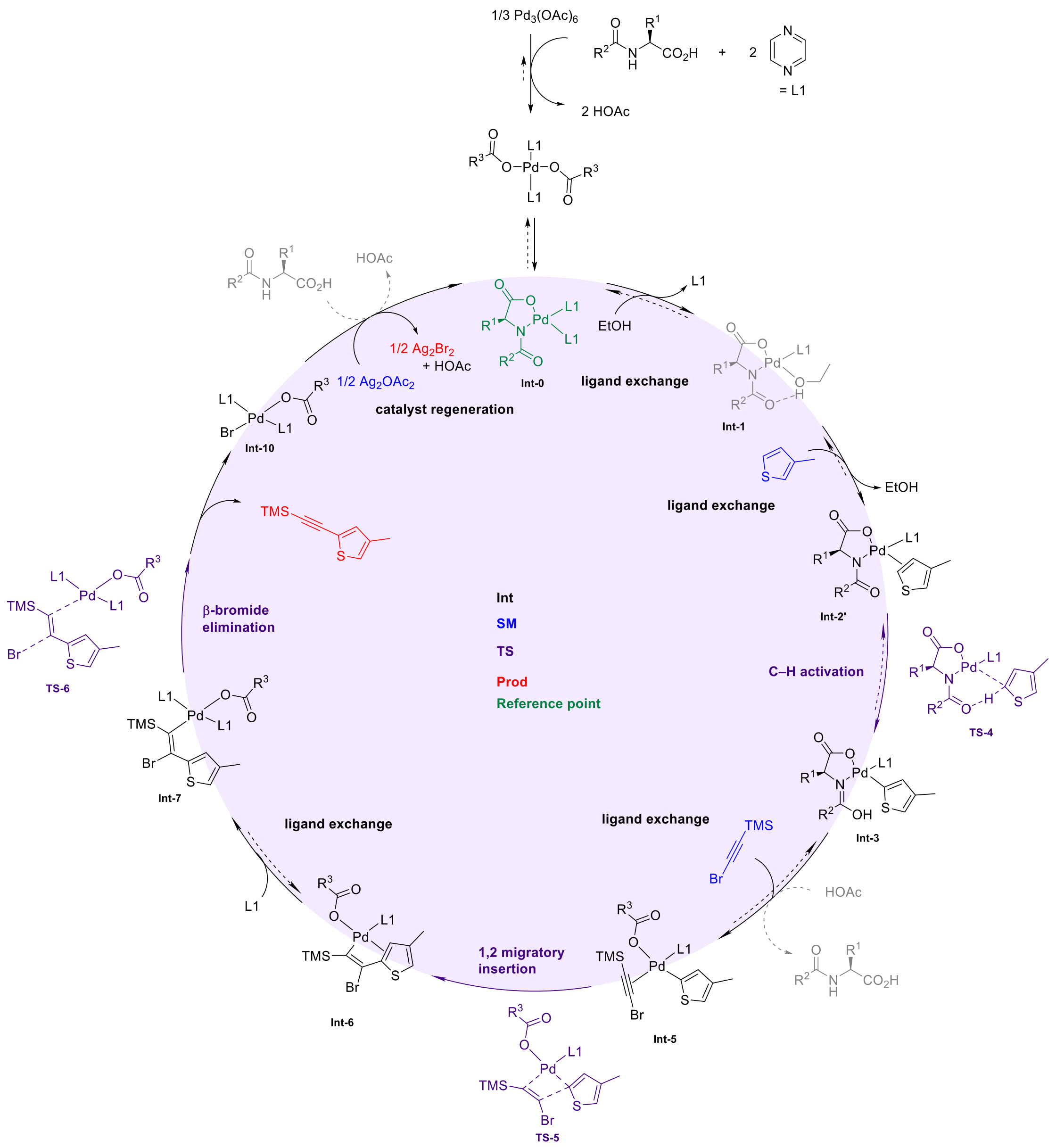

Figure S30: Proposed catalytic cycle for the C5 and C2 selective thiophene alkynylation.

L16 and **L2** are chosen to compare the overall reaction energies. Several factors will make an accurate prediction of the relative barriers of **TS-4** and **TS-5** difficult: the relative barriers between C2 and C5 for **TS-4** and especially for **TS-5** are prone to error as discussed extensively in section 3.2. Hence C5 would likely be lowered due to beneficial solute-solvent interactions compared to C2. Additionally, errors in the DFT

functional lead to errors in the relative barriers. This can be prohibitively large since even good performing functionals show MADs of up to 1-2 kcal/mol in classical DFT benchmarks.[46] The relative error here is of course estimated to be lower since a certain amount of error cancellation can be assumed for conformer ranking and relative barriers that have similar types of ligands. Additionally, the implicit solvation model will have a major influence due to the rather different nature of **TS-4** and **TS-5**. Especially in **TS-5** considering a coordination of N-acyl amino acid ligand poses a potential risk, since here many polar groups are present that can in addition to conformational freedom interact with the solvent (H-bond, etc.). Even for performant models like COSMO-RS the MAE is considered to be at least 1-2 kcal/mol (2-3 kcal/mol for large systems).[74] Therefore, the results should be considered carefully since they are subject to a non-negligible error. The goal is hence not to accurately predict the selectivities using classical DFT but rather to show that the barriers of **TS-4** and **TS-5** are in a similar energetic range and thus the proposed mechanism is not unreasonable.

Several functionals and solvent models were chosen for the energy evaluation as well as two functionals for the geometry optimization (Figure S19). The difference of functional for the geometry optimization and thermochemistry calculation for the absolute barriers are rather small (max ~0.5-2 kcal/mol) and relative trends are conserved. For **TS-4** C2 no TS was initially located since PBE0-D3(BJ)/def2-TZVP did not result negative frequencies for the TS regardless of the C–H bond length. Different grid settings were also unsuccessful so we conducted a relaxed surface scan along the C–H bond in small steps and considered the maximum as the TS. ωB97X-D3BJ did not suffer from the same problem. As discussed before, the main differences lie within the relative barriers for **TS-5**. More drastic effects are observed for the solvent model: **TS-4** is slightly increased in energy by switching from CPCM or dCOSMORS to SMD. **TS-5** on the other hand is slightly decreased by switching from CPCM to SMD. Switching from CPCM to dCOSMORS leads to a large increase in energy for **TS-5** especially in the silver mediated case.

Table S16: Functional and solvation model comparison for the relevant steps in the switch of selectivity determining step. Higher lying and hence selectivity determining step for C2 marked in red, competing, lower step marked in green.

	Reference: Int-0-L16							
**Method**	**TS-4-L16 C2**	C5	**TS-5-AcO C2**	C5	**TS-5-L16 C2**	C5	**TS-5-AgOAc C2**	C5
CPCM(EtOH) PWPB95-D3(BJ)/def2-QZVPP//ωB97X-D3BJ/def2-TZVP	**18.9**	21.4	20.4	19.9	**13.9**	16.4	18.8	20.6
SMD(EtOH) PWPB95-D3(BJ)/def2-QZVPP//ωB97X-D3BJ/def2-TZVP	**20.4**	22.9	20.2	19.4	**13.7**	16.2	18.5	20.3
CPCM(EtOH) ωB97X-D3BJ/def2-TZVP //ωB97X-D3BJ/def2-TZVP	**20.8**	23.8	20.7	20.6	**14.9**	16.7	17.0	18.5
SMD(EtOH) ωB97X-D3BJ/def2-TZVP //ωB97X-D3BJ/def2-TZVP	**22.3**	25.3	20.6	20.2	**14.7**	16.6	16.8	18.4
dCOSMORS ωB97X-D3BJ/def2-QZVPP //ωB97X-D3BJ/def2-TZVP	**21.6**	24.5	25.8	25.3	**21.1**	22.9	27.8	29.2
dCOSMORS MN15/def2-QZVPP //ωB97X-D3BJ/def2-TZVP	**21.1**	23.9	25.4	24.6	**20.7**	22.6	26.0	28.1
CPCM(EtOH) PWPB95-D3(BJ)/def2-QZVPP//PBE0-D3(BJ)/def2-TZVP	**18.6**	21.0	20.2	21.6	**13.3**	14.9	18.2	20.1
dCOSMORS CCSD(T)/def2-QZVPP//PBE0-D3(BJ)/def2-TZVP	**21.5**	24.0	25.1	26.5	**20.0**	20.9	26.5	27.8

dCOSMORS CCSD(T)/CBS//PBE0-D3(BJ)/def2-TZVP	**21.9**	24.2	25.8	27.2	**20.7**	21.4	27.1	28.3
dCOSMORS ωB97X-D3BJ/def2-TZVPP // PBE0-D3(BJ)/def2-TZVP	**21.4(4)**	24.3	24.2	25.9	**21.3(5)**	22.2	27.8	29.1

Reference: Int-0-L2

Method	TS-4-L2 C2	C5	TS-5-AcO C2	C5	TS-5-L2 C2	C5	TS-5-AgOAc C2	C5
CPCM(EtOH) PWPB95-D3(BJ)/def2-QZVPP//ωB97X-D3BJ/def2-TZVP	**14.8**	17.0	20.1	19.2	**16.5**	17.8	18.5	20.3
SMD(EtOH) PWPB95-D3(BJ)/def2-QZVPP//ωB97X-D3BJ/def2-TZVP	**16.1**	18.4	19.3	18.5	**15.6**	17.5	17.6	19.4
CPCM(EtOH) wB97X-D3BJ/def2-TZVP //ωB97X-D3BJ/def2-TZVP	**16.6**	19.3	20.2	20.0	**17.1**	18.1	16.4	18.0
SMD(EtOH) wB97X-D3BJ/def2-TZVP //wB97X-D3BJ/def2-TZVP	**18.0**	20.8	19.6	19.1	**16.2**	18.0	15.7	17.4
dCOSMORS wB97X-D3BJ/def2-QZVPP //ωB97X-D3BJ/def2-TZVP	**19.8**	22.0	25.1	24.5	**23.2**	23.8	27.0	28.5
dCOSMORS MN15/def2-QZVPP //ωB97X-D3BJ/def2-TZVP	**18.5**	21.0	23.7	22.9	**23.1**	23.9	24.3	26.4
CPCM(EtOH) PWPB95-D3(BJ)/def2-QZVPP//PBE0-D3(BJ)/def2-TZVP	**13.6***	17.4	19.1	20.5	**15.7**	17.0	17.1	19.0
dCOSMORS CCSD(T)/def2-QZVPP//PBE0-D3(BJ)/def2-TZVP	**17.2***	21.2	21.8	23.2	**21.2**	21.7	23.2	24.5
dCOSMORS CCSD(T)/CBS//PBE0-D3(BJ)/def2-TZVP	**17.4***	21.1	21.7	23.1	**21.3**	21.7	22.9	24.1
dCOSMORS ωB97X-D3BJ/def2-TZVPP // PBE0-D3(BJ)/def2-TZVP	**17.8***	22.6	24.2	25.9	**22.8**	23.5	26.0	27.2

* A constrained surface scan was conducted since PBE0 did not converge to a TS. The maximum on this PES was considered for the TS.

It can hence be shown that all functionals employed here in combination with CPCM or dCOSMORS at least qualitatively verify the proposed hypothesis. COSMORS has often been benchmarked to be a superior model. SMD is often termed more complete and often outperforms CPCM but this is highly system dependent and reactions with a better performance of CPCM are also known.[31,35,94] These results suggest that a switch in selectivity determining step might be feasible. Especially coupled cluster results, in combination with the physically sound dCOSMORS model and the good benchmarked PBE0-D3(BJ)/def2-TZVP functional point towards the proposed pathway.

Please note: We concluded before that the calculated relative barriers for TS should rather be $\Delta\Delta G^{\ddagger}$(**TS-4** C2 vs C5) ~ -1 kcal/mol and $\Delta\Delta G^{\ddagger}$(**TS-5** C2 vs C5) ~ 1 kcal/mol. Assuming C2 is correctly calculated, C5 should be ~+1kcal/mol relative to **TS-4** C2 and ~-1 kcal/mol to **TS-5** C2.

The entire pathway from **Int-0** until the MI is visualized in Figure S31 for C2. The values using dCOSMORS(EtOH) ωB97X-D3BJ/def2-QZVPP//ωB97X-D3BJ/def2-TZVP are shown since this combination gives qualitatively a good agreement for the dCOSMORS CCSD(T) results and is computationally viable.

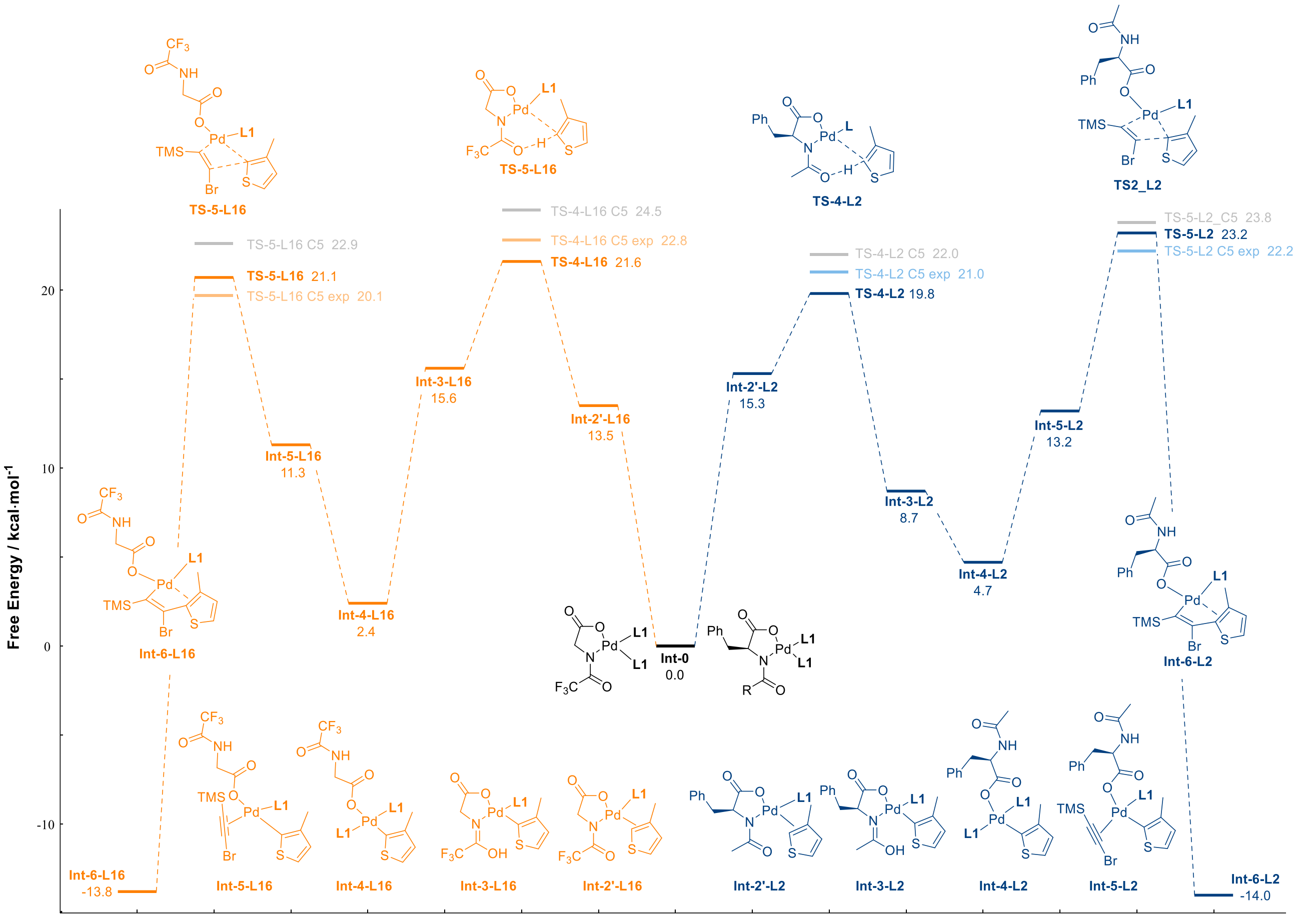

Figure S31: Comparison of the absolute barriers for the C-H activation sequence and MI for **L16** and **L2** at dCOSMORS(EtOH) ωB97X-D3BJ/def2-QZVPP//ωB97X-D3BJ/def2-TZVP level of theory. *Exp* refers to the experimental $\Delta\Delta G^{\ddagger}$ values for C5 which are referenced relative to the calculated absolute barriers for C2.

The MI is then followed by a ligand exchange and a 1,2-bromide elimination. Different possibilities for the elimination step are discussed in Figure S36. In Figure S32 the remaining simplified steps for the C2 product are displayed. Please note that coordination of N-acyl amino acid ligand is also possible, but is not shown for simplicity's sake.

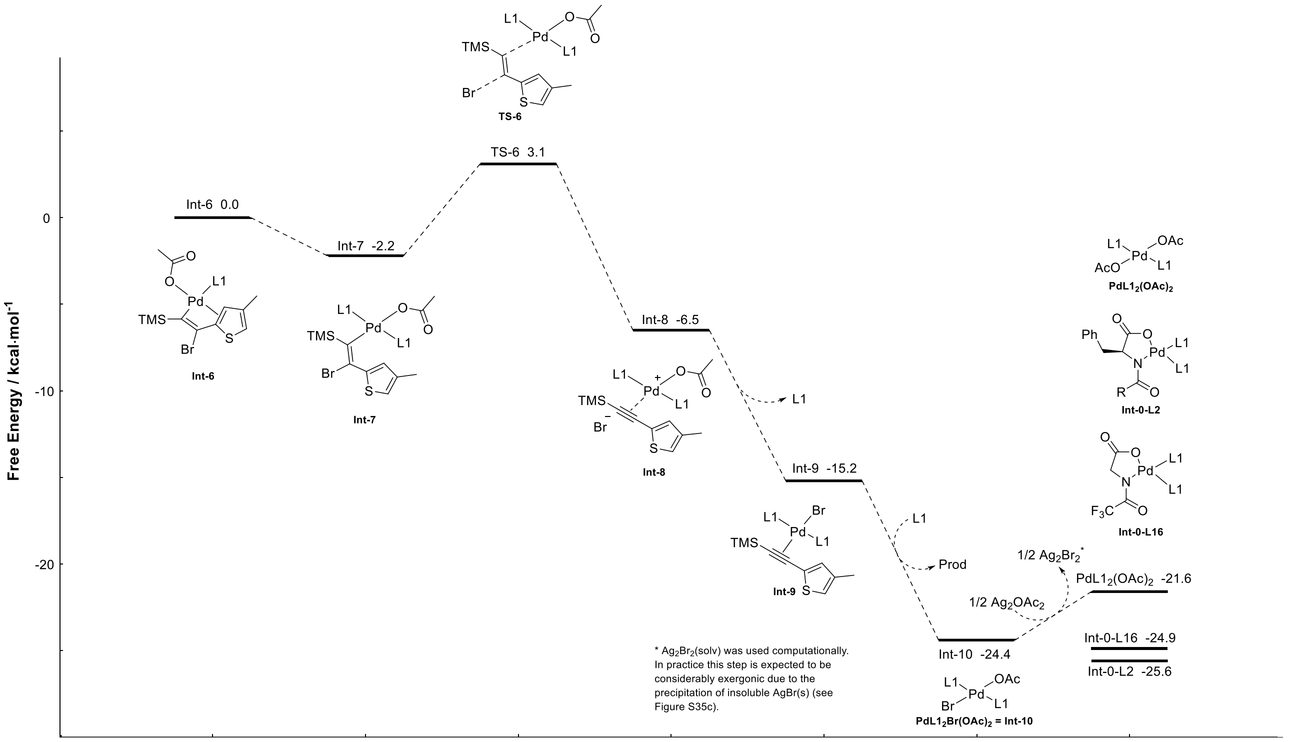

Figure S32: Final steps after the MI insertion calculated at the dCOSMORS(EtOH) ωB97X-D3BJ/def2-QZVPP//ωB97X-D3BJ/def2-TZVP level of theory.

As a first approximation, using k_{obs} from the initial rates obtained in the parallel KIE studies (Table S18) and using the Eyring equation at 313K, one obtains ΔG_{obs} = 24.9 and 23.4 kcal/mol for **L2** C2 and C5 respectively and ΔG_{obs} = 23.8 and 24.6 kcal/mol for **L16** C2 and C5. Due to the difficulty regarding modelling solvation in the MI step and an assumed error of at least 1 kcal/mol even for CCSD(T) calculations as well as the errors of solvation models, the computational results are in line with the experimental observables.

An unusual inverse solvent kinetic isotope effect led us to investigate steps with solvent involvement further. A hypothesis was derived that coordination of ethanol to the catalyst and subsequent de-coordination could be the reason for the inverse isotope effect. Therefore, the steps regarding substrate coordination starting from **Int-0** were investigated in more detail (Figure S33). Ligand exchange from the resting state could occur via a dissociative pathway where pyrazine de-coordinates and the amide oxygen of the N-acyl amino acid ligands binds to Pd instead. This pathway is comparatively high in energy and an associative pathway was investigated. Direct association of thiophene and replacement of pyrazine was indeed lower in energy compared to the dissociative pathway. The lowest energy pathway was found to proceed via the initial coordination of ethanol to replace pyrazine in **Int-1**. Afterwards a replacement of ethanol by thiophene can take place. Here different coordination modes were probed and it was found that the pathway proceeding via sulfur coordination is lowest in energy. The π-coordination is higher in energy, which rules out the advent of a selectivity determining step in the substrate coordination since the π-coordination could in principle have yielded **Int-2'** in a C2- or C5-preferential position. Interestingly, the thiophene S-coordination **TS-2** appears to be slightly higher in energy than C2 selective C–H activation **TS-4**. A high barrier is in line with the experimental observation for the difference in inverse KIE for C2 and C5 (*vide infra*).

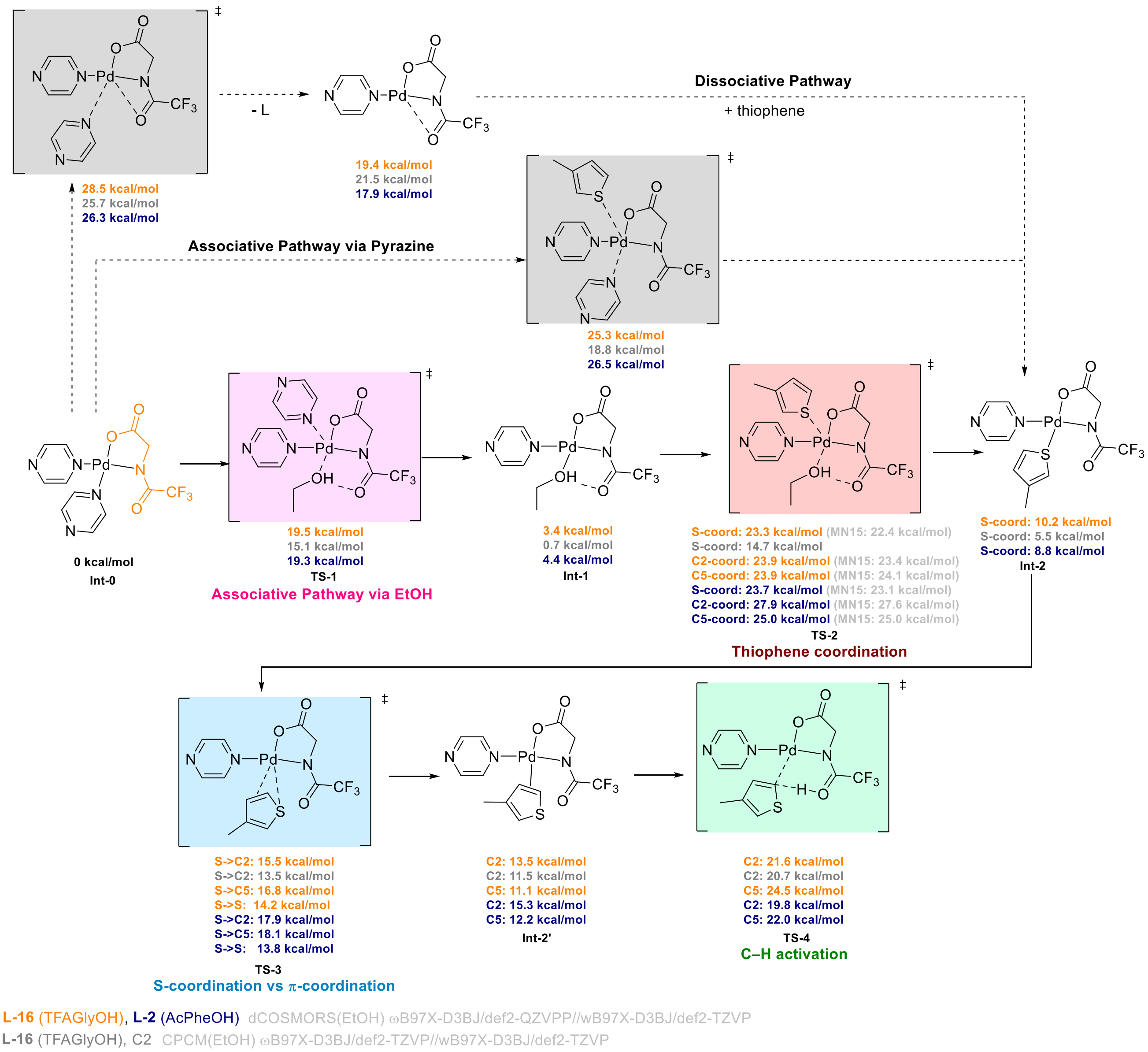

Figure S33: Possibilities for coordination of thiophene prior to the C–H activation. Calculated with **L-2** and **L-16**.

The S-coordinated thiophene can rearrange via **TS-3** while remaining coordinated to Pd. This delivers a π-coordination in **Int-2'** required for the subsequent C–H activation in C2 or C5 position. **Int-2'**-C2 can subsequently react via **TS-4**-C2 to ultimately yield C2 product and the isomer **Int-2'**-C5 reacts to form C5 product via **TS-4**-C5. The interconversion of π-coordinated **Int-2'**-C2 to **Int-2'**-C5 via was found to be energetically facile.

Accordingly, these findings do not alter conclusions regarding the Curtin-Hammett like scenario for the observed ligand dependent selectivity difference, but include one additional high energy transition state that is relevant for product formation.

We want to underline a certain error margin for the relative barrier heights. The large concentration differences of solvent are accounted for in our calculations (*vide supra*), but different concentrations of Pd, substrate, and ligand are not considered, a standard concentration being assumed. Additionally, entropy effects are often poorly estimated for solvent exchange reactions using static DFT and are often modelled

using MD.[95] Furthermore, errors caused by the solvation model of 2-3 kcal/mol even for COSMO-RS can contribute to the overall error. Calculations carried out with CPCM (Figure S33 grey values) show very similar barrier heights for the C–H activation step and also show good agreement for the C2 vs C5 selectivity switch (vide supra, Figure S18) but show lower barriers here for the preceding ligand exchange steps. The higher barrier for ligand and substrate coordination without explicit EtOH is also observed with CPCM. The CPCM results are further corroborated to by SMD results. Overall, these solvation models identify the C–H activation or MI rather than the substrate coordination as turn over limiting step, which is in line with the experimentally derived expectations. Overall, it is not surprising that implicit solvation models can perform differently well for different steps.

We expect the results to be purely qualitative as we have shown that especially steps with explicit EtOH might be subjected to some error. Also due to the solvent attenuation a quantitative comparison of the C2 vs C5 path for one ligand is not feasible since we expect similar errors on other intermediates. We have shown (*vide supra*) that the attenuation of dispersion interaction for e.g. C2 is similar in magnitude for the C–H activation and MI step. One can therefore assume that it is valid to compare the subsequent path with direct C2 coordination due to a similar geometric environment leading to similar effects. Please also note that the effect using 1-Me is much less pronounced than for 1-Ph and therefore qualitative conclusions can reliably be drawn.

Calculation of KIEs for Key Structures

To compare with experiment kinetic isotope effects (KIE) were calculated for several structures. The hessian files from the geometry optimization were used as an input for PyQuiver[96] to obtain corrected values for the KIEs. The temperature was set to the experimental 313K and a correction factor for the harmonic frequencies as specified in the literature[97] was used. Different tunneling correction schemes like proposed by Wigner[98] or inverted parabola by Truhlar[99] are implemented and the inverted parabole results are discussed below unless otherwise specified. They possess a similar magnitude for all secondary KIEs. Please note that especially for primary $^{1}H/^{2}H$ KIEs the magnitude of the KIE can be heavily affected by tunneling which is often only approximately accounted for by the respective corrections and alternate approaches like variational transition state theory are preferred.[100]

Several isotope effects were measured and will be discussed. A larger KIE for **L16** compared to **L2** is observed. A primary KIE for a C–H bond cleavage in a CMD is usually rather large. The values observed for **L16**, however, are around 2. This is in line with a reversible C–H activation and an additional high-energy step with a KIE of 1. In one extreme case, where the C–H activation is turnover-limiting and irreversible, one would expect a KIE close to the theoretical value (Figure S34a). In the other extreme, where the follow-up or previous step is turnover limiting and the C–H activation low in energy, one would expect a KIE of one. In an intermediate scenario one can approximate that the higher in energy the respective other step, the lower the observed KIE and vice versa.[48,101] We interpret a smaller KIE for **L2** as indicative of a lower lying C–H activation step compared to **L16**. Either the ligand exchange or the migratory insertion could be higher in energy. The computationally estimated KIEs for the isolated C–H activation step (**Int-2’** -> **TS-4**) are well above 3 and correspond to values typically encountered for primary KIEs in turnover limiting C–H activation steps. The precise values should not be overinterpreted due to very rough approximation of tunneling effects. Information on the experimental measurements can be found in section 4.2.

The $^{12}C/^{13}C$ KIEs were used to discriminate between a potential normal MI and an inverse MI followed by a silyl group transfer. Values around unity for C_B (Figure S34b) and larger values for C_A are predicted, which fits the expectations for a normal MI but is exactly opposite to the expectation for an inverse migratory insertion. This was also probed using TIPS instead of TMS and similar results were obtained for the normal MI. A potential oxidative addition would also display values in a similar range as the normal migratory insertion but with larger values on C_B. Please note that a discrimination between OA and MI solely based on the KIE data is not reliable within the respective experimental errors. The oxidative addition was computationally ruled out before (*vide supra*). Experimentally the KIE study was performed with an excess in thiophene and using **L2** and is described in more detail in section 0.

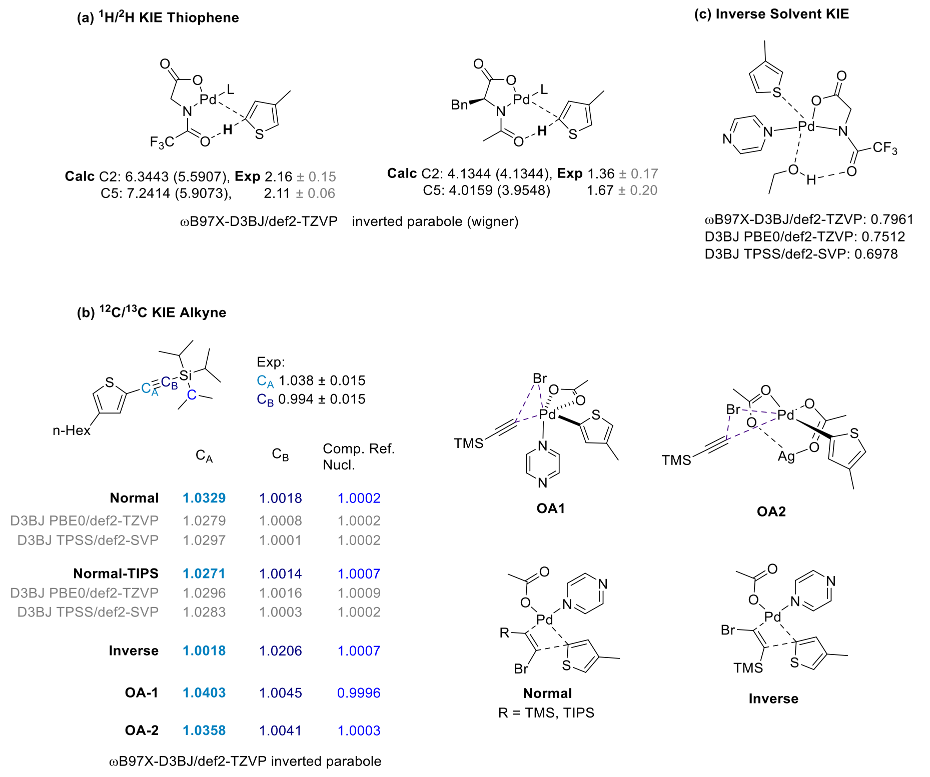

	C_A	C_B	Comp. Ref. Nucl.
**Normal**	1.0329	1.0018	1.0002
D3BJ PBE0/def2-TZVP	1.0279	1.0008	1.0002
D3BJ TPSS/def2-SVP	1.0297	1.0001	1.0002
**Normal-TIPS**	1.0271	1.0014	1.0007
D3BJ PBE0/def2-TZVP	1.0296	1.0016	1.0009
D3BJ TPSS/def2-SVP	1.0283	1.0003	1.0002
**Inverse**	1.0018	1.0206	1.0007
**OA-1**	1.0403	1.0045	0.9996
**OA-2**	1.0358	1.0041	1.0003

ωB97X-D3BJ/def2-TZVP inverted parabole

Figure S34: (a) Estimation of the theoretical KIE for the C–H activation step. (b) Estimation of the $^{12}C/^{13}C$ KIE for the normal and inverse MI and an OA. (c) EtOH coordination replacement by thiophene as a potential source for the inverse solvent isotope effect.

An additional observation is an inverse solvent isotope effect. Several scenarios have been suggested for inverse solvent isotope effects in the context of enzyme catalysis.[102] A potentially relevant scenario is the coordination of the solvent to the metal center in an equilibrium preceding the rate limiting step. The solvent isotope effect would therefore arise from an equilibrium isotope effect (EIE) rather than a kinetic isotope effect in the rate determining step. Ethanol could potentially be coordinated in an intermediate prior to the turnover limiting step via a O–Pd to the metal center and via a strong hydrogen bond to the ligand (H–OC). Further diffuse effects (e.g. solvent viscosity), restriction of ground- or excited state vibrational modes, erroneous results due to vastly different water contents that slow down the EtOH reference reaction could in principle also be responsible for the observed solvent isotope effect but are less likely. A similar catalytic system for the deuteration of arenes was found to undergo ligand exchange via de-coordination of monodentate ligand, an intermediate coordination of the amide oxygen, and subsequent replacement by the respective arene.[48] Here, it was found that the analogous intermediate is rather high in energy, almost

comparable to the subsequent C–H activation step. It is therefore not unreasonable to assume that de-coordination of pyrazine is assisted by the protic solvent, here ethanol. The coordination of ethanol was found to be energetically viable, but less stable than the originally proposed intermediate. The equilibrium isotope effect of the ethanol de-coordination – thiophene coordination is predicted to be ~0.7 which would be in a range comparable with the experimentally found isotope effect. The equilibrium isotope effect of the pyrazine de-coordination – ethanol coordination is ~1.5 which is roughly the inverse of the following step. Since the faster equilibrium is reported to be dominant, we discarded the hypothesis *the inverse solvent KIE is due to a EIE* also since **Int-2** is high in energy and the assumption of an equilibrium is therefore questionable. The EtOH replacement by thiophene nevertheless seemed like a suitable step to show an inverse secondary kinetic isotope effect. The values calculated using different functionals for **L16** vary slightly, but all exhibit an isotope effect around ~0.7-0.8. This is within the experimental error of the experimentally observed solvent KIEs. Interestingly, the observed inverse KIE is with both ligands smaller for the C2 than for the C5 product (Table S19). We rationalize this in a similar way as for the aforementioned primary KIE. Due to the higher barrier of the C–H activation in the C5 position, the overall KIE is controlled slightly more by this TS, for which a solvent KIE of 1 is expected (we probed possible EtOH involvement in the C–H activation, but all barriers were higher in energy compared to an implicitly solvated TS).

3.4 Role of silver

Different roles of silver have been described in Pd-catalyzed C–H activations.[103,104] Initially we assumed the role of silver to be twofold for the formal reaction (see Figure S35a): as a scavenger of Br^- (formation of insoluble AgBr) and as a base (generation of H_2O from Ag_2O). In a hypothetical silver free reaction HBr would formally be generated, of which we assumed that it would act as a catalyst poison due to the accumulation of strongly binding bromide and the acidification of the reaction mixture.

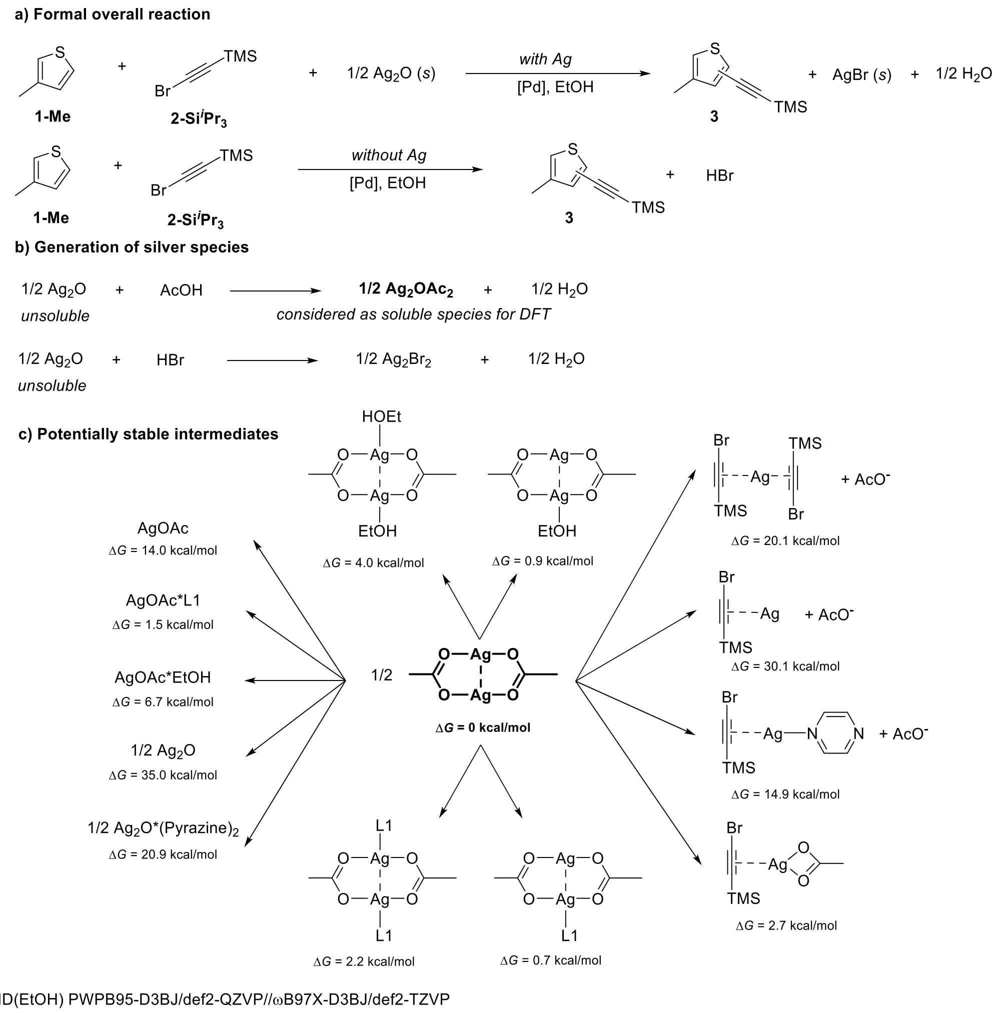

Figure S35: a) Formal overall reaction equation in the presence and absence of silver. b) formation of soluble silver species from silver oxide considered to be operative and suitable for DFT modelling. c) possible silver intermediates that were computationally considered as thermodynamically more stable species.

The potential involvement of silver in the present reaction was nevertheless investigated. Since Ag_2O is reported to be insoluble in ethanol[90] and hence difficult to model, we focused on silver acetate which reportedly is slightly soluble in EtOH and will therefore likely give more realistic results. The AgOAc dimer was used as the silver species of interest to model potential implications of homogenous silver. It is reported that silver oxide can react in the presence of acid to the respective silver salt and water.[105] This furthermore allows for a more reliable determination of reaction barriers by the more appropriate use of silver acetate over naked silver cations. During catalyst turnover formally HBr is generated so that poorly soluble AgBr could directly be generated from Ag_2O.[4] Since no HBr is present for the initial turnover we employ ½ Ag_2OAc_2 as the silver source in the computational investigation. Other intermediates derived from AgOAc were investigated as shown in Figure S35c, but appear all to be energetically unfavorable.

Stabilizing Ag–Alkyne interactions could in principle explain side reactions taking place in the absence of silver, but were not observed with the present substrate silver combination.

[4] Solubility in cold water: Ag_2O 0.0025 g/L, AgBr 0.000014 g/L, AgOAc 1.04 g/L, solubility in Ethanol: Ag_2O: no, AgBr: no.

An involvement of silver in the migratory insertion step was considered. In line with previous studies from Paton and Maiti[57] for a p template mediated N-acyl amino acid Pd-catalyzed system or for a rhodium system with identical alkyne source as reported by Zhang and Maiti,[51] no explicit need for silver for the migratory insertion step was observed. However, depending on the solvation model the involvement of silver is slightly favored or disfavored. Silver renders the MI less favorable according to the physically sounder dCOSMO-RS model. Importantly, even assuming an involvement of silver, its role would be secondary and the migratory insertion would still be feasible in the absence of silver according to the relative energy barriers. Since silver was experimentally found to be required, the following β-bromide elimination step was additionally investigated. The aforementioned study by Paton and Maiti[57] on a dual-ligand (pyrimidine template & N-acetylglycine) Pd-system suggests that the role of silver lies in facilitating the β-bromide elimination step following the MI. **TS-6-1** in Figure S36 would be systematically similar to their reported lowest silver free transition state. In our case de-coordination of the thiophene π-system and replacement by an additional pyrazine ligand (**TS-6-3**) leads to a large decrease in activation barrier and a more stable intermediate. This process even outcompetes the silver mediated transition in **TS-6-4**. In the Paton-Maiti system this additional N-heterocycle coordination was either unfortunately not explicitly considered or is sterically less feasible due to the more strained template. Overall, we concluded from our calculations that silver is not essential for product formation during the initial catalytic turnover.

a) Migratory Insertion

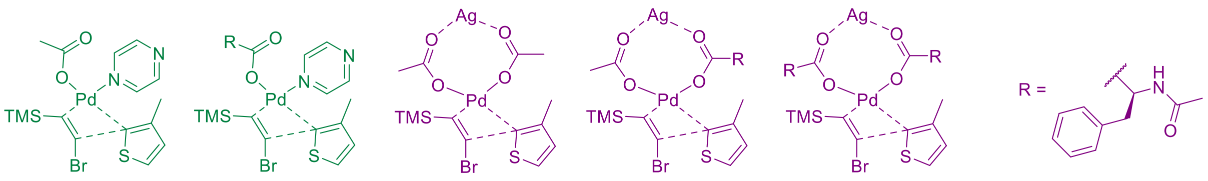

	**TS-5-1**	**TS-5-2**	**TS-5-3**	**TS-5-4**	**TS-5-5**	
CPCM	19.4	16.2	16.9	11.6	11.8	kcal/mol
SMD	18.9	15.3	16.4	11.5	11.3	kcal/mol
dCOSMORS*	24.2	22.8	27.2	24.1	27.4	kcal/mol

b) β-Bromide Elimination

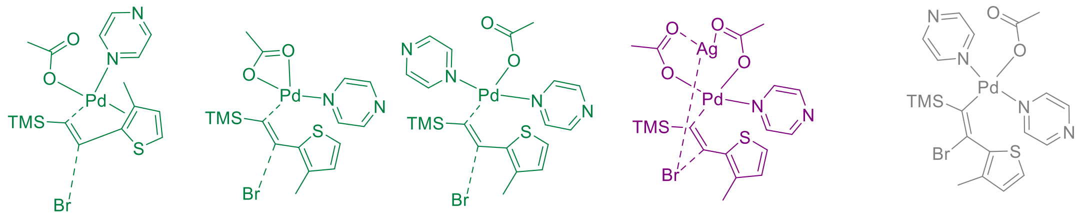

	**TS-6-1**	**TS-6-2**	**TS-6-3**	**TS-6-4**	**Int-7**	
CPCM	3.3	-8.4	-19.1	-11.4	-26.0	kcal/mol
SMD	9.0	-2.6	-14.0	-12.2	-27.0	kcal/mol
dCOSMORS*	9.4	-5.0	-12.1	0.8	-15.8	kcal/mol

ωB97X-D3BJ/def2-TZVP//D3BJ PBE0/def2-TZVP, Reference: Int-0-L2
*ωB97X-D3BJ/def2-QZVPP////D3BJ PBE0/def2-TZVP

Figure S36: Silver and silver-free transition states for a) the migratory insertion and b) the β-bromide elimination.

Previous studies did not assess the possibility of silver to recycle the catalyst or to suppress side reactions. During the initial catalyst cycle **PdL1OAcBr** (**Int-10**) is generated (see Figure S37). Silver facilitates (ΔG = 0.7 kcal/mol vs 9.1 kcal/mol in absence of Ag) striping of bromide which frees an essential coordination space for

the bidentate ligand and allows for regeneration of the resting state. The slightly endergonic recycling of $Pd(OAc)_2$ is to be taken with a grain of salt and is expected to be exergonic in reality, since the insolubility of AgBr(s) is not adequately factored in. Experimentally the enthalpy of precipitation of AgBr in H_2O amounts to -20.3 kcal/mol.[106] The shortcomings of implicit solvation models and accounting for solubility would require explicit solvation and modeling of solid silver bromide which is outside the scope of this study and not necessary to qualitatively account for the observed behavior. It can be concluded that the regeneration of the resting state in the presence of silver (precipitation of AgBr) is therefore exergonic.

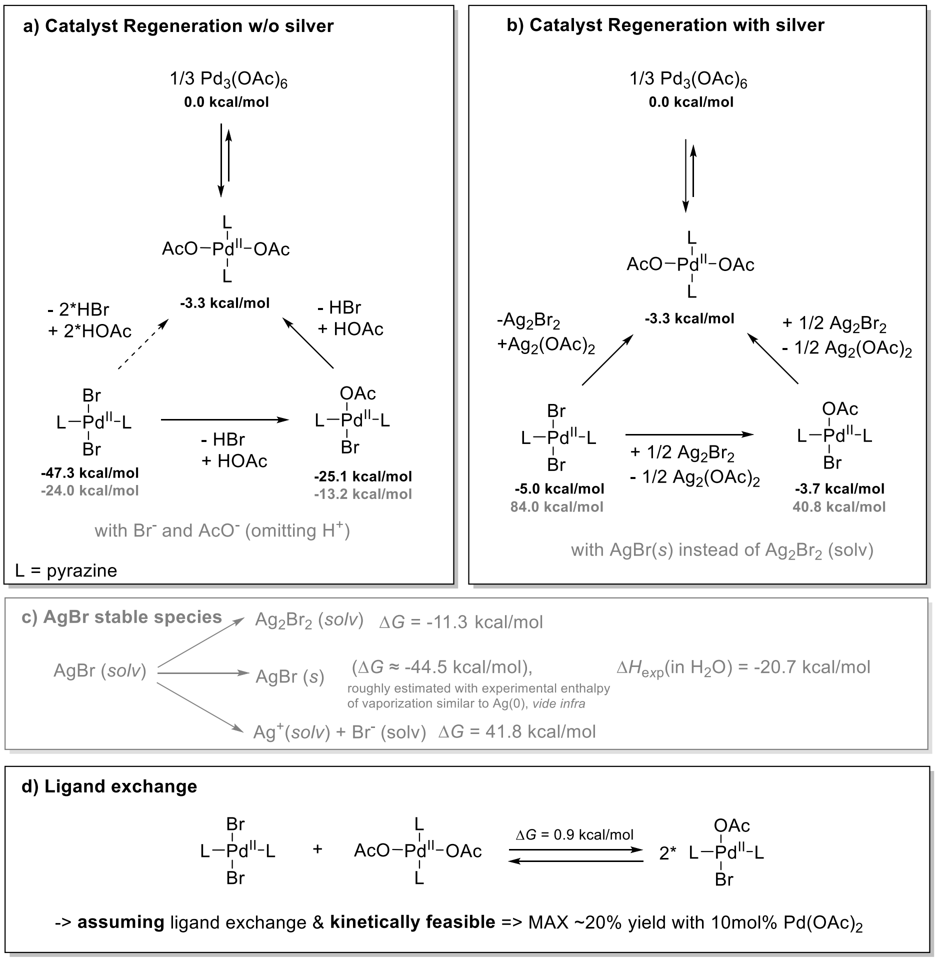

Figure S37: Comparison of catalyst regeneration (a) in the presence and (b) absence of silver with a model complex. (c) Different silver intermediates considered. (d) Possible ligand exchange.

Overall this explains, why a catalytic turnover in the absence of silver is not feasible due to the additional penalty of at least ~10kcal/mol. It has to be noted that upon ligand exchange and generation of $PdBr_2L1_2$, $Pd(OAc)_2$ can be regenerated to some extent (see Figure S37d). A maximum of ~20% yield could hence be obtained using 10 mol% of catalyst and no silver. Experimentally only a much lower amount of product formation is observed in the absence of silver and, more strikingly, only a low amount of product is formed using stoichiometric amounts of $Pd(OAc)_2$ and ligand but no Ag_2O. The halide-scavenging hypothesis described above does not yet account for this experimental observation (Figure S78).

The role of silver can be fully deciphered by computationally inspecting the experimentally observed formation of a Glaser-type bisalkyne side product (SP) shown in Figure S38.

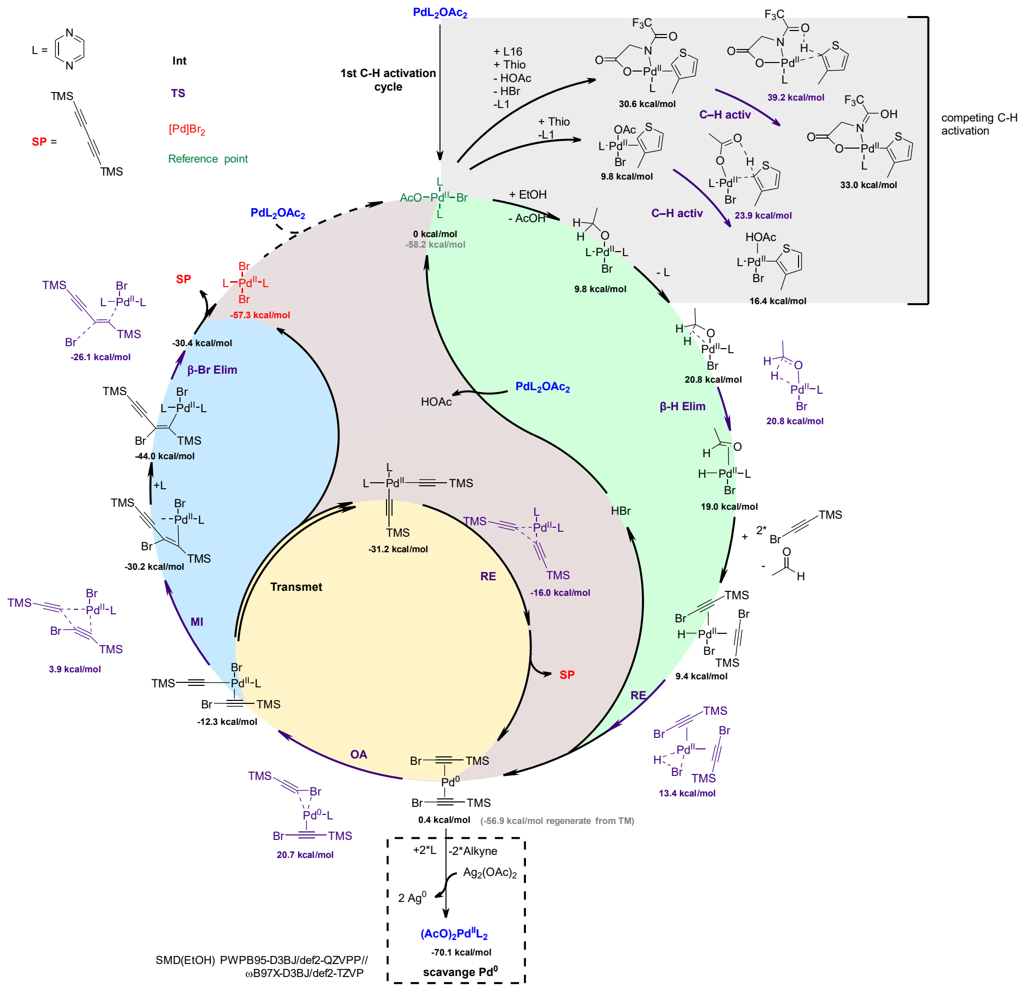

Figure S38: Proposed pathway for formation of Glaser type byproduct after one catalyst turnover with Pd(OAc)Pyrazine$_2$Br as the new reference point.

After the first catalytic cycle **Pd(OAc)$_2$** is converted to **PdL1OAcBr** (**Int-10**). In the absence of silver, the pathways towards C–H activation of the thiophene are energetically unfavorable since either Br needs to dissociate to free a coordination space for the bidentate N-acyl amino acid ligand or only a monodentate CMD group like acetate can cleave the C–H bond. Both processes are energetically more costly (39.2 and 23.9 kcal/mol) compared to the initial C–H activation. Ethanol can coordinate as an anionic ligand and undergo β-hydride elimination which is less favorable in the absence of bromide compared to the competing pathways (*vide infra*). This process is energetically favored even when compared to the initial C–H activation. The following reductive elimination generates a Pd^0 species and HBr. HBr in turn can react with the remaining Pd(OAc)$_2$ to reform more PdL1OAcBr. Notably, the Pd^0 species could readily be re-oxidized in the presence of silver. This illustrates a potential second role of silver in the present reaction.

We estimated the free reaction enthalpy for the re-oxidation using an approach suggested by Harvey et al.[107] which is also employed in a recent study by Bistoni et al. for solid zinc.[108] The Gibbs free energy of Ag(0) is hereby calculated by subtracting the experimental sublimation free energy (here 58.3 kcal/mol at 313K for 1 mol solid silver)[109] from the free energy of silver in the gas phase. A single zinc atom was used in the literature whereas we employ ½ of a silver dimer. This is necessary since unlike zinc the unpaired electron renders the single Ag atom artificially destabilized in the gas phase compared to the solid phase. We probed the influence of using a zinc dimer in the present palladium oxidation (EtOH + $ZnCl_2$ -> $Zn(0)_{dim/mon}$ + Acetaldehyde + 2*HCl)

to see whether the dimerization itself would lead to erroneous results. In case of Zn, the monomer would lead to a 2 kcal/mol lower overall free enthalpy for oxidizing Pd compared to the dimer, whereas in the case of silver (EtOH + Ag_2OAc_2 -> 2*Ag(0)$_{dim/mon}$ + Acetaldehyde + 2*AcOH) the difference would unphysically disfavor the monomer by >380 kcal/mol. We therefore considered our treatment of a silver dimer in gas phase to be sounder. An alternate way to initially form Pd^0 instead of via EtOH could be a Heck-type coupling of an additional thiophene instead of an alkyne yielding a homocoupled thiophene dimer. Since no suspiciously high conversion in thiophene is observed (and a constant reformation of Pd^0 would be required) and since the electronically similar Heck-type sequence described in lower part Figure S15 is rather high in energy, we did not investigate this possibility further.

In the absence of silver, the alkyne can undergo oxidative addition at Pd^0. Two potential pathways can now generate the side product. In the first pathway two equivalents of alkynylated Pd^{II} species undergo transmetallation and form $PdBr_2L_2$ and a dialkynylated intermediate which can undergo reductive elimination and regenerate Pd^0. The second possibility is a migratory insertion and similar to the productive pathway and subsequent β-bromide elimination which also liberates $PdBr_2L_2$ as product. $PdBr_2L_2$ could theoretically undergo ligand exchange with $Pd(OAc)_2$ to form PdBrOAcL and close the catalytic cycle. It has to be noted that for the formation of one equivalent of side product two equivalents of $Pd(OAc)_2$ are converted to their monobrominated analog. Considering the equilibrium described in Figure S37d, a maximum conversion of alkyne equal to the catalyst loading should be observed for initial product formation and subsequently predominant homocoupling (assuming no other decomposition pathways and the absence of silver). This is in line with the experimental observations in Figure S78. After initial catalyst turnover, the formation of homocoupled alkyne seems to be kinetically favored. This can be explained by the barriers for the respective turnover limiting steps: in the first catalytic turnover in case of **L16** the C–H activation is predicted to be turnover limiting. The EtOH coordination and β-hydride elimination is less favored ($\Delta\Delta G^{\ddagger}$ = 4.4 kcal/mol). After initial formation of $PdBrEtOL1_2$ the analogous β-hydride elimination is not only favored compared to the bromide coordinated C–H activation but also faster compared to the initial C–H activation, explaining, why after a certain amount of product formation in the stoichiometric experiment mainly side product is formed. This is confirmed by calculations in Figure S39 using dCOSMORS, SMD and CPCM and methods that performed well in Table S16 compared to the CCSD(T) results. Since previously it was noted that the solvation model can have a large influence on steps involving explicit EtOH (i.e. ligand exchange), Figure S39 serves to confirm that, while the quantitative values are affected by the solvation model, the qualitative trends regarding the most stable step hold true regardless of functional and solvation model.

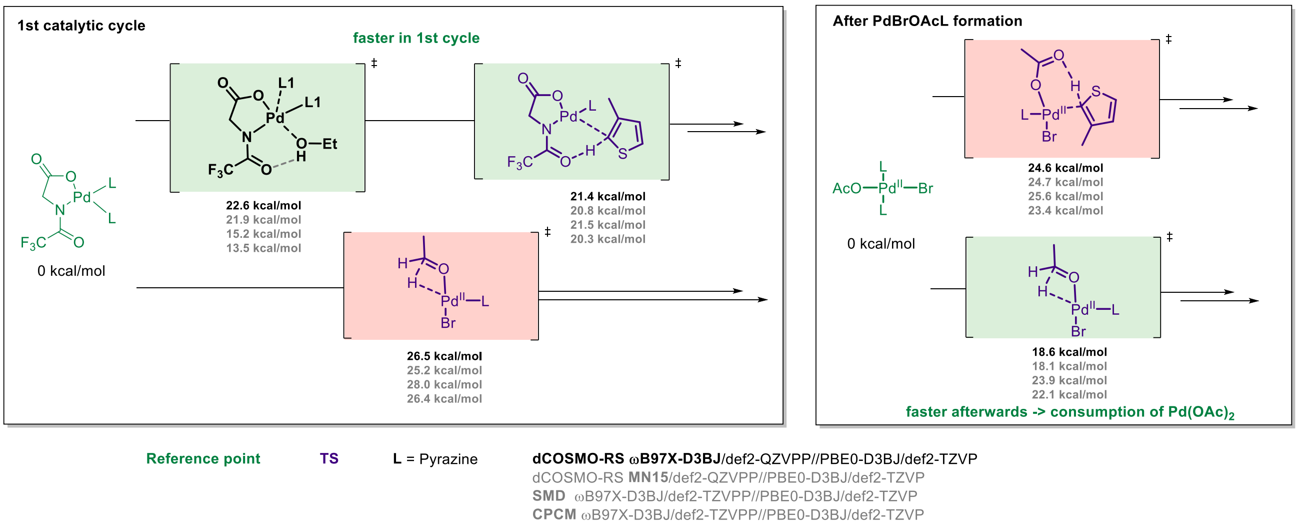

Figure S39: Comparison of the initial high energy steps during the first catalytic turnover and after $PdBrOAcL_2$ formation in the absence of silver.

In summary, the role of silver is to strip Br^- from palladium, thereby preventing an otherwise favorable side reaction and regenerate the active catalyst. Additionally, it could also serve to re-oxidize Pd(0) species formed in side-reactions and therefore prolong the catalyst lifetime. An involvement of silver in the other steps of product formation was found to be unnecessary since all steps are predicted to be energetically feasible even in the absence of silver. However, since the barriers with and without silver are rather similar a complete innocence of silver in these steps under all conditions and ligand combinations cannot be guaranteed.

4 Experimental Studies

4.1 General Information

Nuclear Magnetic Resonance Spectroscopy (NMR)

Proton (^{1}H) nuclear magnetic resonance spectra and carbon (^{13}C) nuclear magnetic resonance spectra were recorded on a Bruker DRX-500 spectrometer. Chemical shifts are given relative to tetramethylsilane (TMS) and are referenced to the residual proton signal of the NMR solvent.[1] Data are presented as follows: chemical shift, multiplicity (s=singlet, d=doublet, t=triplet, q=quartet, m=multiplet, and br=broad signal), coupling constant in Hertz (Hz) and integration. Full assignment of the signals was achieved by using 2D NMR techniques (^{1}H–^{13}C HSQC, ^{1}H–^{13}C HMBC, ^{1}H–^{1}H COSY-DQF).

^{2}H NMR spectra were recorded in deuterated solvent, using a solvent that has a sufficiently large chemical shift difference with the region of interest. The zg2h pulse sequence was employed.

Gas Chromatography (GC)

An Agilent Technologies 7890B setup equipped with a HP5 column (30 m × 0.32 mm, film thickness: 0.25 µm) and an FID sensor was used for GC-FID analysis.

For substrate, reagent, and product quantification gas chromatography coupled with a flame ionization detector (GC-FID) was employed. 1,3,5-Trimethoxybenzene or trimethyl benzene-1,3,5-tricarboxylate (for in situ quantification) was used as internal standard and calibrated prior to the experiment with the pure sample of interest.

Good signal intensity and a retention time on the GC-FID in a similar magnitude with the products retention time renders especially trimethyl benzene-1,3,5-tricarboxylate a suitable internal standard that can be used for *ex-situ* after competition of the reaction but also *in-situ* to monitor reaction progress since it is electron poor enough to not react under the reaction conditions. The signals furthermore don't overlap with the product on a ^{1}H NMR so the results can further be corroborated via NMR if necessary. A heat gradient of 10°C/min starting with 50°C (hold 3 min) to 300°C (hold 3 min) resulting in an overall 31min run was chosen (see Figure S40).

(a)

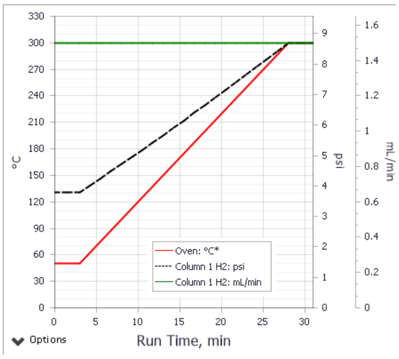

(b)

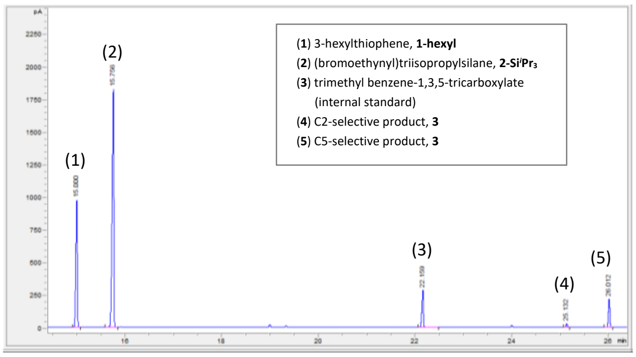

(c) (d)

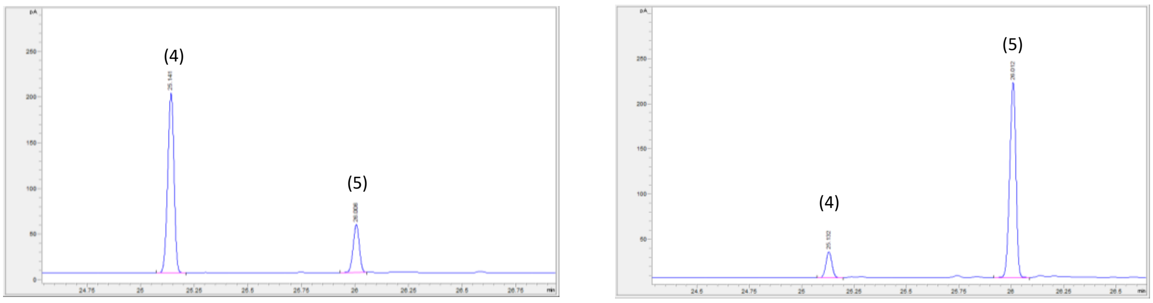

Figure S40: (a) GC-FID temperature gradient, (b) exemplary chromatogram (t_{ret} = 14.5 – 26.2 min) with all relevant species. (c) Zoom of the product region for a C2 selective reaction using **L16** and (d) a C5 selective reaction using **L2**.

For in-situ quantification an aliquot (20 µL, typical values: *c*(thiophene)$_0$ = 0.1 M, *c*(IS) = 0.04 M, *c*(L2,**3**-hexyl C2+C5)$_{after10min}$ = 0.006 M) was retrieved, filtered over a mini-silica plug, eluted with EtOAc, and collected in a GC vial (1 mL, *c*(thiophene)$_0$ = 2 mM, *c*(IS) = 0.8 mM, *c*(L2,C2+C5)$_{after10min}$ = 0.12 mM). Per GC run 1 µL sample were injected. For the instrument used the detection limit is <1.4 pg C/s which is well below the samples used (average peak width: 3s; $\mathbf{C_{10}}H_{16}S$ 8.0·10^4 pg C/s, $\mathbf{C_{12}}H_{12}O_6$ 3.8·10^4 pg C/s, $\mathbf{C_{21}}H_{36}SSi$ = 1.0·10^4 pg C/s).[110] According to the manufacturer the linear dynamic range is over the entire concentration range of 10^7 in a single run which largely contains the studied concentration range.[110] To calibrate the GC-FID each compound was weighed in with the internal standard in at least three different ratios and overall concentrations. Each sample was injected twice on the GC-FID and the average of the response factor (RF) was calculated from the duplicate injection. Afterwards the average of all different ratio/concentration samples was calculated and used to determine the final RF values (see eq. 5) used in the kinetic measurements.

$$RF = \frac{A_{\mathrm{x}}}{A_{\mathrm{IS}}} \cdot \frac{c_{\mathrm{IS}}}{c_{\mathrm{x}}} = \frac{A_{\mathrm{x}}}{A_{\mathrm{IS}}} \cdot \frac{n_{\mathrm{IS}}}{n_{\mathrm{x}}} \qquad (5)$$

with X = analyte, IS = internal standard, A = area of GC-FID peak and can be simplified since the identical volume is used.

Using an internal standard rather the absolute concentration allows for more reliable determination of concentrations since systematic errors like differences in volume during aliquotation become irrelevant.

We probed the whether a linear dynamic range was observed in the relevant concentration range (see Figure S41). We used single concentrations ranging from 0.01 mM to 100 mM which by far exceed our expected concentration ranges (*vide supra*). The high R^2 values indicate that the studied concentration ranges are well within the linear dynamic range allowing for comparison of even unequal IS:product ratios.

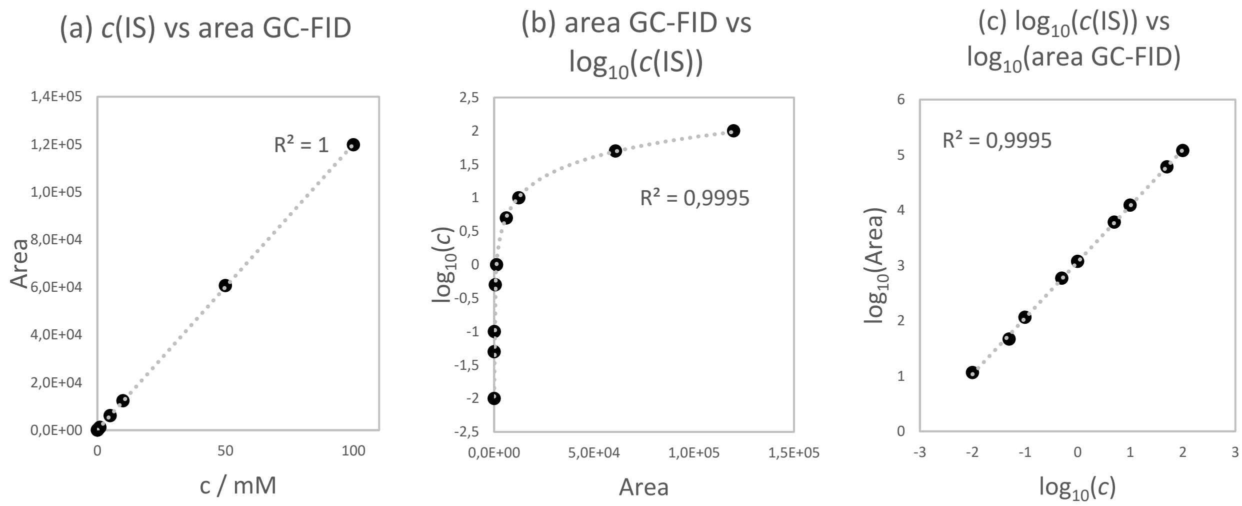

Figure S41: Concentration of trimethyl benzene-1,3,5-tricarboxylate as a function of the GC-FID chromatogram response area. All concentrations in mM. (a) concentration of IS vs area with a linear fit, (b) area of GC-FID vs log10 to allow for a logarithmic fit, (c), log10 of area and concentration with a linear fit.

Reaction monitoring with in-situ internal standard and aliquotation combined with GC-FID[111,112] or NMR[50] for Pd catalyzed C–H activation at a similar scale (and with insoluble reaction partners) has been reported in the literature.

GC-MS spectra were recorded on an Agilent Technologies 7890A GC-system with an Agilent 5975C VL MSD or an Agilent 5975 inert Mass Selective Detector (EI) and a HP-5MS column (30 m × 0.32 mm, film thickness: 0.25 µm).

Additional data from the experimental analysis (concentration vs time plots, raw NMR spectra, calculation of elasticity coefficient) are deposited at the ZENODO archive (DOI: 10.5281/zenodo.15126469).

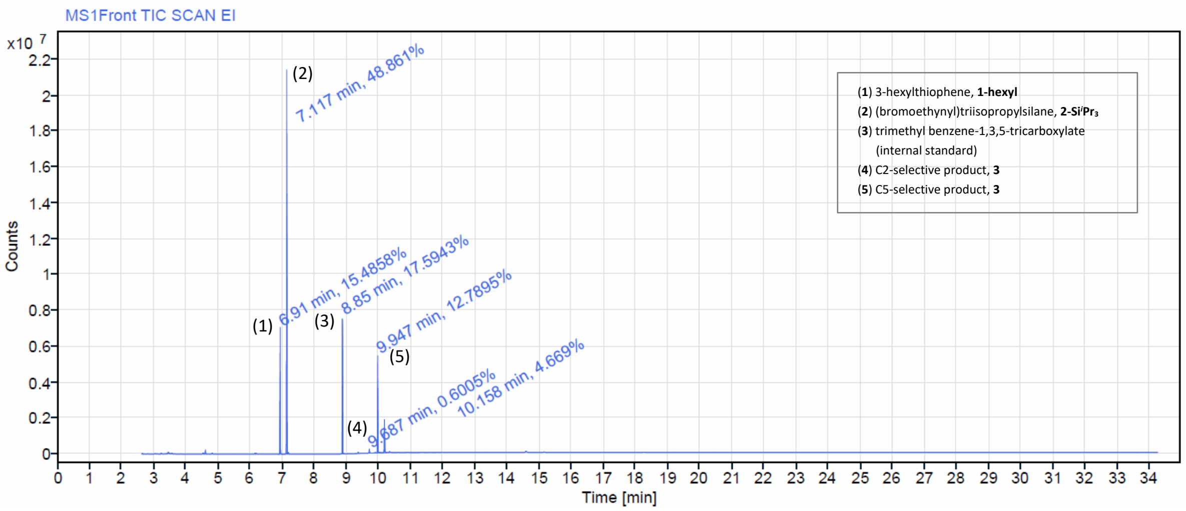

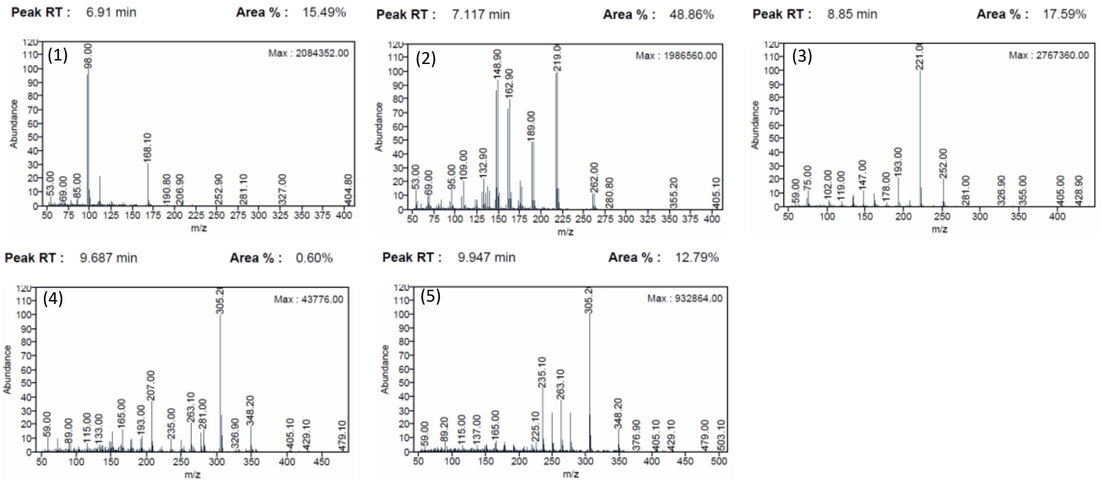

Figure S42: Overview of a typical GC-MS chromatogram for a C5-selective reaction with the respective EI-MS signals.

To verify reproducibility/consistency between different runs, we conducted similar reactions, e.g. the VTNA series for one ligand in a matter of a few days such that no changes in the GC-calibration, solvent and reagent were to be expected. Fine balances, anti-static equipment and back-weighing (re-weighing the empty weighing equipment after transfer of the compound into the reaction vessel to ensure a 0 tare) were employed to ensure consistent results. We also performed a model reaction in triplicate to evaluate the variability within runs set up on the same day. The sample standard deviation for the threefold repetition is given in Figure S43, showing a sufficient reproducibility/consistency to support the conclusions drawn from the kinetic analysis in this study.

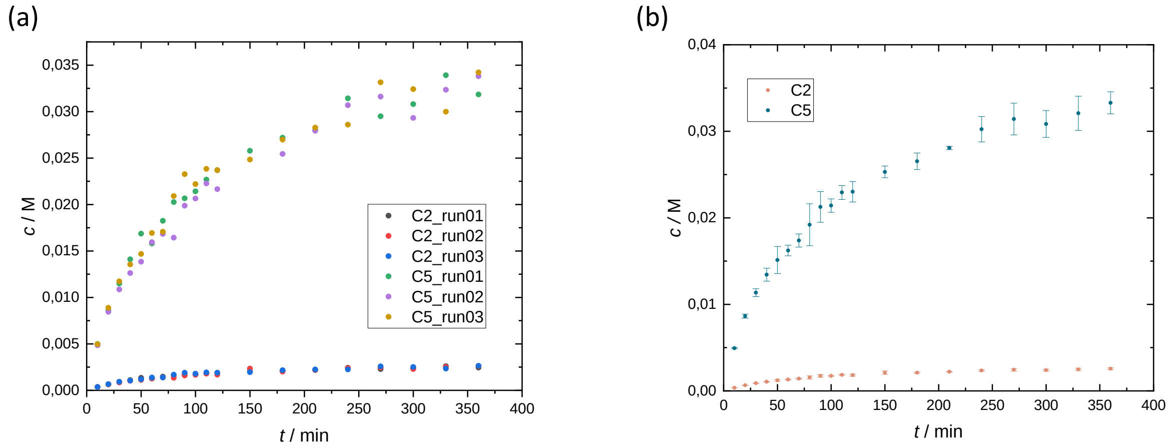

Figure S43: (a) Three-fold repetition of a reaction and (b) averaged runs with the respective sample standard deviation for C2 and C5.

Chemicals

Chemicals used for synthesis were purchased from commercial vendors, unless otherwise specified.

The following commercial compounds were used in mechanistic experiments:

$Pd(OAc)_2$, CAS : 3375-31-3, vendor : SigmaAldrich (≥99.9% trace metals basis)

EtOH, CAS: 64-17-5, vendor: Acros; dry EtOH was transferred to a Schlenk tube and stored over activated 4Å molecular sieves

EtOD, CAS: 925-93-9, vendor: Sigma Aldrich

Trimethyl 1,3,5-benzenetricarboxylate, CAS : 2672-58-4, vendor : TCI

(Bromoethynyl)triisopropylsilane, **2-Si^{i}Pr$_3$**, CAS : 111409-79-1, vendor : BLDPharm

3-Hexylthiophene, **1-hexyl**, CAS: 1693-86-3, vendor: TCI or BLDPharm

N-Acetyl-L-phenylalanine, **L2**, CAS : 2018-61-3, vendor : TCI

2-(2,2,2-Trifluoroacetamido)acetic acid, **L16**, CAS: 383-70-0, SigmaAldrich

Pyrazine, **L1**, CAS : 290-37-9, vendor : SigmaAldrich

Silver(I)oxide, CAS: 20667-12-3, vendor : Carbolution

(2*R*)-2-Acetamido-3-(2,6-difluorophenyl)propanoic acid, **L20**, CAS: 266360-56-9, vendor : Activate Scientific

(2*R*)-2-Acetamidobutanoic acid, **L22**, CAS: 34271-27-7, vendor : BLDPharm

Synthesized compounds

2,5-*d*$_2$-3-hexylthiophene (2,5-*d*$_2$-1-hexyl)

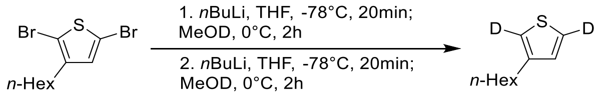

The title compounds was synthesized according to a modified literature procedure.[50]

An oven-dried Schlenk round bottom flask was cycled on a Schlenk line under N_2 protective gas and charged with 2,6-dibromo-3-hexylthiophene (2.00 mL, 3.04 g, 9.32 mmol) and anhydrous THF (30 mL). The solution was cooled -78°C and *n*-BuLi (4.47 mL, 2.5 M in hexanes, 11.2 mmol, 1.2 equiv.) was added in two portions by syringe. After an apparent color change to yellow, the solution was stirred for 20min. The flask was transferred to an ice bath and stirred for 10 minutes. MeOD (1.6 mL) was subsequently added by syringe and the reaction was stirred for additional 2h. Water was added to the reaction mixture and extracted with Et_2O. The combined organic fractions were washed with water, dried over $MgSO_4$ and filtered through a plug of silica. The solvent was removed under reduced pressure and afforded crude 2-*d*-5-bromo-3-hexylthiophene (1.99 g, ~8.03 mmol, ~86%). The initial procedure was repeated.

An oven-dried Schlenk round bottom flask was cycled on a Schlenk line under N_2 protective gas and charged with the prior crude and anhydrous THF (20 mL). The solution was cooled -78°C and *n*-BuLi (3.85 mL, 2.5 M in hexanes, 9.63 mmol, 1.2 equiv.) was added in two portions by syringe. After an apparent color change to yellow, the solution was stirred for 20min. The flask was transferred to an ice bath and stirred for 10 minutes. MeOD (1 mL) was subsequently added by syringe and the reaction was stirred for additional 2h. Water was added to the reaction mixture and extracted with Et_2O. The combined organic fractions were washed with water, dried over $MgSO_4$ and the solvent was removed under reduced pressure. The residue was redissolved in *n*-pentane and filtered through a silica plug. To remove traces of remaining mono- or di-bromo starting material, as evidenced by GC-MS, the liquid crude was purified by Kugelrohr distillation (130°C, 12 mbar) to afford the title compound as a colorless liquid (923 mg, 5.42 mmol, 58%).

^{1}H NMR (500 MHz, $CDCl_3$) δ = 6.95 (s, 1H), 2.77 – 2.49 (m, 2H), 1.68 – 1.60 (m, 2H), 1.42 – 1.28 (m, 6H), 0.94 – 0.88 (m, 3H) ppm.

^{13}C NMR (126 MHz, $CDCl_3$) δ = 143.3, 128.3, 124.9 (t, $^1J_{C\text{-}D}$ = 28.1 Hz), 119.6 (t, $^1J_{C\text{-}D}$ = 27.9 Hz), 31.8, 30.7, 30.4, 29.2, 22.8, 14.2 ppm.

HRMS (ESI) m/z calcd. for $C_{10}H_{15}D_2S^+$ $[M+H]^+$: 171.11765; found: 171.11693.

D_{Tot} (EI-MS) = 1.99

The spectroscopic and mass data agree with the literature.[113]

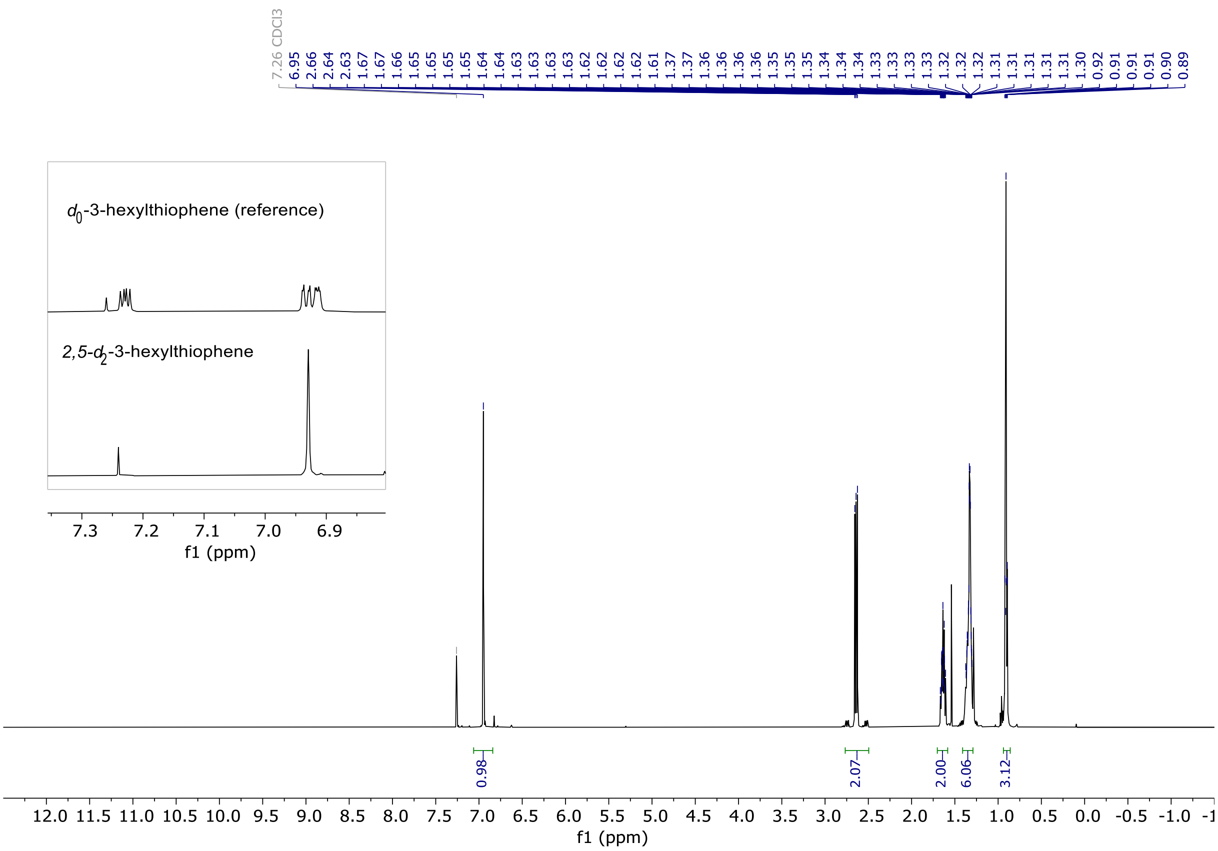

Figure S44: ^{1}H-NMR in $CDCl_3$ of 2,5-d_2-3-hexylthiophene.

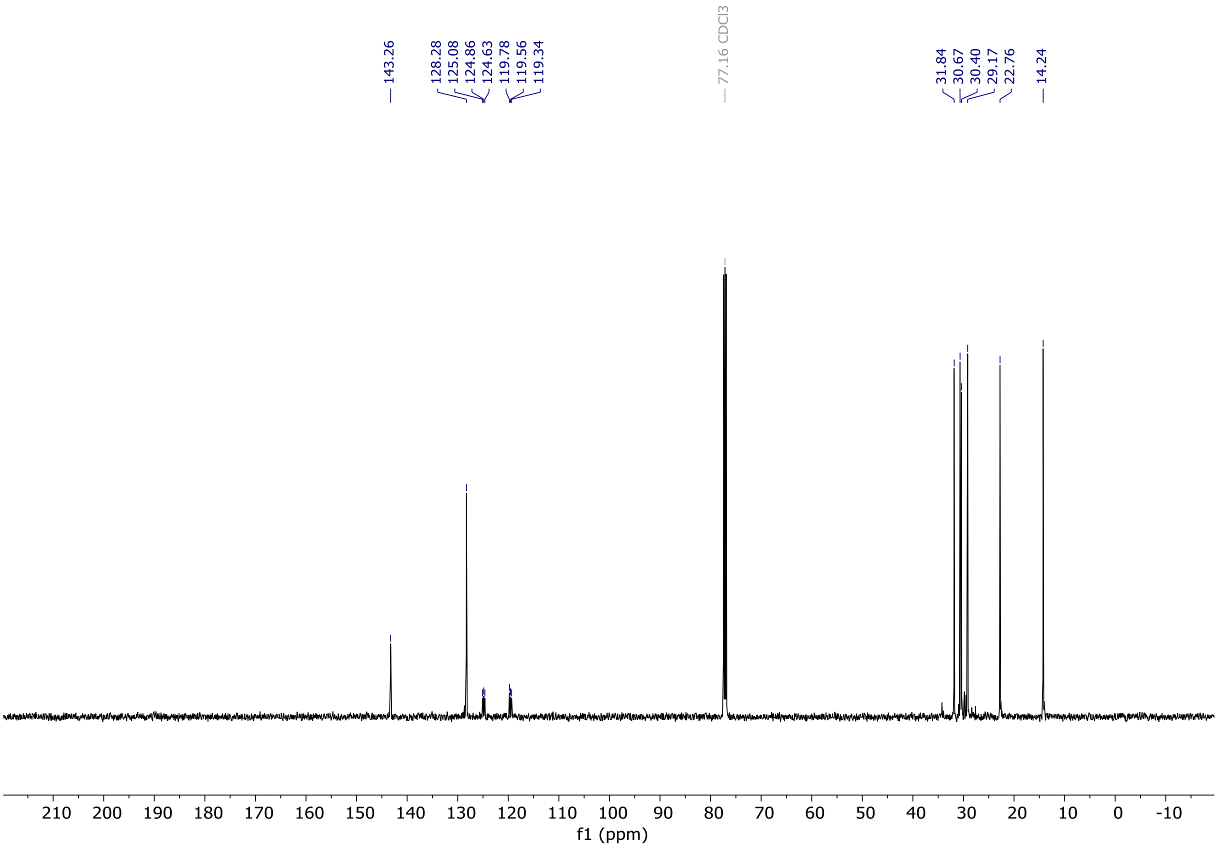

Figure S45: $^{13}C\{^{1}H\}$-NMR in $CDCl_3$ of 2,5-d_2-3-hexylthiophene.

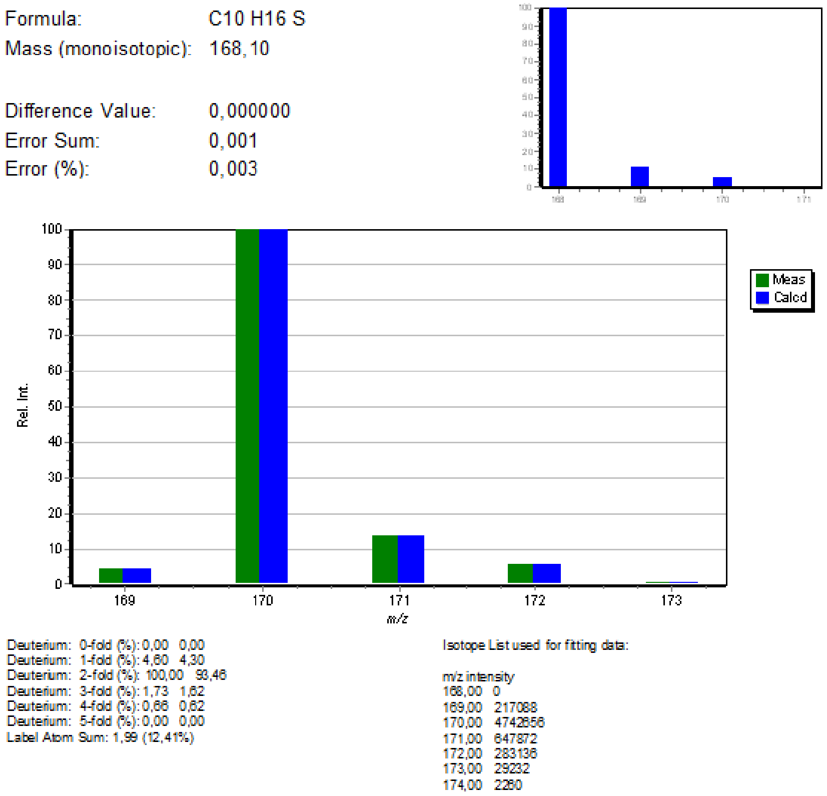

Figure S46: EI-MS determination of deuteration degree for 2,5-d_2-**1-hexyl**.

1-(2,2,2-trifluoroacetamido)cyclopentanecarboxylic acid (L18)

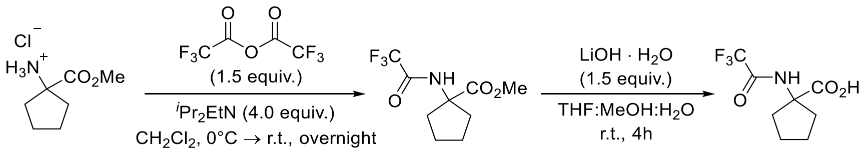

Methyl 1-aminocyclopentanecarboxylate hydrochloride (1.08 g, 6.00 mmol) was added to a round bottom flask and dissolved in dry CH_2Cl_2 (15 mL). Diisopropylethylamine (4.08 mL, 3.10 g, 24.0 mmol, 4.0 equiv.) was added dropwise and the reaction mixture was cooled in an ice bath to 0°C. 2,2,2-trifluoroacetic anhydride (1.25 mL, 1.89 g, 8.99 mmol, 1.5 equiv.) was added dropwise and the reaction was allowed to warm to room temperature and was stirred overnight. The solvent was removed under reduced pressure and methyl 1-(2,2,2-trifluoroacetamido)cyclopentanecarboxylate was purified using column chromatography (SiO_2, 0% → 20% ethyl acetate in *n*-pentane) and was obtained as a colorless crude oil (1.02 g, ~4.25 mmol, ~71%).

Methyl 1-(2,2,2-trifluoroacetamido)cyclopentanecarboxylate and lithium hydroxide monohydrate (268 mg, 6.39 mmol, 1.5 equiv.) was added in a round bottom flask and was dissolved in a solvent mixture of tetrahydrofuran, methanol and water (21 mL, THF:MeOH:H_2O = 1:1:1) and stirred at room temperature for 4h. The reaction mixture was basified with diluted $NaHCO_3$ (pH = 12) and washed twice with CH_2Cl_2. The combined aqueous layers were acidified with HCl (1 M), extracted four times with CH_2Cl_2 and dried over Na_2SO_4 and filtered. The solvent was removed in vacuo and the residue was dissolved in a minimal amount of *i*-PrOH. The solution was layered with *n*-pentane and crystalized at -20°C. The remaining solvent was removed and the crystals were dried under reduced pressure, titurated with *n*-pentane and dried at high vacuum. The title compound was obtained as colorless crystals (327 mg, 1.45 mmol, 24% over two steps).

^{1}H NMR (500 MHz, d_4-MeOH) δ = 4.88 (br. s, 2H), 2.34 – 2.18 (m, 2H), 2.14 – 1.99 (m, 2H), 1.88 – 1.69 (m, 4H) ppm.

^{13}C NMR (126 MHz, d_4-MeOH) δ = 176.2, 158.8 (q, $^2J_{C-F}$ = 37.1 Hz), 117.3 (q, $^1J_{C-F}$ = 286.8 Hz), 67.7, 37.6, 25.5 ppm.

^{19}F NMR (471 MHz, d_4-MeOH) δ = -75.71 ppm.

HRMS (ESI) m/z calcd. for $C_8H_9F_3NO_3^-$ [M-H]$^-$: 224.05340; found: 224.05369.

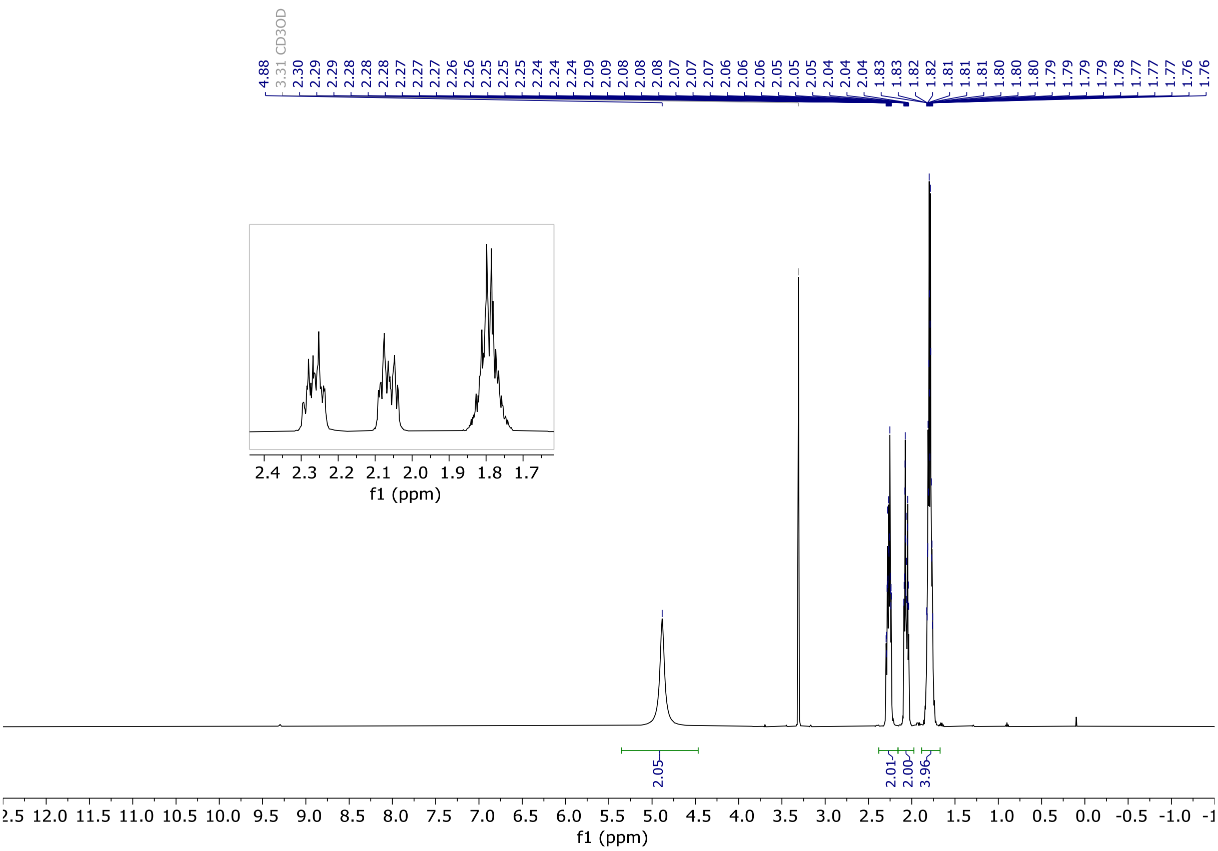

Figure S47: ^{1}H-NMR in d_4-MeOH of 1-(2,2,2-trifluoroacetamido)cyclopentanecarboxylic acid (**L18**).

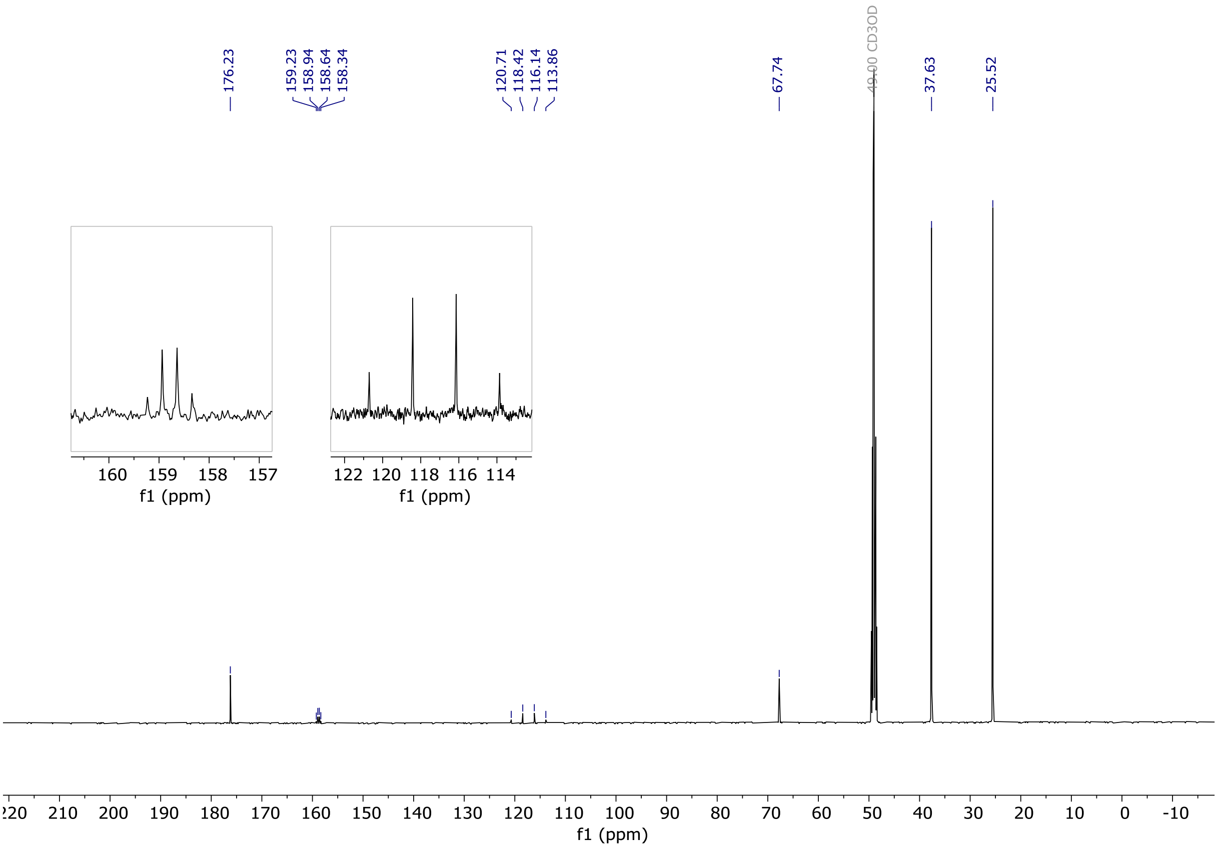

Figure S48: ^{13}C{^{1}H}-NMR in d_4-MeOH of 1-(2,2,2-trifluoroacetamido)cyclopentanecarboxylic acid (**L18**).

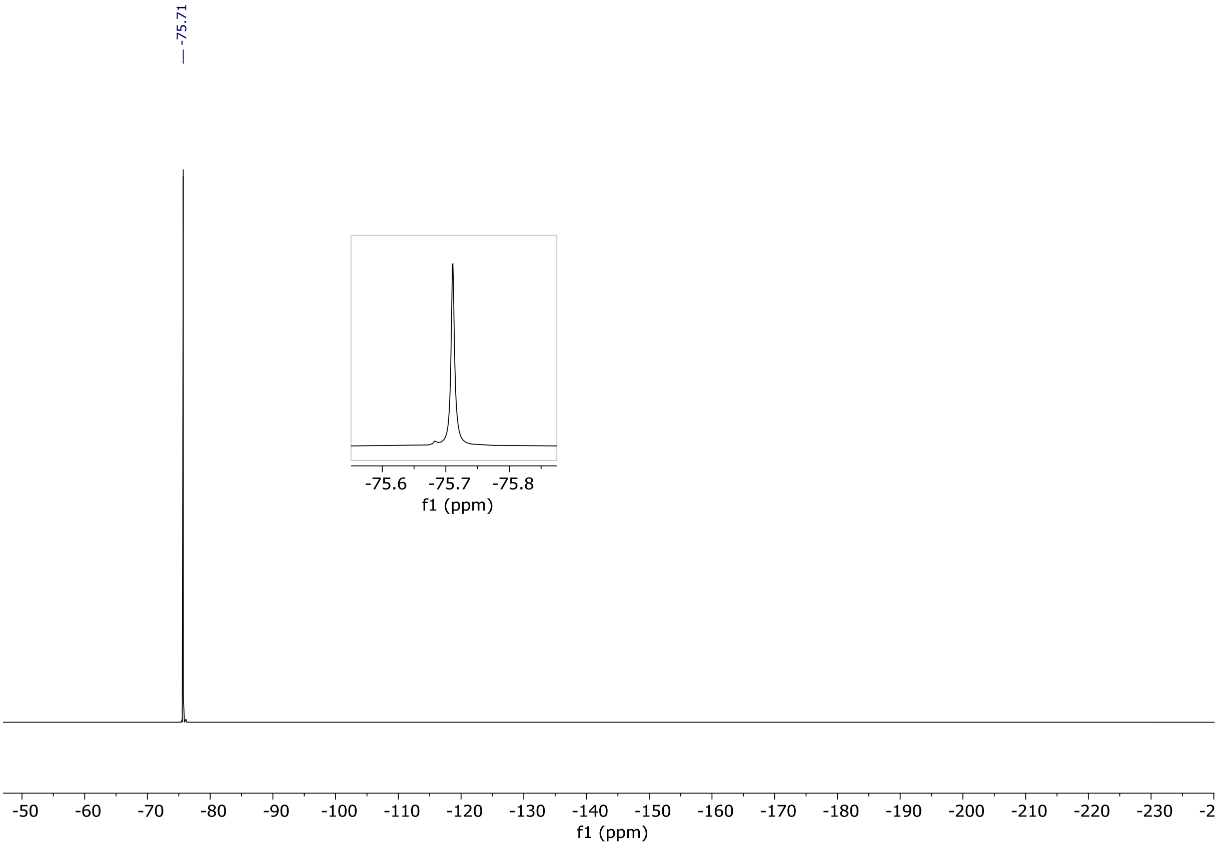

Figure S49: $^{19}F\{^1H\}$-NMR in d_4-MeOH of 1-(2,2,2-trifluoroacetamido)cyclopentanecarboxylic acid (**L18**).

(*S*)-2-acetamido-3-(*p*-tolyl)propanoic acid (L19)

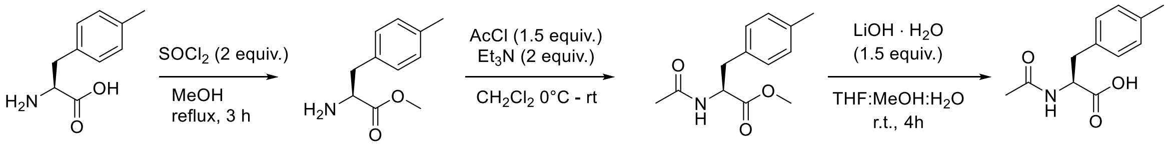

The title compound was synthesized from an adapted literature procedure.[114]

4-Methyl-L-phenylalanine (200 mg, 1.16 mmol, 1.0 equiv.) was dissolved in MeOH (11 mL) then cooled to 0°C. Thionyl chloride (160 µL, 266 mg, 2.0 equiv.) was added dropwise at 0°C. The reaction mixture was refluxed for 3h. The reaction mixture was allowed to cool to room temperature and the solvent was removed under reduced pressure to afford the crude product and used directly in the next step without purification.

The above obtained crude (*S*)-methyl 2-amino-3-(p-tolyl)propanoate was dissolved in CH_2Cl_2 (11 mL) and cooled to 0 °C. Triethylamine (311 µL, 226 mg, 2.23 mmol, 2.0 equiv.) was added slowly. Acetyl chloride (120 µL, 131 mg, 1.67 mmol, 1.5 equiv.) was added dropwise. The reaction mixture was stirred at room temperature for 3h and quenched with aqueous NH_4Cl. The resulting solution was extracted with CH_2Cl_2 and the combined organic phases were washed with brine and dried over anhydrous Na_2SO_4. The solvent was removed under reduced pressure and the residue was purified using column chromatography (SiO_2, 0% → 5% Methanol in CH_2Cl_2) giving the target ester as a colorless oil (210 mg, 0.89 mmol, 79% over 2 steps).

(*S*)-methyl 2-acetamido-3-(*p*-tolyl)propanoate and lithium hydroxide monohydrate (56.2 mg, 1.34 mmol, 1.5 equiv.) were added to a round bottom flask, dissolved in a mixture of tetrahydrofuran, methanol, and water (4.5 mL, THF:MeOH:H_2O = 1:1:1), and stirred at room temperature for 4h. The reaction mixture was basified with diluted $NaHCO_3$ (pH = 12) and washed twice with CH_2Cl_2. The aqueous layer was acidified with HCl (1 M), extracted four times with CH_2Cl_2 and the combined organic layers were dried over Na_2SO_4. The solvent was removed under reduced pressure and the residue was further dried under high vacuum. The title compound was obtained as yellow solid (180 mg, 90% yield). The observed analytical data are in accordance with those reported in the literature.[114]

^{1}H NMR (500 MHz, DMSO) δ = 12.62 (s, 1H), 8.14 (d, *J* = 8.0 Hz, 1H), 7.13 – 7.05 (m, 4H), 4.36 (m, 1H), 2.98 (m, 1H), 2.78 (m, 1H), 2.25 (s, 3H), 1.77 (s, 3H) ppm.

^{13}C NMR (126 MHz, DMSO) δ = 173.1, 169.1, 135.2, 134.4, 128.8, 128.6, 53.5, 39.3, 22.2, 20.5 ppm.

HRMS (EI pos) m/z: Calcd for $C_{12}H_{15}N_1O_3$ 221.10519, Found 221.10512.

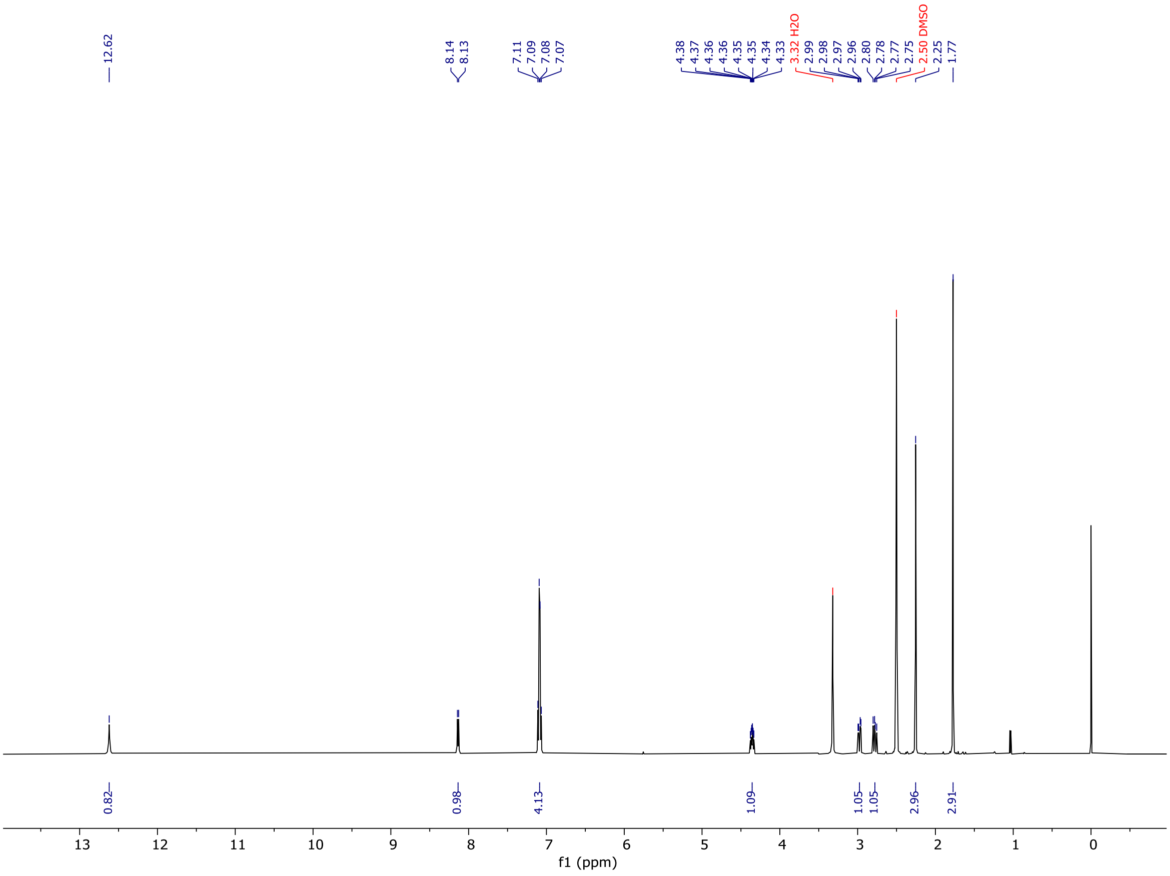

Figure S50: ^{1}H-NMR in d_6-DMSO of (*S*)-2-acetamido-3-(p-tolyl)propanoic acid, **L19**

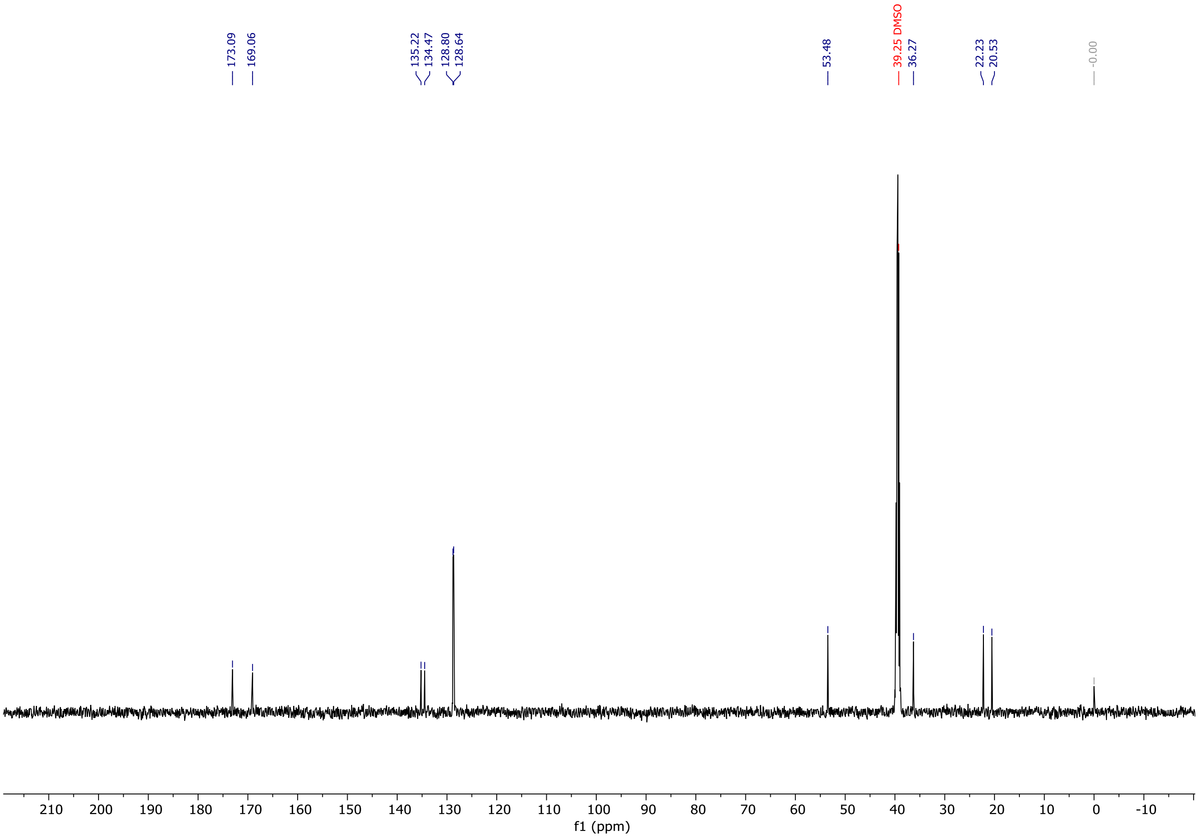

Figure S51: ^{13}C{^{1}H}-NMR in d_6-DMSO of (*S*)-2-acetamido-3-(p-tolyl)propanoic acid, **L19**

Methyl 2-(2-cyclopentylacetamido)acetate

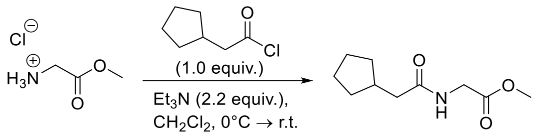

Methyl 2-aminoacetate hydrochloride (313 mg, 2.5 mmol) and dry CH_2Cl_2 (2.5 mL) were added in a round bottom flask and Et_3N (762 µL, 557 mg, 5.50 mmol, 2.2 equiv.) was added. The solution was stirred for 5 min at room temperature. 2-Cyclopentylacetyl chloride (337 µL, 367 mg, 2.50 mmol, 1.0 equiv.) was dissolved in dry CH_2Cl_2 (1.3 mL) and added dropwise at 0°C. The reaction was allowed to warm to room temperature and was stirred overnight. The reaction mixture was diluted with CH_2Cl_2 and washed with an HCl solution (aqueous, 1 M) and brine. The combined organic layers were dried over Na_2SO_4 and the solvent was removed under reduced pressure. The product was purified using flash chromatography (SiO_2, EtOAc in n-pentane 0%→100%) and was obtained as a colorless oil (426 mg, 86%).

^{1}H NMR (500 MHz, d_4-MeOH) δ = 4.78 (br. s, 1H), 3.92 (s, 2H), 3.71 (s, 3H), 2.33 – 2.13 (m, 3H), 1.91 – 1.78 (m, 2H), 1.70 – 1.61 (m, 2H), 1.61 – 1.53 (m, 2H), 1.25 – 1.17 (m, 2H) ppm.

^{13}C NMR (126 MHz, d_4-MeOH) δ = 176.1, 171.7, 52.5, 42.7, 41.7, 38.4, 33.3, 25.8 ppm.

HRMS (ESI pos) m/z: Calcd for $C_{10}H_{18}O_3N$ 200.12812, Found 200.12785.

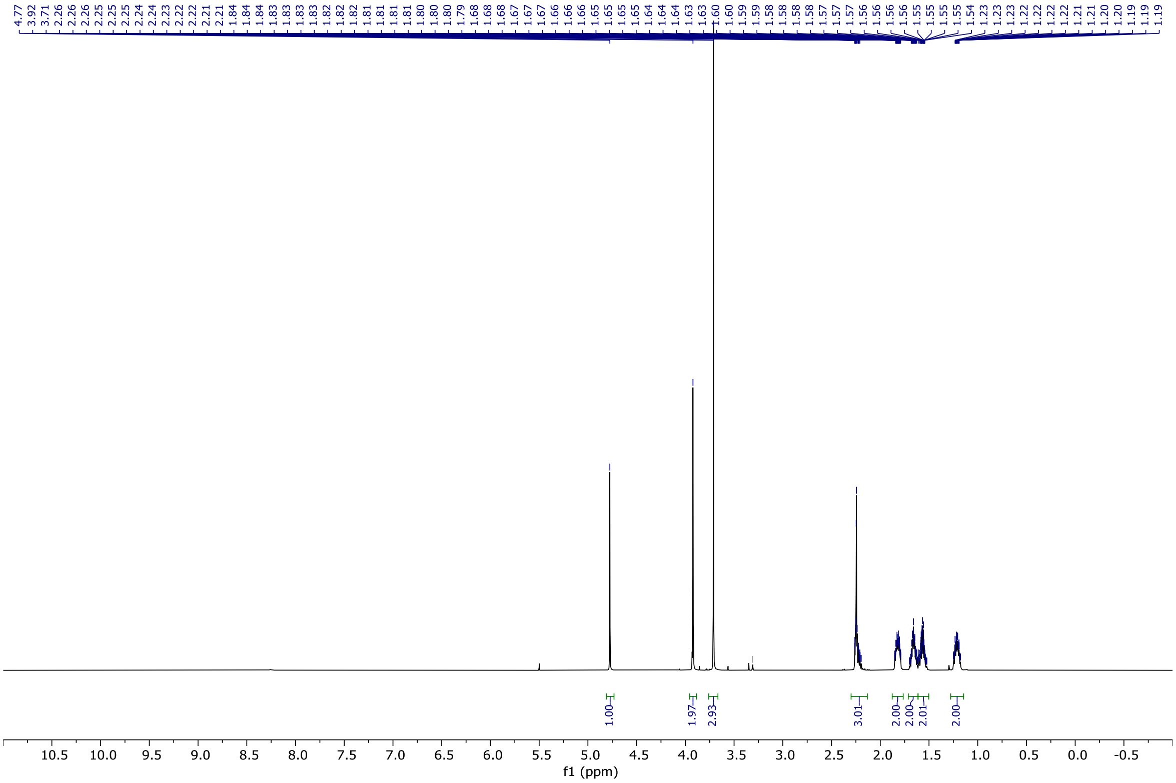

Figure S52: ^{1}H-NMR in d_4-MeOH of methyl 2-(2-cyclopentylacetamido)acetate.

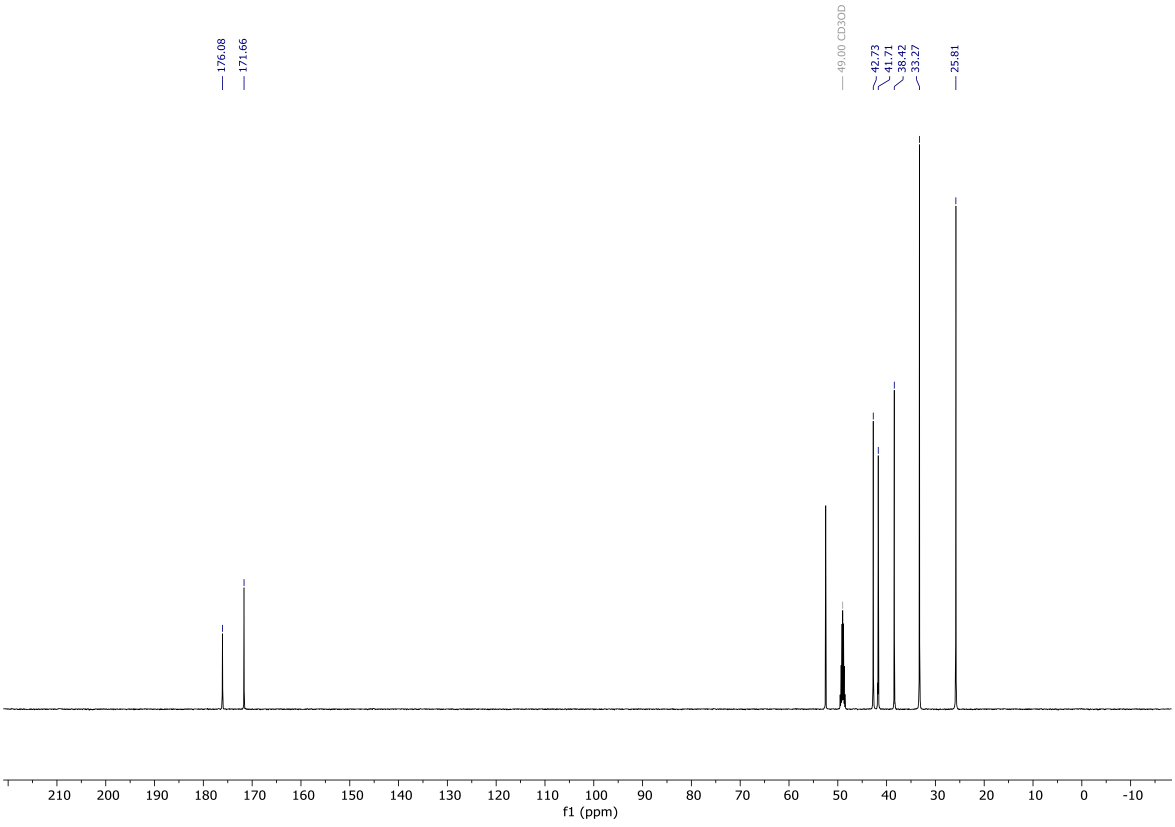

Figure S53: $^{13}C\{^{1}H\}$-NMR in d_4-MeOH of methyl 2-(2-cyclopentylacetamido)acetate.

2-(2-cyclopentylacetamido)acetic acid (L23)

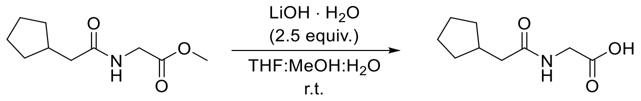

Methyl 2-(2-cyclopentylacetamido)acetate (152 mg, 0.764 mmol) was added in a round bottom flask with LiOH·H_2O (80.1 mg, 1.91 mmol, 2.5 equiv.) and a 1:1:1 mixture of MeOH:THF:H_2O (10 mL). After 4 h the reaction mixture was acidified with a solution of HCl (aqueous, 2 M) to pH 1-2 and was extracted with CH_2Cl_2. The combined organic layers were dried over Na_2SO_4, filtered through a short silica plug, and the solvent was removed under reduced pressure to yield the title compound as a colorless solid (142 mg, quant.).

^{1}H NMR (500 MHz, *d*$_4$-MeOH) δ = 4.90 (s, 2H), 3.89 (s, 2H), 2.26 – 2.18 (m, 3H), 1.87 – 1.78 (m, 2H), 1.71 – 1.62 (m, 2H), 1.61 – 1.53 (m, 2H), 1.31 – 1.15 (m, 2H) ppm.

^{13}C NMR (126 MHz, *d*$_4$-MeOH) δ = 176.3, 173.0, 42.9, 41.7, 38.5, 33.3, 25.9 ppm.

HRMS (ESI pos) m/z: Calcd for $C_9H_{16}NO_3$ 186.11247, Found 186.11242.

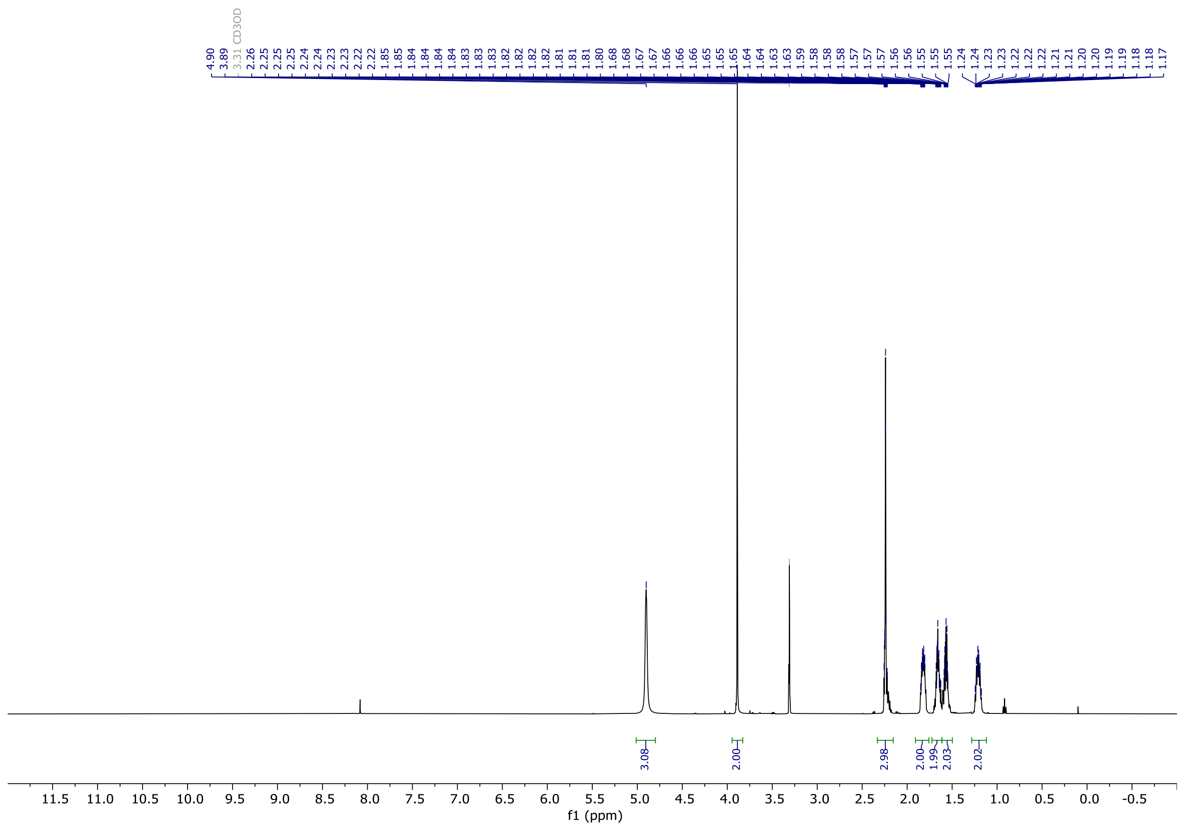

Figure S54: ^{1}H-NMR in d_4-MeOH of 2-(2-cyclopentylacetamido)acetic acid (**L23**).

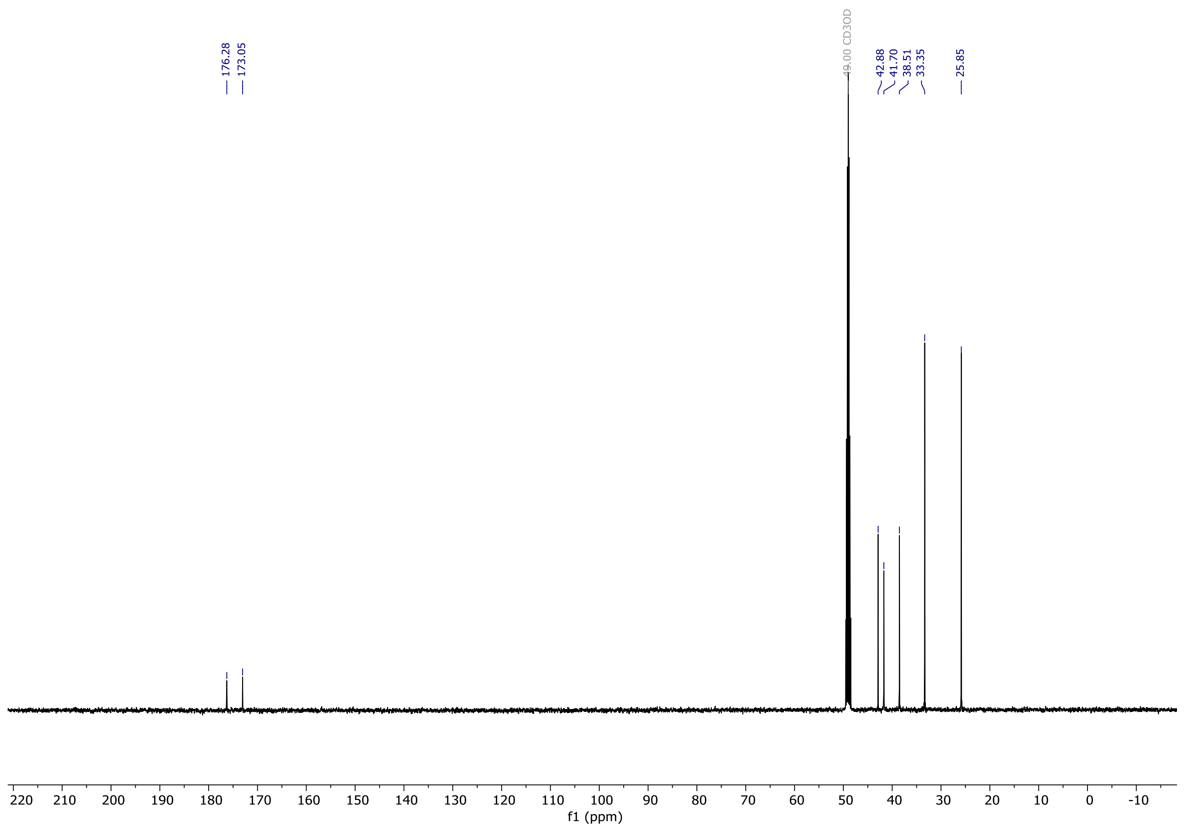

Figure S55: ^{13}C{^{1}H}-NMR in d_4-MeOH of 2-(2-cyclopentylacetamido)acetic acid (**L23**).

C–H Activation Set-Up

Please be referred to our previous report for more details (SI, section "custom set-up") regarding the standard handling of C–H activation reactions, preparation of GC samples, or analysis of deuteration degrees in our laboratory and to the original publication for the final conditions (see Figure S56).[115,116]

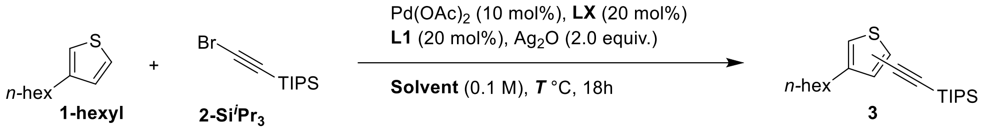

optimized C2 selective conditions

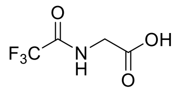

L16, MeOH, 30°C

	**Yield**	**Ratio (C2:C5)**
**As above**	**71%**	**87:13**
No Pd(OAc)2, L1, and pyrazine	0%	-
No L1	19%	61:39
No pyrazine	9%	45:55
As above*	61%	86:14
instead 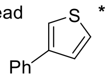 *	53%	89:11
instead Br—≡—^{t}Bu *	26%	83:17

optimized C5 selective conditions

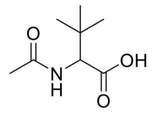

L5, Octanol, 40°C

	**Yield**	**Ratio (C2:C5)**
**As above**	**71%**	**6:94**
No Pd(OAc)2, L1, and pyrazine	0%	-
No L1	19%	47:53
No pyrazine	9%	33:67
As above*	70%	6:94
instead 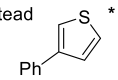 *	53%	7:93
instead Br—≡—^{t}Bu *	38%	5:95

* isolated yields and NMR ratios insted of GC-FID

Figure S56: Final reaction conditions as reported by Mondal and van Gemmeren[116] with the respective control experiments showing the importance of a dual-ligand catalytic system.

General procedure

An oven-dried Schlenk tube (10 mL) was charged with a magnetic stirring-bar. Then $Pd(OAc)_2$ (10 mol%), amino-acid derived ligand (30 mol%), and Ag_2O (2.0 equiv.) were added. Pyrazine (20 mol%) was added last due to volatility concerns. Half of the solvent (final c = 0.1 M) was added. (Bromoethynyl)triisopropylsilane (1.5 equiv.) and 3-hexyl thiophene were added followed by the remaining EtOH. The tube was then tightly sealed and was stirred on a pre-heated metal block at 40°C for 18h. The reaction mixture was allowed to cool to room temperature after completion.

4.2 Deuterium Kinetic Isotope Effect

$^1H/^2H$ parallel KIE

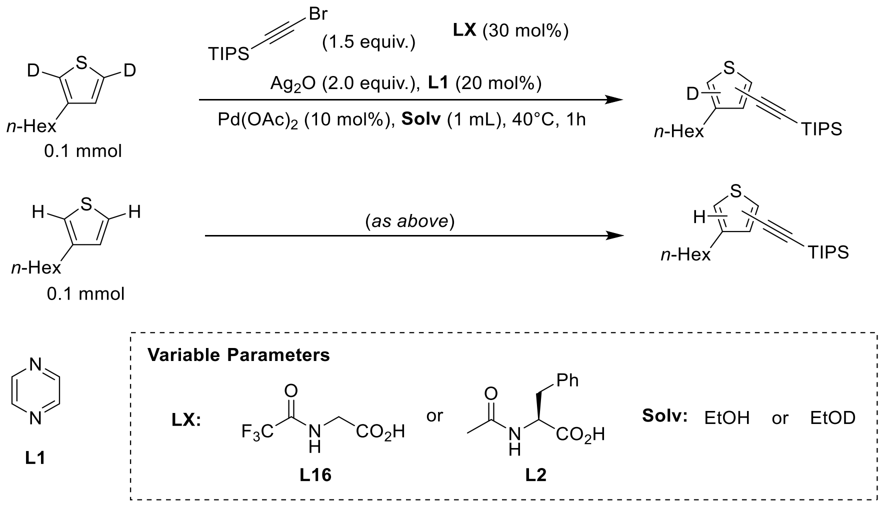

An oven-dried Schlenk tube (10 mL) was charged with a magnetic stirring-bar. Then $Pd(OAc)_2$ (2.3 mg, 0.010 mmol, 10 mol%), *N*-Acetyl-L-phenylalanine (6.2 mg, 0.030 mmol, 30 mol%) or 2-(2,2,2-trifluoroacetamido)acetic acid (5.1 mg, 0.030 mmol, 30 mol%), and Ag_2O (46.3 mg, 0.20 mmol, 2.0 equiv.) were added. Trimethyl 1,3,5-benzenetricarboxylate (~10 mg, 0.040 mmol, precise weight noted down per reaction) was added as internal standard. Pyrazine (1.6 mg, 0.020 mmol, 20 mol%) was added last due to volatility concerns. Half of the solvent (final c = 0.1 M) was added. (Bromoethynyl)triisopropylsilane (39 mg, 1.5 equiv.) and 3-hexyl thiophene (17 mg, 0.10 mmol) or 2,5-d_2-3-hexyl thiophene (17 mg, 0.10 mmol) were added followed by the remaining EtOH or EtOD. The tube was then tightly sealed with an inverted septum and parafilm and was stirred on a pre-heated metal block at 40°C for 1h. An aliquot (20 µL) was retrieved every 5 min for 1 h using a Hamilton syringe. The aliquot was filtered through a plug of silica and eluted with EtOAc. The resulting solution was submitted to GC-FID and optionally GC-MS analysis.

The reaction was carried out for **L16** and **L2** with deuterated and non-deuterated starting material as well as mono-deuterated and non-deuterated ethanol (see Table S17).

Table S17: Time and concentration values for the parallel KIE experiments with duplicates. An error of 2% was estimated on the concentration.

**L2**		**Run01**, d_0-3-hexylthiophene, EtOH; c / mol·L^{-1}					**L16**		**Run01**, d_0-3-hexylthiophene, EtOH; c / mol·L^{-1}				
t / min	C2+C5	σ(C2+C5)	C2	σ(C2)	C5	σ(C5)	t / min	C2+C5	σ(C2+C5)	C2	σ(C2)	C5	σ(C5)
6	4.532E-03	9.1E-05	3.120E-04	6.2E-06	4.22E-03	8.4E-05	5	3.608E-04	7.2E-06	2.355E-04	4.7E-06	1.254E-04	2.5E-06
10	6.564E-03	1.3E-04	5.214E-04	1.0E-05	6.04E-03	1.2E-04	10	1.206E-03	2.4E-05	8.660E-04	1.7E-05	3.403E-04	6.8E-06
15	8.457E-03	1.7E-04	6.374E-04	1.3E-05	7.82E-03	1.6E-04	15	2.208E-03	4.4E-05	1.664E-03	3.3E-05	5.445E-04	1.1E-05
20	9.942E-03	2.0E-04	7.695E-04	1.5E-05	9.17E-03	1.8E-04	20	3.098E-03	6.2E-05	2.382E-03	4.8E-05	7.167E-04	1.4E-05
25	1.110E-02	2.2E-04	8.689E-04	1.7E-05	1.02E-02	2.0E-04	25	4.004E-03	8.0E-05	3.111E-03	6.2E-05	8.928E-04	1.8E-05
30	1.224E-02	2.4E-04	9.576E-04	1.9E-05	1.13E-02	2.3E-04	30	4.929E-03	9.9E-05	3.857E-03	7.7E-05	1.072E-03	2.1E-05
35	1.331E-02	2.7E-04	1.037E-03	2.1E-05	1.23E-02	2.5E-04	35	5.721E-03	1.1E-04	4.493E-03	9.0E-05	1.228E-03	2.5E-05
40	1.407E-02	2.8E-04	1.101E-03	2.2E-05	1.30E-02	2.6E-04	40	6.465E-03	1.3E-04	5.091E-03	1.0E-04	1.374E-03	2.7E-05
45	1.492E-02	3.0E-04	1.178E-03	2.4E-05	1.37E-02	2.7E-04	45	7.421E-03	1.5E-04	5.861E-03	1.2E-04	1.560E-03	3.1E-05
50	1.573E-02	3.1E-04	1.219E-03	2.4E-05	1.45E-02	2.9E-04	50	8.018E-03	1.6E-04	6.337E-03	1.3E-04	1.682E-03	3.4E-05
55	1.639E-02	3.3E-04	1.272E-03	2.5E-05	1.51E-02	3.0E-04	55	8.622E-03	1.7E-04	6.823E-03	1.4E-04	1.799E-03	3.6E-05
60	1.693E-02	3.4E-04	1.316E-03	2.6E-05	1.56E-02	3.1E-04	60	9.308E-03	1.9E-04	7.371E-03	1.5E-04	1.937E-03	3.9E-05

**L2**		**Run02**, d_0-3-hexylthiophene, EtOH; c / mol·L^{-1}					**L16**		**Run02**, d_0-3-hexylthiophene, EtOH; c / mol·L^{-1}				
t / min	C2+C5	σ(C2+C5)	C2	σ(C2)	C5	σ(C5)	t / min	C2+C5	σ(C2+C5)	C2	σ(C2)	C5	σ(C5)
6	3.609E-03	7.2E-05	2.833E-04	5.7E-06	3.326E-03	6.7E-05	5	4.387E-04	8.8E-06	2.861E-04	5.7E-06	1.527E-04	3.1E-06
10	5.671E-03	1.1E-04	4.104E-04	8.2E-06	5.260E-03	1.1E-04	10	1.486E-03	3.0E-05	1.059E-03	2.1E-05	4.271E-04	8.5E-06
15	7.456E-03	1.5E-04	5.754E-04	1.2E-05	6.880E-03	1.4E-04	15	2.507E-03	5.0E-05	1.893E-03	3.8E-05	6.144E-04	1.2E-05
20	8.490E-03	1.7E-04	6.403E-04	1.3E-05	7.849E-03	1.6E-04	20	3.676E-03	7.4E-05	2.828E-03	5.7E-05	8.479E-04	1.7E-05
25	9.572E-03	1.9E-04	7.505E-04	1.5E-05	8.821E-03	1.8E-04	25	4.861E-03	9.7E-05	3.761E-03	7.5E-05	1.100E-03	2.2E-05
30	1.051E-02	2.1E-04	8.188E-04	1.6E-05	9.694E-03	1.9E-04	30	5.789E-03	1.2E-04	4.508E-03	9.0E-05	1.281E-03	2.6E-05
35	1.146E-02	2.3E-04	8.799E-04	1.8E-05	1.058E-02	2.1E-04	35	6.675E-03	1.3E-04	5.249E-03	1.0E-04	1.425E-03	2.9E-05
40	1.214E-02	2.4E-04	9.433E-04	1.9E-05	1.119E-02	2.2E-04	40	7.520E-03	1.5E-04	5.928E-03	1.2E-04	1.592E-03	3.2E-05
45	1.305E-02	2.6E-04	9.995E-04	2.0E-05	1.205E-02	2.4E-04	45	8.546E-03	1.7E-04	6.756E-03	1.4E-04	1.790E-03	3.6E-05
50	1.362E-02	2.7E-04	1.040E-03	2.1E-05	1.258E-02	2.5E-04	50	9.504E-03	1.9E-04	7.473E-03	1.5E-04	2.031E-03	4.1E-05
55	1.406E-02	2.8E-04	1.079E-03	2.2E-05	1.299E-02	2.6E-04	55	1.011E-02	2.0E-04	8.002E-03	1.6E-04	2.104E-03	4.2E-05
60	1.477E-02	3.0E-04	1.127E-03	2.3E-05	1.364E-02	2.7E-04	60	1.086E-02	2.2E-04	8.561E-03	1.7E-04	2.295E-03	4.6E-05

**L2**		**Run03**, 2,5-d_2-3-hexylthiophene, EtOH; c / mol·L^{-1}					**L16**		**Run03**, 2,5-d_2-3-hexylthiophene, EtOH; c / mol·L^{-1}				
t / min	C2+C5	σ(C2+C5)	C2	σ(C2)	C5	σ(C5)	t / min	C2+C5	σ(C2+C5)	C2	σ(C2)	C5	σ(C5)
5	2.156E-03	4.3E-05	1.763E-04	3.5E-06	1.979E-03	4.0E-05	5	2.750E-04	5.5E-06	1.974E-04	3.9E-06	7.757E-05	1.6E-06
10	3.621E-03	7.2E-05	3.221E-04	6.4E-06	3.299E-03	6.6E-05	10	5.264E-04	1.1E-05	3.625E-04	7.3E-06	1.639E-04	3.3E-06
15	4.715E-03	9.4E-05	4.957E-04	9.9E-06	4.219E-03	8.4E-05	15	1.015E-03	2.0E-05	7.467E-04	1.5E-05	2.680E-04	5.4E-06
20	5.628E-03	1.1E-04	5.871E-04	1.2E-05	5.041E-03	1.0E-04	20	1.555E-03	3.1E-05	1.185E-03	2.4E-05	3.700E-04	7.4E-06
25	6.344E-03	1.3E-04	6.578E-04	1.3E-05	5.687E-03	1.1E-04	25	2.108E-03	4.2E-05	1.639E-03	3.3E-05	4.689E-04	9.4E-06
30	7.083E-03	1.4E-04	7.487E-04	1.5E-05	6.335E-03	1.3E-04	30	2.625E-03	5.2E-05	2.061E-03	4.1E-05	5.636E-04	1.1E-05
35	7.763E-03	1.6E-04	8.434E-04	1.7E-05	6.920E-03	1.4E-04	35	3.137E-03	6.3E-05	2.480E-03	5.0E-05	6.568E-04	1.3E-05
40	8.418E-03	1.7E-04	9.122E-04	1.8E-05	7.506E-03	1.5E-04	40	3.625E-03	7.2E-05	2.882E-03	5.8E-05	7.428E-04	1.5E-05
45	9.036E-03	1.8E-04	9.982E-04	2.0E-05	8.037E-03	1.6E-04	45	4.084E-03	8.2E-05	3.261E-03	6.5E-05	8.224E-04	1.6E-05
50	9.509E-03	1.9E-04	1.062E-03	2.1E-05	8.447E-03	1.7E-04	50	4.584E-03	9.2E-05	3.669E-03	7.3E-05	9.146E-04	1.8E-05
55	1.003E-02	2.0E-04	1.143E-03	2.3E-05	8.883E-03	1.8E-04	55	4.966E-03	9.9E-05	3.986E-03	8.0E-05	9.798E-04	2.0E-05
60	1.053E-02	2.1E-04	1.232E-03	2.5E-05	9.302E-03	1.9E-04	60	5.353E-03	1.1E-04	4.308E-03	8.6E-05	1.045E-03	2.1E-05

**L2**		**Run04**, 2,5-d_2-3-hexylthiophene, EtOH; c / mol·L^{-1}					**L16**		**Run04**, 2,5-d_2-3-hexylthiophene, EtOH; c / mol·L^{-1}				
t / min	C2+C5	σ(C2+C5)	C2	σ(C2)	C5	σ(C5)	t / min	C2+C5	σ(C2+C5)	C2	σ(C2)	C5	σ(C5)
5	1.710E-03	3.4E-05	1.620E-04	3.2E-06	1.548E-03	3.1E-05	5	1.845E-04	3.7E-06	1.219E-04	2.4E-06	6.261E-05	1.3E-06
11	3.065E-03	6.1E-05	2.938E-04	5.9E-06	2.771E-03	5.5E-05	10	6.815E-04	1.4E-05	4.888E-04	9.8E-06	1.926E-04	3.9E-06
15	3.681E-03	7.4E-05	3.694E-04	7.4E-06	3.311E-03	6.6E-05	15	1.233E-03	2.5E-05	9.227E-04	1.8E-05	3.106E-04	6.2E-06
20	4.328E-03	8.7E-05	4.526E-04	9.1E-06	3.875E-03	7.8E-05	20	1.923E-03	3.8E-05	1.488E-03	3.0E-05	4.348E-04	8.7E-06
25	4.911E-03	9.8E-05	5.359E-04	1.1E-05	4.375E-03	8.7E-05	25	2.430E-03	4.9E-05	1.899E-03	3.8E-05	5.312E-04	1.1E-05
30	5.469E-03	1.1E-04	6.322E-04	1.3E-05	4.837E-03	9.7E-05	30	3.001E-03	6.0E-05	2.369E-03	4.7E-05	6.314E-04	1.3E-05
35	5.937E-03	1.2E-04	6.679E-04	1.3E-05	5.269E-03	1.1E-04	35	3.555E-03	7.1E-05	2.830E-03	5.7E-05	7.247E-04	1.4E-05
40	6.347E-03	1.3E-04	7.100E-04	1.4E-05	5.637E-03	1.1E-04	40	4.138E-03	8.3E-05	3.304E-03	6.6E-05	8.339E-04	1.7E-05
45	6.742E-03	1.3E-04	7.506E-04	1.5E-05	5.991E-03	1.2E-04	45	4.627E-03	9.3E-05	3.706E-03	7.4E-05	9.209E-04	1.8E-05
50	7.189E-03	1.4E-04	8.193E-04	1.6E-05	6.370E-03	1.3E-04	50	5.151E-03	1.0E-04	4.142E-03	8.3E-05	1.009E-03	2.0E-05
55	7.521E-03	1.5E-04	8.559E-04	1.7E-05	6.665E-03	1.3E-04	55	5.591E-03	1.1E-04	4.503E-03	9.0E-05	1.088E-03	2.2E-05
60	7.838E-03	1.6E-04	8.893E-04	1.8E-05	6.948E-03	1.4E-04	60	5.909E-03	1.2E-04	4.773E-03	9.5E-05	1.136E-03	2.3E-05

**L2**		**Run05**, 2,5-d_2-3-hexylthiophene, EtOD; c / mol·L^{-1}					**L16**		**Run05**, 2,5-d_2-3-hexylthiophene, EtOD; c / mol·L^{-1}				
t / min	C2+C5	σ(C2+C5)	C2	σ(C2)	C5	σ(C5)	t / min	C2+C5	σ(C2+C5)	C2	σ(C2)	C5	σ(C5)
5	1.889E-03	3.8E-05	2.862E-04	5.7E-06	1.603E-03	3.2E-05	5	2.362E-04	4.7E-06	1.651E-04	3.3E-06	7.107E-05	1.4E-06
10	3.341E-03	6.7E-05	4.734E-04	9.5E-06	2.867E-03	5.7E-05	10	8.238E-04	1.6E-05	6.299E-04	1.3E-05	1.939E-04	3.9E-06
15	4.609E-03	9.2E-05	7.888E-04	1.6E-05	3.820E-03	7.6E-05	15	1.536E-03	3.1E-05	1.221E-03	2.4E-05	3.148E-04	6.3E-06
20	5.539E-03	1.1E-04	9.249E-04	1.8E-05	4.614E-03	9.2E-05	20	2.226E-03	4.5E-05	1.798E-03	3.6E-05	4.281E-04	8.6E-06
25	6.216E-03	1.2E-04	1.073E-03	2.1E-05	5.142E-03	1.0E-04	25	2.931E-03	5.9E-05	2.397E-03	4.8E-05	5.335E-04	1.1E-05
30	6.901E-03	1.4E-04	1.198E-03	2.4E-05	5.703E-03	1.1E-04	30	3.625E-03	7.3E-05	2.959E-03	5.9E-05	6.658E-04	1.3E-05
35	7.640E-03	1.5E-04	1.372E-03	2.7E-05	6.269E-03	1.3E-04	35	4.256E-03	8.5E-05	3.507E-03	7.0E-05	7.486E-04	1.5E-05
40	8.237E-03	1.6E-04	1.462E-03	2.9E-05	6.775E-03	1.4E-04	40	4.821E-03	9.6E-05	3.984E-03	8.0E-05	8.369E-04	1.7E-05
45	8.806E-03	1.8E-04	1.529E-03	3.1E-05	7.277E-03	1.5E-04	45	5.437E-03	1.1E-04	4.515E-03	9.0E-05	9.228E-04	1.8E-05
50	9.222E-03	1.8E-04	1.624E-03	3.2E-05	7.598E-03	1.5E-04	50	5.918E-03	1.2E-04	4.911E-03	9.8E-05	1.007E-03	2.0E-05
55	9.752E-03	2.0E-04	1.693E-03	3.4E-05	8.059E-03	1.6E-04	55	6.350E-03	1.3E-04	5.274E-03	1.1E-04	1.076E-03	2.2E-05
60	1.029E-02	2.1E-04	1.795E-03	3.6E-05	8.491E-03	1.7E-04	60	6.802E-03	1.4E-04	5.656E-03	1.1E-04	1.147E-03	2.3E-05

**L2**		**Run06**, 2,5-d_2-3-hexylthiophene, EtOD; c / mol·L^{-1}					**L16**		**Run06**, 2,5-d_2-3-hexylthiophene, EtOD; c / mol·L^{-1}				
t / min	C2+C5	σ(C2+C5)	C2	σ(C2)	C5	σ(C5)	t / min	C2+C5	σ(C2+C5)	C2	σ(C2)	C5	σ(C5)
5	2.042E-03	4.1E-05	3.138E-04	6.3E-06	1.729E-03	3.5E-05	5	1.690E-04	3.4E-06	1.143E-04	2.3E-06	5.469E-05	1.1E-06
10	3.394E-03	6.8E-05	5.605E-04	1.1E-05	2.834E-03	5.7E-05	10	6.412E-04	1.3E-05	4.691E-04	9.4E-06	1.721E-04	3.4E-06

15	4.471E-03	8.9E-05	7.165E-04	1.4E-05	3.754E-03	7.5E-05	15	1.307E-03	2.6E-05	1.020E-03	2.0E-05	2.870E-04	5.7E-06
20	5.326E-03	1.1E-04	9.084E-04	1.8E-05	4.417E-03	8.8E-05	20	2.228E-03	4.5E-05	1.812E-03	3.6E-05	4.159E-04	8.3E-06
25	6.102E-03	1.2E-04	1.059E-03	2.1E-05	5.044E-03	1.0E-04	25	2.664E-03	5.3E-05	2.161E-03	4.3E-05	5.027E-04	1.0E-05
30	6.877E-03	1.4E-04	1.201E-03	2.4E-05	5.676E-03	1.1E-04	30	3.347E-03	6.7E-05	2.744E-03	5.5E-05	6.028E-04	1.2E-05
35	7.430E-03	1.5E-04	1.336E-03	2.7E-05	6.094E-03	1.2E-04	35	3.891E-03	7.8E-05	3.199E-03	6.4E-05	6.922E-04	1.4E-05
40	8.061E-03	1.6E-04	1.451E-03	2.9E-05	6.610E-03	1.3E-04	40	4.475E-03	8.9E-05	3.682E-03	7.4E-05	7.931E-04	1.6E-05
45	8.563E-03	1.7E-04	1.509E-03	3.0E-05	7.054E-03	1.4E-04	45	5.005E-03	1.0E-04	4.139E-03	8.3E-05	8.652E-04	1.7E-05
50	9.103E-03	1.8E-04	1.615E-03	3.2E-05	7.488E-03	1.5E-04	50	5.511E-03	1.1E-04	4.555E-03	9.1E-05	9.566E-04	1.9E-05
55	9.663E-03	1.9E-04	1.730E-03	3.5E-05	7.933E-03	1.6E-04	55	5.971E-03	1.2E-04	4.941E-03	9.9E-05	1.030E-03	2.1E-05
60	1.010E-02	2.0E-04	1.809E-03	3.6E-05	8.287E-03	1.7E-04	60	6.465E-03	1.3E-04	5.362E-03	1.1E-04	1.104E-03	2.2E-05
**L2**		**Run07**, d_0-3-hexylthiophene, EtOD; *c* / mol·L^{-1}					**L16**		**Run07**, d_0-3-hexylthiophene, EtOD; *c* / mol·L^{-1}				
t / min	C2+C5	σ(C2+C5)	C2	σ(C2)	C5	σ(C5)	*t* / min	C2+C5	σ(C2+C5)	C2	σ(C2)	C5	σ(C5)
5	3.792E-03	7.6E-05	5.341E-04	1.1E-05	3.258E-03	6.5E-05	5	8.370E-04	1.7E-05	5.781E-04	1.2E-05	2.589E-04	5.2E-06
10	6.189E-03	1.2E-04	8.689E-04	1.7E-05	5.320E-03	1.1E-04	10	2.271E-03	4.5E-05	1.736E-03	3.5E-05	5.346E-04	1.1E-05
15	7.536E-03	1.5E-04	1.019E-03	2.0E-05	6.517E-03	1.3E-04	15	3.771E-03	7.5E-05	2.961E-03	5.9E-05	8.099E-04	1.6E-05
20	8.715E-03	1.7E-04	1.143E-03	2.3E-05	7.572E-03	1.5E-04	20	5.190E-03	1.0E-04	4.120E-03	8.2E-05	1.070E-03	2.1E-05
25	9.841E-03	2.0E-04	1.294E-03	2.6E-05	8.547E-03	1.7E-04	25	6.417E-03	1.3E-04	5.133E-03	1.0E-04	1.284E-03	2.6E-05
30	1.096E-02	2.2E-04	1.406E-03	2.8E-05	9.556E-03	1.9E-04	30	7.669E-03	1.5E-04	6.155E-03	1.2E-04	1.514E-03	3.0E-05
35	1.177E-02	2.4E-04	1.500E-03	3.0E-05	1.027E-02	2.1E-04	35	7.658E-03	1.5E-04	6.184E-03	1.2E-04	1.473E-03	2.9E-05
40	1.257E-02	2.5E-04	1.578E-03	3.2E-05	1.100E-02	2.2E-04	40	1.000E-02	2.0E-04	8.058E-03	1.6E-04	1.947E-03	3.9E-05
45	1.335E-02	2.7E-04	1.661E-03	3.3E-05	1.169E-02	2.3E-04	45	1.101E-02	2.2E-04	8.882E-03	1.8E-04	2.132E-03	4.3E-05
50	1.393E-02	2.8E-04	1.721E-03	3.4E-05	1.221E-02	2.4E-04	50	1.193E-02	2.4E-04	9.623E-03	1.9E-04	2.304E-03	4.6E-05
55	1.461E-02	2.9E-04	1.794E-03	3.6E-05	1.282E-02	2.6E-04	55	1.325E-02	2.7E-04	1.069E-02	2.1E-04	2.560E-03	5.1E-05
60	1.527E-02	3.1E-04	1.848E-03	3.7E-05	1.342E-02	2.7E-04	60	1.360E-02	2.7E-04	1.099E-02	2.2E-04	2.607E-03	5.2E-05
**L2**		**Run08**, d_0-3-hexylthiophene, EtOD; *c* / mol·L^{-1}					**L16**		**Run08**, d_0-3-hexylthiophene, EtOD; *c* / mol·L^{-1}				
t / min	C2+C5	σ(C2+C5)	C2	σ(C2)	C5	σ(C5)	*t* / min	C2+C5	σ(C2+C5)	C2	σ(C2)	C5	σ(C5)
5	3.501E-03	7.0E-05	5.065E-04	1.0E-05	2.994E-03	6.0E-05	5	5.940E-04	1.2E-05	4.184E-04	8.4E-06	1.756E-04	3.5E-06
10	6.194E-03	1.2E-04	8.406E-04	1.7E-05	5.353E-03	1.1E-04	10	1.732E-03	3.5E-05	1.311E-03	2.6E-05	4.210E-04	8.4E-06
15	8.055E-03	1.6E-04	1.040E-03	2.1E-05	7.015E-03	1.4E-04	15	3.097E-03	6.2E-05	2.434E-03	4.9E-05	6.628E-04	1.3E-05
20	9.343E-03	1.9E-04	1.182E-03	2.4E-05	8.161E-03	1.6E-04	20	4.251E-03	8.5E-05	3.391E-03	6.8E-05	8.603E-04	1.7E-05
25	1.048E-02	2.1E-04	1.320E-03	2.6E-05	9.160E-03	1.8E-04	25	5.417E-03	1.1E-04	4.355E-03	8.7E-05	1.063E-03	2.1E-05
30	1.146E-02	2.3E-04	1.429E-03	2.9E-05	1.003E-02	2.0E-04	30	6.627E-03	1.3E-04	5.346E-03	1.1E-04	1.282E-03	2.6E-05
35	1.249E-02	2.5E-04	1.542E-03	3.1E-05	1.095E-02	2.2E-04	35	7.684E-03	1.5E-04	6.217E-03	1.2E-04	1.467E-03	2.9E-05
40	1.335E-02	2.7E-04	1.633E-03	3.3E-05	1.172E-02	2.3E-04	40	8.568E-03	1.7E-04	6.941E-03	1.4E-04	1.627E-03	3.3E-05
45	1.424E-02	2.8E-04	1.726E-03	3.5E-05	1.252E-02	2.5E-04	45	9.545E-03	1.9E-04	7.743E-03	1.5E-04	1.802E-03	3.6E-05
50	1.502E-02	3.0E-04	1.807E-03	3.6E-05	1.321E-02	2.6E-04	50	1.032E-02	2.1E-04	8.369E-03	1.7E-04	1.953E-03	3.9E-05
55	1.560E-02	3.1E-04	1.866E-03	3.7E-05	1.373E-02	2.7E-04	55	1.116E-02	2.2E-04	9.060E-03	1.8E-04	2.096E-03	4.2E-05
60	1.630E-02	3.3E-04	1.936E-03	3.9E-05	1.437E-02	2.9E-04	60	1.200E-02	2.4E-04	9.746E-03	1.9E-04	2.254E-03	4.5E-05

All runs were repeated twice and the values were averaged for the subsequent linear regression analysis (Table S18, Figure S57).

Table S18: Rates derived from linear regression of datapoints averaged from two runs.

		L2	*k* / mol·L^{-1}·min^{-1}	σ_k / mol·L^{-1}·min^{-1}	R²	L16	*k* / mol·L^{-1}·min^{-1}	σ_k / mol·L^{-1}·min^{-1}	R²
Run01,02									
5 - 60 min	C2+C5		2.39883E-4	1.66037E-5	0.95428		1.89098E-4	2.64251E-6	0.99805
5 - 25 min	C2+C5		3.52404E-4	3.35268E-5	0.97356		1.97381E-4	2.60341E-6	0.99948
5 - 60 min	C2		1.90975E-5	1.39357E-6	0.94944		1.49473E-4	2.00165E-6	0.99821
5 - 25 min	C2		2.85268E-5	2.63731E-6	0.97500		1.52716E-4	4.01825E-6	0.99793
5 - 60 min	C5		2.20791E-4	1.52124E-5	0.95468		3.90755E-5	9.99715E-7	0.99350
5 - 25 min	C5		3.23872E-4	3.09176E-5	0.97339		4.37942E-5	1.23506E-6	0.99762
Run03,04									
5 - 60 min	C2+C5		1.4551E-4	8.47869E-6	0.96716		9.97233E-5	2.53584E-6	0.99358
5 - 25 min	C2+C5		1.98573E-4	1.50681E-5	0.98302		9.30805E-5	6.44784E-6	0.98581
5 - 60 min	C2		1.79125E-5	8.74945E-7	0.97437		7.86071E-5	2.86331E-6	0.98691
5 - 25 min	C2		2.30331E-5	1.28250E-6	0.99078		7.04375E-5	6.54491E-6	0.97475
5 - 60 min	C5		1.27578E-4	7.60601E-6	0.96568		2.02832E-5	3.63084E-7	0.99681
5 - 25 min	C5		1.75454E-4	1.38914E-5	0.98154		2.18054E-5	1.69428E-7	0.99982
Run05,06									
5 - 60 min	C2+C5		1.65885E-4	9.51794E-6	0.96813		1.23992E-4	2.3496E-6	0.99642
5 - 25 min	C2+C5		2.25743E-4	1.53665E-5	0.98629		1.23741E-4	6.38875E-6	0.99207
5 - 60 min	C2		3.09002E-5	1.64417E-6	0.97247		1.02051E-4	2.38298E-6	0.99458
5 - 25 min	C2		4.06198E-5	1.79496E-6	0.99418		9.93733E-5	6.62885E-6	0.98683
5 - 60 min	C5		1.34970E-4	7.88849E-6	0.96697		2.13466E-5	5.08345E-7	0.99436
5 - 25 min	C5		1.84987E-4	1.36886E-5	0.98384		2.35121E-5	3.3269E-7	0.9994
Run07,08									
5 - 60 min	C2+C5		2.43510E-4	1.67468E-5	0.95484		2.41153E-4	5.26441E-6	0.99526
5 - 25 min	C2+C5		3.50747E-4	3.43802E-5	0.97198		2.64396E-4	3.36922E-6	0.99951
5 - 60 min	C2		2.73652E-5	2.19777E-6	0.93941		1.97088E-4	4.12186E-6	0.99565
5 - 25 min	C2		4.18933E-5	4.89855E-6	0.96060		2.14313E-4	3.13343E-6	0.99936
5 - 60 min	C5		2.16127E-4	1.45446E-5	0.95667		4.39087E-5	1.15161E-6	0.99317
5 - 25 min	C5		3.08822E-4	2.94849E-5	0.97338		4.96811E-5	1.11344E-6	0.9985

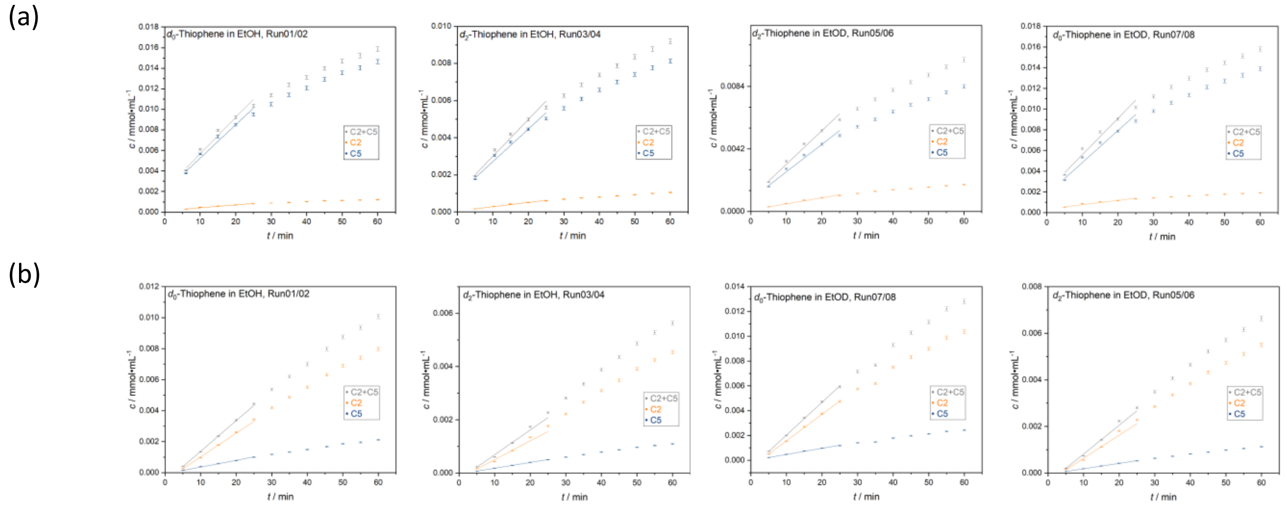

Figure S57: Overview of the concentration vs time plots used for deriving initial rates for (a) **L2** and (b) **L16**.

Since a substantial background reaction was suspected due to exchange with the solvent in the presence of catalyst and silver source, we opted for carrying out the reaction in deuterated solvent. Carrying out the reaction with deuterated substrate in non-deuterated solvent would likely lead to an underestimation of the KIE: since parts of the deuterated starting material would react to non-deuterated starting material that could preferentially react (assuming a normal KIE, $k_H > k_D$). It thus makes sense to determine k_D in deuterated solvent and with deuterated starting material. The reference reaction (k_H) could be done either in non-deuterated solvent with the advantage of no isotope exchange or in deuterated solvent with the advantage of having identical solvent conditions. Since we know from the original study that the identity of the solvent has an impact on the reaction outcome, we argued that using non-deuterated starting material in deuterated solvent is the ideal combination, since a potential deuteration as side reaction does not pose a major problem – since initial rates are considered, a major amount of non-deuterated product and starting material will still be present at the end of the reaction (as evidenced by GC-MS). Deuterated starting material is expected to react slower and hence has a minor influence on the rate compared to the excess of faster reacting non-deuterated starting material.

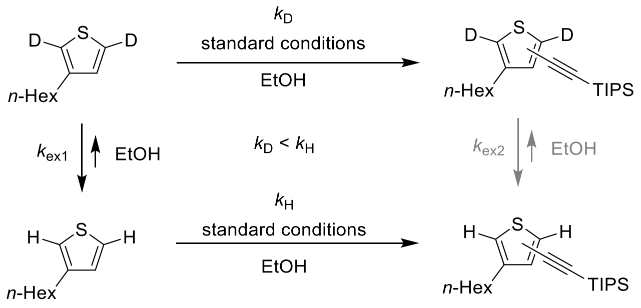

Figure S58: Complications of the KIE determination in a protic solvent.

Different permutations of the final KIE determination are reported for completeness's sake (k_H and k_D in EtOH, k_H in EtOH, k_D in EtOD and the described k_H and k_D in EtOD) that all support the main finding of KIE (**L2**) < KIE (**L16**) as shown in Table S19. Please note that larger deviations are suspected for the minor isomer of each respective ligand due to GC-FID detection limits.

Table S19: Parallel kinetic isotope effects in different experimental permutations.

**L2**		KIE					
k_H	k_D	C2+C5	σ_{C2+C5}	C2	σ_{C25}	C5	σ_{C5}
d_0-SM, EtOH	d_2-SM, EtOH	1.77	0.22	1.24	0.13	1.85	0.23
d_0-SM, EtOH	d_2-SM, EtOD	1.56	0.18	0.92	0.10	1.75	0.21
**d_0-SM, EtOD**	**d_2-SM, EtOD**	**1.55**	**0.19**	**1.36**	**0.17**	**1.67**	**0.20**
d_0-SM, EtOH	d_0-SM, EtOD	1.00	0.14	0.68	0.10	1.05	0.14
**L16**		KIE					
k_H	k_D	C2+C5	σ_{C2+C5}	C2	σ_{C25}	C5	σ_{C5}
d_0-SM, EtOH	d_2-SM, EtOH	2.12	0.15	2.17	0.21	2.01	0.06
d_0-SM, EtOH	d_2-SM, EtOD	1.60	0.11	1.54	0.15	1.86	0.05
**d_0-SM, EtOD**	**d_2-SM, EtOD**	**2.14**	**0.11**	**2.16**	**0.15**	**2.11**	**0.06**
d_0-SM, EtOH	d_0-SM, EtOD	0.75	0.01	0.71	0.02	0.88	0.03

An inverse solvent isotope effect could be observed especially for **L16** leading to a faster reaction in deuterated solvent compared to non-deuterated solvent. This is in line with an intermediate coordination of EtOH prior to the C–H activation as discussed above. The preceding C–H activation step itself is expected to have a KIE of 1. The overall magnitude of the solvent isotope effect hence depends on the magnitude of the intrinsic magnitude of the solvent KIE and the barrier height of the turnover limiting step. A higher barrier is expected to give a value closer to 1 which explains why C2 has a smaller solvent isotope effect compared to C5.

Comparison of ratios

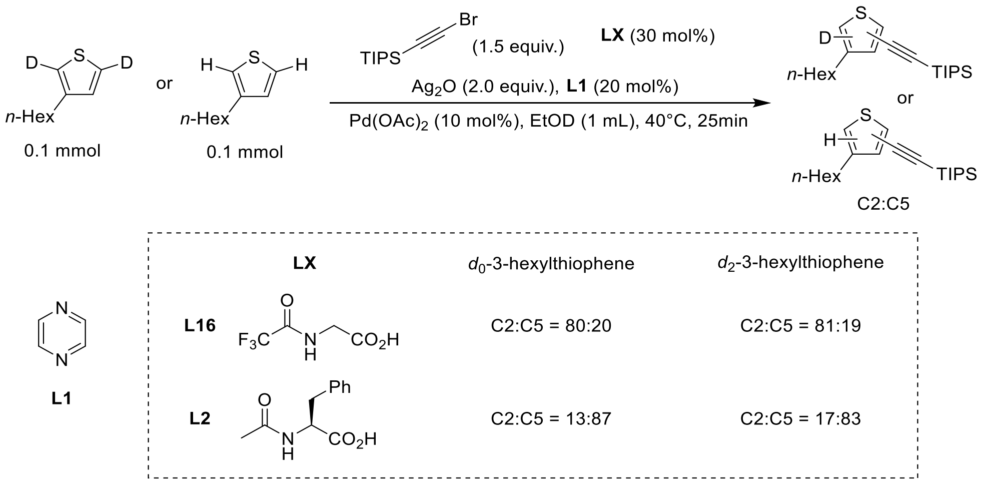

Figure S59: Comparison of ratios for deuterated and non-deuterated starting material after 25 min.

A further qualitative conclusion can be drawn by comparing the C2:C5 ratios of the respective reaction after 25 min in EtOD (see Figure S59). In case of **L16** the reaction with non-deuterated starting material yields a ratio of C2:C5 = 80:20 whereas the deuterated starting materials gives a ratio of C2:C5 = 81:19. The ratio is virtually unchanged within the experimental error, which would be expected from a reaction with a selectivity

determining C–H activation step. An even higher barrier of a potential C–D activation should not alter the ratios.[5]

For **L2** the ratios of C2:C5 for non-deuterated vs deuterated arene are 13:87 vs 17:83. Here the ratio is actively altered. Since the C–H activation step is proposed to be C2 selective and both, the C–H activation and C5-selective MI are proposed to be close in energy, an increased barrier of the C–D activation can directly influence that ratio by increasing the relative amount of C2 product.

Deuteration of SM in the absence of alkyne

To investigate the selectivity in the C–H activation step we carried out a deuteration of **1**-hexyl in EtOD to see the positional incorporation of deuterium (see Figure S60). We have outlined in detail how to determine %D, D_{Tot} by NMR (see Figure S61) in a previous publication.[115] A higher relative %D of C2 vs C5 is indicative of a lower C–H activation barrier since the reversible C–H activation and therefore the isotope exchange proceeds more readily in this position.

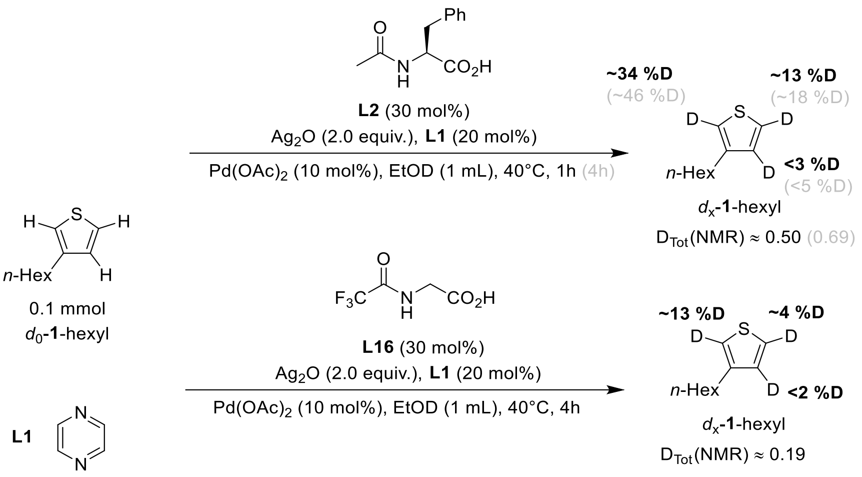

Figure S60: Investigation of positional deuteration of **1**-hexyl in EtOD with **L2** and **L16** without alkyne coupling partner using crude NMR.

We see a higher degree of deuterium incorporation in the C2 position for **L2** and **L16**, as computationally predicted, indicating that the C–H activation step is C2 selective even for the overall C5 selective ligand **L2**. This experiment points further towards our mechanistic hypothesis of a C2-selective C–H activation and a Curtin-Hammett scenario with a C5-selective subsequent step.

The reaction with **L16** was stopped after 4h instead of 1h, since at 1h the deuterium incorporation was too low to reliably determine the %D ratios.[6] The lower reactivity of **L16** also indicates a higher barrier for C–H activation for **L16** vs **L2** as suggested before.

[5] Unless the relative barriers $\Delta\Delta G^{‡}$(C2 vs C5) were significantly affected which is highly unlikely due to the isomeric nature of **TS-4**

[6] Please note that the %D ratios are only qualitatively indicative of the selectivity of the C–H activation step. Over the course of the reaction a mixture of d_1-**1**-hexyl and d_2-**1**-hexyl forms, since mono-deuterated **1** can react further. This distorts the observed C2 vs C5 selectivity at longer reaction times, while at low %D the relative error on integration of the NMR spectra is larger. Consequently, no quantitative data on the relative barriers for C2 and C5 C–H activation can be derived from this experiment.

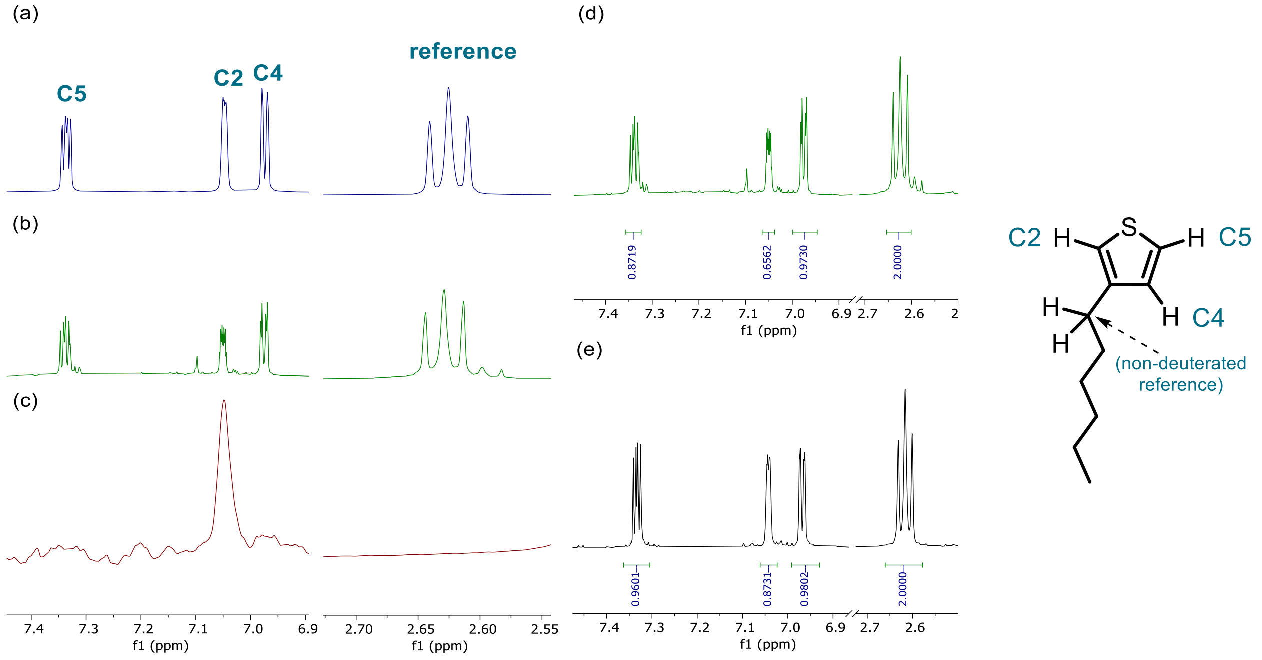

Figure S61: Comparison of ^{1}H-NMR spectra of d_0-**1**-hexyl (a) and deuterated d_x-**1**-hexyl with **L2** (b) and ^{2}H-NMR with **L2** (c) under standard reaction conditions without alkyne after 1h for deriving %D in d_6-acetone, 500 MHz, measured with zg10 for precise integration (d) with **L2** and (e) with **L16** after 4h.

^{1}H/^{2}H competition KIE

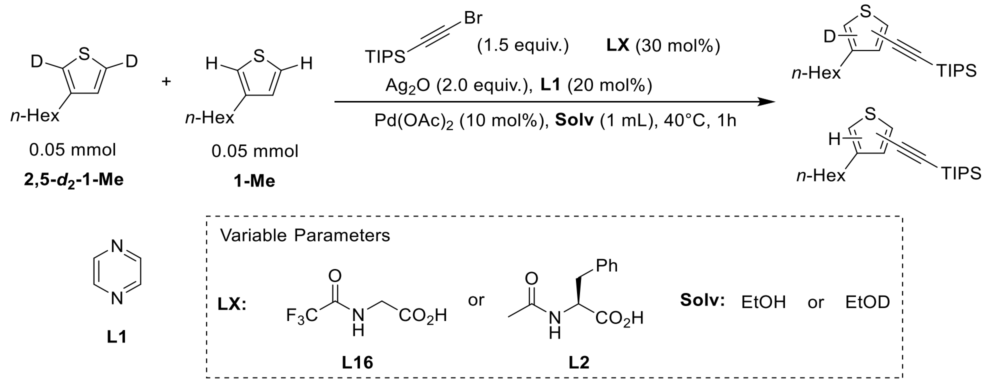

An oven-dried Schlenk tube (10 mL) was charged with a magnetic stirring-bar. Then $Pd(OAc)_2$ (2.3 mg, 0.010 mmol, 10 mol%), *N*-Acetyl-L-phenylalanine (6.2 mg, 0.030 mmol, 30 mol%) or 2-(2,2,2-trifluoroacetamido)acetic acid (5.1 mg, 0.030 mmol, 30 mol%), and Ag_2O (46.3 mg, 0.20 mmol, 2.0 equiv.) were added. Trimethyl 1,3,5-benzenetricarboxylate (~10 mg, 0.040 mmol, noted down per reaction) was added as internal standard. Pyrazine (1.6 mg, 0.020 mmol, 20 mol%) was added last due to volatility concerns. Half of the solvent (0.5 mL) was added. (Bromoethynyl)triisopropylsilane (39 mg, 1.5 equiv.) and 3-hexyl thiophene (8.4 mg, 0.050 mmol) and 2,5-d_2-3-hexyl thiophene (8.5 mg, 0.050 mmol) were added followed by the remaining EtOH or EtOD (0.5 mL). The tube was then tightly sealed with an inverted septum and parafilm and was stirred on a pre-heated metal block at 40°C for 18h. An aliquot (20 µL) was retrieved after 20min, 1 h and 18h. The aliquot was filtered through a plug of silica and eluted with EtOAc. The resulting solution was submitted to GC-FID and GC-MS analysis.

The reaction was carried out in duplicates in EtOH and EtOH for both ligands respectively and a sample was retrieved after 5 min, 60 min, and 18 h. The starting material was analyzed (see Table S20). Initially an almost equal distribution of 0-fold and 2-fold deuterated starting material is observed, as expected, even though in case of **L2** this balance is distorted slightly towards more 2-fold deuterated in case of EtOD and 0-fold deuterated material in case of EtOH. This effect is not pronounced for **L16**. This difference is even more pronounced after 60 min or 18 h. The appearance of a large amount of 1-fold deuterated starting material should be noted. This can only be explained by deuterium isotope exchange with the solvent. For **L16** the effect is small and larger overall degrees of deuteration (D_{Tot}) after 18h can be explained by the faster reactivity of the non-deuterated starting material since the increased D_{Tot} appears for EtOH (D_{Tot}=1.24) and EtOD (D_{Tot}=1.26). In case of **L2** a deuteration degree of D_{Tot} = 0.71 is seen in EtOH and D_{Tot} = 1.76 in EtOD. This is clearly caused by exchange with the reaction solvent. This observation provides additional qualitative support for a lower barrier of the C–H activation step and hence an easier possibility for isotope exchange with the excess isotope source from the solvent (higher $k_{ex,1}$ in Figure S58). A strong dependence on the D_{Tot} on the reaction media is seen in Table S21 and is either due to different amounts of deuterated starting material, caused by an isotope exchange of the starting material, or due to reversible C–H activation of the product and deuterium exchange with the solvent (higher $k_{ex,2}$ in Figure S58). This complicates a competition KIE determination for **L2**. Unlike in the parallel KIE determination, where product formation regardless of deuteration degree is determined, a major influence of deuterium exchange on the measured product ratios is observed here.

Table S20: Total deuterium (D_{Tot}) content of remaining d_0/d_2-thiophene mixture and distribution of non-, mono- and di-deuterated material as determined by GC-MS.

**3-Hexylthiophene**				
**5 min**	D_{Tot}	***0 fold***	***1 fold***	***2 fold***
**L2**, EtOH	0.92	50.0%	8.0%	41.9%
**L2**, EtOD	1.08	42.6%	7.6%	49.7%
**L16**, EtOH	1.04	47.1%	2.6%	50.3%
**L16**, EtOD	1.04	47.3%	2.5%	49.9%
**60 min**	D_{Tot}	***0 fold***	***1 fold***	***2 fold***
**L2**, EtOH	0.91	44.2%	20.7%	35.1%
**L2**, EtOD	1.28	26.8%	19.3%	53.6%
**L16**, EtOH	1.02	47.9%	3.1%	49.1%
**L16**, EtOD	1.06	45.2%	4.1%	50.4%
**18 h**	D_{Tot}	***0 fold***	***1 fold***	***2 fold***
**L2**, EtOH	0.71	37.3%	54.6%	8.0%
**L2**, EtOD	1.76	2.3%	20.2%	77.3%
**L16**, EtOH	1.24	35.1%	6.3%	58.5%
**L16**, EtOD	1.26	33.2%	8.0%	58.7%

Table S21: Total deuterium (D_{Tot}) content of the d_0/d_1-product mixture for C2 and C5 product respectively and distribution of non- and mono-deuterated material as determined by GC-MS.

	**C2**			**C5**		
**60 min**	D_{Tot}	***0 fold***	***1 fold***	D_{Tot}	***0 fold***	***1 fold***
**L2**, EtOH	0.42	60.7%	37.9%	0.20%	80.9%	18.8%
**L2**, EtOD	0.43	58.0%	41.6%	0.63%	38.4%	61.3%
**L16**, EtOH	0.26	75.6%	23.7%	0.23%	80.3%	19.0%
**L16**, EtOD	0.27	73.0%	27.0%	0.25%	76.6%	22.5%
**18 h**	D_{Tot}	***0 fold***	***1 fold***	D_{Tot}	***0 fold***	***1 fold***
**L2**, EtOH	0.58	43.1%	56.1%	0.14	86.3%	13.6%
**L2**, EtOD	0.65	36.3%	63.1%	0.86	15.9%	82.6%
**L16**, EtOH	0.30	70.3%	29.5%	0.28	75.1%	23.5%
**L16**, EtOD	0.32	69.1%	30.7%	0.32	68.3%	31.4%

A conservative error of σ_{fold} = ± 3% which corresponds to the maximal standard deviation between two averaged product analyses is assumed and the competition KIEs after 60min and 18h are indicated in Table S22 with their respective standard errors.

Table S22: Competition KIE for **L2** and **L16**.

	**C2**		**C5**	
**60 min**	KIE_{60min}	σ_{KIE}	KIE_{60min}	σ_{KIE}
**L2**, EtOH	1.60	0.15	4.31	0.71
**L2**, EtOD	1.39	0.12	0.63	0.06
**L16**, EtOH	3.19	0.42	4.23	0.69
**L16**, EtOD	2.71	0.32	3.41	0.47
**18 h**	KIE_{18h}	σ_{KIE}	KIE_{18h}	σ_{KIE}
**L2**, EtOH	0.77	0.07	6.36	1.42
**L2**, EtOD	0.58	0.05	0.19	0.04
**L16**, EtOH	2.39	0.26	3.19	0.43
**L16, EtOD**	**2.25**	**0.24**	**2.18**	**0.23**

The **L16** in EtOD experiment after 18h can be considered the only reliable competition KIE experiment since here a) the reaction is completed, b) no major isotope exchange was detected, c) a reduced influence due to solvent isotope effect and hence a better comparability with the parallel KIE experiments is ensured. The values are remarkably close to the parallel KIE values thus strengthening the conclusions from parallel KIE experiments.

4.3 Variable Time Normalization Analysis

An oven-dried Schlenk tube (10 mL) was charged with a magnetic stirring-bar. Then $Pd(OAc)_2$ (4.5 mg, 0.020 mmol, 10 mol%), *N*-Acetyl-L-phenylalanine (12.3 mg, 0.030 mmol, 30 mol%) or 2-(2,2,2-trifluoroacetamido)acetic acid (10.3 mg, 0.030 mmol, 30 mol%), and Ag_2O (92.6 mg, 0.20 mmol, 2.0 equiv.) were added. Trimethyl 1,3,5-benzenetricarboxylate (~10 mg, 0.040 mmol, noted down per reaction) was added as internal standard. Pyrazine (3.2 mg, 0.020 mmol, 20 mol%) was added last due to volatility concerns. Half of the solvent (1.0 mL) was added. (Bromoethynyl)triisopropylsilane (78 mg, 1.5 equiv.) and 3-hexyl thiophene (34 mg, 0.20 mmol) or 2,5-d_2-3-hexyl thiophene (34 mg, 0.20 mmol) were added followed by the remaining EtOH (1.0 mL). The tube was then tightly sealed with an inverted septum and parafilm and was stirred on a pre-heated metal block at 40°C for 6h. An aliquot (20 µL) was retrieved every 10 min for 2 h, every 30 min for 2-6h. The aliquot was filtered through a plug of silica and eluted with EtOAc. The resulting solution was submitted to GC-FID analysis.

The amounts of catalyst ($Pd(OAc)_2$, **L1**, and N-acyl amino acid), alkyne source and thiophene were varied from the above mentioned standard condition as specified in Table S23. A variable time normalization analysis (VTNA) was performed, as described in the literature.[117,118] For a less biased determination of reaction orders, we employed the newly developed Auto-VTNA tool.[119] The curve with the standard concentration was used as a reference and therefore averaged over two runs to ensure comparability.

Order in thiophene

The order in thiophene was determined at 0.05 M and compared with the standard concentration at 0.10 M (Figure S62, Figure S62). For L2 the experiment at 0.05 M was repeated to investigate experimental fluctuations. For L16 an additional curve was generated for 0.15 M to confirm the order and investigate potential concentration dependencies. For **L16** orders around 1 were detected (see Figure S64q-t). For **L2** Auto-VTNA initially determined orders around 1.6 and 1.3 for C2 and C5 respectively (Figure S64). Since this appeared to be rather large, we duplicated the results leading to qualitatively similar results (1.7 and 1.3). Upon closer inspection of the VTNA plot, we see an imperfect alignment of both curves. Using values of 1 for the orders, the curves close to the initial concentrations align significantly better (see Figure S64c,d,g,h) at a cost of a larger deviation in the latter part of the curve. To ensure no detrimental effects are caused, by estimating the concentration of remaining thiophene via the formed product,[7] we determined the concentration of remaining thiophene directly via GC-FID and repeated the VTNA procedure. Now, values closer to unity are observed (C2 1.2 and C5 1.0, Figure S64i,l and j,m) and the VTNA plot shows overall better alignment with the curve obtained under standard reaction conditions. We therefore deem these values to be

[7] We originally expected that this method is more appropriate in our case since the internal standard is closer in retention time to the product and a nice linear build-up is observed for the formed product, while larger fluctuations are observed in some runs for starting material determination.

trustworthy. Looking at Figure S62 it is apparent when comparing the calculated and measured *c* vs *t* profiles of **1** that (especially for **L2**) [T] decreases faster than expected from product formation. Using the actual concentration in thiophene led to trustworthy results in this case, since they take into account reagent substrate decomposition at higher conversions. In case of **L16** virtually identical values are obtained for both methods of concentration determination (0.9, 1.0 vs 0.9, 1.0 Figure S64o,p vs q,r).

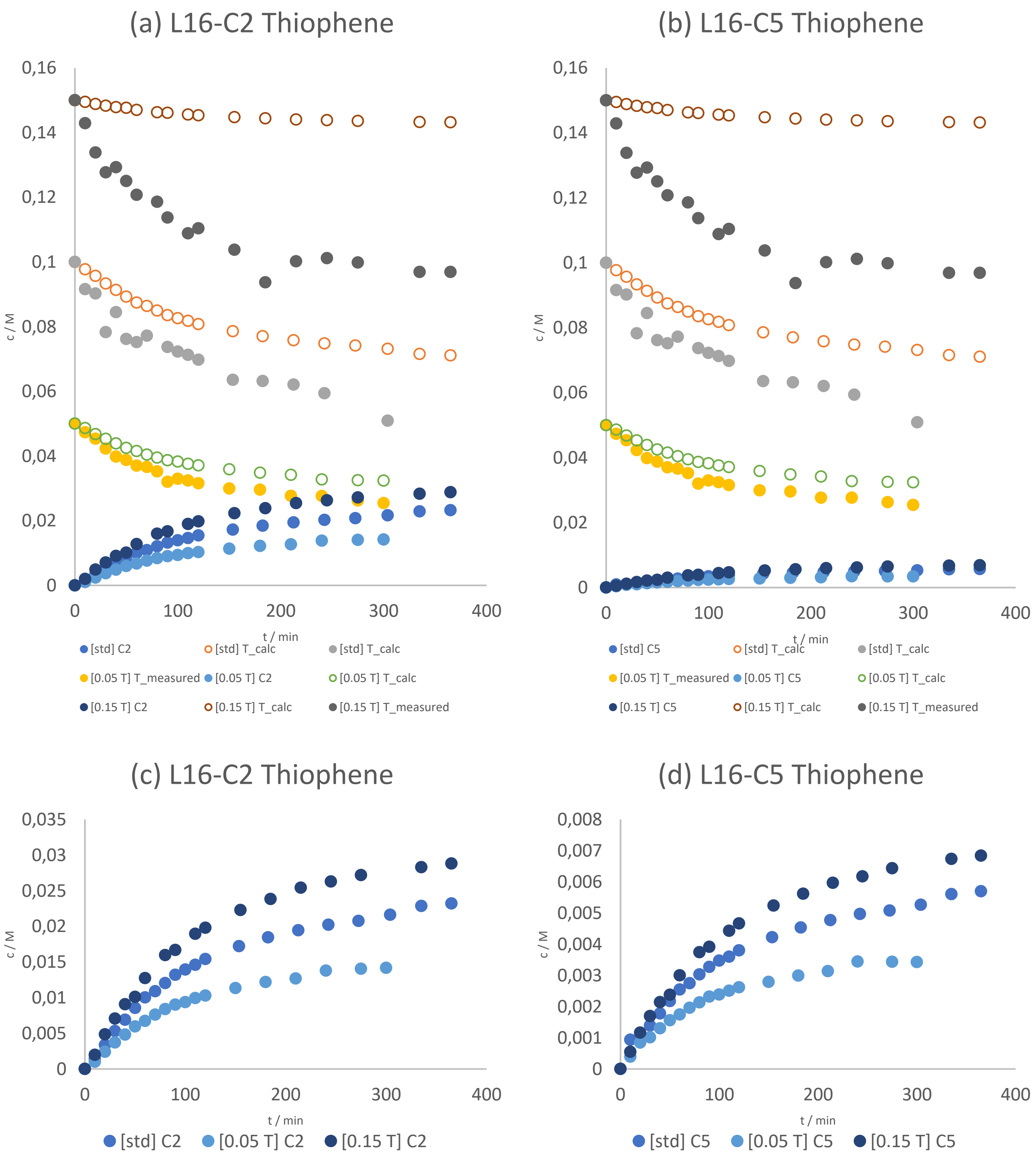

Figure S62: Concentration (M) vs time profiles (min) for the thiophene order determination of (a) L16-C2, (b) L16-C5 with zoom on only product formation for (c) **L16**-C2 and (d) **L16**-C5 with concentrations of product **3** under standard and varied thiophene concentrations [T]. Concentration of thiophene calculated based on product formation or values derived directly from GC-FID.

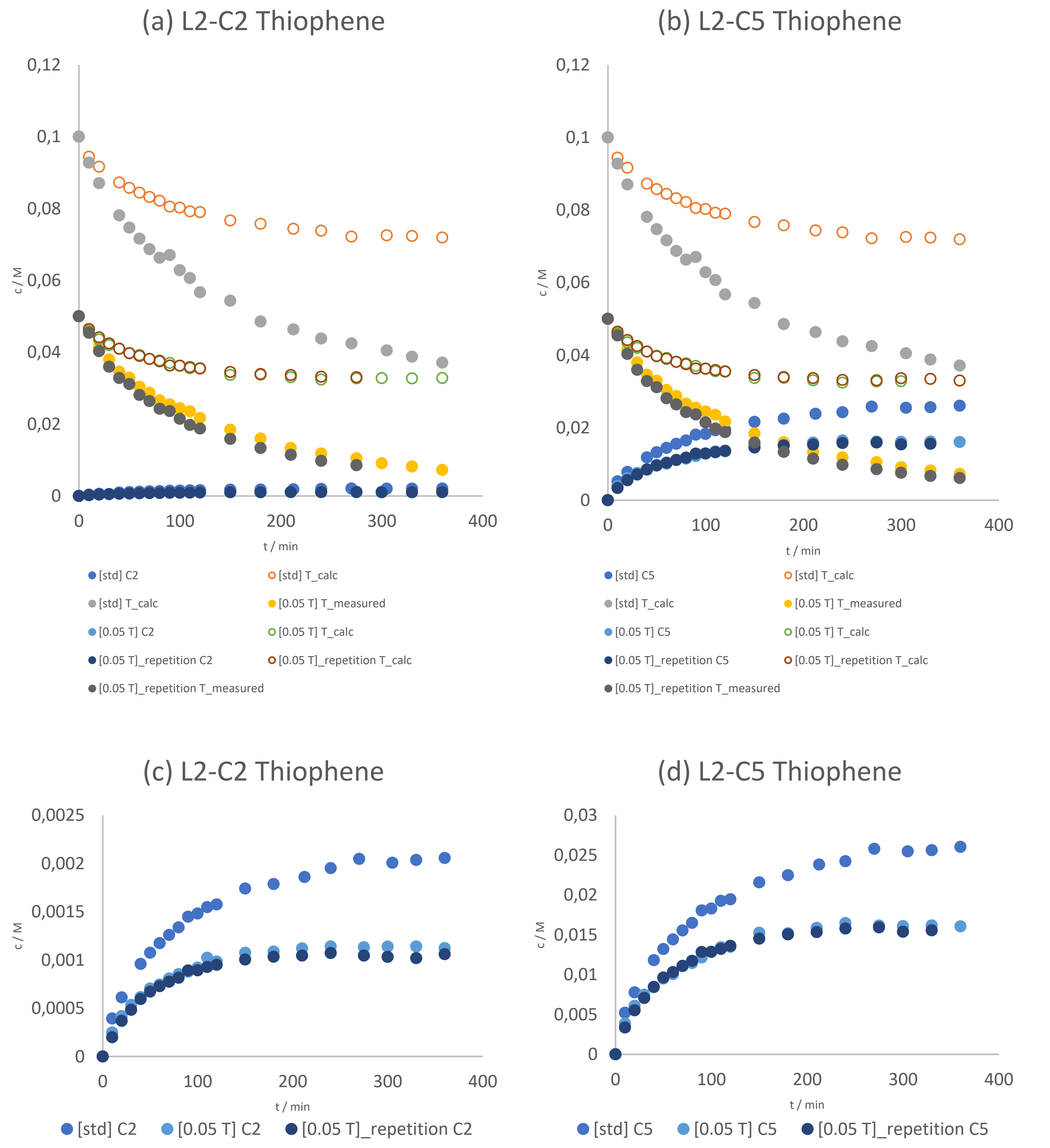

Figure S63: Concentration (M) vs time profiles (min) for the thiophene order determination of (a) **L2**-C2, (b) **L2**-C5 with zoom on only product formation for (c) **L2**-C2 and (d) **L2**-C5 with concentrations of product **3** under standard and varied thiophene concentrations [T]. Concentration of thiophene calculated based on product formation or values derived directly from GC-FID.

(a)

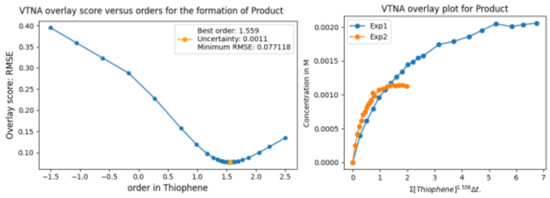

(b)

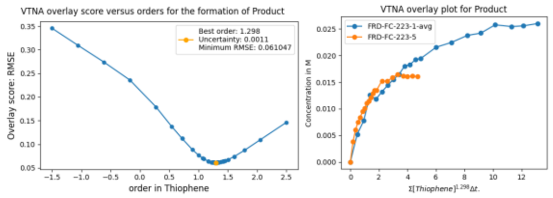

(c)

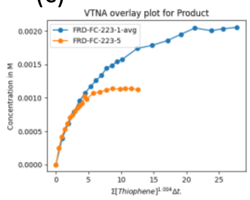

(d)

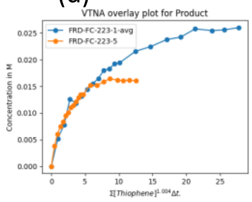

(e)

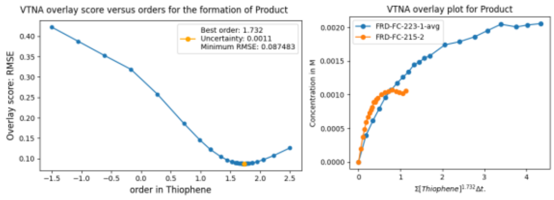

(f)

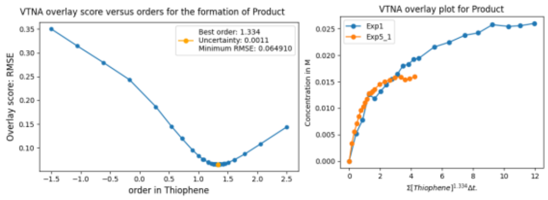

(g)

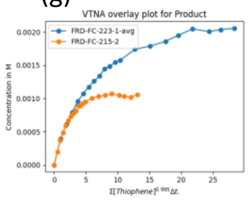

(h)

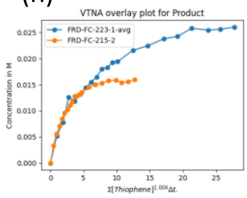

(i)

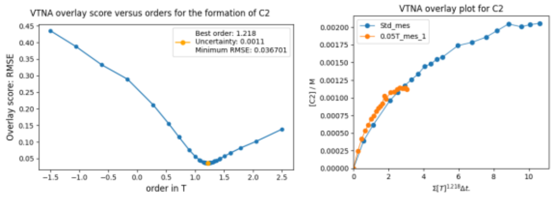

(j)

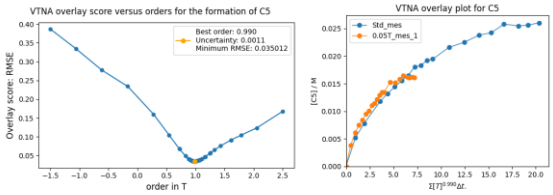

(k)

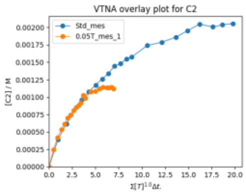

(l)

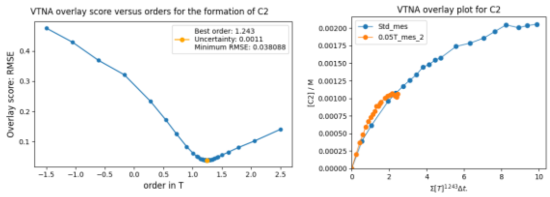

(m)

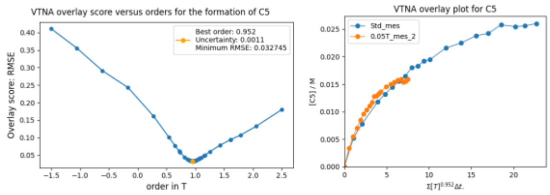

(n)

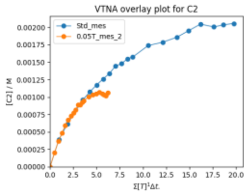

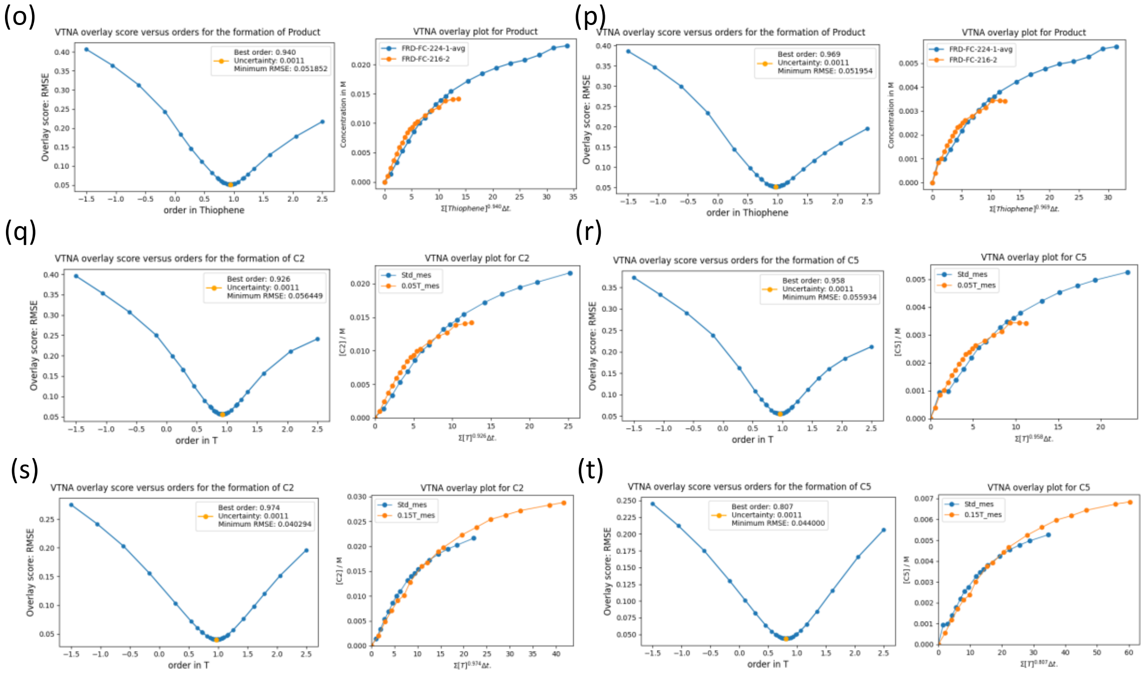

Figure S64: Determination of thiophene order (0.10 M vs X M) with Auto-VTNA showing the RMSE score and the actual VTNA plot at the best order for **L2** with a repetition experiment (a, e) C2 and (b, f) C5 with 0.05 M thiophene with the concentration in remaining thiophene derived from product formation. VTNA plot for (c, g) C2 and (d, h) C5 with an order of 1 for comparison. RMSE score and VTNA plots for (i, l) C2 and (j, m) C5 with 0.05 M thiophene with the concentration in thiophene determined by GC-FID and VTNA plot for (k, n) C2 with the order of 1 for comparison. Identical plots for **L16** are shown for the order in thiophene (0.1 M vs X m) (o) C2 and (p) C5 with 0.05 M thiophene with the concentration in remaining thiophene derived from product formation. RMSE score and VTNA plots for (q) C2, (r) C5 with 0.05 M and (s) C2, (t) C5 0.15 M thiophene with the concentration in thiophene determined by GC-FID.

Order in alkyne

The order in alkyne was determined at 0.0375 M compared to the original concentration of 0.15 M (Figure S65, Figure S66) for both ligands. We used the measured concentration in alkyne for the VTNA analysis (see Figure S65 for the reasons outlined for thiophene and found higher orders in alkyne when using **L2** compared to using **L16**. To ensure the results are reliable, we repeated the experiment at concentrations close to 0.0375 M for **L2**, namely 0.025 M and 0.05 M and similar values were obtained (within the reasonable error margin). This led us to conclude that the results are in principle trustworthy. Since the order in alkyne for **L16** was rather high, we wanted to investigate the concentration-dependency and also determined the order at a higher alkyne concentration 0.1 M for **L2**. Here effectively lower orders were observed. One can rationalize this dependency and the fact that non-zero orders were obtained for **L16** and non-unit orders for **L2** using the elasticity coefficient (*vide infra*).

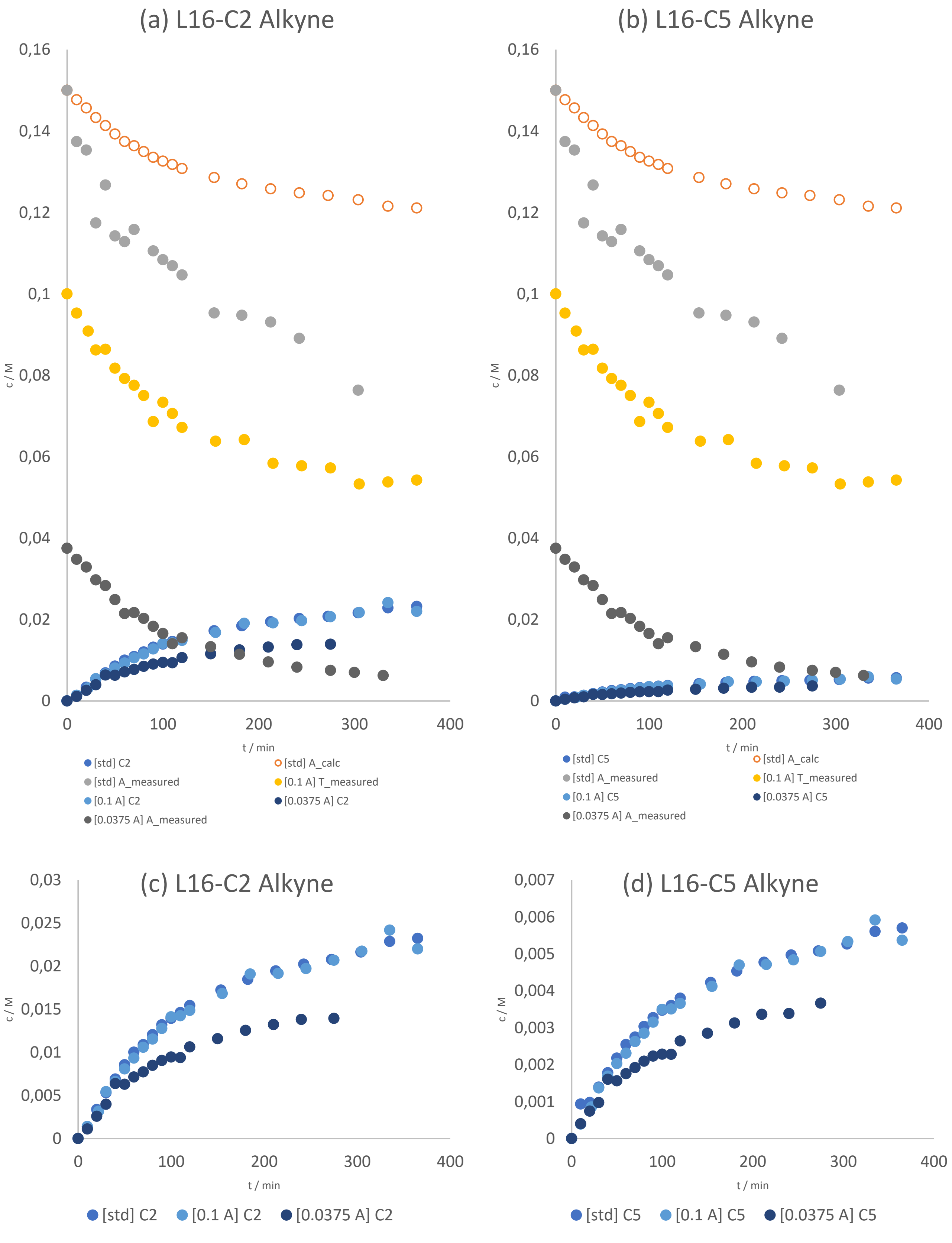

Figure S65: Concentration vs time profiles for the alkyne order determination of (a) **L16**-C2, (b) **L16**-C5, zoom on only product formation for (c) **L16**-C2 and (d) **L16**-C5 with concentrations of product **3** under standard and varied alkyne concentrations [A].

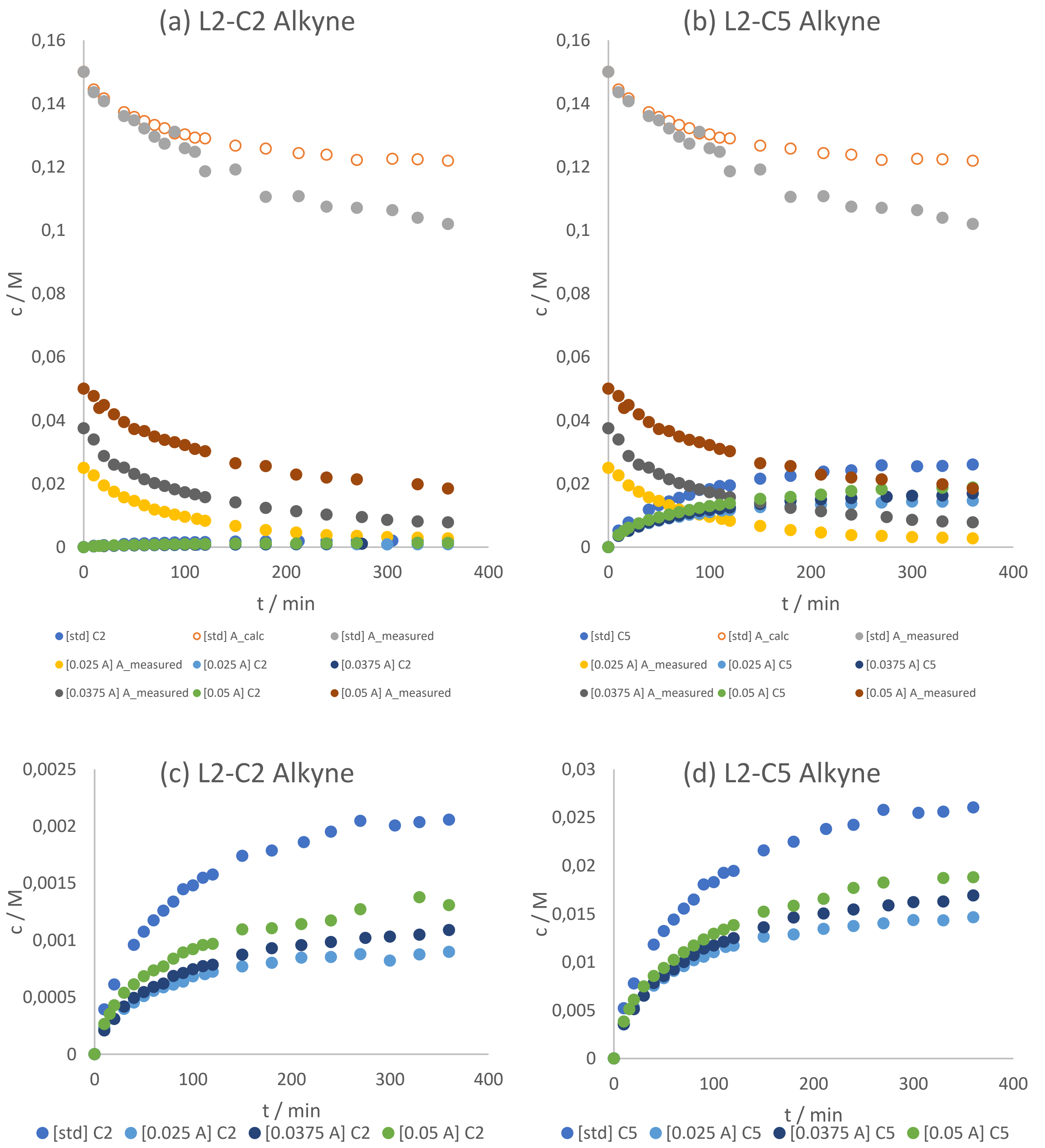

Figure S66: Concentration vs time profiles for the alkyne order determination of (a) **L2**-C2, (b) **L2**-C5, zoom on only product formation for (c) **L2**-C2 and (d) **L2**-C5 with concentrations of product **3** under standard and varied alkyne concentrations [A].

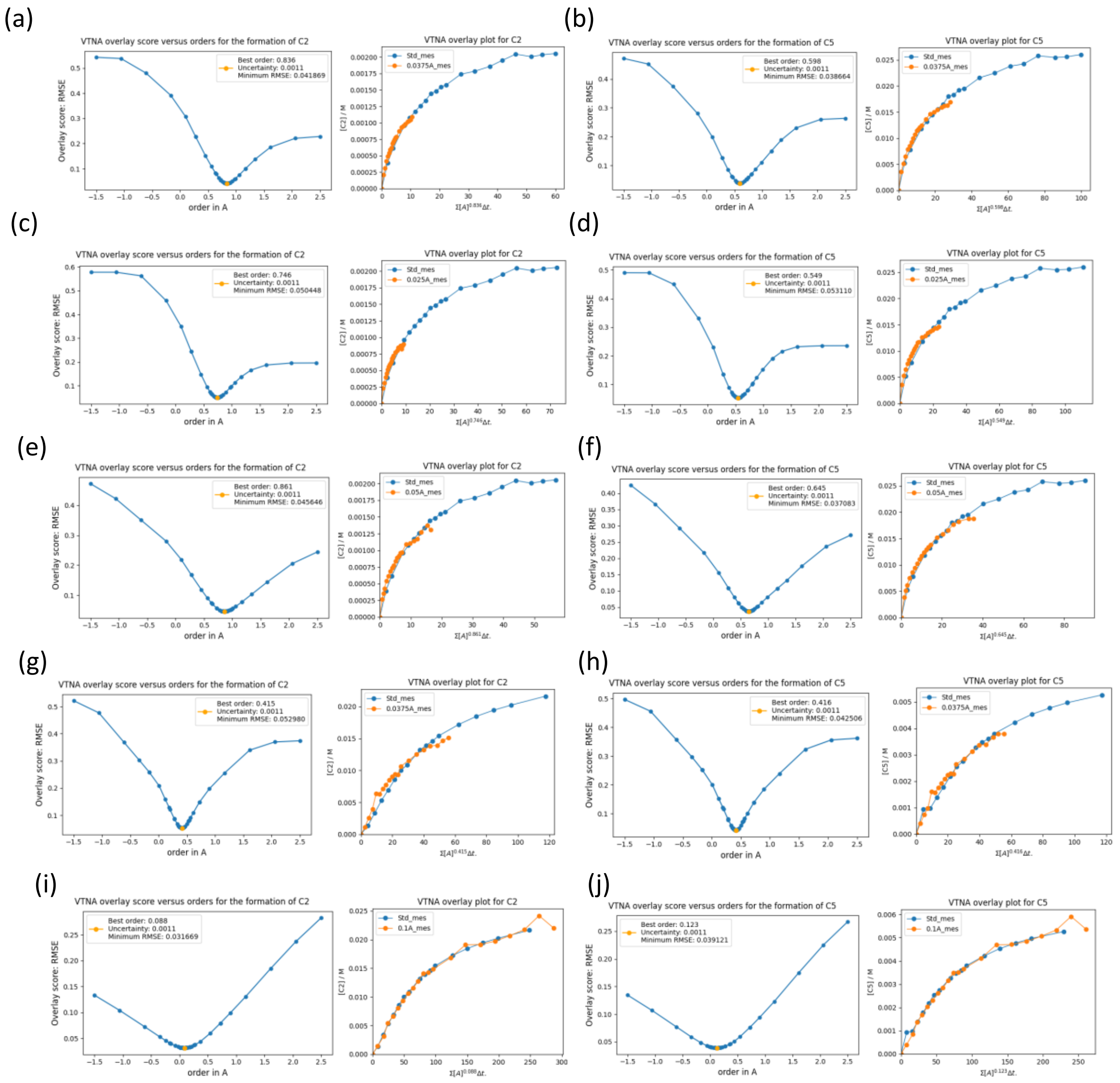

Figure S67: Determination of alkyne order (0.15 M vs X M) with Auto-VTNA showing the RMSE score and the actual VTNA plot at the best order for **L2** (a) C2, (b) C5 with 0.0375 M alkyne (c) C2, (d) C5 with 0.025 M alkyne and (e) C2, (f) C5 with 0.05 M alkyne. Identical plots for **L16** are shown for the order in alkyne (g) C2, (h) C5 with 0.0375 M alkyne and (i) C2, (j) C5 with 0.1 M alkyne.

Order in catalyst

We initially attempted to determine the order in catalyst over the entire course of the reaction but this led to physically unsound results since catalyst decomposition in this system cannot be monitored via GC-FID (Figure S68) and a certain amount of catalyst decomposition is expected at later time points.

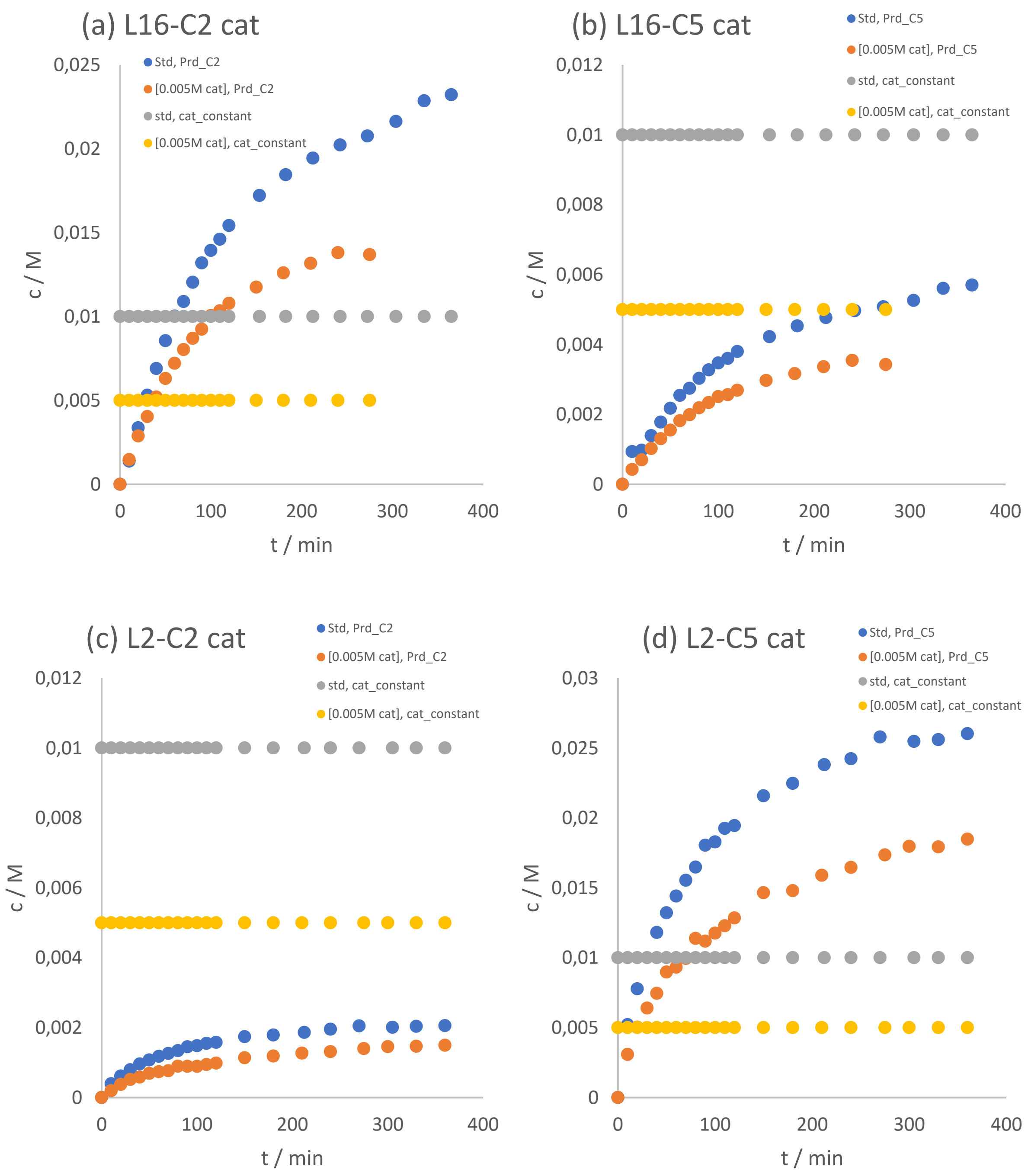

Figure S68: Concentration vs time profiles for the catalyst order determination of (a) L16-C2, (b) L16-C5, (c) L2-C2 and (d) L2-C5 with concentrations of product **3** under standard and lowered catalyst concentrations [cat] and concentration of catalyst assumed to be constant and with exponential decomposition.

We therefore opted to only consider time points from 0 – 60 min since in this regime catalyst decomposition should still be negligeable.

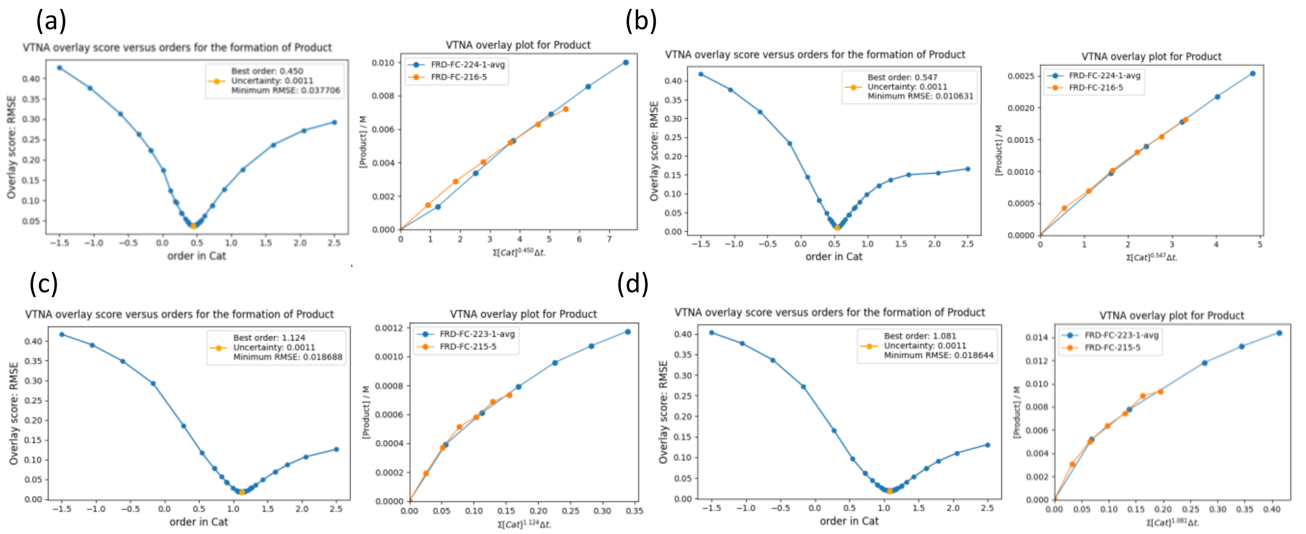

Figure S69: Determination of catalyst order (0.01 M vs 0.005 M) with Auto-VTNA showing the RMSE score and the actual VTNA plot at the best order for **L16** (a) C2 (b) and **L2** (c) C2 and (d) C5 for t = 0 – 60 min.

Summarized orders and elasticity coefficient

The determined orders and the deviation from the standard conditions are summarized in Table S23.

Table S23: Reaction order derived from deviation from standard conditions: $Pd(OAc)_2$ 0.01 M, **L1** (0.02 M), L1 (0.03 M), Ag_2O (0.2 M), Alkyne (0.15 M), 3-hexylthiophene (0.2 M), EtOH (1 mL), 40°C.

L2, Deviation from standard conditions	Order C2 ±0.2	Order C5 ±0.2	L16, Deviation from standard conditions	Order C2 ±0.2	Order C5 ±0.2
**Thiophene**			**Thiophene**		
0.05 mmol	1.56[a]	1.30[a]	0.05 mmol	0.93[b]	0.96[b]
0.05 mmol	1.22[b]	0.99[b]	0.15 mmol	0.97[b]	0.81[b]
0.05 mmol (repetition)	1.72[a]	1.33[a]			
0.05 mmol (repetition)	1.24[b]	0.95[b]			
**Alkyne**			**Alkyne**		
0.0375 mmol	0.83[b]	0.62[b]	0.0375 mmol	0.42[b]	0.42[b]
0.025 mmol	0.74[b]	0.57[b]	0.10 mmol	0.06[b]	0.11[b]
0.050 mmol	0.89[b]	0.67[b]			
**Catalyst**			**Catalyst**		
0.005 mmol	1.12[d]	1.08[d]	0.005 mmol	0.45[d]	0.55[d]

[a] concentration of species of interest derived indirectly via product formation, [b] concentration of species of interest estimated by GC-FID, [c] estimated assuming no catalyst decomposition, [d] using only time points until t = 60 min and constant concentration in catalyst.

We carried out order determinations for altered concentrations in at least two permutations (either repetition or slightly altered concentrations) and compared the results to the standard reference conditions in the VTNA analysis that were averaged over two runs. We estimate an error of ±0.2 on all orders based on the repetition experiments and the fluctuations from the alkyne order determination in a narrow concentration range. Please also be referred to the individual RMSE plots in Figure S64, Figure S67, and Figure S69 for more details on the goodness-of fit: a steep curve indicates an easy and precise determination of the order whereas a flat

curve possibly offers a broader range of orders within an acceptable RMSE range. The absolute RMSE value also gives information on the fit and or data quality.

Conclusions order thiophene:

Near unity orders are obtained for thiophene in all cases. One thiophene molecule needs to be present in the turnover limiting transition state. This is the case for both the C–H activation and the MI step.

Conclusions order alkyne:

To explain the non-zero order in alkyne for **L16** and the non-unity order for **L2** we set out to study the elasticity coefficient (eq. 6)[120] as a function of the concentration in alkyne[A] and the difference in MI vs C–H activation barrier.[8]

$$\varepsilon_{[A]}^{r} = \frac{dr}{d[A]} \cdot \frac{[A]}{r} \text{ elasticity coefficient for changes in [A]} \tag{6}$$

Herein, the elasticity coefficient or normalized sensitivity is the order of the catalytic reaction over different concentrations in [A]. In order to apply the normalized sensitivity to our reaction we derived it for the simplified catalytic cycle in Figure S70.

Simplified Catalytic Cycle

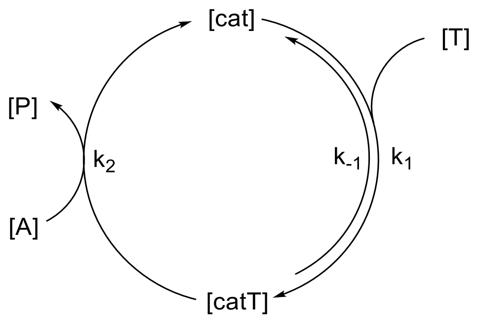

k_1 = rate constant of the C–H activation

k_{-1} = rate constant of the reverse C–H activation

k_2 = rate constant of the MI

$[cat]_{Tot}$ = [cat] + [catT] (mass balance of catalytic species)

[T] = rate constant of the concentration in thiophene

[A] = rate constant of the concentration in alkyne

Figure S70: Simplified catalytic cycle to probe the concentration dependency of the catalyst order.

The high energy steps, i.e. the C–H activation and MI are included since both can in principle be turnover limiting. The steps after the MI are overall very exergonic and are therefore not featured in the cycle.

To derive the elasticity coefficient the steady-state approximation is applied on the change in catalyst concentration.

$$\frac{d[cat]}{dt} = [catT](k_{-1} + k_2[A]) - k_1[T][cat] = 0 \text{ steady state approximation} \tag{7}$$

The overall concentration in catalyst is unchanged and divided between [cat] and [catT]

---

[8] Please note that this is a mere qualitative analysis. Calculated values are used as an input to show that different order dependencies can in principle be rationalized. Importantly, only computationally derived values can be used for such a simulation, since we do not have a means to experimentally determine some of the values required, e.g. the $\Delta\Delta G^{\ddagger}_{\text{C2 vs C5}}$ values for the non-selectivity determining step. Also, the $\Delta\Delta G^{\ddagger}_{\text{C–H activation vs MI}}$ values cannot be determined experimentally since again one non-rate determining step is under study. Furthermore, during the experimental order determination, the concentration change in the VTNA analysis whereas we regard orders at a fixed concentration here. We furthermore use a simplified yet representative catalytic cycle and use the Eyring equation which are again simplifications. Nevertheless, this qualitative analysis allows us to rationalize important experimental observables.

$$[cat]_{\mathrm{Tot}} = [cat] + [catT] \text{ mass balance of catalytic species} \quad (8)$$

The rate law for product formation can be expressed accordingly.

$$\text{Rate law: } r = \frac{d[P]}{dt} = k_2 \cdot [A] \cdot [catT] \quad (9)$$

One can then rearrange mass balance:

$$[cat] = [cat]_{\mathrm{Tot}} - [catT] \quad (10)$$

And insert into steady-state approximation and solve for [catT]:

$$[catT] = \frac{k_1[T][cat]_{\mathrm{Tot}}}{k_{-1} + k_2[A] + k_1[T]} \quad (11)$$

Replace [catT] can then be inserted in rate law to derive the following expression

$$r = \frac{k_1 k_2 \, [A][T][cat]_{\mathrm{Tot}}}{k_{-1} + k_2[A] + k_1[T]} \quad (12)$$

Which can in turn be used to derive the elasticity coefficient as a function of [T]

$$\varepsilon^{r}_{[A]} = \frac{dr}{d[A]} \cdot \frac{[A]}{r} = \frac{d\left(\frac{k_1 k_2 \, [A][T][cat]_{\mathrm{Tot}}}{k_{-1} + k_2[A] + k_1[T]}\right)}{d[A]} \cdot [A] \frac{k_{-1} + k_2[A] + k_1[T]}{k_1 k_2 \, [A][T][cat]_{\mathrm{Tot}}} \quad (13)$$

The elasticity coefficient in its final form depends on the individual rates, the concentration in [A] and [T]

$$\boldsymbol{\varepsilon^{r}_{[A]} = \frac{k_{-1} + k_1[T]}{k_1[T] + k_{-1} + k_2[A]}} \quad (14)$$

Using the Eyring equation one can derive the reaction rate constants k_1, k_{-1} and k_2. By fixing the MI barrier and allowing the C–H activation barrier to change one can derive the order dependency on the relative height of the MI and C–H activation barrier, the difference $\Delta\Delta G^{\ddagger}_{\text{C–H activation vs MI}}$ at a fixed concentration of [A].[9] For this, equation 14 can be used to calculate $\varepsilon_{[A]}$ at different concentrations. We used [A] = 0.15 M for the high concentration and [A] = 0.0375 M for the low concentration run with [T] = 0.10 M in both cases, similar to the VTNA experiments and kept the concentrations constant. As shown in Figure S71a, only the C–H activation barrier is varied for this simulation, the MI barrier and Int[10] are fixed. This results in changed values for k_1 and k_{-1} upon changing the C–H activation barrier and a constant k_2.[11] Figure S71b shows that for low concentration in alkyne potentially higher order values are obtained than for high alkyne concentration. More importantly, it also shows that a non-zero order in alkyne is to be expected even when the C–H activation barrier is higher in energy (right-hand side of Figure S71b). It furthermore shows that values smaller than 1 are obtained even when the MI step has the highest energy barrier (left-hand side of Figure S71b). It also shows that large differences in energy between C–H activation and MI are needed for the alkyne to approach an order of 0 or 1.

---

[9] Note that this is not the same as the experimental selectivity since this is defined as the relative selectivity of C2 vs C5 for e.g. the C–H activation ($\Delta\Delta G^{\ddagger}_{\text{C2 vs C5}}$(C–H activation)) or the MI ($\Delta\Delta G^{\ddagger}_{\text{C2 vs C5}}$(MI)). $\Delta\Delta G^{\ddagger}_{\text{MI vs C–H activation}}$ could be derived for C2 and C5 individually.

[10] We tried also varying the positioning of Int between 0 – 18 kcal/mol and it had virtually no influence on the final graph and therefore set it to 3 kcal/mol as this corresponds approximately to the calculated value for Int-4 of e.g. **L2** (*vide supra*).

[11] We set the MI TS to a representative value of 23 kcal/mol, choosing a different value here would not change the final graph since we only look at relative differences between C–H activation and MI activation barriers.

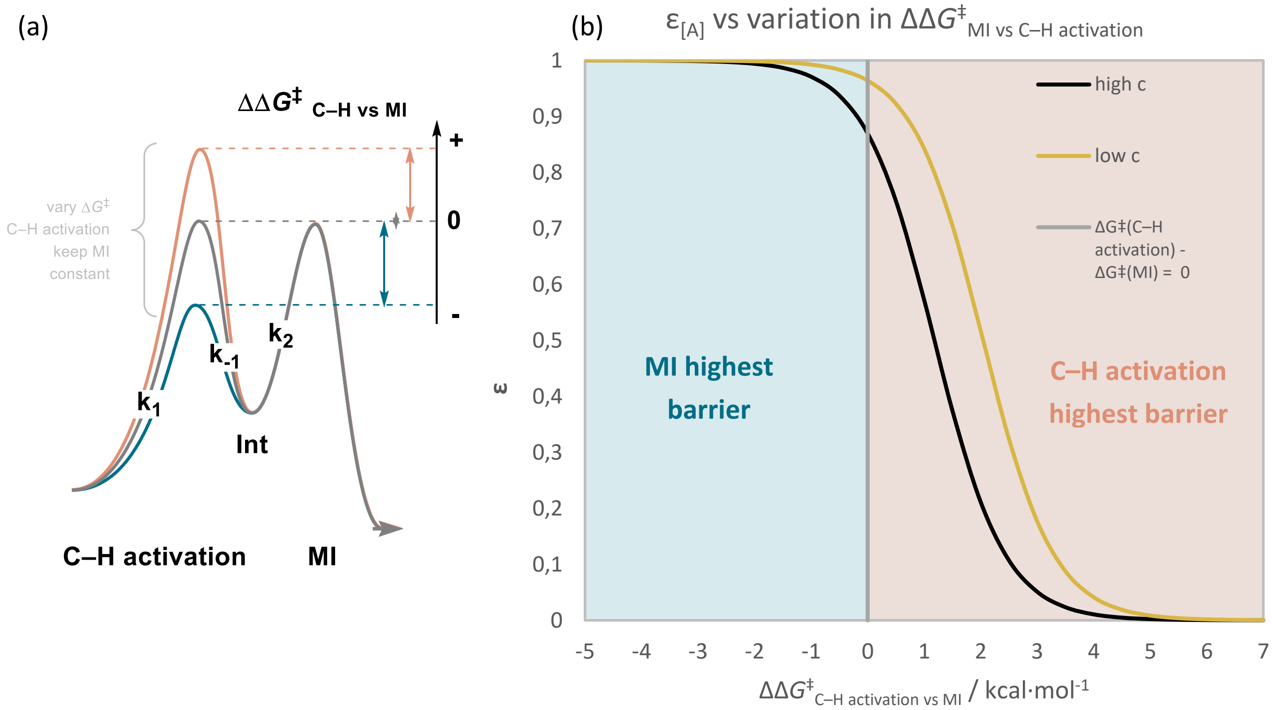

Figure S71: (a) Variation of the C–H activation barrier at a constant Int and MI to derive changed k_1 and k_{-1} as a function of $\Delta\Delta G^{\ddagger}_{\text{C–H activation vs MI}}$. (b) Qualitative change in elasticity coefficient at high [A] = 0.15 M and low [A] = 0.0375 M as a function of $\Delta\Delta G^{\ddagger}_{\text{C–H activation vs MI}}$ / kcal·mol^{-1}.

Different orders can hence be obtained for the different isomers C2 vs C5 using one ligand. For **L2**-C2 a higher MI-barrier is expected, since this product is experimentally disfavored and the C–H activation has been shown to be C2 selective. A larger difference between MI and C–H activation can therefore be assumed in case of L2-C2 vs **L2**-C5 and hence higher orders for **L2**-C2 vs **L2**-C5 which is also experimentally observed. The overall higher orders in alkyne using **L2** indicate that MI barrier is larger compared to **L16**.

The identical procedure was repeated for [T] using equation 15. A constant order of ~1 is visible regardless of low or high concentration as expected, due to the involvement of thiophene in both steps of the pathway. At very high values for [T] a saturation behavior might be possible, which is not captured in the model and not observed according to Table S23.

$$\varepsilon^{r}_{[T]} = \frac{\boldsymbol{k_{-1} + k_2[A]}}{\boldsymbol{k_1[T] + k_{-1} + k_2[A]}} \qquad (15)$$

Figure S72 shows the order dependency of thiophene with [A] = 0.15 M and [T] = 0.1 M (standard reaction conditions).

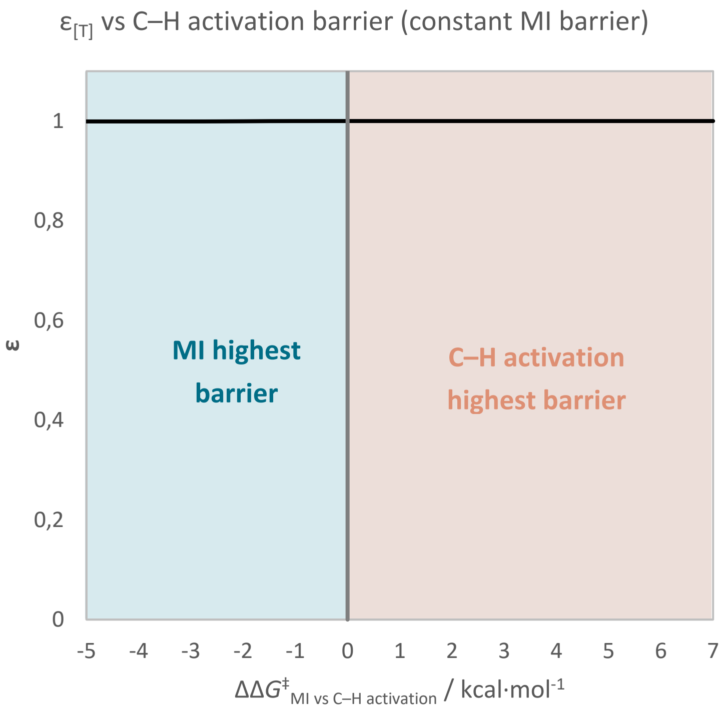

Figure S72: Change in elasticity coefficient at a constant [T] as a function of $\Delta\Delta G^{\ddagger}_{\text{C–H activation vs MI}}$ / kcal·mol^{-1}.

One can then derive how the order could theoretically change as a function of the concentration in [A] in Figure S73. We set $[A]_0 = [T]_0$ with quantitative product formation $[A] = [A]_0 – [P]$ and $[T] = [T]_0 – [P]$. Note that [T] has very little influence within the concentration ranges considered.[12]

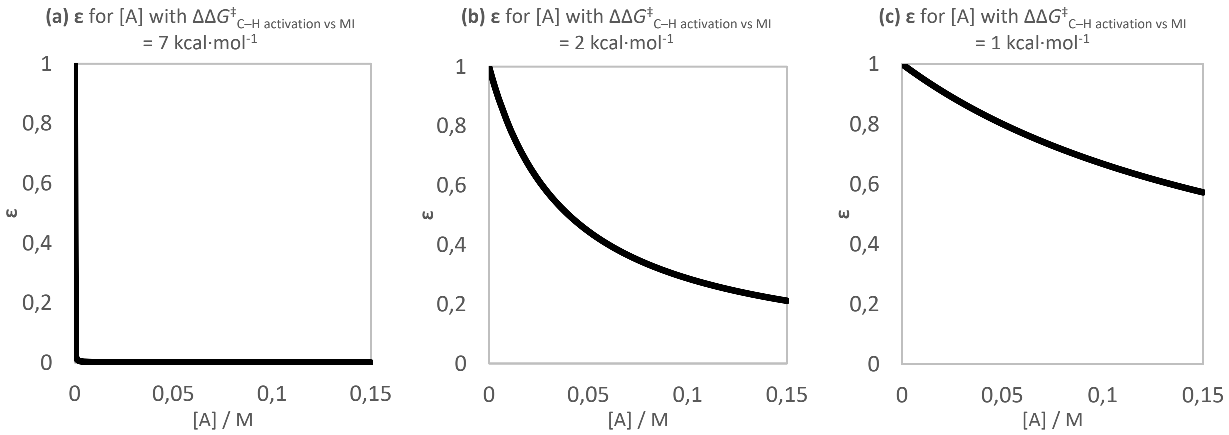

[12] Figure S73b, $\Delta\Delta G^{\ddagger}_{\text{C–H activation vs MI}}$ = 2 kcal·mol^{-1}: High alkyne: **[A] = 0.15 M, [T] = 0.15 M, $\varepsilon_{[A]}$ = 0.211**; [A] = 0.15 M, [T] = 0 M, $\varepsilon_{[A]}$ = 0.211; [A] = 0.15 M, [T] = 0.015 M, $\varepsilon_{[A]}$ =0.211; [A] = 0.15 M, [T] = 1.5 M, $\varepsilon_{[A]}$ = 0.213. Low alkyne: [A] = 0.015 M, [T] = 0.15 M, $\varepsilon_{[A]}$ = 0.728; [A] = 0.015 M, [T] = 0 M, $\varepsilon_{[A]}$ = 0.728; **[A] = 0.015 M, [T] = 0.015 M, $\varepsilon_{[A]}$ =0.728**; [A] = 0.015 M, [T] = 1.5 M, $\varepsilon_{[A]}$ = 0.730.
Figure S73e, $\Delta\Delta G^{\ddagger}_{\text{C–H activation vs MI}}$ = -1 kcal·mol^{-1}: High alkyne: **[A] = 0.15 M, [T] = 0.15 M, $\varepsilon_{[A]}$ = 0.971**; [A] = 0.15 M, [T] = 0 M, $\varepsilon_{[A]}$ = 0.971; [A] = 0.15 M, [T] = 0.015 M, $\varepsilon_{[A]}$ = 0.971; [A] = 0.15 M, [T] = 1.5 M, $\varepsilon_{[A]}$ = 0.971. Low alkyne: [A] = 0.015 M, [T] = 0.15 M, $\varepsilon_{[A]}$ = 0.997; [A] = 0.015 M, [T] = 0 M, $\varepsilon_{[A]}$ = 0.997; **[A] = 0.015 M, [T] = 0.015 M, $\varepsilon_{[A]}$ = 0.997**; [A] = 0.015 M, [T] = 1.5 M, $\varepsilon_{[A]}$ = 0.997.

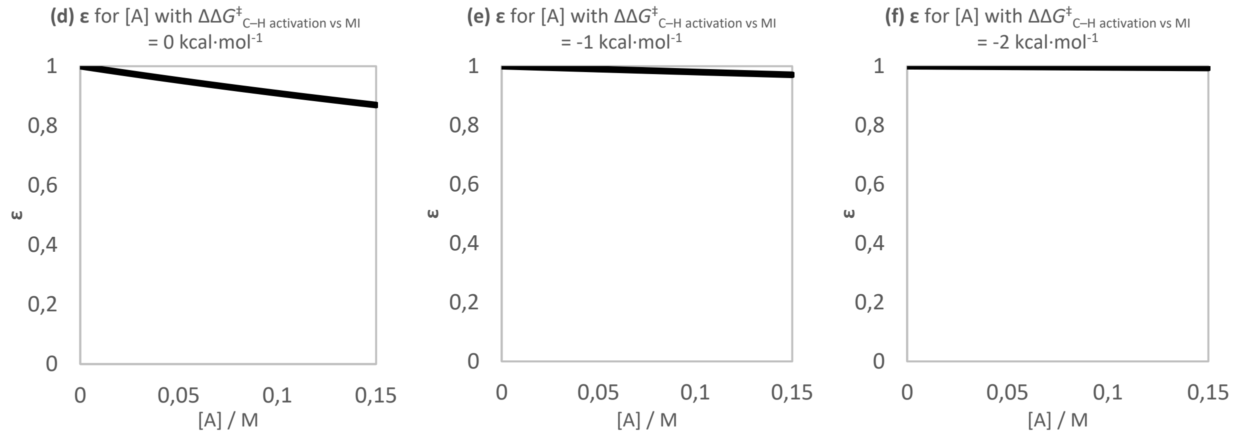

Figure S73: Change in elasticity coefficient as a function of [A] for $\Delta\Delta G^{\ddagger}_{\text{C–H activation vs MI}}$ / kcal·mol^{-1} with (a) 7 kcal·mol^{-1}, (b) 2 kcal·mol^{-1}, (c) 1 kcal·mol^{-1}, (d) 0 kcal·mol^{-1}, (e) -1 kcal·mol^{-1}, (f) -2 kcal·mol^{-1}.

At very high barrier differences, i.e. very high C–H activation TS, this dependency becomes very steep and essentially a 0 order would be expected at most studied concentrations (see Figure S73a).

From the plot it becomes apparent that in the approximate concentration range under study there is a strong dependency of $\varepsilon_{[A]}$ on the concentration [A], when the C–H activation barrier is higher (~1-2 kcal/mol) than the MI barrier (see Figure S73b,c). This is a range to be expected for **L16** and is in line with the observed order dependency in Table S23.

We have experimentally investigated the concentration dependency on purpose at lower alkyne concentrations and not higher alkyne concentrations compared to the standard conditions since at higher concentrations saturation behavior and apparent lower orders might be observed as proposed by e.g. Carrow due to a favorable coordination of substrate to the catalyst.[49,50] This could in principle cause lower orders, but only at very high alkyne concentrations which are not considered here. We have computationally probed resting states involving alkyne (and thiophene) as π-donating ligands or a combination of both, but none were found to be competitive with the proposed resting states (see Figure S74). At higher alkyne concentrations equilibria might still be slightly pushed slightly towards catalyst species bearing coordinated alkyne(s) (instead of two L1) making quantitative predictions of the order difficult. However, at concentrations in alkyne relevant to this study, this effect should be negligible.

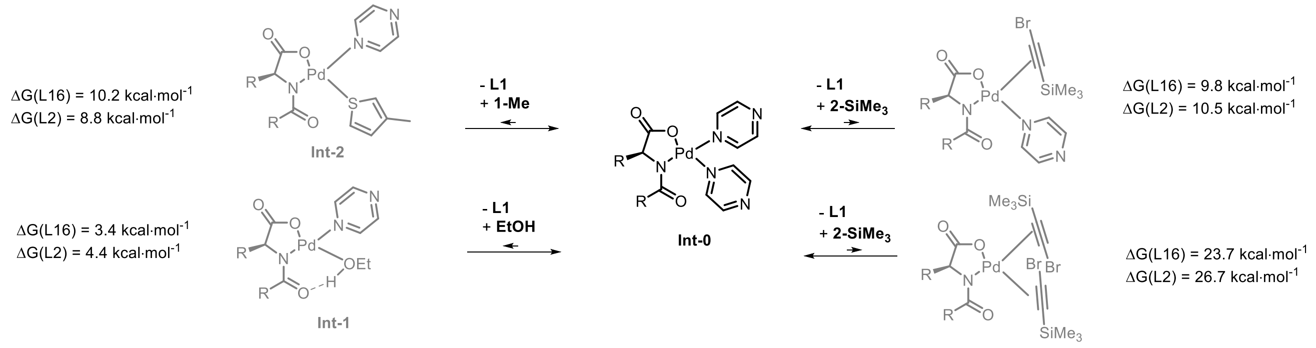

Figure S74: Resting state vs alkyne and alternate monodentate coordination at the dCOSMORS(EtOH) ωB97X-D3BJ/def2-QZVPP//ωB97X-D3BJ/def2-TZVP level of theory.

The experimental observations (with the respective error margins and at the relatively low alkyne concentration) can be qualitatively best explained with the elasticity coefficient and the relative MI and C–H

activation barriers (i.e. rate constants). The higher order in C2 alkyne vs C5 alkyne for L2 is also in line with the elasticity coefficient as discussed above.

Conclusions catalyst:

In a previous publication we derived the order dependency for varying the catalyst loading.[48] Assuming an N-acylamino acid (AA) and monodentate ligand (L) form an active catalyst PdAAL that is in equilibrium with a resting state $PdAAL_2$. We could show in equation 16 the effective concentration in PdAAL as a function of the amount $[Pd]_{tot}$ assuming the experimental Pd:L ratio is 1:2 for all experiments (as done here).

$$[\mathrm{PdAAL}] = \frac{\sqrt{4K[\mathrm{Pd}]_{tot}+1}-1}{2K} \quad \text{with } K = \frac{[\mathrm{PdAAL_2}]}{[\mathrm{PdAAL}][\mathrm{L}]} \tag{16}$$

For larger equilibrium constants *K*, where the equilibrium shifts more strongly to the $PdAAL_2$ complex when both the concentration of palladium and the ligands are increased, an order of 0.5 is expected, whereas for smaller equilibrium constants an order of 1 can be expected in the concentration range under study. In Figure S75a,b we exemplify this by simulating the effective concentration of active catalyst PdAAL as a function of $[Pd]_{tot}$ at high ($1000M^{-1}$ ~ 4.3 kcal/mol at 313K) and low K ($10M^{-1}$ ~ 1.4 kcal/mol) and orders of 0.58 and 0.91 are obtained respectively. The active catalyst would be first order and therefore fitting $[PdAAL]=a\cdot[Pd]_{tot}^{b}$ can yield the order b. Alternatively the order can be obtained by simulating the reaction profiles using COPASI[121] and VTNA. Using the steady-state approximation (i.e. the bulk amount of Pd accumulates before the turnover-limiting step) and a first order reaction PdAAL + substrate -> PdAAL + product similar orders are obtained (see Figure S75c,d).

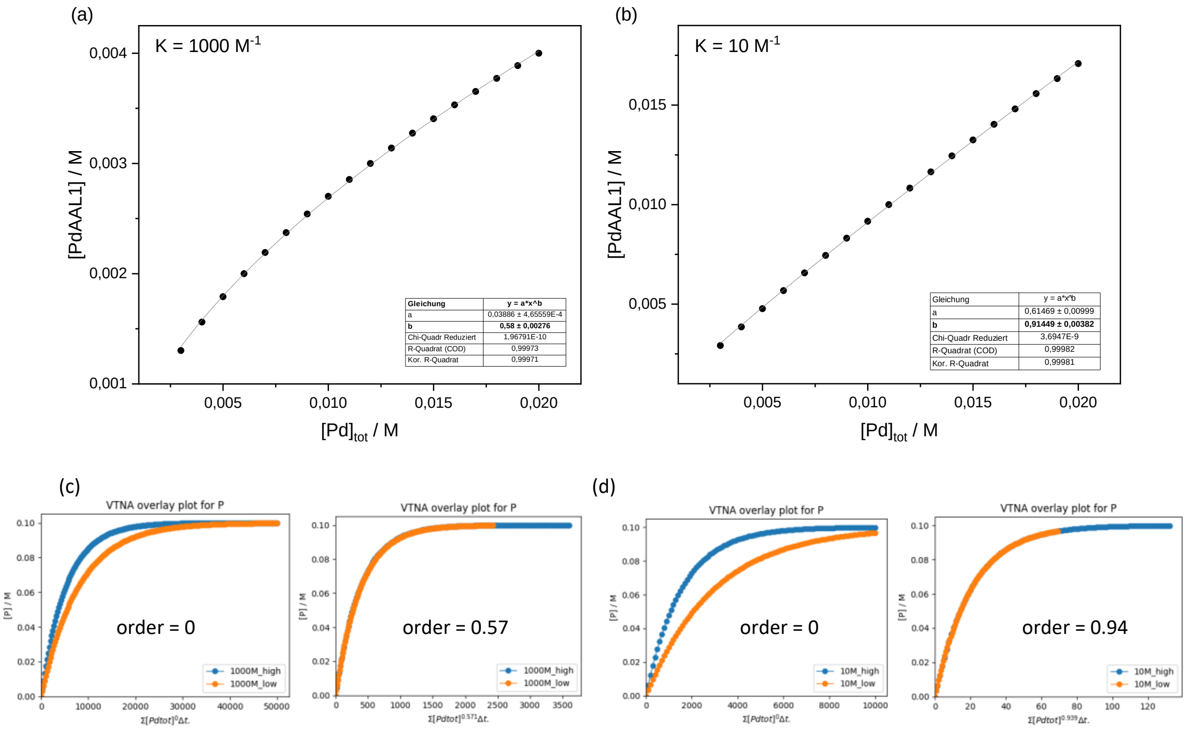

Figure S75: Simulated concentration dependency of active catalyst for (a) high and (b) low K values at a typically used concentration range of $[Pd]_{tot}$. The identical information can be obtained by using simulated t vs concentration curves and VTNA analysis for (a) high and (b) low *K* values.

The same catalyst system with different equilibrium constants between resting state and ligand dissociated catalyst (e.g. possibly **Int-1** or **Int-2**)[13] can therefore show different orders. A dimer-monomer equilibrium with the dimer as active catalyst can be ruled out since orders substantially >1 would be expected.[122] It is established for similar catalytic systems that different mononuclear species (or potentially dinuclear) species can co-exist in a complex equilibrium.[123] We have studied this in our previous study with a near identical catalyst system and all obtained results pointed toward the exclusive presence of mononuclear species.[48] Note that a hypothetical lower-lying resting state would not change the relative MI vs C–H activation barriers since it has a similar influence on both barriers. Dinuclear resting states have mainly been noticed with strong X,L-type bidentate ligands (or substrates with directing groups) and bridging carboxylate groups.[29,124,125] Here the formation of dimers is favored to allow for full occupation of the four Pd-coordination sites while maintaining a neutral charge (no monodentate L-type ligand present). Examples with X,X-type N-acyl amino acids or carboxylate and L-type monodentate pyridines are rare.

Further mechanistic control experiments

In addition to the initial control experiments (see Figure S56), further control experiments were performed to gain deeper mechanistic insights.

A comparison of methyl vs hexyl substituents (Figure S76) shows a shift towards more C2 product. We attribute this to the less steric congestion making the C2 site more favorable compared the bulkier analogue. These values serve as a comparison for selectivities predicted with classical DFT. Ligand **L18** was previously predicted to be better performing than **L16** which was experimentally verified in this screening.

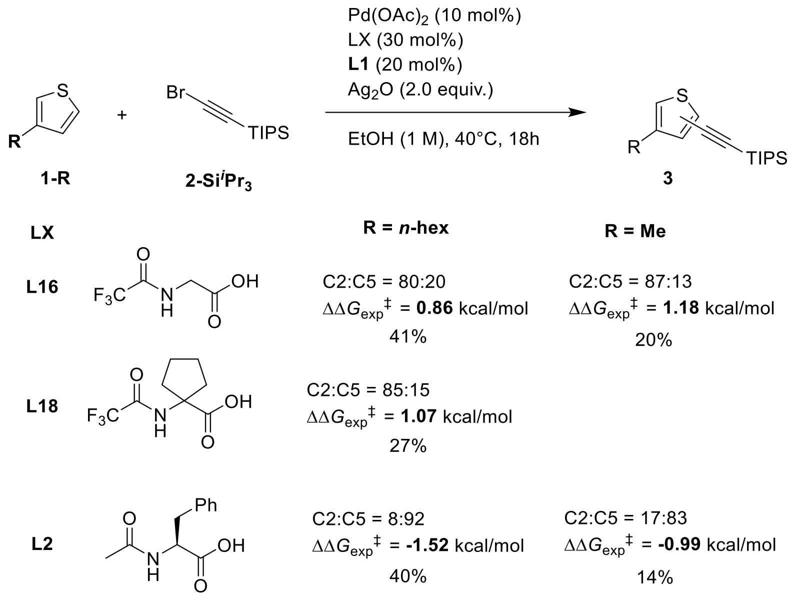

Figure S76: Comparison of the crude reaction of *n*-hexyl and methyl substituted thiophene with the standard ligands to better compare with MLR (hexyl dataset) and DFT calculation (methyl group for simplification). The previously predicted ligand **L18** was test,ed under identical conditions.

[13] In both cases [T] or [EtOH] would be >> [Pd] in the initial regime and can hence be assumed to be constant and therefore part of K.

Due to a certain analogy of the Fujiwara-Moritani reaction with the alkynylation (both are predicted to proceed through the same C–H activation TS followed by a MI), the selectivities were investigated using identical ligands and conditions as shown in Figure S77.

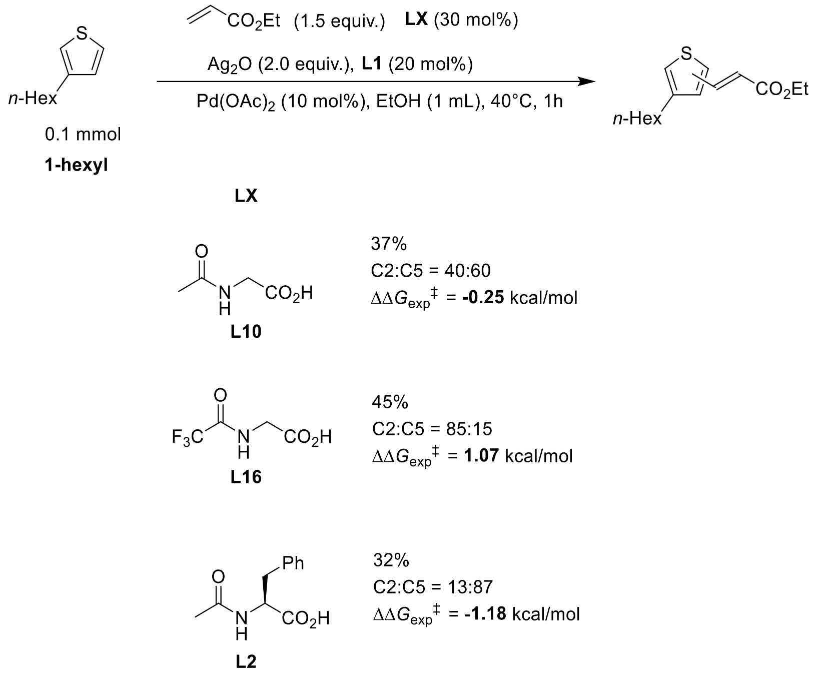

Figure S77: Use of ethyl acrylate instead of alkyne source to compare selectivities and the influence of ligands with a conceptually related reagent in the follow up step.

These results indicate a similar mechanism to be operative.

To elucidate the role of silver several control experiments were conducted (Figure S78). Please note that most results are merely suggestive, since low yields are observed. The catalyst does not necessarily complete one turnover potentially rendering it a stoichiometric reaction which makes comparison with a catalytic system difficult. Several side reactions could explain diverging selectivities and potentially one catalytic cycle could be required to form the catalytically most relevant species.

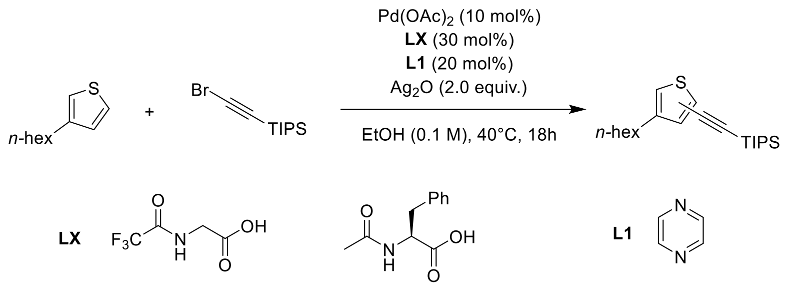

#	L16	L2	Deviation from std. conditions
influence of silver			
**1**	C2:C5 = 80:20<br>41%	C2:C5 = 8:92<br>40%	with Ag_2O (2.0 equiv.)<br>*van Gemmeren, et al.* [102]
**2**	C2:C5 = 74:26<br>4%	C2:C5 = 56:44<br>5%	no Ag_2O
**3**	C2:C5 = 31:69<br>3%	C2:C5 = 40:59<br>4%	no Ag_2O, $Pd(OAc)_2$ 1.0 equiv,<br>Pyrazine 2.0 equiv<br>L1 3.0 equiv, **0.1 M**
**4**	C2:C5 = 79:21<br>6%	C2:C5 = 44:56<br>8%	no Ag_2O, $Pd(OAc)_2$ 1.0 equiv,<br>Pyrazine 2.0 equiv<br>L1 3.0 equiv, **0.01 M**
	13%<br>**69%**	13%<br>**67%**	%Conv Thiophene<br>**%Conv Alkyne**
**5**	C2:C5 = 74:26<br>9.6%	C2:C5 = 27:73<br>9.7%	Ag_2O (10 mol%)
	14%<br>**18%**	25%<br>**23%**	%Conv Thiophene<br>**%Conv Alkyne**
**6**		C2:C5 = 26:74<br>6%	with CsOAc (1.0 equiv.) instead of Ag_2O
**7**		C2:C5 = 13:87<br>32%	with Ag_2O (2.0 equiv)., ethyl acrylate instead of alkyne
**8**		C2:C5 = 50:50<br>4%	without Ag_2O, ethyl acrylate instead of alkyne
**9**		C2:C5 = 24:77<br>9%	with MnO_2 (1.0 equiv.) instead of Ag_2O, ethyl acrylate instead of alkyne
influence of acetate			
**10**	C2:C5 = 82:18<br>35%	C2:C5 = 9:91<br>34%	$Pd(TFA)_2$ instead of $Pd(OAc)_2$
**11**	C2:C5 = 78:22<br>27%	C2:C5 = 23:77<br>38%	$Pd(OPiv)_2$ instead of $Pd(OAc)_2$

Figure S78: Reactions to probe the influence of silver and acetate on the reaction selectivity and yield.

Initially the influence of omitting Ag_2O was probed (Figure S78, 1) and a substantially lower yield was observed for both ligands. In case of **L16** only minor changes in selectivity were observed whereas for **L2** an inversion of selectivity was visible. Silver can potentially take several roles. We hypothesize that it aids the stripping of halide, prevents catalyst poisoning by additional free halide and controls the reaction acidity (formally HBr is liberated in the reaction).

We attempted experiments with stoichiometric amounts of catalyst. With entry 2 having the same concentration in substrate. Very low yields and drastically altered selectivities were observed. We assume that a high concentration in catalyst components can lead to insolubilities and distort speciation equilibria hence preventing the formation of the active catalyst. Therefore, we repeated the experiment at higher dilution which corresponds to stoichiometric catalyst at the originally reported concentration. Here we see low

product formation. This initially appeared to be counterintuitive assuming silver merely acts as a halide scavenger to prevent catalyst poisoning, since with stoichiometric quantities of catalyst a higher conversion would be expected. Upon careful investigation of the reaction mixture, we detected a high conversion (~70% = 0.1 mmol) in alkyne despite the low formation of product (>10%) and low conversion in thiophene (~10%). The remaining alkyne was converted to the homocoupled Glaser-type product (Figure S79). This indicates that silver is responsible for suppressing a competing pathway.

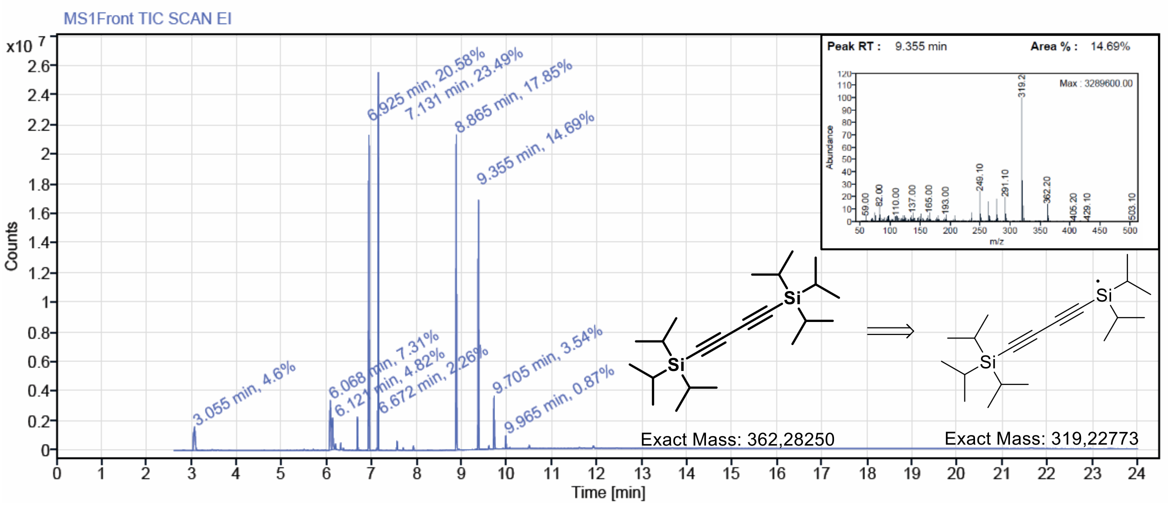

t_{Ret} / min	6.925	7.131	8.865	9.355	9.705	9.965
**Species**	**1**-hexyl (thiophene)	**2-Si(^{i}Pr)$_3$** (alkyne)	internal standard	**4** homocoupled alkyne	**3** Product C2	**3** Product C5

Figure S79: GC-MS trace for a reaction with stoichiometric catalyst in the absence of silver.

Adding a catalytic amount of silver partially restored the selectivity but only slightly improved the yield (strictly speaking not above 1 TON). Around 20% of alkyne (~0.03 mmol) were consumed. This is in line with one catalyst turnover (0.01 mmol) and two subsequent turnovers (0.02 mmol) enabled by restoring the catalyst with Ag^+ (0.02 mmol).

To probe the influence of other halide scavenging agents, we employed CsOAc that has been reported in other reactions to replace silver and we indeed could see an improved C5 selectivity for **L2** in entry 6 Figure S78.[104] However, no restoration of the yield was observed. This can either be due to a lower halide scavenging ability (higher solubility in EtOH) and/or due to the additional oxidizing ability of Ag^+ that can readily re-oxidize potentially formed Pd^0.

We additionally determined the kinetic order of silver to be 0 when using **L2** (see Figure S80). Please note that due to the heterogeneous nature of the silver salt an order determination can be difficult since the effective concentration in solution is unknown. A saturation in soluble silver for low conversions with high silver loadings hence the zeroth order is merely suggestive of a non-involvement of silver in the turnover-limiting step but not conclusive.

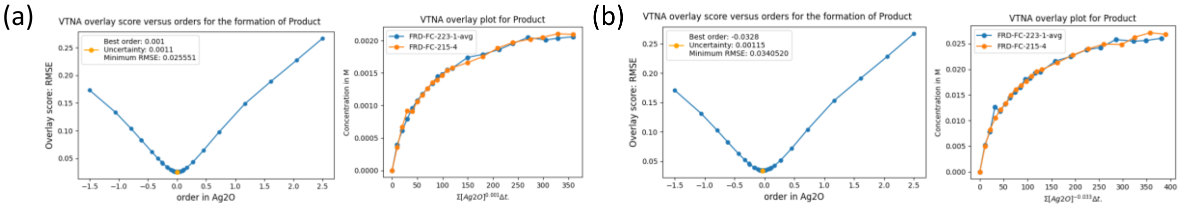

Figure S80: Order determination via Auto-VTNA for **L2** using standard conditions (0.2 M Ag_2O) and lower silver loading (0.1 M Ag_2O).

To probe the influence of Ag_2O on the related Fujiwara-Moritani reaction, we carried out similar control reactions (see Figure S78). Here silver is assumed to be relevant as the oxidizing agent. The omission of silver also led to a deterioration in yield and C5 selectivity for **L2**. Both can be restored to some extent by switching to an alternate oxidizing agent like MnO_2.

We interpret the combined result of Figure S78 in a way that silver is indeed crucial for enabling a catalytic turnover and it takes several roles depending on the reagent (acrylate vs haloalkyne). It is likely not involved in the turnover limiting step but in a follow-up step to allow for catalyst regeneration or to avoid detrimental catalyst poisoning. Selectivity and yield can, to some extent be restored by using a species that replaces the hypothesized task silver: namely halide-scavenger or oxidant. Please note that the reaction conditions are not optimized for the respective silver replacement and potentially higher yields or selectivities could be achieved upon employing altered reaction conditions. Be referred to the Supporting Information of our work on heteroarene olefination,[126] where replacing silver with other oxidants like MnO_2, oxone, or benzoquinone only led to a minor decrease in yield and identical selectivities for 3-hexylthiophene. Also, a stoichiometric reaction in catalyst without silver was performed in our related arene olefination. Here 1,1,1,3,3,3-hexafluoropropan-2-yl benzoate was olefinated under standard conditions in 46% yield and in 39% yield under stoichiometric, silver free conditions.[14] This illustrates that for the olefination no suppression of a side reaction is needed and silver acts exclusively as terminal oxidant.

The role of acetate in the reaction was also probed by replacing $Pd(OAc)_2$ with the respective trifluoroacetate and pivalate analogs (Figure S78, 10&11). Based on common acidity trends, the N-acyl amino acid should be in between trifluoracetate and pivalate in terms of binding strength and acidity.

The results show that the reaction is not very sensitive to the precise nature of the carboxylate anion, giving comparable results in the presence of pivalate, acetate, and trifluoroacetate anions in terms of yield and selectivity. This is in line with the key role of the externally added bidentate N-acyl amino acid ligand in enabling the desired reactivity.

[14] The slightly altered selectivities (o:m:p 18:70:12 standard vs 20:59:21 Ag free, stoichiometric cat.) can likely be attributed to concentration effects ([Pd] 0.01 M standard vs 0.1 M Ag free, stoichiometric cat) and the stronger Lewis acidity of silver which can coordinate the ester rendering a *meta*-functionalization more favorable according to classical eCMD/BIES selectivities.

4.4 $^{12}C/^{13}C$ KIE

High conversion sample

An oven-dried Schlenk tube (10 mL) was charged with a magnetic stirring-bar. Then $Pd(OAc)_2$ (4.5 mg, 0.020 mmol, 10 mol%), *N*-Acetyl-L-phenylalanine (12.3 mg, 0.030 mmol, 30 mol%), and Ag_2O (92.6 mg, 0.20 mmol, 2.0 equiv.) were added. Pyrazine (3.2 mg, 0.020 mmol, 20 mol%) was added last due to volatility concerns. EtOH (1.0 mL) was added. (Bromoethynyl)triisopropylsilane (78 mg, 0.05 mmol, 0.25 equiv.) and 3-hexyl thiophene (34 mg, 0.20 mmol) were added followed by EtOH (1.0 mL). The tube was tightly sealed and stirred in a pre-heated metal block at 40°C for 18h. The reaction was set-up three times in parallel. A defined amount of trimethyl 1,3,5-benzenetricarboxylate was added as internal standard. The reaction mixture was diluted with EtOAc, filtered through a plug of silica, and eluted with EtOAc. A small aliquot of the resulting solution was submitted to GC-FID analysis and the remaining solvent was removed under reduced pressure. The crude product was filtered through a short plug of silica eluting with *n*-pentane. The product was separated from lower-boiling compounds by Kugelrohr distillation (12 mbar, 140°C). The leftover fraction was subjected to preparative TLC (SiO_2, *n*-pentane). The high conversion sample was obtained as a slightly yellow liquid (crude: conv. Alkyne 97%, yield 55%, C2:C5 = 6:94, isolated: 35.7 mg, 17%).

Low conversion sample

An oven-dried Schlenk tube (10 mL) was charged with a magnetic stirring-bar. Then $Pd(OAc)_2$ (4.5 mg, 0.020 mmol, 10 mol%), *N*-Acetyl-L-phenylalanine (12.3 mg, 0.030 mmol, 30 mol%), and Ag_2O (92.6 mg, 0.20 mmol, 2.0 equiv.) were added. Pyrazine (3.2 mg, 0.020 mmol, 20 mol%) was added last due to volatility concerns. EtOH (1.0 mL) was added. (Bromoethynyl)triisopropylsilane (78 mg, 0.05 mmol, 0.25 equiv.) and 3-hexyl thiophene (34 mg, 0.20 mmol) were added followed by EtOH (1.0 mL). The tube was tightly sealed and stirred in a pre-heated metal block at 40°C for 10min. The reaction was set-up twelve times in parallel. EtOAc was added to dilute and thereby quench the reactions and a defined amount of trimethyl 1,3,5-benzenetricarboxylate was added as internal standard. The combined reactions were filtered through a plug of silica and eluted with EtOAc. A small aliquot of the resulting solution was submitted to GC-FID analysis and the remaining solvent was removed under reduced pressure. The crude product was filtered through a short plug of silica eluting with *n*-pentane. The product was separated from lower-boiling compounds by Kugelrohr distillation (12 mbar, 140°C). The leftover fraction was subjected to preparative TLC (SiO_2, *n*-pentane). The low conversion sample was obtained as a slightly yellow liquid (crude: conv Alkyne 24%, yield 15%, C2:C5 = 7:93, isolated: 60.9 mg, 7%).

^1H NMR (600 MHz, $CDCl_3$) δ 7.08 – 7.05 (m, 1H), 6.82 – 6.79 (m, 1H), 2.57 – 2.51 (m, 2H), 1.63 – 1.54 (m, 2H), 1.35 – 1.27 (m, 6H), 1.15 – 1.10 (m, 21H), 0.91 – 0.86 (m, 3H) ppm.

^{13}C NMR (151 MHz, $CDCl_3$) δ 143.2, 133.8, 123.3, 122.0, 100.0, 94.8, 31.8, 30.5, 30.4, 29.1, 22.7, 18.8, 14.2, 11.5 ppm.

HRMS (ESI) m/z calcd. for $C_{21}H_{37}SSi^+$ $[M+H]^+$: 349.23852; found: 349.23749.

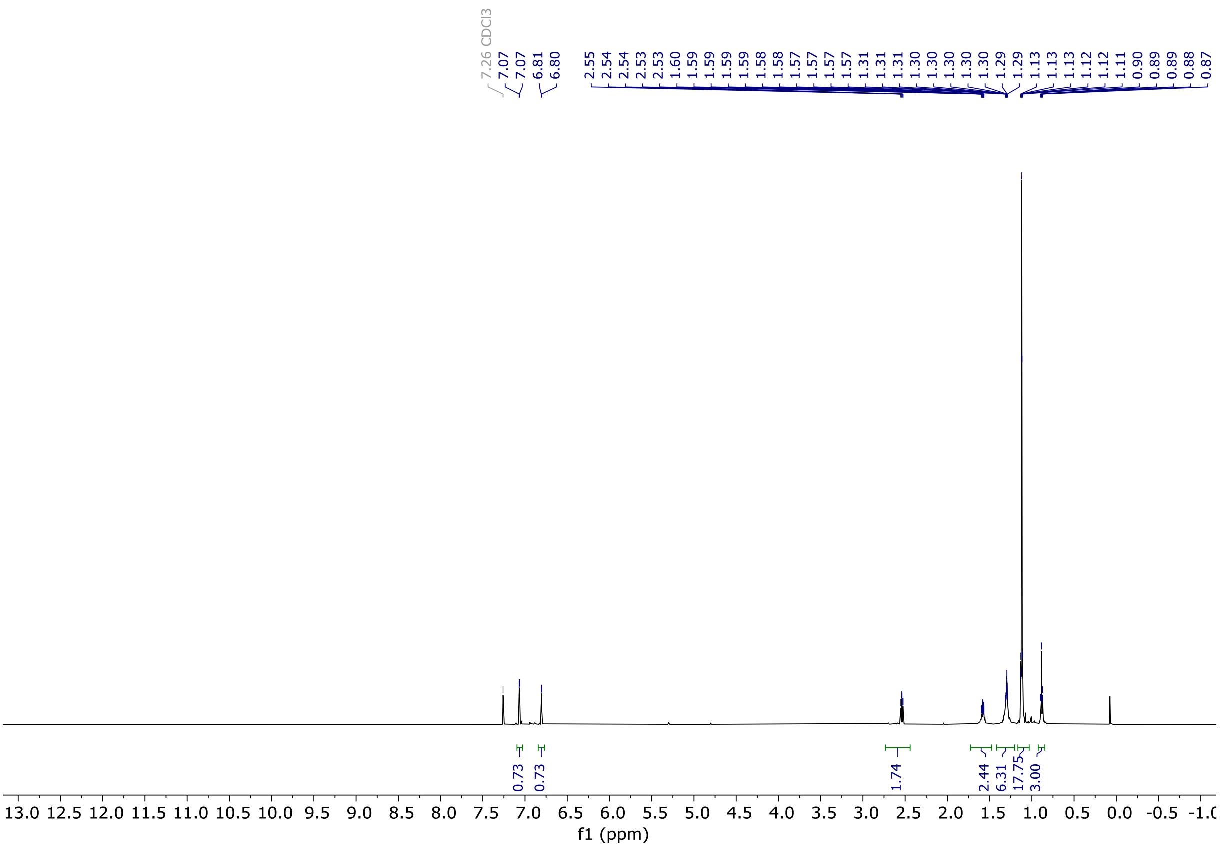

Figure S81: ^{1}H-NMR in $CDCl_3$ of the high conversion sample.

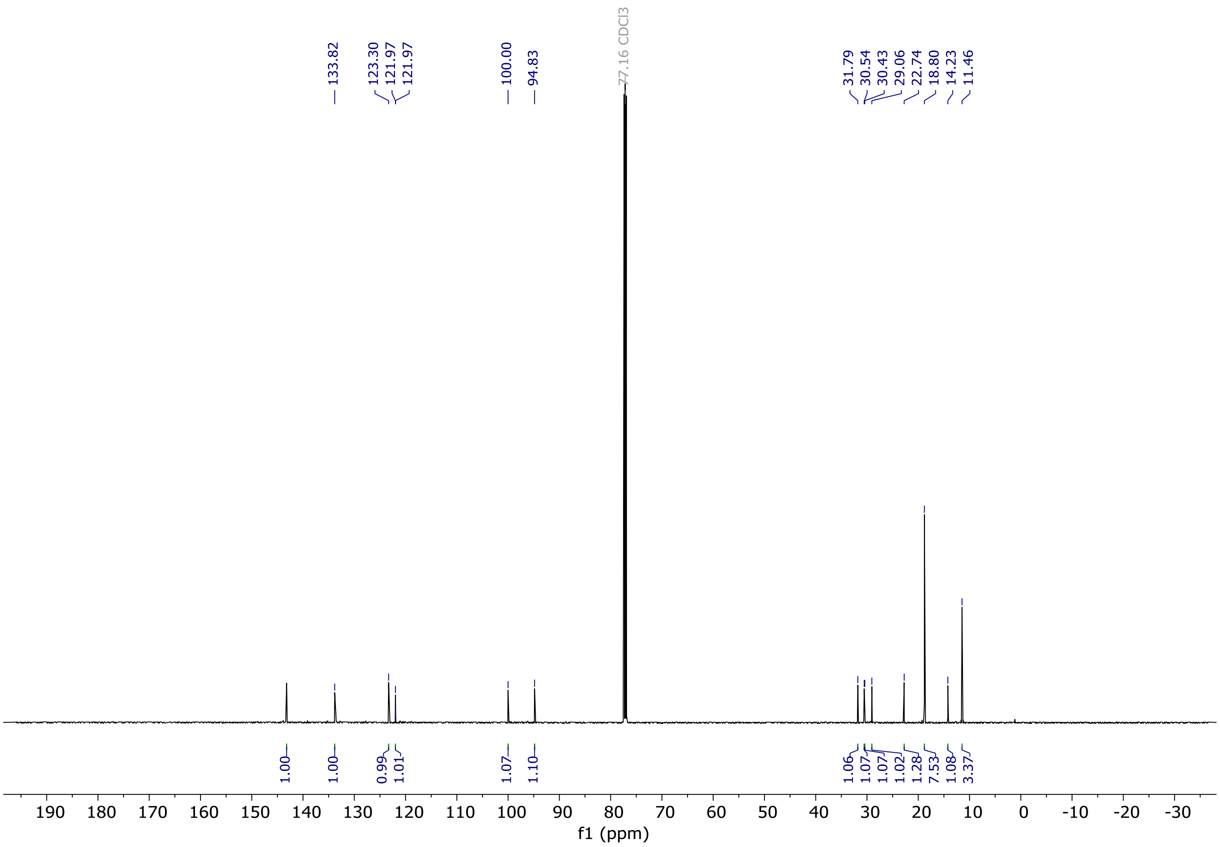

Figure S82: Quantitative $^{13}C\{^1H\}$-NMR in $CDCl_3$ of the high conversion sample.

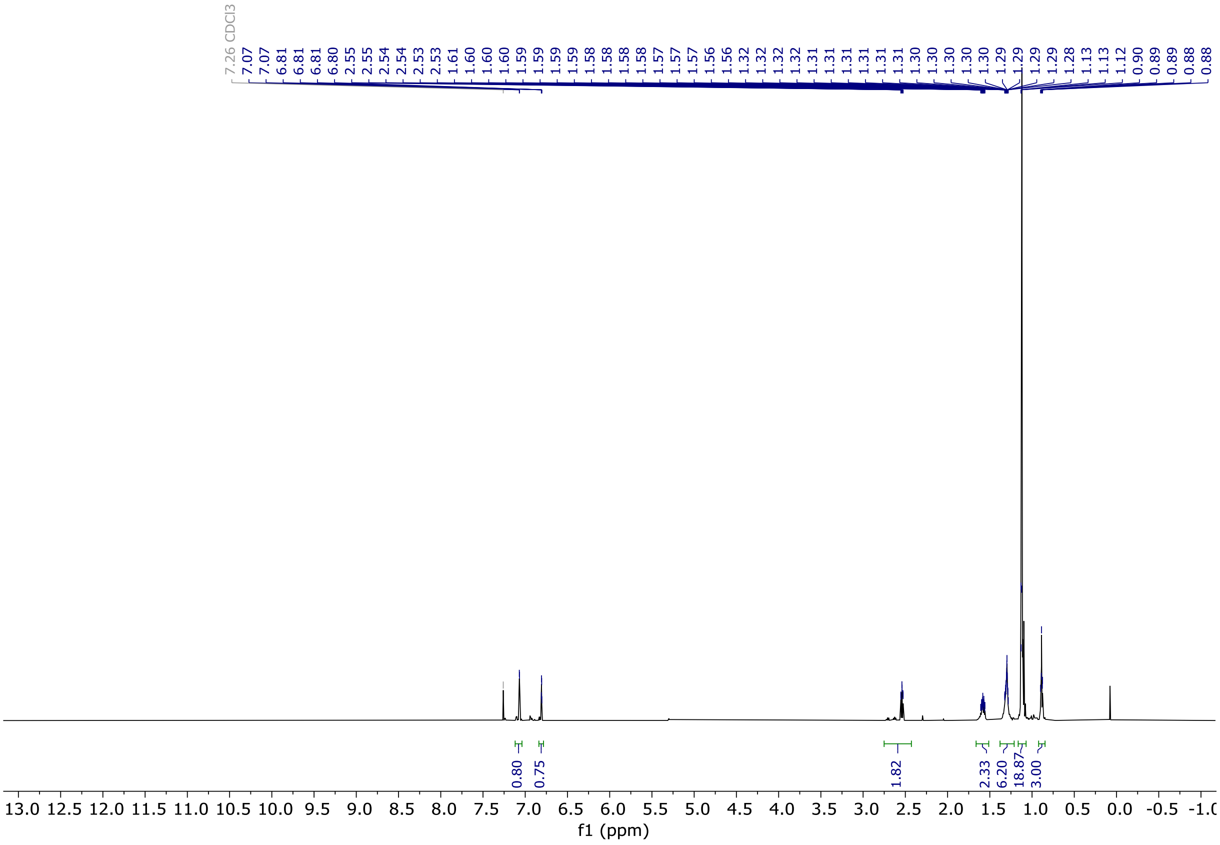

Figure S83: ^{1}H-NMR in $CDCl_3$ of the low conversion sample.

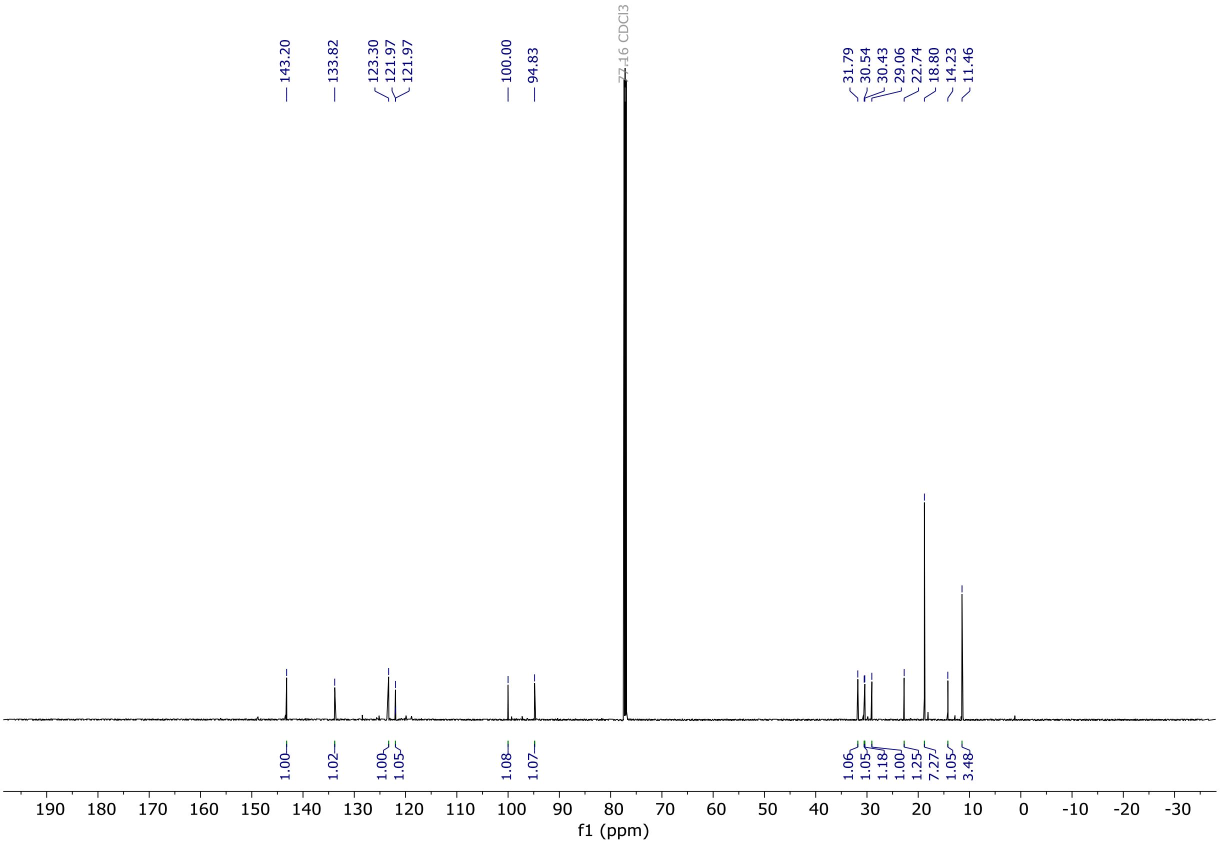

Figure S84: Quantitative $^{13}C\{^1H\}$-NMR in $CDCl_3$ of the low conversion sample.

Initially, the product was fully assigned using ^{1}H, $^{13}C\{^{1}H\}$, mult.ed-HSQC and HMBC experiments (see Figure S86a and Table S24.

Subsequently, an inversion recovery experiment with inverse gated decoupling (t1irig) was used to estimate T_1 relaxation delays for the molecule of interest. Delays τ of 0.05, 1, 2, 3, 4, 5, 6, 7, 8, 10, 20s a sufficiently long relaxation delay (d1 = 30s) and four scans (ns = 4) per experiment were chosen.

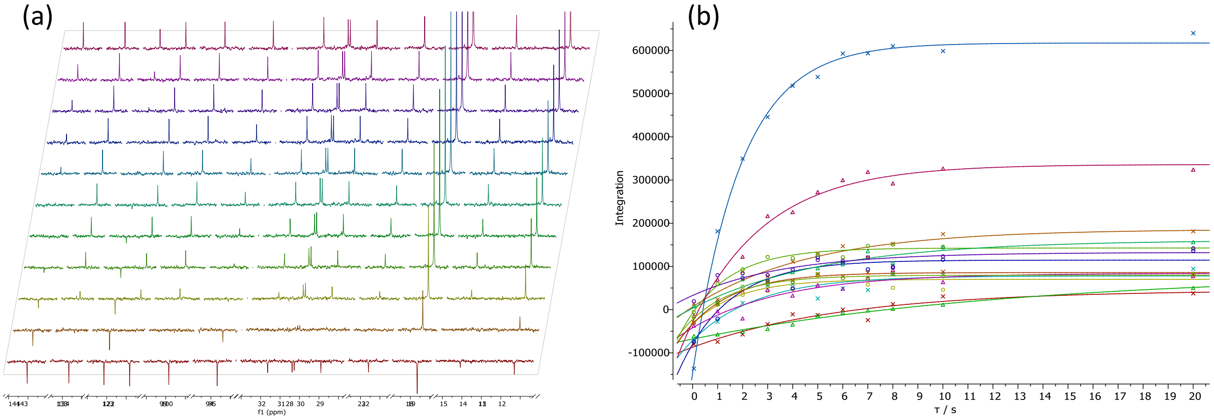

Figure S85: (a) Stacked spectra of inversion recovery experiment with zero crossings for all signals of interest. (b) Fitted integration vs delay τ with a three-parameter exponential fit.

The spectra were processed (baseline correction, zero filling, line broadening), integrated and fitted via a three-parameter exponential fit to determine T_1. The measured times are shown in Figure S86b with the slowest relaxing nuclei having a T_1 of 13.2 s.

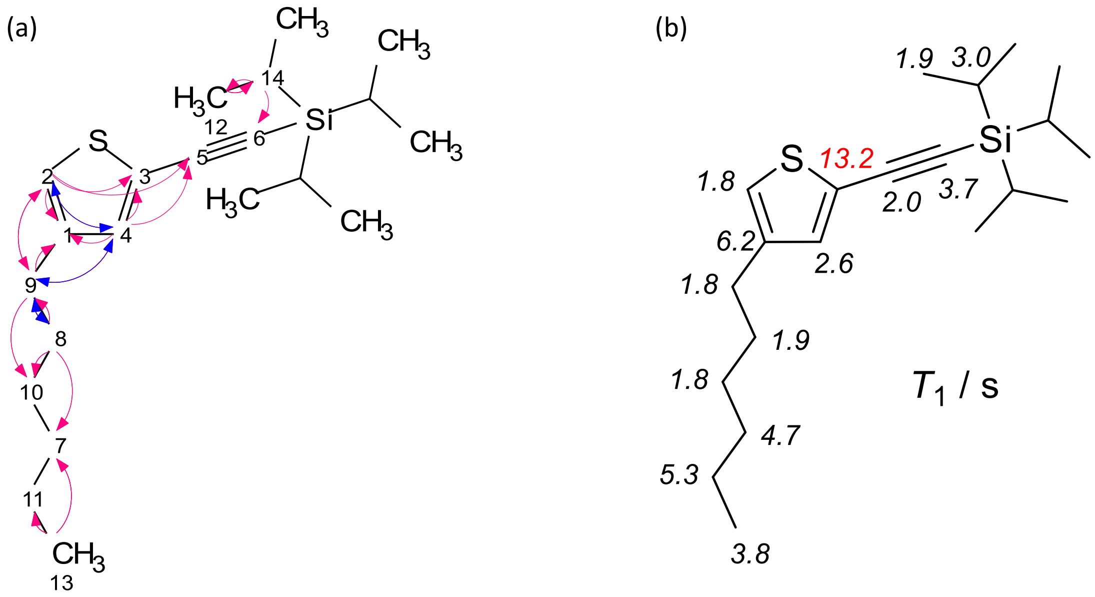

Figure S86: a) COSY (blue) and HMBC (pink) correlations used for assignment with the numbering displayed in Table S24b)

Table S24: Chemical shits and 2D NMR correlations used for the full assignment of ((4-hexylthiophen-2-yl)ethynyl)triisopropylsilane.

Atom / #	δ (ppm)	COSY	HSQC	HMBC	T_1 / s
1 C	143.2			2, 4, 9	6.2
2 C	133.92		2	4, 9	1.8
H	7.07	4	2	1, 3, 4, 5, 9	
3 C	123.3			2, 4	13.2
4 C	122.05		4	2, 9	2.6
H	6.81	2, 9	4	1, 2, 3, 5, 9	
5 C	100			2, 4	2.0
6 C	94.83			14	3.7
7 C	31.82		7	8, 13	4.7
H2	1.29		7		
8 C	30.59		8	9	1.9
H2	1.58	8	8	7, 9, 10	
9 C	30.41		9	2, 4, 8	1.8
H2	2.54		9	1, 2, 4, 8, 10	
10 C	29.09		10	8, 9	1.8
H2	1.33		10		
11 C	22.76		11	13	5.3
H2	1.3		11		
12 C	18.8		12	14	1.9
H3	1.12		12	14	
13 C	14.23		13		3.8
H3	0.89		13	7, 11	
14 C	11.51		14	12	3.0
H	1.12		14	6, 12	

For the $^{12}C/^{13}C$ KIE experiment, the samples (35.7 mg) were filled in a J-Young NMR tube in $CDCl_3$ (0.65 mL). The first sample was loaded, matched and tuned (atmm) and subjected to automatic shimming. After measurement of the first sample, the probe was not re-matched or re-tuned but re-shimmed.

We initially opted for a quantitative $^{13}C\{^1H\}$ experiment using inverse gated decoupling and a 30° pulse, an acquisition time (aq = 2.0s) and a sufficiently long relaxation delay (d1 = 28s), which would allow an effective relaxation of 30s and 128 scans. The observer nuclei offset was set to 80 ppm (240 ppm spectra window) and the ^1H decoupler offset to 4 ppm.

We recorded additional 4 spectra with a delay d1 = 110s, since we assumed the initial results were not quantitative, which later turned out to be an issue of a suitable processing and setting of integrals due to minor

side-product peaks (see Figure S87). The 4 spectra with longer relaxation delay were used, since they result from an identical run of measurements (no additional tuning, shimming, etc.).

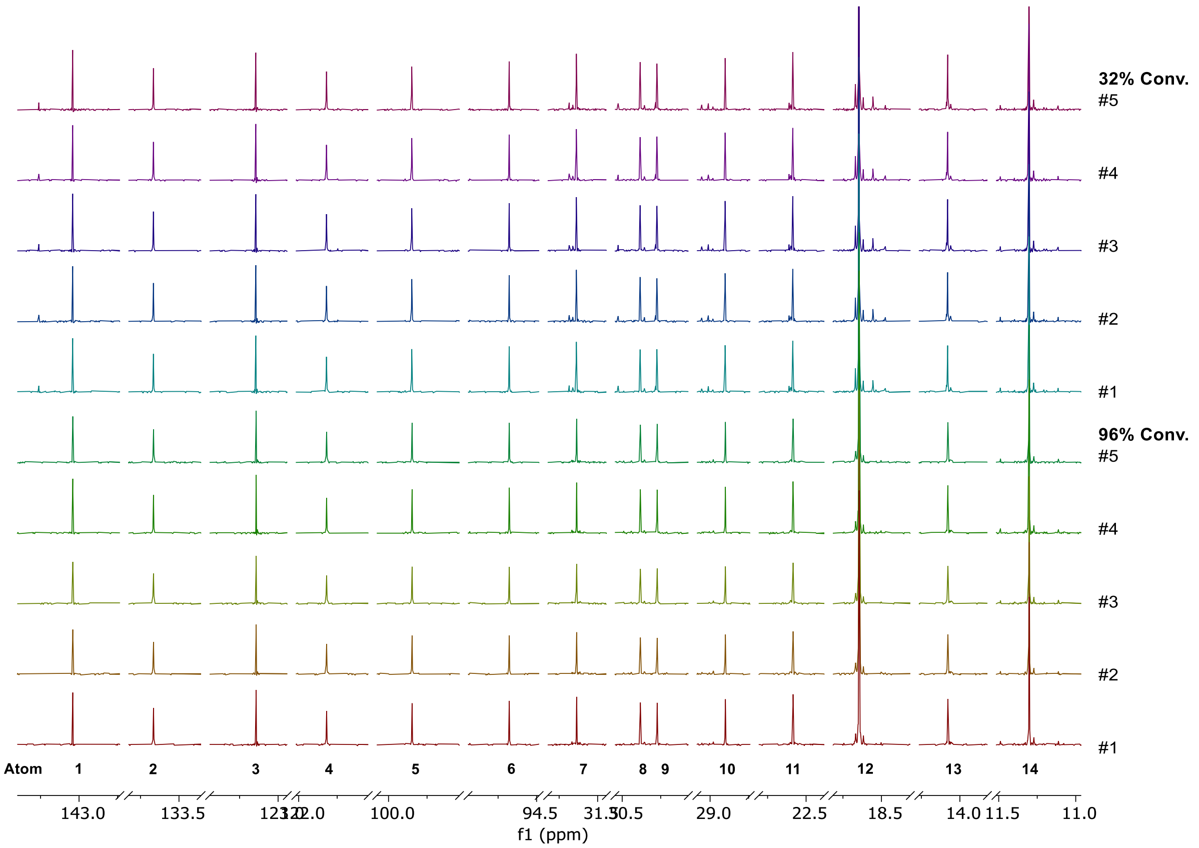

Figure S87: Overview of the quantitative $^{13}C\{^{1}H\}$ spectra with all relevant peaks.

The spectra were phased in Topspin[127] and transferred to MNova[128] processed and integrated. Here linear prediction was applied (1024k datapoints) to insure sharp lines for potential use of deconvolution algorithms. A negative exponential weighting function (-0.05) and a positive Gaussian weighting function (0.3) were applied. Due to this processing sufficiently large (S/N < 70 for signals of interest) but narrow (line-width at half-height ~0.5 Hz) signals could be obtained. It proved to be crucial to set narrow integrals, since especially in the aliphatic region overlap with the minor isomer and traces of thiophene starting material were observed. A 5th order Bernstein polynomial baseline correction was applied between -20 and 170 ppm (see integrals in Table S25).

Several integration settings with narrower and wider integration (sum integration as implemented in MNova), different weighting function and baseline correction algorithms as well as the GSD deconvolution were tested. The influence on the final relative KIE values between the alkyne carbons and the main conclusion (KIE (100.0 ppm) > KIE (94.8 ppm)) were found to be small. The processing procedure described here nevertheless leads to reduced standard errors. The integrals (~±2 Hz from the central line) were set for one spectrum and copied to all other spectra to remove integration bias.

The KIEs were calculated according to eq. 17 with *R* being the normalized integral of interest at fractional conversion (low conversion), R_0 being a normalized integral of product (high conversion) and *F* being the conversion in alkyne for the low conversion sample as described by Jacobsen, Kwan, et al.[129]

$$\mathrm{KIE} = \frac{\ln(1-F)}{\ln\left(1 - F\left(\frac{R}{R_0}\right)\right)} \tag{17}$$

Singleton et al.,[130] detailed the derivation of uncertainty in F and R/R_0 for the determination of KIE with residual starting material at high conversion. We derived the error in F and R/R_0 accordingly in the equation 18 and 19 but for product at low conversion. To achieve this a derivation for eq. 17 with F and R/R_0 is done respectively.

$$\sigma(\mathrm{KIE}_F) = \frac{\partial \mathrm{KIE}}{\partial F}\sigma(F) = $$
$$= \frac{\frac{R}{R_0}\cdot\ln(1-F)}{\left(1-\frac{R}{R_0}\cdot F\right)\cdot\ln^2\left(1-\frac{R}{R_0}\cdot F\right)} - \frac{1}{(1-F)\cdot\ln\left(1-\frac{R}{R_0}\cdot F\right)}\cdot\sigma(F) \tag{18}$$

$$\sigma(\mathrm{KIE}_{\frac{R}{R_0}}) = \frac{\partial \mathrm{KIE}}{\partial \frac{R}{R_0}}\sigma\left(\frac{R}{R_0}\right) = \frac{F\cdot\ln(1-F)}{\left(1-\frac{R}{R_0}\cdot F\right)\cdot\ln^2\left(1-\frac{R}{R_0}\cdot F\right)}\cdot\sigma\left(\frac{R}{R_0}\right) \tag{19}$$

Assuming F and R/R_0 to be independent leads to an overall uncertainty of in eq. 20 according to general uncertainty propagation.

$$\sigma(\mathrm{KIE}) = \sqrt{\left(\frac{\partial \mathrm{KIE}}{\partial F}\right)^2\sigma^2(F) + \left(\frac{\partial \mathrm{KIE}}{\partial \frac{R}{R_0}}\right)^2\sigma^2\left(\frac{R}{R_0}\right)} \tag{20}$$

$\sigma(R/R_0)$ is estimated as sample standard error SE of the mean of the four separate NMR experiments (n=4) (Table S25) run per sample (eq. 21) with $\bar{x}$ being the sample mean, x_i the individual datapoint, and n the number of measurements.

$$SE = \frac{SD_{\mathrm{sample}}}{\sqrt{n}} = \sqrt{\frac{\sum(x_i-\bar{x})^2}{n-1}}\cdot\frac{1}{\sqrt{n}} \tag{21}$$

$\sigma(F)$ is estimated to be 2% of the actual conversion (32%). Both are rather conservative error estimates. $\sigma(R/R_0)$ and $\sigma(F)$ in combination with eq. 20 can be used to estimate the uncertainty in the final KIE values (see Figure S28).

Table S25: Integrals obtained from the low and high conversion sample using an inverse gated $^{13}C\{^1H\}$ experiment.

Atom	5	6	7	8
**97% Conv.**			**Ref**	
#1	0.944936	0.955403	1.000000	0.982366
#2	0.955789	0.944593	1.000000	0.980871
#3	0.947902	0.924430	1.000000	0.979792
#4	0.942179	0.932359	1.000000	0.998095
#5*	0.936211	0.920443	1.000000	1.005754
**32% Conv.**			**Ref**	
#1	0.929369	0.953201	1.000000	0.983128
#2	0.928046	0.938313	1.000000	1.003772
#3	0.919353	0.943327	1.000000	1.004373
#4	0.900609	0.939863	1.000000	0.993655
#5*	0.892263	0.905357	1.000000	0.985678

* d_1 = 28s instead of 110s

Regardless of processing (baseline correction, weighting function, etc.), the nucleus 5 (δ = 100.0 ppm) showed KIE values between ~1.03 – 1.05 and was always higher than nucleus 6 (δ = 94.8 ppm) that showed values between ~0.98 – 1.02. Higher values in one nucleus led to higher values in the respective with similar differences between both.

We also compared different nuclei as reference nucleus. The methylene carbon 7 (31.8 ppm) showed the least error during all our previous processing attempts and is expected to show no KIE itself (see Figure S28). The methyl and methine carbons of the TIPS group were neither reliable as a reference nor for a KIE to be determined due to hidden signals, likely from the other isomer or a small impurity.

Table S26: KIE as a function of the reference nuclei with the respective errors.

**Atom**	**5**		**6**	
**Reference Nucleus**	**KIE**	***σ*(KIE)**	**KIE**	***σ*(KIE)**
**31.8**	**1.038**	**± 0.007**	**0.994**	**± 0.007**
30.5	1.052	± 0.011	1.01	± 0.011

Singleton pointed out the advantages of determining heavy isotope KIEs based on residual starting material owing to a reduced error.[130] Due to potential side reaction (e.g. traces of side product formation formation), we assumed that looking at product formation would experimentally yield more meaningful results despite the larger experimental error and was shown in several studies to also give trustworthy results.[129] We also want to state that the errors on our ^{13}C KIEs are comparably high, but significant enough to a) observe a non-unit KIE indicating that under these conditions (low concentration in alkyne with C5-selective ligand **L2**) the alkyne involving step is comparably high in energy (C–H activation does not involve alkyne) and b) discard a potential inverse MI with subsequent silyl migration (see Figure S34). Further statements regarding the nature of the TS should be considered with the respective care warranted by the experimental error.

5 References

[1] F. Neese, *WIREs Comput. Mol. Sci.* **2012**, *2*, 73.

[2] F. Neese, F. Wennmohs, U. Becker, C. Riplinger, *J. Chem. Phys.* **2020**, *152*, 224108.

[3] F. Neese, *WIREs Comput. Mol. Sci.* **2022**, *12*.

[4] F. Neese, *WIREs Comput. Mol. Sci.* **2018**, *8*.

[5] W. Humphrey, A. Dalke, K. Schulten, *J. Mol. Graph.* **1996**, *14*, 33-38.

[6] *The PyMOL Molecular Graphics System*. *2.5.1*, Schrödinger LLC.

[7] M. D. Hanwell, D. E. Curtis, D. C. Lonie, T. Vandermeersch, E. Zurek, G. R. Hutchison, *J. Cheminform.* **2012**, *4*, 17.

[8] C. Bannwarth, E. Caldeweyher, S. Ehlert, A. Hansen, P. Pracht, J. Seibert, S. Spicher, S. Grimme, *WIREs Comput. Mol. Sci.* **2021**, *11*.

[9] S. Grimme, C. Bannwarth, P. Shushkov, *J. Chem. Theory Comput.* **2017**, *13*, 1989.

[10] P. Pracht, F. Bohle, S. Grimme, *Phys. chem. chem. phys.* **2020**, *22*, 7169.

[11] F. Weigend, R. Ahlrichs, *Phys. chem. chem. phys.* **2005**, *7*, 3297.

[12] A. Altun, F. Neese, G. Bistoni, *J. Chem. Theory Comput.* **2020**, *16*, 6142.

[13] F. Neese, E. F. Valeev, *J. Chem. Theory Comput.* **2011**, *7*, 33.

[14] B. C. Haas, D. Kalyani, M. S. Sigman, *Sci. Adv.* **2025**, *11*, eadt3013.

[15] A. Verloop, W. Hoogenstraaten, J. Tipker in *Medicinal Chemistry: A Series of Monographs*. *Drug Design* (Ed.: E. J. Ariëns), Elsevier, **1976**, pp. 165–207.

[16] A. Verloop in *Pesticide Chemistry: Human Welfare and Environment*, Elsevier, **1983**, pp. 339–344.

[17] K. C. Harper, E. N. Bess, M. S. Sigman, *Nat. Chem.* **2012**, *4*, 366.

[18] A. V. Brethomé, S. P. Fletcher, R. S. Paton, *ACS Catal.* **2019**, *9*, 2313.

[19] Guilian Luchini, Toby Patterson, Robert Paton, *DBSTEP*. *DFT Based Steric Parameters*, Zenodo, **2023**.

[20] E. D. Glendening, J. K. Badenhoop, A. E. Reed, J. E. Carpenter, J. A. Bohmann, C. M. Morales, P. Karafiloglou, C. R. Landis, F. Weinhold, *NBO 7.0*, Theoretical Chemistry Institute, University of Wisconsin, Madison, WI, **2018**.

[21] E. D. Glendening, C. R. Landis, F. Weinhold, *J. Comput. Chem.* **2019**, *40*, 2234.

[22] H. Shalit Peleg, A. Milo, *Angew. Chem. Int. Ed.* **2023**, *135*.

[23] The MathWorks Inc., *MATLAB R2023b*, The MathWorks Inc., Natick, Massachusetts, United States, **2023**.

[24] J.-Y. Guo, Y. Minko, C. B. Santiago, M. S. Sigman, *ACS Catal.* **2017**, *7*, 4144.

[25] R. Kohavi (Ed.) *Proceedings of the Fourteenth International Joint Conference on Artificial Intelligence (II)*, *Vol. 95*, Internation Joint Conferences on Aritificial Intelligence Organization, **1995**.

[26] A. Cherkasov, E. N. Muratov, D. Fourches, A. Varnek, I. I. Baskin, M. Cronin, J. Dearden, P. Gramatica, Y. C. Martin, R. Todeschini et al., *J. Med. Chem.* **2014**, *57*, 4977.

[27] K. Wu, N. Lam, D. A. Strassfeld, Z. Fan, J. X. Qiao, T. Liu, D. Stamos, J.-Q. Yu, *Angew. Chem. Int. Ed.* **2024**, *63*, e202400509.

[28] J.-L. Yan, L. Hu, Y. Lu, J.-Q. Yu, *J. Am. Chem. Soc.* **2024**, *146*, 29311.

[29] Y.-F. Yang, G. Chen, X. Hong, J.-Q. Yu, K. N. Houk, *J. Am. Chem. Soc.* **2017**, *139*, 8514.

[30] F. Neese, *WIREs Comput. Mol. Sci.* **2025**, *15*.

[31] M. Bursch, J.-M. Mewes, A. Hansen, S. Grimme, *Angew. Chem. Int. Ed.* **2022**, *61*, e202205735.

[32] S. Grimme, *Chem. Eur. J.* **2012**, *18*, 9955.

[33] J. N. Harvey, F. Himo, F. Maseras, L. Perrin, *ACS Catal.* **2019**, *9*, 6803.

[34] V. Barone, M. Cossi, *J. Phys. Chem. A* **1998**, *102*, 1995.
[35] A. V. Marenich, C. J. Cramer, D. G. Truhlar, *J. Phys. Chem. B* **2009**, *113*, 6378.
[36] S. Sinnecker, A. Rajendran, A. Klamt, M. Diedenhofen, F. Neese, *J. Phys. Chem. A* **2006**, *110*, 2235.
[37] A. Klamt, G. Schüürmann, *J. Chem. Soc., Perkin Trans. 2* **1993**, 799.
[38] A. Klamt, V. Jonas, T. Bürger, J. C. W. Lohrenz, *J. Phys. Chem. A* **1998**, *102*, 5074.
[39] F. Eckert, A. Klamt, *AICHE J.* **2002**, *48*, 369.
[40] R. Ahlrichs, M. Bär, M. Häser, H. Horn, C. Kölmel, *Chem. Phys. Lett.* **1989**, *162*, 165.
[41] S. G. Balasubramani, G. P. Chen, S. Coriani, M. Diedenhofen, M. S. Frank, Y. J. Franzke, F. Furche, R. Grotjahn, M. E. Harding, C. Hättig et al., *J. Chem. Phys.* **2020**, *152*, 184107.
[42] Y. J. Franzke, C. Holzer, J. H. Andersen, T. Begušić, F. Bruder, S. Coriani, F. Della Sala, E. Fabiano, D. A. Fedotov, S. Fürst et al., *J. Chem. Theory Comput.* **2023**, *19*, 6859.
[43] C. Zhao, R. Wu, S. Zhang, X. Hong, *J. Phys. Chem. A* **2023**, *127*, 6791.
[44] S. Dohm, A. Hansen, M. Steinmetz, S. Grimme, M. P. Checinski, *J. Chem. Theory Comput.* **2018**, *14*, 2596.
[45] L. Goerigk, S. Grimme, *Phys. chem. chem. phys.* **2011**, *13*, 6670.
[46] M. A. Iron, T. Janes, *J. Phys. Chem. A* **2019**, *123*, 3761.
[47] M. Steinmetz, S. Grimme, *ChemistryOpen* **2013**, *2*, 115.
[48] P. Wedi, M. Farizyan, K. Bergander, C. Mück-Lichtenfeld, M. van Gemmeren, *Angew. Chem. Int. Ed.* **2021**, *60*, 15641.
[49] L. Wang, B. P. Carrow, *ACS Catal.* **2019**, *9*, 6821.
[50] R. Evans, J. Sampson, L. Wang, L. Lückemeier, B. P. Carrow, *Chem. Commun.* **2021**, *57*, 9076.
[51] U. Dutta, G. Prakash, K. Devi, K. Borah, X. Zhang, D. Maiti, *Chem. Sci.* **2023**, *14*, 11381.
[52] B. E. Haines, R. Sarpong, D. G. Musaev, *J. Am. Chem. Soc.* **2018**, *140*, 10612.
[53] K. Usui, B. E. Haines, D. G. Musaev, R. Sarpong, *ACS Catal.* **2018**, *8*, 4516.
[54] C. Colletto, S. Islam, F. Juliá-Hernández, I. Larrosa, *J. Am. Chem. Soc.* **2016**, *138*, 1677.
[55] M. Steinmetz, K. Ueda, S. Grimme, J. Yamaguchi, S. Kirchberg, K. Itami, A. Studer, *Chem. Asian J.* **2012**, *7*, 1256.
[56] M. D. Lotz, N. M. Camasso, A. J. Canty, M. S. Sanford, *Organometallics* **2017**, *36*, 165.
[57] S. Porey, X. Zhang, S. Bhowmick, V. Kumar Singh, S. Guin, R. S. Paton, D. Maiti, *J. Am. Chem. Soc.* **2020**, *142*, 3762.
[58] L. Goerigk, A. Hansen, C. Bauer, S. Ehrlich, A. Najibi, S. Grimme, *Phys. chem. chem. phys.* **2017**, *19*, 32184.
[59] Y.-Q. Gong, Y.-F. Cheng, J.-M. Gu, X.-R. Hu, *Polyhedron* **1997**, *16*, 3743.
[60] T. Kang, Y. Fu, R. Li-Matsuura, A. L. Liu, T. C. Jankins, A. L. Rheingold, J. B. Bailey, M. Gembicky, P. Liu, K. M. Engle, *Organometallics* **2023**, *42*, 11.
[61] Z. Liu, X. Li, T. Zeng, K. M. Engle, *ACS Catal.* **2019**, *9*, 3260.
[62] M. Sabat, K. A. Satyshur, M. Sundaralingam, *J. Am. Chem. Soc.* **1983**, *105*, 976.
[63] M. D. Walter, R. A. Moorhouse, S. A. Urbin, P. S. White, M. Brookhart, *J. Am. Chem. Soc.* **2009**, *131*, 9055.
[64] J. C. Zhang, L. W. Wang, S. Y. Liu, F. F. Zhang, J. L. Du, L. W. Li, S. X. Wang, S. H. Li, G. Q. Zhou, *Russ. J. Coord. Chem.* **2014**, *40*, 115.
[65] J. Zhang, L. Wang, L. Li, X. Qin, X. Li, CN2010-10202987.
[66] S. Grimme, M. Steinmetz, *Phys. chem. chem. phys.* **2013**, *15*, 16031.
[67] A. Hansen, C. Bannwarth, S. Grimme, P. Petrović, C. Werlé, J.-P. Djukic, *ChemistryOpen* **2014**, *3*, 177.
[68] W. Hujo, S. Grimme, *J. Chem. Theory Comput.* **2011**, *7*, 3866.
[69] E. Caldeweyher, C. Bannwarth, S. Grimme, *J. Chem. Phys.* **2017**, *147*, 34112.

[70] J. Bogaerts, Y. Atilaw, S. Peintner, R. Aerts, J. Kihlberg, C. Johannessen, M. Erdélyi, *RSC Adv.* **2021**, *11*, 4200.
[71] N. M. Kreienborg, C. Merten, *Chem. Eur. J.* **2018**, *24*, 17948.
[72] C. Merten, *Phys. chem. chem. phys.* **2023**, *25*, 29404.
[73] T. Vermeyen, C. Merten, *Phys. chem. chem. phys.* **2020**, *22*, 15640.
[74] S. Grimme, *ChemPhysChem* **2012**, *13*, 1407-9; author reply 1405-6.
[75] H. Jacobsen, L. Cavallo, *ChemPhysChem* **2012**, *13*, 562.
[76] T. Gerlach, S. Müller, A. G. de Castilla, I. Smirnova, *Fluid Ph. Equilibria* **2022**, *560*, 113472.
[77] S. Müller, T. Nevolianis, M. Garcia-Ratés, C. Riplinger, K. Leonhard, I. Smirnova, *Fluid Ph. Equilibria* **2025**, *589*, 114250.
[78] C. Plett, M. Stahn, M. Bursch, J.-M. Mewes, S. Grimme, *J. Phys. Chem. Lett.* **2024**, *15*, 2462.
[79] R. Pollice, F. Fleckenstein, I. Shenderovich, P. Chen, *Angew. Chem. Int. Ed.* **2019**, *58*, 14281.
[80] J. Hwang, B. E. Dial, P. Li, M. E. Kozik, M. D. Smith, K. D. Shimizu, *Chem. Sci.* **2015**, *6*, 4358.
[81] M. A. Strauss, H. A. Wegner, *Eur. J. Org. Chem.* **2019**, *2019*, 295.
[82] L. Yang, C. Adam, G. S. Nichol, S. L. Cockroft, *Nat. Chem.* **2013**, *5*, 1006.
[83] C. Adam, L. Yang, S. L. Cockroft, *Angew. Chem. Int. Ed.* **2015**, *54*, 1164.
[84] R. Pollice, M. Bot, I. J. Kobylianskii, I. Shenderovich, P. Chen, *J. Am. Chem. Soc.* **2017**, *139*, 13126.
[85] V. Gorbachev, A. Savoy, A. Tsybizova, R. Pollice, L. van Tetering, J. Martens, J. Oomens, G. Berden, P. Chen, *J. Am. Chem. Soc.* **2025**.
[86] G. Bistoni, A. Altun, Z. Wang, F. Neese, *Acc. Chem. Res.* **2024**.
[87] Q. Lu, F. Neese, G. Bistoni, *Phys. chem. chem. phys.* **2019**, *21*, 11569.
[88] D. Yepes, F. Neese, B. List, G. Bistoni, *J. Am. Chem. Soc.* **2020**, *142*, 3613.
[89] W. B. Schneider, G. Bistoni, M. Sparta, M. Saitow, C. Riplinger, A. A. Auer, F. Neese, *J. Chem. Theory Comput.* **2016**, *12*, 4778.
[90] W. M. Haynes, D. R. Lide, T. J. Bruno, *CRC Handbook of Chemistry and Physics*, CRC Press, **2016**.
[91] V. Kubyshkin, P. Durkin, N. Budisa, *New J. Chem.* **2016**, *40*, 5209.
[92] C. Bannwarth, S. Ehlert, S. Grimme, *J. Chem. Theory Comput.* **2019**, *15*, 1652.
[93] L. Martínez, R. Andrade, E. G. Birgin, J. M. Martínez, *J. Comput. Chem.* **2009**, *30*, 2157.
[94] B. W. McCann, S. McFarland, O. Acevedo, *J. Phys. Chem. A* **2015**, *119*, 8724.
[95] F. H. Hodel, P. Deglmann, S. Luber, *J. Chem. Theory Comput.* **2017**, *13*, 3348.
[96] *PyQuiver*, Anderson, T.L.; Kwan, E.E.
[97] M. K. Kesharwani, B. Brauer, J. M. L. Martin, *J. Phys. Chem. A* **2015**, *119*, 1701.
[98] R. P. Bell, *Chem. Soc. Rev.* **1974**, *3*, 513.
[99] R. T. Skodje, D. G. Truhlar, *J. Phys. Chem.* **1981**, *85*, 624.
[100] J. L. Bao, D. G. Truhlar, *Chem. Soc. Rev.* **2017**, *46*, 7548.
[101] E. M. Simmons, J. F. Hartwig, *Angew. Chem. Int. Ed.* **2012**, *51*, 3066.
[102] P. L. Fernandez, A. S. Murkin, *Molecules* **2020**, *25*.
[103] T. Bhattacharya, S. Dutta, D. Maiti, *ACS Catal.* **2021**, *11*, 9702.
[104] A. Mondal, M. van Gemmeren, *Angew. Chem. Int. Ed.* **2022**, *61*, e202210825.
[105] Holleman, F. Arnold, N. Wiberg, *Lehrbuch der Anorganischen Chemie*, de Gruyter, Berlin, **2007**.
[106] D. D. Wagman, M. V. Kilday, *J. Res. Natl. Bur. Stand. A Phys. Chem.* **1973**, *77A*, 569.
[107] A. Darù, X. Hu, J. N. Harvey, *ACS omega* **2020**, *5*, 1586.
[108] L. Baldinelli, P. Belanzoni, G. Bistoni, *J. Am. Chem. Soc.* **2024**, *146*, 6016.

[109] "NIST Chemistry WebBook, SRD 69. silver", can be found under https://webbook.nist.gov/cgi/cbook.cgi?Name=silver&Units=SI.

[110] Agilent Technologies Inc., "Agilent 7890B Gas Chromatograph. Data Sheet".

[111] R. E. Plata, D. E. Hill, B. E. Haines, D. G. Musaev, L. Chu, D. P. Hickey, M. S. Sigman, J.-Q. Yu, D. G. Blackmond, *J. Am. Chem. Soc.* **2017**, *139*, 9238.

[112] D. E. Hill, K. L. Bay, Y.-F. Yang, R. E. Plata, R. Takise, K. N. Houk, J.-Q. Yu, D. G. Blackmond, *J. Am. Chem. Soc.* **2017**, *139*, 18500.

[113] E.-C. Li, G.-Q. Hu, Y.-X. Zhu, H.-H. Zhang, K. Shen, X.-C. Hang, C. Zhang, W. Huang, *Org. Lett.* **2019**, *21*, 6745.

[114] Y. Chen, J. He, C. Zhuang, Z. Liu, K. Xiao, Z. Su, X. Ren, T. Wang, *Angew. Chem. Int. Ed.* **2022**, *61*, e202207334.

[115] F. Deufel, M. van Gemmeren, *J. Chem. Educ.* **2024**, *101*, 3410.

[116] A. Mondal, M. van Gemmeren, *Angew. Chem. Int. Ed.* **2021**, *60*, 742.

[117] C. D.-T. Nielsen, J. Burés, *Chem. Sci.* **2019**, *10*, 348.

[118] J. Burés, *Angew. Chem. Int. Ed.* **2016**, *55*, 16084.

[119] D. Dalland, L. Schrecker, K. K. Hii, *Digit. Discov.* **2024**, *3*, 2118.

[120] J. Burés, *Top. Catal.* **2017**, *60*, 631.

[121] S. Hoops, S. Sahle, R. Gauges, C. Lee, J. Pahle, N. Simus, M. Singhal, L. Xu, P. Mendes, U. Kummer, *Bioinformatics* **2006**, *22*, 3067.

[122] D. E. Hill, Q. Pei, E. Zhang, J. R. Gage, J.-Q. Yu, D. G. Blackmond, *ACS Catal.* **2018**, *8*, 1528.

[123] B. J. Gorsline, L. Wang, P. Ren, B. P. Carrow, *J. Am. Chem. Soc.* **2017**, *139*, 9605.

[124] J. J. Gair, B. E. Haines, A. S. Filatov, D. G. Musaev, J. C. Lewis, *Chem. Sci.* **2017**, *8*, 5746.

[125] J. J. Gair, B. E. Haines, A. S. Filatov, D. G. Musaev, J. C. Lewis, *ACS Catal.* **2019**, *9*, 11386.

[126] H. Chen, M. Farizyan, F. Ghiringhelli, M. van Gemmeren, *Angew. Chem. Int. Ed.* **2020**, *59*, 12213.

[127] *Topspin*, Bruker Corporation.

[128] *Mnova*, Mestrelab Research.

[129] E. E. Kwan, Y. Park, H. A. Besser, T. L. Anderson, E. N. Jacobsen, *J. Am. Chem. Soc.* **2017**, *139*, 43.

[130] D. A. Singleton, A. A. Thomas, *J. Am. Chem. Soc.* **1995**, *117*, 9357.
